# Whole-Genome Sequencing Coupled to Imputation Discovers Genetic Signals for Anthropometric Traits

**DOI:** 10.1016/j.ajhg.2017.04.014

**Published:** 2017-05-25

**Authors:** Ioanna Tachmazidou, Dániel Süveges, Josine L. Min, Graham R.S. Ritchie, Julia Steinberg, Klaudia Walter, Valentina Iotchkova, Jeremy Schwartzentruber, Jie Huang, Yasin Memari, Shane McCarthy, Andrew A. Crawford, Cristina Bombieri, Massimiliano Cocca, Aliki-Eleni Farmaki, Tom R. Gaunt, Pekka Jousilahti, Marjolein N. Kooijman, Benjamin Lehne, Giovanni Malerba, Satu Männistö, Angela Matchan, Carolina Medina-Gomez, Sarah J. Metrustry, Abhishek Nag, Ioanna Ntalla, Lavinia Paternoster, Nigel W. Rayner, Cinzia Sala, William R. Scott, Hashem A. Shihab, Lorraine Southam, Beate St Pourcain, Michela Traglia, Katerina Trajanoska, Gialuigi Zaza, Weihua Zhang, María S. Artigas, Narinder Bansal, Marianne Benn, Zhongsheng Chen, Petr Danecek, Wei-Yu Lin, Adam Locke, Jian’an Luan, Alisa K. Manning, Antonella Mulas, Carlo Sidore, Anne Tybjaerg-Hansen, Anette Varbo, Magdalena Zoledziewska, Chris Finan, Konstantinos Hatzikotoulas, Audrey E. Hendricks, John P. Kemp, Alireza Moayyeri, Kalliope Panoutsopoulou, Michal Szpak, Scott G. Wilson, Michael Boehnke, Francesco Cucca, Emanuele Di Angelantonio, Claudia Langenberg, Cecilia Lindgren, Mark I. McCarthy, Andrew P. Morris, Børge G. Nordestgaard, Robert A. Scott, Martin D. Tobin, Nicholas J. Wareham, SpiroMeta Consortium, GoT2D Consortium, Paul Burton, John C. Chambers, George Davey Smith, George Dedoussis, Janine F. Felix, Oscar H. Franco, Giovanni Gambaro, Paolo Gasparini, Christopher J. Hammond, Albert Hofman, Vincent W.V. Jaddoe, Marcus Kleber, Jaspal S. Kooner, Markus Perola, Caroline Relton, Susan M. Ring, Fernando Rivadeneira, Veikko Salomaa, Timothy D. Spector, Oliver Stegle, Daniela Toniolo, André G. Uitterlinden, arcOGEN Consortium, Understanding Society Scientific Group, UK10K Consortium, Inês Barroso, Celia M.T. Greenwood, John R.B. Perry, Brian R. Walker, Adam S. Butterworth, Yali Xue, Richard Durbin, Kerrin S. Small, Nicole Soranzo, Nicholas J. Timpson, Eleftheria Zeggini

**Affiliations:** 1 The Wellcome Trust Sanger Institute, Wellcome Trust Genome Campus, Hinxton CB10 1SA, UK; 2 MRC Integrative Epidemiology Unit, School of Social and Community Medicine, University of Bristol, Bristol BS8 2BN, UK; 3 Usher Institute of Population Health Sciences & Informatics, University of Edinburgh, Edinburgh EH16 4UX, UK; 4 MRC Institute of Genetics and Molecular Medicine, University of Edinburgh, Edinburgh EH16 4UX, UK; 5 European Molecular Biology Laboratory, European Bioinformatics Institute, Wellcome Trust Genome Campus, Hinxton CB10 1SD, UK; 6 Boston VA Research Institute, Boston, MA 02130, USA; 7 BHF Centre for Cardiovascular Science, Queen’s Medical Research Institute, University of Edinburgh, Edinburgh EH16 4TJ, UK; 8 Department of Neurological, Biomedical and Movement Sciences, University of Verona, Verona 37134, Italy; 9 Department of Medical, Surgical and Health Sciences, University of Trieste, Trieste 34100, Italy; 10 Department of Nutrition and Dietetics, School of Health Science and Education, Harokopio University, Athens 17671, Greece; 11 Department of Health, National Institute for Health and Welfare, Helsinki 00271, Finland; 12 The Generation R Study Group, Erasmus Medical Center, University Medical Center, Rotterdam 3000 CA, the Netherlands; 13 Department of Epidemiology, Erasmus Medical Center, University Medical Center, Rotterdam 3000 CA, the Netherlands; 14 Department of Pediatrics, Erasmus Medical Center, University Medical Center, Rotterdam 3000 CA, the Netherlands; 15 Department of Epidemiology and Biostatistics, School of Public Health, Imperial College London, London W2 1PG, UK; 16 Department of Internal Medicine, Erasmus Medical Center, University Medical Center, Rotterdam 3000 CA, the Netherlands; 17 Department of Twin Research and Genetic Epidemiology, King’s College London, London SE1 7EH, UK; 18 William Harvey Research Institute, Barts and the London School of Medicine and Dentistry, Queen Mary University of London, London EC1M 6BQ, UK; 19 Wellcome Trust Centre for Human Genetics, University of Oxford, Oxford OX3 7BN, UK; 20 Oxford Centre for Diabetes, Endocrinology and Metabolism, University of Oxford, Churchill Hospital, Oxford OX3 7LJ, UK; 21 Division of Genetics and Cell Biology, San Raffaele Scientific Institute, Milan 20132, Italy; 22 Department of Cardiology, Ealing Hospital NHS Trust, Middlesex UB1 3EU, UK; 23 Max Planck Institute for Psycholinguistics, Nijmegen 6500, the Netherlands; 24 Renal Unit, Department of Medicine, Verona University Hospital, Verona 37126, Italy; 25 Genetic Epidemiology Group, Department of Health Sciences, University of Leicester, Leicester LE1 7RH, UK; 26 Cardiovascular Epidemiology Unit, Department of Public Health & Primary Care, University of Cambridge, Cambridge CB1 8RN, UK; 27 Faculty of Health and Medical Sciences, University of Copenhagen, Copenhagen 2200, Denmark; 28 Department of Biostatistics and Center for Statistical Genetics, University of Michigan, Ann Arbor, MI 48109, USA; 29 Department of Clinical Biochemistry, Rigshospitalet, Copenhagen University Hospital, Copenhagen 2100, Denmark; 30 McDonnell Genome Institute, Washington University School of Medicine, Saint Louis, MO 63108, USA; 31 MRC Epidemiology Unit, University of Cambridge School of Clinical Medicine, Cambridge CB2 0QQ, UK; 32 Center for Human Genetics Research, Massachusetts General Hospital, Boston, MA 02114, USA; 33 Program in Medical and Population Genetics, Broad Institute, Cambridge, MA 02142, USA; 34 Department of Medicine, Harvard University Medical School, Boston, MA 02115, USA; 35 Istituto di Ricerca Genetica e Biomedica (IRGB-CNR), Cagliari 09100, Italy; 36 Università degli Studi di Sassari, Sassari 07100, Italy; 37 Institute of Cardiovascular Science, Faculty of Population Health, University College London, London WC1E 6BT, UK; 38 Mathematical and Statistical Sciences, University of Colorado Denver, Denver, CO 80204, USA; 39 University of Queensland Diamantina Institute, Translational Research Institute, Brisbane, QLD 4072, Australia; 40 Institute of Health Informatics, University College London, London NW1 2DA, UK; 41 School of Medicine and Pharmacology, The University of Western Australia, Crawley, WA 6009, Australia; 42 Department of Endocrinology and Diabetes, Sir Charles Gairdner Hospital, Nedlands, WA 6009, Australia; 43 The National Institute for Health Research Blood and Transplant Unit (NIHR BTRU) in Donor Health and Genomics at the University of Cambridge, Cambridge CB1 8RN, UK; 44 Li Ka Shing Centre for Health Information and Discovery, The Big Data Institute, University of Oxford, Oxford OX3 7BN, UK; 45 Oxford NIHR Biomedical Research Centre, Churchill Hospital, Oxford OX3 7LJ, UK; 46 Department of Biostatistics, University of Liverpool, Liverpool L69 3GL, UK; 47 Estonian Genome Center, University of Tartu, Tartu, Tartumaa 51010, Estonia; 48 National Institute for Health Research (NIHR) Leicester Respiratory Biomedical Research Unit, Glenfield Hospital, Leicester LE3 9QP, UK; 49 D2K Research Group, School of Social and Community Medicine, University of Bristol, Bristol BS8 2BN, UK; 50 Imperial College Healthcare NHS Trust, London W2 1NY, UK; 51 Division of Nephrology and Dialysis, Columbus-Gemelli University Hospital, Catholic University, Rome 00168, Italy; 52 Medical Genetics, Institute for Maternal and Child Health IRCCS “Burlo Garofolo”, Trieste 34100, Italy; 53 Vth Department of Medicine, Medical Faculty Mannheim, Heidelberg University, Mannheim 68167, Germany; 54 National Heart and Lung Institute, Imperial College London, Hammersmith Hospital Campus, London W12 0NN, UK; 55 Institute for Molecular Medicine (FIMM), University of Helsinki, Helsinki 00290, Finland; 56 University of Cambridge Metabolic Research Laboratories, and NIHR Cambridge Biomedical Research Centre, Wellcome Trust-MRC Institute of Metabolic Science, Addenbrooke’s Hospital, Cambridge CB2 0QQ, UK; 57 Lady Davis Institute for Medical Research, Jewish General Hospital, Montréal, QC H3T 1E2, Canada; 58 Department of Epidemiology, Biostatistics and Occupational Health, McGill University, Montréal, QC H3A 1A2, Canada; 59 Department of Oncology, McGill University, Montréal, QC H2W 1S6, Canada; 60 Department of Haematology, University of Cambridge, Cambridge CB2 0AH, UK

**Keywords:** UK10K, genetic association study, next-generation whole-genome sequencing, imputation, UK Biobank, anthropometry, DXA traits

## Abstract

Deep sequence-based imputation can enhance the discovery power of genome-wide association studies by assessing previously unexplored variation across the common- and low-frequency spectra. We applied a hybrid whole-genome sequencing (WGS) and deep imputation approach to examine the broader allelic architecture of 12 anthropometric traits associated with height, body mass, and fat distribution in up to 267,616 individuals. We report 106 genome-wide significant signals that have not been previously identified, including 9 low-frequency variants pointing to functional candidates. Of the 106 signals, 6 are in genomic regions that have not been implicated with related traits before, 28 are independent signals at previously reported regions, and 72 represent previously reported signals for a different anthropometric trait. 71% of signals reside within genes and fine mapping resolves 23 signals to one or two likely causal variants. We confirm genetic overlap between human monogenic and polygenic anthropometric traits and find signal enrichment in *cis* expression QTLs in relevant tissues. Our results highlight the potential of WGS strategies to enhance biologically relevant discoveries across the frequency spectrum.

## Introduction

The escalating global epidemic of overweight and obesity can be ascribed to a complex interplay between environmental and genetic factors. Body size, shape, and composition are anthropometric measures correlated with obesity and patterns of fat deposition and are associated with important metabolic health outcomes.^1^, ^2^, ^3^ Large-scale genome-wide association studies (GWASs) for body mass index (BMI), waist to hip ratio, and height have to date focused on the role of common-frequency variants and have unveiled numerous associations that explain a modest proportion of trait variance;^4^, ^5^, ^6^ the role of low-frequency variants has not been systematically explored across the entire genome.

The application of whole-genome sequencing (WGS) at a population scale and generation of high performance imputation reference panels allows GWASs to systematically evaluate variation across the low- and common-frequency minor allele frequency (MAF) spectra. Here, we assessed the contribution of 15,844,966 sequence variants to 12 anthropometric traits of medical relevance using a hybrid approach of cohort-wide low-depth WGS^7^ and imputation based on a sequence-based reference panel comprising 9,746 haplotypes^8^ in a discovery set of 57,129 individuals (stage 1, Table S1). We followed up suggestive association signals at p ≤ 10^−5^ in 210,823 individuals (stage 2, Table S1) of European descent and identify 106 previously unreported signals for anthropometric traits.

## Material and Methods

### Sequence Data Production

Low-read depth (∼7×) WGS was performed in two UK cohorts, the St Thomas’ Twin Registry^9^ (TwinsUK; n = 1,990) and the Avon Longitudinal Study of Parents and Children^10^ (ALSPAC; n = 2,040) as part of the UK10K project.^7^ Methods for the generation of these data are described in detail in Walter et al.^7^ and Huang et al.^8^ In brief, low-coverage WGS was performed at both the Wellcome Trust Sanger Institute and the Beijing Genomics Institute. Sequencing reads that failed QC were removed and the rest were aligned to the GRCh37 human reference. Further processing to improve SNP and INDEL calling included realignment around known indels, base quality score recalibration, addition of BAQ tags, merging, and duplicate marking using GATK, Picard, and samtools. SNPs and indels were called using samtools/bcftools by pooling the alignments from 3,910 individual low-coverage BAM files. All-samples and all-sites genotype likelihood files (bcf) were created with samtools mpileup. Variants were then called using bcftools to produce a VCF file.

After post-calling filtering, variant quality score recalibration (VQSR) filtering was used to filter sites. VQSLOD scores are calibrated by the number of truth sites retained when sites with a VQSLOD score below a given threshold are filtered out. For SNPs and INDELs, a truth sensitivity of 99.5% and 97% was selected, respectively. Sites that did not fail a number of further filters (DP, MQ, AC, AN, LowQual, MinVQSLOD, BaseQRankSum, Dels, FS, HRun, HaplotypeScore, InbreedingCoeff, MQ0, MQRankSum, QD, ReadPosRankSum) were marked as PASS and brought forward to the genotype refinement stage.

Low-quality samples were identified by comparing the samples to their GWAS genotypes using ∼20,000 sites on chromosome 20. Comparing the raw genotype calls to existing GWAS data, a total of 112 samples were removed for one or more of the following causes: (1) high overall discordance to SNP array data, (2) heterozygosity rate > 3 standard deviations (SD) from population mean, (3) no SNP array data available for that sample, or (4) sample below 4× mean coverage. Overall, 3,798 samples were brought forward to the genotype refinement step.

Missing and low-confidence genotypes in the filtered VCFs were filtered out through an imputation procedure with BEAGLE. Additional sample-level QC steps were carried out on refined genotypes, leading to the exclusion of additional 17 samples for one or more of the following causes: (1) non-reference discordance with GWAS SNP data > 5%, (2) contamination identified by multiple relations (>25 to other samples with IBS > 0.125), or (3) failed sex check. A final set of 3,781 samples (1,854 TwinsUK and 1,927 ALSPAC) in VCF files were submitted to the European Genome-phenome Archive (EGA).

### Cohort Descriptions

We consider 12 anthropometric traits: BMI, weight, height, waist circumference, hip circumference, waist to hip ratio, total fat mass, total lean mass, and trunk fat mass. Waist circumference, hip circumference, and waist to hip ratio were also adjusted for BMI. Our discovery stage consisted of 3 WGS and 20 GWAS datasets genotyped on a variety of genotyping platforms (Table S2, Figure S1). The WGS sets are from two UK cohorts, TwinsUK^9^ (EGAS00001000108) and ALSPAC^10^ (EGAS00001000090) as part of the UK10K project,^7^ and from a Finnish cohort.^11^ Each of the 20 GWAS datasets was imputed on the combined UK10K and 1000 Genomes Project imputation panel (EGAS00001000713), comprised of 4,873 WGSed individuals.^8^ The imputation of GWAS data was conducted as follows. Raw data were obtained genome-wide from each individual study, having undergone study-specific quality control. The data were prephased with SHAPEIT v.2 and the phased genotypes were then imputed to the combined UK10K and 1000 Genomes Project haplotype reference panel.^8^ Imputation was carried out with IMPUTE v.2 with standard settings.^12^ In total, GWAS data contributed up to 52,339 individuals of European ancestry (UK, Italy, Greece, Germany, the Netherlands) (Tables S1 and S2). Therefore, our discovery phase included up to 57,129 individuals from 23 cohorts of European origin. We followed up the top signals de novo and in silico. Follow-up through de novo genotyping was sought in up to 37,851 UK^13^ and Danish samples^14^ using Sequenom genotyping (Supplemental Data). In silico follow-up was sought in up to 175,318 Europeans, the majority of whom were imputed on the combined UK10K and 1000 Genomes Project panel (Figure S1; Table S2). Descriptions of each of the cohorts are given in the Supplemental Data.

### Datasets Used for mQTL and eQTL Analyses

#### ARIES Data

The Accessible Resource for Integrative Epigenomic Studies (ARIES) dataset represents genome-wide DNA methylation levels on ALSPAC samples selected from 1,018 mother-child pairs at three time points in children and two time points in their mothers from cord blood drawn from the umbilical cord upon delivery or peripheral blood^15^ using different cell types. The DNA methylation data were corrected for cellular heterogeneity (Supplemental Data).

#### MuTHER-ALSPAC Data

The UK10K MuTHER-ALSPAC gene expression dataset is comprised of the subset of UK10K individuals with microarray expression profiles available from the TwinsUK MuTHER study^16^ and ALSPAC expression study.^17^ Complete details can be found in Grundberg et al.^16^ and Bryois et al.^17^ Both datasets were profiled on the same Illumina HT12v3 array in the same facility within the same year. Expression data were available for 823 lymphoblastoid cell lines (LCL) (394 TwinsUK/MuTHER and 429 ALSPAC) and 2 primary tissues in MuTHER/TwinsUK only (391 subcutaneous fat and 367 skin). All individuals were unrelated.

### Phenotype Preparation Protocol

A standardized protocol for preparation of phenotypes was applied to each cohort, as follows. Female and male participants were divided into separate groups and transformations were undertaken in a sex-specific manner. Outliers greater than 5 SD were manually checked for data entry errors. Outliers greater than 3, 4, or 5 SD (depending on trait and cohort) from the mean were removed and raw phenotypes were then transformed to obtain a normal distribution using an inverse normal transformation. Subsequently, the transformed traits were regressed on covariates and the resulting residuals were standardized to have a mean of 0 and a SD of 1. Females and males were standardized separately before being combined. Covariates (age and age^2^) were fitted as fixed effects. The DXA traits were further adjusted for height, whereas waist circumference, hip circumference, and waist to hip ratio were also adjusted for BMI. Analyses of all anthropometric traits in GoT2D were performed with similar methodology to previous publications by the GIANT Consortium. Within each study, height was first adjusted for age and sex, as well as relevant study-specific covariates such as principal components in a linear regression model, and residuals were standardized. Similarly, all obesity measures (waist circumference, hip circumference, and waist to hip ratio) were adjusted for age, age^2^, sex, and study-specific covariates in linear regression, and the residuals were inverse normalized. Information on trait measurements and units is summarized in Table S2.

### Single-Variant Tests

Assuming an additive genetic model, we used the likelihood ratio test within a linear regression framework to model relationships between standardized traits, residualized for relevant covariates, and genetic variants. To account for the genotype uncertainty that might arise from sequencing and imputation, we used genotype dosages, where each genotype was expressed on a quantitative scale between [0:2] (using in SNPTEST^18^ the function -method expected). Cohorts that contained related samples were analyzed using GEMMA^19^ or EMMAX,^20^ standard linear mixed models that control for family and cryptic relatedness (Table S2). Only variants with MAF ≥ 0.1%, minor allele count (MAC) ≥ 4, imputation quality score ≥ 0.4 (Figure S2), and Hardy-Weinberg equilibrium (HWE) p ≥ 10^−6^ were analyzed.

### Meta-analysis Strategy

Summary statistics from individual studies (filtered for HWE, imputation quality score, MAC, and MAF) were combined using fixed-effect inverse variance meta-analysis implemented in METAL^21^ software package. We discarded any variants whose signal was from a single cohort and also any variants that were not successfully analyzed in any of the four ALSPAC and TwinsUK cohorts. None of the traits showed evidence of inflation due to population stratification (genomic control inflation factors estimated close 1; Figures S3–S14). The variance explained by each SNP was calculated using the weighted effect allele frequency (f) and beta (β) from the overall meta-analysis using the formula β^2^(1 − f)2f.

### Clumping of Single Point Summary Statistics

We next applied a clumping procedure to represent each signal from the association analysis as a clump of correlated variants. This is achieved by assigning sets of variants to discrete LD bins if their pairwise LD is r^2^ ≥ 0.2 and if they are within 500 kb. For each LD bin, the variant with the greatest evidence for association with the trait in question was considered as the representative or index variant for that locus.

### Annotation of Index Variants for Previously Reported Loci

A list of previously identified, GWAS-significant (p ≤ 5 × 10^−8^) anthropometric and obesity signals were collected from the NHGRI-EBI GWAS catalog^22^ (accessed 4 March 2015, version 1.0). In addition to the GWAS catalog, our list contained signals reported in the most recent anthropometric studies published by the GIANT consortium.^4^, ^5^, ^6^ From these results, any signal reaching genome-wide significance, either in the sex-specific or in sex-combined analyses, was included in our positive control list with the lowest reported p value. The total fat mass variants that we regard as “known” are the total fat percentage variants reported previously^23^, ^24^ while the total lean mass variants reported in the literature are for lean body mass.^25^ During the course of the study, we updated our positive control list using the GWAS catalog and by manual curation of all associations reported in the literature reaching the same genome-wide significance cutoff.

### Conditional Analysis

Conditional single-variant association analyses were carried out to investigate statistical independence between index variants from the clumping procedure and previously reported variants. Associations of SNPs with the respective quantitative trait were conditioned on all previously reported variants within 1 Mb of the index variant. The conditional analysis was performed independently for each discovery phase cohort for which we had access to the raw genotypes (17 out of a total 23 cohorts) and a meta-analysis was conducted. A variant was considered independent if it had a conditional p value ≤ 10^−5^ or a p value difference between conditional and unconditional analysis of less than 2 orders of magnitude. Variants were classified as known (denoting either a previously reported variant, or a variant for which the association signal disappears after conditioning on a previously reported locus) or newly identified (denoting a variant that is conditionally independent of previously reported loci).

### Genome-wide Significance Threshold

We consider p ≤ 5 × 10^−8^ as genome-wide significant. To account for testing of multiple phenotypes, we used the biggest cohort with all phenotypes available (ALSPAC) and the eigenvalues of the correlation matrix of the 12 anthropometric traits tested^26^ to calculate the effective number of independent phenotypes as 4.482. This yields a Bonferroni-corrected threshold that controls the FWER at 5% as 0.05/4.482. We used this threshold, as well as a 5% false discovery rate (FDR), for enrichment of association signal in discovery and monogenic and syndromic disorder-associated genes.

### Fine Mapping

For both newly identified (Tables 1, 2, and S3) and previously reported (those with p ≤ 5 × 10^−8^ in Table S4) variants, we constructed regions for fine mapping, by taking a window of at least 0.1 centimorgans (HapMap estimates following previous suggestions^27^) either side of the variant. The region was extended to the furthest variant with r^2^ > 0.1 with the index variant within a 1 Mb window. For each region we implemented the Bayesian fine-mapping method CAVIARBF,^28^ which uses association summary statistics and correlations among variants to calculate Bayes’ factors and posterior probabilities of each variant being causal. We assumed a single causal variant in each region and calculated 95% credible sets.

**Table 1:** Genome-wide Significant Associations at Newly Identified Loci

**SNP**	**Trait**	**Chr:position**	**Nearest Gene**	**Effect/Other Allele**	**Stage 1**						**Stage 2**						**Stage 1 + Stage 2**						**Variance Explained (%)**
					**Frequency (Effect Allele)**	**Beta (SE)**	**p Value**	**n**	**I**^**2**^	***P***_**het**_	**Frequency (Effect Allele)**	**Beta (SE)**	**p Value**	**n**	**I**^**2**^	***P***_**het**_	**Frequency (Effect Allele)**	**Beta (SE)**	**p Value**	**n**	**I**^**2**^	***P***_**het**_	
**Low-Frequency or Rare**																							

rs202238847	height	3: 49,263,637	*CCDC36*	C/CT	0.021	0.1091 (0.0233)	2.83 × 10^−6^	51,309	26.8	0.132	0.023	0.0908 (0.0129)	2.04 × 10^−12^	134,797	0.0	1.000	0.022	0.0951 (0.0113)	3.76 × 10^−17^	186,106	24.3	0.153	0.0787

**Common**																							

rs1264622	height	6: 30,256,936	*HLA-L/HCG17/HCG18*	T/C	0.190	0.0455 (0.0087)	1.76 × 10^−7^	50,372	13.0	0.296	0.202	0.0257 (0.0047)	4.61 × 10^−8^	134,797	0.0	1.000	0.199	0.0302 (0.0041)	3.05 × 10^−13^	185,169	22.9	0.172	0.0291
rs11042397	hip	11: 9,524,255	*ZNF143*	T/C	0.056	0.0763 (0.0150)	3.56 × 10^−7^	45,588	2.3	0.429	0.057	0.0386 (0.0082)	2.68 × 10^−6^	134,797	0.0	1.000	0.056	0.0473 (0.0072)	5.20 × 10^−11^	180,385	18.3	0.226	0.0238
rs13213884	height	6: 141,665,522	*RP11-63E9.1*	T/C	0.247	0.0419 (0.0074)	1.57 × 10^−8^	51,309	49.5	0.007	0.257	0.0176 (0.0043)	4.68 × 10^−5^	134,797	0.0	1.000	0.254	0.0238 (0.0037)	1.94 × 10^−10^	186,106	56.2	0.001	0.0215
rs12424892	height	12: 132,623,389	*DDX51*	C/G	0.153	0.0457 (0.0095)	1.60 × 10^−6^	44,180	0.0	0.907	0.148	0.0241 (0.0053)	5.80 × 10^−6^	134,797	0.0	1.000	0.149	0.0292 (0.0046)	3.06 × 10^−10^	178,977	0.0	0.731	0.0216
rs35863206	height	11: 101,055,183	*RP11-788M5.4*	C/CAG	0.222	−0.0384 (0.0082)	2.77 × 10^−6^	45,588	21.8	0.190	0.224	−0.0185 (0.0046)	5.17 × 10^−5^	134,797	0.0	1.000	0.224	−0.0232 (0.004)	5.91 × 10^−9^	180,385	31.0	0.093	0.0187

SNP positions are reported according to build 37 and their alleles are coded based on the positive strand. The reported gene is the closest in physical distance. Association p values are based on the inverse-variance weighted meta-analysis model (fixed effects). Effect sizes are measured in standard deviation units. Abbreviations are as follows: BMI, body mass index; SNP, single-nucleotide polymorphism; Beta, effect size; SE, standard error; n, sample size; I^2^, measure of heterogeneity (based on Cochran’s Q-test for heterogeneity) that indicates the percentage of variance in a meta-analysis that is attributable to study heterogeneity; *P*_*het*_, p value assessing evidence of heterogeneity as reported by METAL.

**Table 2:** Genome-wide Significant Independent Associations at Established Anthropometric Trait Loci

**SNP**	**Trait**	**Chr:position**	**Nearest Gene**	**Effect/Other Allele**	**Stage 1**						**Stage 2**						**Stage 1 + Stage 2**						**Variance Explained (%)**
					**Frequency (Effect Allele)**	**Beta (SE)**	**p Value**	**n**	**I**^**2**^	***P***_**het**_	**Frequency (Effect Allele)**	**Beta (SE)**	**p Value**	**n**	**I**^**2**^	***P***_**het**_	**Frequency (Effect Allele)**	**Beta (SE)**	**p Value**	**n**	**I**^**2**^	***P***_**het**_	
**Low-Frequency or Rare**																							

rs62621197	height	19: 8,670,147	*ADAMTS10*	T/C	0.038	−0.1356 (0.0202)	2.13 × 10^−11^	47,739	0.0	0.657	0.042	−0.1398 (0.0086)	1.87 × 10^−59^	204,461	0.0	0.529	0.042	−0.1392 (0.0079)	3.22 × 10^−69^	252,200	0.0	0.738	0.1542
rs62107261	BMI	2: 422,144	*AC105393.2*	C/T	0.049	−0.0712 (0.0169)	2.57 × 10^−5^	47,476	29.7	0.094	0.047	−0.0763 (0.0076)	9.32 × 10^−24^	208,397	0.0	0.461	0.047	−0.0754 (0.0069)	1.27 × 10^−27^	255,873	22.6	0.146	0.0510
rs114976626	height	19: 56,001,665	*SSC5D*	T/C	0.029	−0.1109 (0.0218)	3.87 × 10^−7^	44,180	0.0	0.691	0.026	−0.0915 (0.0119)	1.73 × 10^−14^	134,797	0.0	1.000	0.027	−0.096 (0.0105)	5.00 × 10^−20^	178,977	0.0	0.712	0.0479
rs183677281	height	1: 218,537,632	*TGFB2*	C/T	0.031	0.0993 (0.0225)	9.78 × 10^−6^	44,639	0.0	0.937	0.026	0.0618 (0.0126)	9.80 × 10^−7^	134,797	0.0	1.000	0.027	0.0708 (0.011)	1.24 × 10^−10^	179,436	0.0	0.885	0.0261
rs62038850	height	16: 2,262,987	*PGP*	A/G	0.023	0.1046 (0.0234)	7.48 × 10^−6^	51,309	8.6	0.349	0.025	0.0605 (0.0127)	1.84 × 10^−6^	122,318	0.0	1.000	0.024	0.0706 (0.0112)	2.45 × 10^−10^	173,627	15.0	0.264	0.0237
rs142854193	height	7: 33,045,510	*FKBP9*	T/C	0.025	0.1058 (0.0232)	5.24 × 10^−6^	51,309	0.0	0.720	0.022	0.06 (0.0138)	1.36 × 10^−5^	134,797	0.0	1.000	0.023	0.0719 (0.0119)	1.31 × 10^−9^	186,106	0.0	0.593	0.0227

**Common**																							

rs61734601	height	11: 67,184,725	*PPP1CA/CARNS1*	A/G	0.077	−0.0877 (0.0138)	1.96 × 10^−10^	45,588	14.1	0.282	0.083	−0.1177 (0.0057)	1.19 × 10^−93^	204,253	47.6	0.106	0.082	−0.1133 (0.0053)	1.38 × 10^−101^	249,841	29.5	0.088	0.1933
rs41271299	height	6: 19,839,415	*ID4*	T/C	0.054	0.1322 (0.0157)	4.25 × 10^−17^	51,309	51.1	0.005	0.056	0.1209 (0.0077)	3.86 × 10^−56^	175,844	0.0	0.502	0.055	0.1231 (0.0069)	1.90 × 10^−71^	227,153	44.8	0.010	0.1583
rs72755233	height	15: 100,692,953	*ADAMTS17*	A/G	0.112	−0.082 (0.0117)	2.10 × 10^−12^	44,180	0.0	0.679	0.112	−0.0842 (0.006)	3.16 × 10^−45^	134,635	0.0	1.000	0.112	−0.0837 (0.0053)	5.42 × 10^−56^	178,815	0.0	0.740	0.1394
rs73175572	height	3: 185,490,184	*IGF2BP2*	G/A	0.125	0.0783 (0.0104)	5.62 × 10^−14^	45,588	31.5	0.094	0.112	0.0626 (0.0061)	8.09 × 10^−25^	134,797	0.0	1.000	0.115	0.0666 (0.0053)	8.27 × 10^−37^	180,385	32.1	0.084	0.0903
rs6930571	height	6: 32,383,208	*BTNL2*	T/G	0.166	0.0561 (0.010)	2.03 × 10^−8^	42,873	0.0	0.787	0.182	0.0336 (0.0049)	6.61 × 10^−12^	134,462	0.0	1.000	0.179	0.0379 (0.0044)	6.01 × 10^−18^	177,335	0.0	0.563	0.0422
rs3888183	height	10: 121,604,702	*MCMBP*	T/C	0.120	−0.0549 (0.0104)	1.50 × 10^−7^	45,588	0.0	0.898	0.118	−0.0337 (0.0059)	8.86 × 10^−9^	134,797	0.0	1.000	0.118	−0.0388 (0.0051)	3.29 × 10^−14^	180,385	0.0	0.782	0.0314
rs35279483	height	12: 23,996,141	*SOX5*	C/CA	0.401	−0.0313 (0.007)	6.71 × 10^−6^	45,588	0.0	0.717	0.402	−0.0232 (0.0039)	1.83 × 10^−9^	134,797	0.0	1.000	0.402	−0.0251 (0.0034)	1.00 × 10^−13^	180,385	0.0	0.707	0.0303
rs2003476	BMI	19: 18,806,668	*CRTC1*	C/T	0.400	−0.0341 (0.007)	1.12 × 10^−6^	45,341	7.3	0.366	0.406	−0.0218 (0.0039)	3.31 × 10^−8^	134,509	0.0	1.000	0.404	−0.0248 (0.0034)	5.89 × 10^−13^	179,850	12.7	0.296	0.0296
rs4360494	height	1: 38,455,891	*SF3A3*	G/C	0.454	0.033 (0.0069)	1.78 × 10^−6^	45,588	15.5	0.265	0.445	0.021 (0.0038)	3.23 × 10^−8^	134,797	0.0	1.000	0.447	0.0238 (0.0033)	8.98 × 10^−13^	180,385	19.6	0.211	0.0280
rs78281959	height	7: 148,772,669	*ZNF786*	T/C	0.065	0.0587 (0.0131)	7.55 × 10^−6^	51,309	10.3	0.327	0.062	0.0439 (0.0079)	2.77 × 10^−8^	134,797	0.0	1.000	0.063	0.0478 (0.0068)	1.56 × 10^−12^	186,106	9.6	0.334	0.0268
rs62065847	waist	17: 46,593,125	*HOXB1*	C/T	0.487	−0.0299 (0.0067)	8.15 × 10^−6^	45,996	0.0	0.523	0.485	−0.0197 (0.0039)	3.23 × 10^−7^	134,798	0.0	1.000	0.486	−0.0222 (0.0033)	2.86 × 10^−11^	180,794	0.0	0.474	0.0246
rs13059073	height	3: 55,491,810	*WNT5A*	C/T	0.453	0.0288 (0.0064)	6.82 × 10^−6^	51,309	0.0	0.982	0.456	0.0192 (0.0038)	4.52 × 10^−7^	134,797	0.0	1.000	0.455	0.0217 (0.0033)	3.23 × 10^−11^	186,106	0.0	0.967	0.0234
rs4303473	height	16: 84,901,475	*CRISPLD2*	C/G	0.388	0.032 (0.0066)	1.23 × 10^−6^	51,309	0.0	0.855	0.377	0.0188 (0.0039)	1.60 × 10^−6^	134,797	0.0	1.000	0.380	0.0222 (0.0034)	4.08 × 10^−11^	186,106	0.0	0.739	0.0232
rs16888802	height	4: 13,537,668	*LINC01097*	G/T	0.1787	0.0433 (0.0086)	4.57 × 10^−7^	51,309	24.9	0.151	0.175	0.0231 (0.005)	3.19 × 10^−6^	134,615	0.0	1.000	0.176	0.0282 (0.0043)	5.49 × 10^−11^	185,924	32	0.0796	0.0231
rs56130800	waist	11: 43,729,853	*RP11-472I20.4/ HSD17B12*	A/G	0.318	0.0367 (0.0073)	4.16 × 10^−7^	44,742	0.0	1.000	0.317	0.0191 (0.0041)	4.08 × 10^−6^	134,798	0.0	1.000	0.317	0.0234 (0.0036)	7.52 × 10^−11^	179,540	0.0	0.976	0.0237
rs2122823	WHR	7: 25,939,161	*CTD-2227E11.1*	T/C	0.209	0.0465 (0.0099)	2.66 × 10^−6^	32,507	0.0	0.789	0.211	0.0234 (0.0048)	9.97 × 10^−7^	134,795	0.0	1.000	0.211	0.0278 (0.0043)	1.14 × 10^−10^	167,302	0.0	0.523	0.0257
rs1848053	height	15: 48,947,962	*RP11-227D13.1*	G/A	0.248	−0.0385 (0.0075)	3.16 × 10^−7^	51,309	0.0	0.933	0.248	−0.0194 (0.0044)	1.24 × 10^−5^	134,797	0.0	1.000	0.248	−0.0243 (0.0038)	2.00 × 10^−10^	186,106	0.0	0.747	0.0220
rs12591979	height	15: 89,309,892	*RP11-343B18.2*	C/G	0.162	−0.0416 (0.0094)	9.22 × 10^−6^	45,588	0.0	0.889	0.165	−0.0236 (0.0052)	4.86 × 10^−6^	134,797	0.0	1.000	0.164	−0.0278 (0.0045)	8.06 × 10^−10^	180,385	0.0	0.788	0.0212
rs57158761	height	3: 185,371,172	*IGF2BP2*	G/A	0.445	−0.0301 (0.0068)	9.73 × 10^−6^	45,588	0.0	0.857	0.435	−0.0174 (0.0038)	5.20 × 10^−6^	134,797	0.0	1.000	0.437	−0.0205 (0.0033)	8.35 × 10^−10^	180,385	0.0	0.756	0.0207
rs765876	BMI	6: 143,185,891	*HIVEP2*	G/A	0.476	−0.0297 (0.0069)	1.52 × 10^−5^	44,092	33.1	0.086	0.491	−0.0177 (0.0039)	4.56 × 10^−6^	134,509	0.0	1.000	0.488	−0.0206 (0.0034)	9.64 × 10^−10^	178,601	35.1	0.066	0.0212
rs2808290	height	10: 27,900,882	*PPP1CA/CARNS1*	T/C	0.499	0.0308 (0.0064)	1.58 × 10^−6^	51,309	12.7	0.296	0.503	0.016 (0.0038)	2.63 × 10^−5^	134,797	0.0	1.000	0.502	0.0198 (0.0033)	1.34 × 10^−9^	186,106	22.3	0.175	0.0196
rs116878242	height	17: 70,002,330	*ID4*	A/G	0.071	0.0688 (0.0126)	4.34 × 10^−8^	51,309	0.0	0.733	0.077	0.0224 (0.0067)	7.84 × 10^−4^	167,024	0.0	0.616	0.075	0.0326 (0.0059)	3.14 × 10^−8^	218,333	16.9	0.233	0.0148

SNP positions are reported according to build 37 and their alleles are coded based on the positive strand. The reported gene is the closest in physical distance. Association p values are based on the inverse-variance weighted meta-analysis model (fixed effects). Effect sizes are measured in standard deviation units. Abbreviations are as follows: BMI, body mass index; SNP, single-nucleotide polymorphism; Beta, effect size; SE, standard error; n, sample size; I^2^, measure of heterogeneity (based on Cochran’s Q-test for heterogeneity) that indicates the percentage of variance in a meta-analysis that is attributable to study heterogeneity; *P*_*het*_, p value assessing evidence of heterogeneity as reported by METAL.

To inform the prediction of causal variants using functional prediction information, we also applied a fine-mapping method that assigns a relative “probability of regulatory function” (PRF) score among candidate causal variants, reweighting association statistics based on epigenomic annotations. In brief, we collected a set of 70 genomic and epigenomic annotations, primarily Gencode (v.19) gene annotations, FANTOM transcription start sites and enhancers,^29^, ^30^ Roadmap Epigenomics histone marks, DNase hypersensitivity, and ChromHMM genome segmentations for the lymphoblastoid cell line epigenome (GM12878).^31^, ^32^ We used fgwas^33^ to train a Bayesian hierarchical model to compute enrichment of eQTLs in these annotations based on summary statistics from the Geuvadis RNA-sequencing project.^34^ We used forward stepwise selection followed by cross-validation to arrive at a combined model with 37 annotations and their associated enrichments. The respective annotations from 119 Roadmap epigenomes were used to compute PRF scores for each GWAS variant in each of the 119 epigenomes. At each locus we selected the top four epigenomes based on the maximum regulatory score among variants in the 95% credible set and examined the regulatory annotations for variants in the credible set (Table S5, Figure S15). We also produced Genomic Evolutionary Rate Profiling (GERP) scores^35^, ^36^ as a measure of cross-species conservation of the sequences around each identified association (Figure S16).

### Genetic Correlation

**Figure 1:**
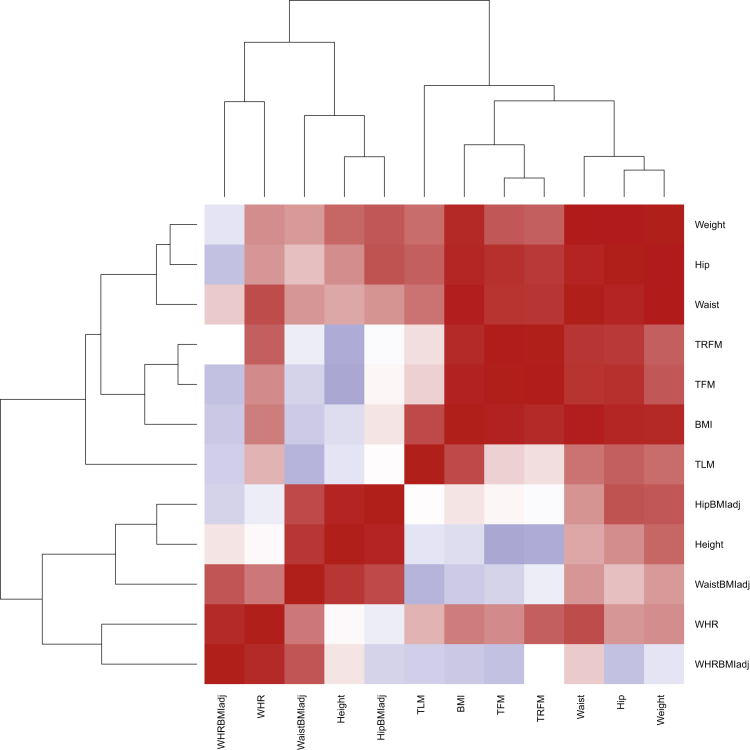
Heatmap of Pairwise Genetic Correlation Estimates between Anthropometric Traits Correlation estimates with their 95% confidence intervals and 5% FDR q values across all 66 possible pairs are given in Table S6. Abbreviations are as follows: BMI, body mass index; WHR, waist to hip ratio; WaistBMIadj, waist circumference adjusted for BMI; HipBMIadj, hip circumference adjusted for BMI; WHRBMIadj, waist to hip ratio adjusted for BMI; TFM, total fat mass; TLM, total lean mass; TRFM, trunk fat mass.

To investigate the genetic correlation between the 12 anthropometric traits studied here, we ran the LD Score^37^ method that uses genome-wide summary statistics (independent of p value thresholds) and LD estimates between variants while accounting for sample overlap. We used summary statistics from our discovery phase and LD Score restricts analyses to common variants to avoid biases due to inherent model assumptions (Figure 1, Table S6).

### Enrichment of Association Signal

To evaluate enrichment of association signal in the meta-analysis, we used the binomial test to determine whether the observed number of variants with p value ≤ 10^−5^ is higher than expected by chance. We performed this test on all independent variants (r^2^ < 0.2) present in the meta-analysis results and also after excluding any previously identified variants (stringently defined as all variants within 1 Mb window centered around previously reported variants) (Figure S17). We also tested for enrichment within different MAF categories (0.1% ≤ MAF ≤ 1%, 1% < MAF ≤ 5%, and MAF > 5%) (Figure S18).

To identify approximately independent variants, we used a greedy selection strategy that processed variants sorted by their association p value. We first retained the variant with the greatest evidence of association and then filtered out any other variants linked to it at an r^2^ threshold of 0.2 (calculated from the combined ALSPAC and TwinsUK WGS data using the PLINK software^38^) and then retained the next most strongly associated variant that has not yet been filtered and repeat this process until there are no further unfiltered variants remaining.

### Enrichment of Association Signal in Monogenic and Syndromic Genes Associated with Obesity, Height, and Lipodystrophy

We examined whether the meta-analysis association signals cluster near biologically relevant genes, specifically (1) genes mutated in human syndromes characterized by abnormal skeletal growth, (2) genes whose mutations lead to known human obesity-associated genetic disorders and syndromes, and (3) Mendelian lipodystrophy-associated genes. To this end, we used 241 abnormal skeletal/growth-associated genes identified by Lango Allen et al.^39^ (see Lango Allen’s Table S10) and 32 obesity-associated genes (separated into 6 monogenic and 26 syndromic genes, i.e., obesity with developmental delay or dysmorphology) identified via the OMIM database using the keywords obesity, growth, size, and adipose tissue. The results were manually curated to identify 32 genes whose variation directly leads to human obesity (Table S7) and 15 OMIM genes with lipodystrophy morbidity (Table S8).

**Figure 2:**
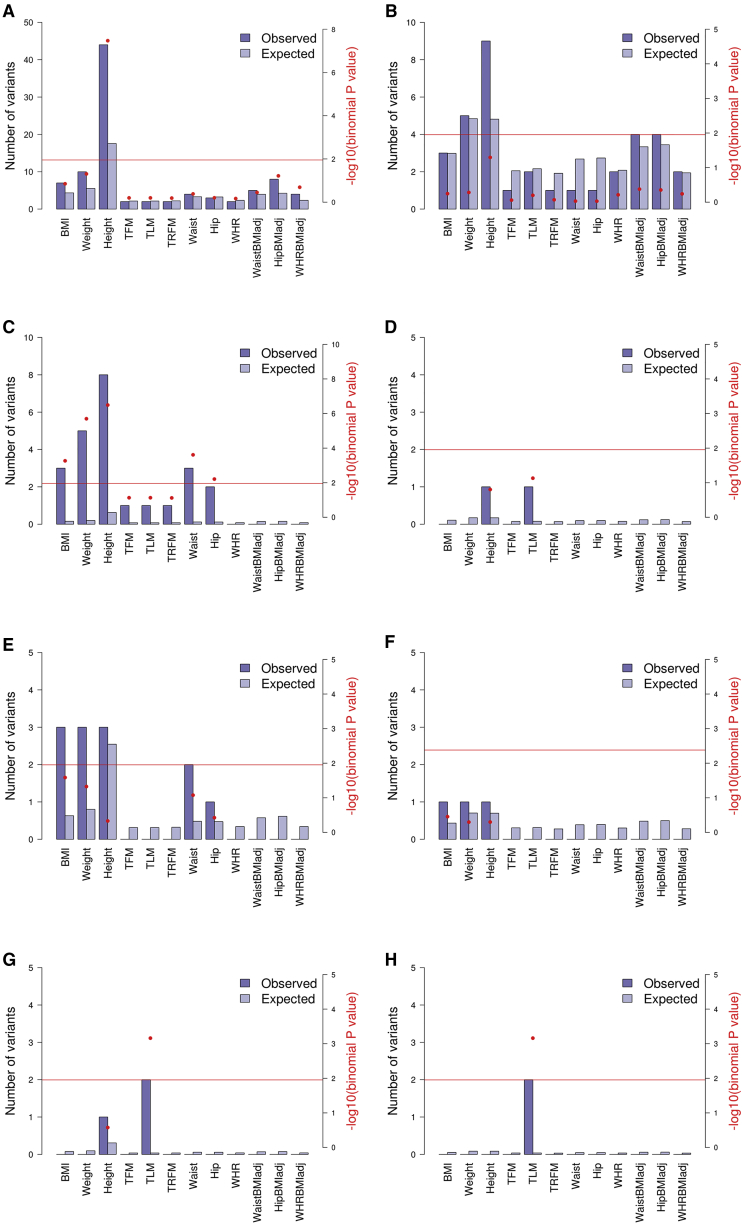
Enrichment of Discovery Meta-analysis Results in Mendelian Height-, Monogenic Obesity-, Syndromic Obesity-, and Mendelian Lipodystrophy-Associated Genes We used independent variants (r^2^ < 0.2) with MAF ≥ 0.1% (left) and after excluding previously reported loci (±500 kb) (right). Shown are Mendelian height (A and B), monogenic obesity (C and D), syndromic obesity (E and F), and Mendelian lipodystrophy (G and H). Enrichment of signal is observed if the p value (one-sided) from the binomial test of the observed versus the expected number of variants with p ≤ 10^−5^ in Mendelian-associated genes (as calculated by GREAT and denoted by the red dot) is less than 0.05/4.482 (5% significance level Bonferroni corrected for the effective number of independent traits; horizontal red line). Observed and expected counts, Bonferroni corrected p values, and FDR q values are given in Table S24. Abbreviations are as follows: BMI, body mass index; WHR, waist to hip ratio; WaistBMIadj, waist circumference adjusted for BMI; HipBMIadj, hip circumference adjusted for BMI; WHRBMIadj, waist to hip ratio adjusted for BMI; TFM, total fat mass; TLM, total lean mass; TRFM, trunk fat mass.

We then used GREAT^40^ to test whether variants with p value ≤ 10^−5^ are more likely to overlap with these sets of pre-defined genomic regions than we would expect by chance. We defined the “regulatory domain” of all protein-coding genes annotated in Ensembl release 74^41^ using the GREAT “basal plus extension” strategy: each gene is assigned a basal domain 5 kb upstream and 1 kb downstream of the gene’s transcription start site. This domain is then extended in both directions to the nearest gene’s basal domain but no more than 1 Mb in either direction. We counted the number of independent variants at the relevant p value and MAF thresholds overlapping any of the regulatory domains in each set of monogenic disorder-associated genes. If a variant overlapped more than one domain, it was counted only once. To establish whether there is a greater than expected number of variants overlapping the domains, we computed the proportion of the genome covered by the regulatory domains of each gene in the set and used this as the expected proportion of overlapping variants under the null hypothesis. To compute the proportion of genome covered by the gene set, we divided the total length of the regulatory domains of all genes in the set by the total length of the genome, excluding assembly gaps taken from the UCSC database.^42^ We then tested whether the observed overlap was greater than expected using a binomial test. We performed this test on all independent variants (r^2^ < 0.2) present in the meta-analysis results and also after excluding any previously reported variants (±500 kb) (Figure 2). We also tested for enrichment within different MAF categories (0.1% ≤ MAF ≤ 1%, 1% < MAF ≤ 5%, and MAF > 5%) (Figures S19 and S20).

### mQTL and eQTL Enrichment

Previous studies have suggested links between DNA methylation, QTLs, and complex traits.^43^, ^44^ We tested the hypotheses that methylation and expression quantitative trait loci (mQTLs and eQTLs) are enriched among anthropometric GWAS signals by calculating fold enrichment of variants at various significance cutoffs in the ARIES mQTL resource which comprises *cis* and *trans* mQTLs in blood samples^15^ and the MuTHER-ALSPAC eQTL resource^16^, ^17^ containing *cis* eQTLs for LCLs, subcutaneous fat, and skin tissue. We computed enrichments for signals using all variants and also after excluding previously reported variants (and variants within 500 kb) using GARFIELD.^45^ GARFIELD performs greedy pruning of SNPs (LD r^2^ > 0.1) and then annotates them based on overlap with the mQTLs. Fold enrichment (FE) was calculated at various p value cutoffs and assessed by permutation testing, while matching for MAF, distance to nearest transcription start site (TSS), and number of LD proxies (r^2^ > 0.8). FE = (Nat/Nt)/(Na/N), where N is the total number of pruned variants, Na is the total number of annotated variants (from the pruned set), Nt is the number of variants that pass a p value threshold T, and Nat is the number of annotated variants at threshold T. We calculated fold enrichments for traits only when there were ten or more annotated variants. We used 0.05/30 (2 GWAS annotations^∗^five time points^∗^3 mQTL annotations) as threshold to determine enrichment significance for mQTLs and 0.05/6 (3 tissues^∗^2 annotations) for eQTLs.

### eQTL Analysis

eQTL analysis was performed in the subset of UK10K individuals with microarray expression profiles available from the TwinsUK MuTHER study^16^ and ALSPAC expression study.^17^ Analysis was performed with the program PANAMA, which is based on a probabilistic model that accounts for confounding factors within an eQTL analysis.^46^ Each probe was tested for association with all variants within 250 kb of the gene inclusive of the gene body and MAF ≥ 1%. Each anthropometric trait-associated variant was evaluated for *cis*-eQTL effects by identifying associated *cis*-probes and performing mutual conditional analysis with the lead *cis*-eQTL for the corresponding probe (Table S9). We consider a GWAS and eQTL signal coincident (tagging the same underlying variant) if the eQTL p value of both the lead GWAS variant and lead eQTL variant is >0.01 when conditioned on the opposite SNP. In the UK10K expression dataset, ∼40% of genes with an eQTL have a secondary independent *cis*-eQTL. We consider the GWAS variant an independent secondary eQTL if the p value of the association between the GWAS variant and expression when conditioned on the lead eQTL variant still passes the FDR 1% threshold defined for that probe. FDR thresholds were defined via permutation at each locus.

### mQTL Analysis

mQTL analysis was performed in The Accessible Resource for Integrative Epigenomic Studies (ARIES). Of the 106 anthropometric trait-associated SNPs, 97 SNPs were genotyped or successfully imputed and passed QC (MAF > 0.001 and imputation quality score > 0.4) in ARIES. Association analysis of SNPs with CpG sites was performed using an additive model (rank-normalized CpG methylation on SNP allele count) where age (excluding birth), sex (children only), the top ten ancestry principal components, bisulfite conversion batch, and estimated white blood cell counts (using an algorithm based on differential methylation between cell types)^47^ were fitted as covariates. We removed probes that had a SNP at the CpG with a MAF > 0.01 in Europeans from the 1000G project and probes that mapped to multiple locations.^48^ We inspected the distribution of CpGs for possible effects of a SNP at the CpG or a SNP in the probe sequence. For significant CpGs, the lead mQTL SNP (p < 10^−7^) within 1 Mb of the GWAS SNP was fitted as covariate to examine whether the GWAS SNP CpG association coincided with the mQTL association (Table S10). We defined a mQTL as significant if the conditional p value > 10^−7^.

## Results

### Association Signals

In the discovery stage across 57,129 individuals, we observe an excess of suggestive association signals at p ≤ 10^−5^ (Figures S2–S14, S17, and S18, Tables S4 and S11). We followed up these in 210,823 individuals (stage 2) of European descent (Figure S1, Tables S1 and S2). In addition to genome-wide significant association at 187 established signals (Tables S4, S12, and S13, Figure S21), we report 106 genome-wide significant associations with no previous association evidence, the majority of which are associated with human height and all of which individually have small effects (each explaining < 1% trait variance) (Tables 1, 2, and S3).

Six signals reside in genomic regions that have not been implicated with related traits before (there are no established positive controls for any of the 12 anthropometric traits within 500 kb either side of the index variant; Table 1, Figure S22), and 100 signals represent conditionally independent associated variants at previously reported loci (Tables 2 and S3, Figure S23). Of these 100 signals, 28 are conditionally independent of all positive controls for any of the traits studied (Tables 2, S14, and S15). Nine associations are at low-frequency variants. These are not captured by the HapMap reference panel. 75 of the index variants reside within genes, 9 are coding, and 6 are missense (Table S16). Of the 6 variants implicating novel regions (Table 1), 2 are indels, while of 28 SNPs that are independent from positive controls (Table 2), 1 is an indel. There are 10 indels among the 72 variants in Table S3.

### Sex-Specific Analysis

We also performed sex-specific single-point analyses to investigate the presence of anthropometric trait signals in males or females that are not present in the sex-combined analysis. Using the same phenotype preparation protocol, single-point and meta-analysis strategies, and LD clumping as in sex-combined analysis, we found eight signals in males and nine signals in females (Table S17) that reached GWAS significance (p ≤ 5 × 10^−8^) and are not previously reported or identified in our sex-combined analysis. For each of these variants and for the phenotypes they were selected for, we computed p values testing for difference between the meta-analyzed men-specific and women-specific beta-estimates using a t-statistic^49^ and the Spearman rank correlation coefficient across all SNPs for each phenotype. We observe differences between sexes for these variants at a 5% FDR (Table S17).

### Rare Variant Tests

As part of the UK10K effort,^7^ burden tests (SKAT^50^ and SKAT-O^51^) were run separately for the ALSPAC and TwinsUK WGS datasets, and their summary statistics were combined using metaSKAT and metaSKAT-O^52^ (Figure S24). The list of regions with metaSKAT or metaSKAT-O p value ≤ 10^−5^ for the anthropometric traits can be found in Tables S3 and S10 of Walter et al.^7^ There are seven regions (five non-overlapping) associated with height, weight, total fat mass, or total lean mass with p ≤ 10^−7^ across either metaSKAT or metaSKAT-O results (Table S18), but no region reached stringent genome-wide significance. All region associations appeared to be led by a single variant, whose signal was weakened with the inclusion of imputed cohorts (with good imputation quality scores). Overall, rare variant association tests appeared underpowered to detect strong associations using our combined WGS sample size (3,049–3,559) for anthropometric traits.

### Sample Overlap across UK-Based Cohorts

The meta-analysis method used here assumes that individual cohorts are independent from each other, i.e., samples are not shared or related. Using raw genotypes genome-wide, we calculated IBD estimates for the UK-based studies, namely UK Biobank (application numbers 10205 and 7439), UKHLS (EGAD00010000918), TwinsUK WGS and GWAS data, arcOGEN (EGAS00001001017), and 1958 Birth Cohort (we did not include ALSPAC WGS or GWAS data, as it consists of children only). The number of overlapping pairs of samples (pi-hat > 0.98) between each dataset and UK Biobank as well as related pairs (pi-hat > 0.2) is given in Table S19. To investigate the effect of sample overlap and relatedness across cohorts, we focused on height and meta-analyzed the discovery cohorts with UK Biobank using METACARPA, a meta-analysis method that corrects for sample overlap and relatedness across studies, as well as METAL (which does not correct for overlap) for a direct comparison. METACARPA was run in two stages. In the first stage, we used genome-wide results from all cohorts to estimate correlation across studies, and in the second stage we meta-analyzed betas across cohorts corrected for relatedness for the variants associated with height (Table S20). As expected, p values uncorrected for relatedness are inflated compared to the corrected p values but the difference is not significant (Figure S25). The correlation between the uncorrected and corrected effect sizes is almost 1 (Figure S25), and therefore the presence of any relatedness in our data has a minimal effect on the effect sizes.

### Genetic Correlation

We observe genetic correlation in 43 pairs of anthropometric traits out of 66 possible pairs at 5% FDR (Figure 1, Table S21). For example, we observe high genetic correlation of BMI with weight (0.81, p < 10^−320^), DXA traits (0.64–0.86, p 7.14 × 10^−25^–1.34 × 10^−42^), waist circumference (0.89, p < 10^−320^), hip circumference (0.83, p = 8.70 × 10^−119^), and waist to hip ratio (0.43, p = 2.98 × 10^−6^). In contrast, genetic correlation was not significant between BMI and traits adjusted for BMI, such as height, waist circumference, hip circumference, and waist to hip ratio adjusted for BMI. Overall, we observe that when trait A is positively correlated with traits B and C, the correlation between trait A and trait B adjusted for trait C drops significantly, for example hip versus waist circumference and hip versus waist circumference adjusted for BMI.

We also observe high genetic correlation of height with weight (0.53, p = 5.77 × 10^−55^), hip (0.37, p = 2.30 × 10^−13^) and waist circumference (0.28, p = 1.62 × 10^−9^), as well as total fat mass (−0.25, p = 5.21 × 10^−4^) and trunk fat mass (−0.23, p = 3.05 × 10^−3^) at 5% FDR. When adjusting hip and waist circumference for BMI, their statistical correlation with height becomes more significant (0.84, p = 1.32 × 10^−67^ and 0.73, p = 1.11 × 10^−51^, respectively), which implies that height could play a mediating role in the genetic associations of these traits through its inverse relationship to BMI. More generally, when trait A is positively correlated with trait B and negatively correlated with trait C, the correlation between trait A and trait B adjusted for trait C (or trait D positively correlated with trait C) increases significantly. These findings are compatible with previous work^53^ suggesting that unintended bias, known as collider bias, can be introduced when a trait is adjusted for another trait.

Total fat mass is highly correlated with trunk fat mass (0.95, p = 3.11 × 10^−79^), but total lean mass is not correlated to either of these traits. DXA traits are highly correlated with BMI, weight, waist circumference, and hip circumference. Compatible with the observations above, the strongest correlations of DXA traits are with BMI, implying a mediator role of height. Also, as expected, the correlation between DXA traits and waist and hip circumference disappears when the latter traits are adjusted for BMI.

The pleiotropy among anthropometric traits is recapitulated by examining the overlap of all 106 signals (Tables 1, 2, and S3) robustly associated with an anthropometric trait at p ≤ 5 × 10^−8^ in stage1+stage2 (Table S15) with each of the other anthropometric traits studied. As expected, we observe significant overlap of variants associated with both weight and height (49, Figure S26A), while 11/13 variants associated with BMI are also associated with weight (Figure S26A) and both total fat mass signals are also trunk fat mass and BMI signals (Figure S26B). Furthermore, 8/13 BMI signals are associated with waist and hip circumference (Figure S26C), but this overlap disappears once waist and hip circumference analyses are adjusted for BMI (Figure S26E). 25/35 hip circumference signals are also height signals (Figure S26D). Again, we confirm systematic relationships between waist and hip circumference signals adjusted for BMI with height variants, as 22/23 and 52/53 of those, respectively, are also height signals (Figure S26F).

### Collider Bias

Collider bias can be introduced when a trait is adjusted for another trait,^53^ for example when adjusting waist to hip ratio for BMI or DXA traits for height. To investigate whether false phenotype-genotype associations are induced when the phenotype of interest is adjusted for another phenotype, we initially looked at the effect sizes in our discovery meta-analysis for waist circumference adjusted for BMI and BMI. Out of 146 independent (pairwise r^2^ < 0.2 and further than 500 kb) variants associated with waist circumference adjusted for BMI in the discovery meta-analysis with p < 10^−5^, 77 (52.74%) had opposite direction of effects for BMI and waist circumference adjusted for BMI, and therefore there was no evidence of enrichment for SNPs harboring opposite marginal effects on the two traits (binomial p = 0.28). The expected proportion of SNPs having effect in opposite direction in a model where the genetic variant is associated with the outcome but not the covariate is smaller or equal to 50%,^53^ which is what we observed in our results, indicating absence of collider bias. We observed similar results for the effect of BMI on hip circumference and waist to hip ratio adjusted for BMI, as well as height on DXA traits (Table S21, Figure S27). Moreover, variants that reached genome-wide significance for waist or hip circumference and for waist to hip ratio adjusted for BMI are not significantly associated with BMI (their discovery meta-analysis p values are between 0.85 and 0.01, while their overall p value ranged between 0.96 and 2.64 × 10^−4^, Table S15). The two variants associated with total and trunk fat mass reached genome-wide significance for height but also for BMI (Table S15), which suggests true association with adiposity rather than mediation through height. We concluded that there is no evidence that our results suffer from collider bias.

### Fine-Mapping

**Figure 3:**
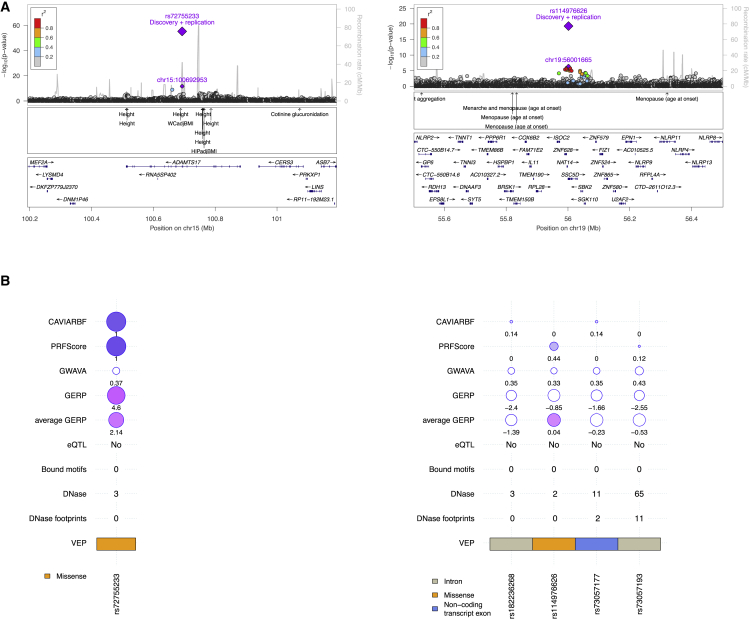
Combined Information from Fine-Mapping Methods, Functional Prediction Scores, and eQTL Analysis to Assess the Overall Evidence Supporting Functional and Causal Interpretation at Fine-Mapped Regions of Newly Identified Variants Example of fine-mapping and annotation at the *ADAMTS17* (left) and *SSC5D* (right) loci for association with height. LocusZoom regional association plot shown in (A) and posterior probability (PP) statistics shown in (B) are from the fine-mapping methods CAVIARBF and PRFScore (only variants with PP > 0.1 in either methods are shown); genome-wide annotation of variants (GWAVA) scores; genomic evolutionary rate profiling (GERP) scores; average GERP (in a 100 bp window around each variant) scores; whether the variant is an eQTL signal; number of cell lines in which the variant overlaps with a DNase footprints (peak calls from ENCODE); number of overlapping transcriptional factor binding sites based on ENCODE and JASPAR ChIP-seq; number of cell lines in which the queried locus overlaps with a DNase hypersensitivity site (ENCODE data, peaks from Ensembl); and Variant Effect Predictor (VEP) genic annotation. Circle sizes and colors for all scores are scaled with respect to score type and numbers are plotted below each circle. Probabilities of causality from CAVIARBF and PRFScore are colored in shades of purple. GWAVA scores range between [0,1] and scores greater than 0.5 indicate functionality (colored in white for scores < 0.5 and in shades of orchid for scores > 0.5). GERP scores range between [−12.3,6.17] with scores above zero indicating constraint (colored in white for scores < 0 and in shades of orchid for scores > 0).

To examine the fine-mapping potential of deep WGS imputation, we undertook fine mapping^28^ of the 106 associations reported here. By combining variants predicted to be causal with posterior probability of association over 0.1 by either CAVIARBF or PRFScore, we find that out of 30 regions that successfully produced 95% credible intervals, 14 credible sets narrowed down to a single variant, 12 narrowed down to 2 or 3 variants, and 3 sets were reduced down to 4 variants (Tables S5 and S22). To assess the overall evidence supporting functional and causal interpretation at the 30 fine-mapped regions, we combined information from the two fine-mapping methods, two functional prediction scores (Genome Wide Annotation of Variants^54^ [GWAVA] and GERP scores), and eQTL analysis (Figures 3 and S28). Of the 30 regions, 6 were fine-mapped to a coding variant (5 missense and 1 synonymous) and 9 were fine-mapped to a variant that was identified as an eQTL.

Two missense variants predicted to be causal are associated with height and reside in genes of the *ADAMTS* family of extracellular matrix proteases, which have been previously associated with height.^39^, ^55^, ^56^ rs72755233 (weighted effect allele frequency [WEAF] 11.2%, beta = −0.0837, p = 5.42 × 10^−56^) resides in *ADAMTS17* and causes a non-conservative threonine to isoleucine amino acid change in the protease domain of this peptidase. Similarly, rs62621197 (WEAF 4.2%, beta = −0.139, p = 3.22 × 10^−69^) resides in *ADAMTS10*, null mutations in which are implicated in Weill-Marchesani syndrome, characterized by short stature.^57^ Previously reported, independent variants associated with height at this locus reside upstream of *ADAMTS10* (rs4072910^6^) and in intronic sequence (rs7249094^55^) (Table S14). rs62621197, identified here, results in an amino acid substitution (p.Arg62Gln) directly adjacent to the furin cleavage site, where the presence of glutamine may decrease ADAMTS10 activation efficiency.^58^

We also undertook fine mapping^28^ of 186 anthropometric trait loci established in the literature which also reached p ≤ 5 × 10^−8^ in the discovery stage (Table S4). We find that 14 credible sets 95% likely to contain the causal variant are narrowed down to a single variant, and 6 are narrowed down to 2 causal variants (Table S23).

For example, fine-mapping of the region around the previously established variant rs28929474 resulted in a credible set of two missense variants associated with height. rs28929474 (WEAF 2.1%, beta = 0.138, height p = 5.35 × 10^−41^) in *SERPINA1* encodes a missense change (p.Glu366Lys) in the serine protease inhibitor domain of alpha-1-antitrypsin (AAT). Homozygosity results in AAT deficiency, associated with increased risk of early-onset chronic obstructive pulmonary disease.^59^ rs28929474 heterozygosity has been associated with increased pulmonary function and height.^60^ AAT inhibits cleavage of the reactive center loop of corticosteroid binding globulin (CBG) (coded by *SERPINA6*, located next to *SERPINA1*), preventing the release of cortisol. Variation in this locus has been associated with plasma cortisol levels^61^ and there is epidemiological evidence that cortisol and height are inversely correlated.^62^

### Enrichment of Association Signal in Monogenic and Syndromic Disorder-Associated Genes

Consistent with previous work,^4^, ^6^, ^63^ we find enrichment of height-associated signals in genes mutated in human syndromes characterized by abnormal skeletal growth (2.51-fold enrichment; p = 3.38 × 10^−8^), of BMI-related signals in genes implicated in monogenic obesity (19.32-fold enrichment for BMI; p = 5.43 × 10^−4^) and of total lean mass-related associations in Mendelian lipodystrophy-associated genes (52.86-fold enrichment for BMI; p = 6.90 × 10^−4^) (Figure 2, Table S24). Enrichment remains after the removal of established lipodystrophy loci and is attenuated when previously identified height and BMI common-frequency variant signals are removed (Figures 2, S19, and S20, Table S24).

We also observe enrichment of BMI-, weight-, waist-, and height-related signals in monogenic obesity-related genes (Figures 2 and S20), which can be explained by the fact that these phenotypes are highly correlated (Figure 1). The absence of enrichment of hip circumference, waist to hip ratio, and DXA-related signals (despite their significant correlation to BMI, estimated using genome-wide estimates independent of p value thresholds) is likely due to low power to detect enough signals with p < 10^−5^ (their sample sizes in our discovery phase are approximately 37K and 15K).

### Proximity to OMIM Genes

We examined whether any genes with an associated OMIM morbidity identifier were located within 1 Mb of the identified variants, and we found 268 such genes across 103 out of the 106 signals (Table S25). Among these genes many were implicated in bone development and musculoskeletal phenotypes. One gene (*ADAMTS10*) was overlapping with an identified signal for height (index variant rs62621197) and it is involved in Weill-Marchesani syndrome (MIM: 277600), a connective tissue disorder characterized by short stature.^57^ Other genes and their implicated roles are summarized in Table S25. Pathogenic mutations associated with these OMIM genes were not in LD with our reported signal (r^2^ is 0) and were not present in the UK10K WGS dataset.

### Musculoskeletal Phenotypes

Consistent with previous work,^5^, ^6^ we observe a strong theme of musculoskeletal implications (79 of 106 variants). A variant was considered to have musculoskeletal implications if (1) it is located within 100 kb or if it is an eQTL for a gene that has a relevant OMIM annotation, including association with human syndromes and animal models of relevant gene knock-outs,^64^, ^65^, ^66^, ^67^, ^68^, ^69^, ^70^, ^71^, ^72^, ^73^, ^74^, ^75^, ^76^, ^77^, ^78^, ^79^, ^80^, ^81^, ^82^, ^83^ such as abnormal skeletal, muscle, or cartilage development and abnormal body size or bone morphology, and (2) there are any skeletal-related GWAS signals within 100 kb, such as bone mineral density. For example, rs35863206 (WEAF 22.35%, beta = −0.0232, height p = 5.91 × 10^−9^) is a deletion located 53 kb upstream of *PGR*, which encodes the progesterone receptor protein and is correlated with rs147581469 (r^2^ = 0.72), a previously identified eQTL for *PGR*.^84^ *Pgr* mouse knock-out models exhibit severe abnormal ossification and skeletal irregularities.^67^

### eQTL Analysis Results

We find *cis* eQTL enrichment (p < 0.008, Table S26) for BMI, height, weight, waist circumference, and waist to hip ratio adjusted for BMI signals in subcutaneous fat and for BMI, height, weight, and waist circumference in lymphoblastoid cell lines (Table S26). BMI and height show the strongest enrichments at multiple GWAS thresholds. No significant eQTL enrichments are found for waist to hip ratio, hip circumference, hip circumference adjusted for BMI, total fat mass, total lean mass, or trunk fat mass. Overall, no enrichments are found for skin eQTLs. After excluding regions of previously identified loci, the enrichment remains significant for height and waist circumference adjusted for BMI in subcutaneous fat and for all traits in LCLs. Subcutaneous fat eQTLs is enriched among height and waist circumference adjusted for BMI GWAS signals. GWAS signals show enrichments at GWAS thresholds of 10^−5^ and 10^−6^. Given that the LCL sample size is twice as that of the other two tissues (n = 823 in LCLs, n = 391 adipose tissue, n = 367 skin tissue) and that the expression data of a transformed cell line is less prone to environmental effects, the number of eQTLs for LCLs is larger than for fat and skin, which may explain the larger number of LCL eQTLs enrichments among anthropometric traits.

To integrate the identified variants with the eQTL data, reciprocal conditional analyses were performed in the expression data with the lead GWAS variant and peak eSNP to identify coincident signals. Several of the GWAS variants coincided with the lead eQTL for neighboring genes, including rs3888183 for *MCMBP* in all three tissues, rs4360494 for *FHL3* in adipose and LCLs, rs6901225 for *ABT1* in LCLs and rs577721086 for *RSPO3* in adipose (Table S9). Additional GWAS variants were associated with gene expression after conditioning on the lead eQTL, indicating that they are tagging independent secondary eQTLs. We note that as some variants have low MAF, the relatively modest size of the UK10K expression dataset is underpowered to detect eQTLs and larger expression studies may reveal further regulatory effects associated to these variants.

### mQTL Analysis Results

We find signal enrichment for mQTL (p < 0.002, Table S27, Figure S29) in blood samples at three time points in the life course of ALSPAC participants and two time points in the life course of their mothers^15^ at different p value thresholds, mostly driven by *cis* mQTLs for BMI, height, waist circumference, weight, total fat mass, and trunk fat mass. After excluding previously reported variants (and all variants within 500 kb), BMI, height, waist circumference, weight, total fat mass, and trunk fat mass variants remained significantly enriched for mQTLs for several time points. However, the total fat mass and trunk fat mass enrichments disappeared after removing previous published BMI and obesity GWAS signals.

Height and weight show enrichment of *trans* mQTLs during pregnancy and birth, whereas BMI was not enriched for *trans* mQTLs using the same sample size in the GWAS analysis. Enrichment of *trans* mQTLs is consistent with the possibility that the relative influence of the environment on methylation levels increases over time. Also, given that *trans* mQTL signals may be polygenic themselves, enrichment of *trans* mQTLs may be explained by the polygenic architecture of traits such as height. Overall, stronger enrichments were found for *cis* mQTLs than *trans* mQTLs and a lower GWAS threshold resulted in stronger enrichments. Comparing different GWAS thresholds confirms that among associations that do not surpass the genome-wide significance p value threshold, functional information can enhance discovery of true associations. These findings confirm that trait-associated SNPs will often affect the trait by gene regulation. Using large sample sizes leads to higher power to detect enrichment for complex polygenic traits, such as the anthropometric traits studied here.

Of the 97 reported variants tested in ARIES, 76 variants showed evidence for mQTL (664 unique SNP-CpG pairs across all time-points, p < 10^−7^) of which 550 associations were in *cis* and 114 in *trans* (Table S10).

## Discussion

We have conducted a sequence-based association scan for anthropometric traits empowered by deep imputation (Figures S30 and S31). A key message derived from our findings is that large-scale, well-imputed association scans continue to discover complex trait loci. As an exemplification of the point, we identify associations at low-frequency variants, not captured by previous reference panels, including a large number of associations at common-frequency variants, which were missed by previous studies.^4^, ^5^, ^6^, ^85^ These are signals for traits not studied extensively before (n = 40/97 in Table S3) but are genetically correlated to other well-studied anthropometric traits, not tagged by previous imputation approaches (n = 7/28 in Table 2, n = 16/97 in Table S3), or reaching sub-threshold significance levels in previous studies (n = 21/28 in Table 2, n = 41/97 in Table S3). Therefore, further increasing sample size and sequencing depth and building large reference panels to facilitate accurate imputation is likely to identify further potentially functional variants underpinning the genetic architecture of medically relevant human complex traits. Transethnic fine-mapping of deeply imputed datasets can then deliver further resolution of causal genes and variants.^86^

We found moderate overlap of genes implicated by the GWAS, the two fine-mapping methods, and eQTL and mQTL analyses (Table 3). Altogether we have found 283 unique genes, 225 (79.5%) of which were found by only one method, while there were no genes identified by all methods (46 and 12 genes were found by two or three methods, respectively). Out of 99 genes identified by the GWAS, 13 were identified by fine-mapping, 8 by eQTL, and 41 by mQTL. The observed moderate overlap across analysis strands suggests that the closest protein-coding gene to a susceptibility variant is not necessarily the gene affected by the variant, or that indeed the variant does not affect gene methylation or expression. Out of these 13 genes that were identified by both GWAS and fine mapping, 12 (*CDK6*, *IGF2BP2*, *HSD17B12*, *ID4*, *ZBTB38*, *ADAMTS10*, *RSPO3*, *MAPK3*, *DLEU1*, *ADAMTS17*, *GDF5*, and *PDXDC1*) have been previously associated with anthropometric GWAS signals.

**Table 3:** Pairwise Overlap of Genes Implicated by the GWAS, Two Fine-Mapping Methods, eQTL and mQTL Analyses

	**GWAS**	**Fine-Mapping**	**eQTL**	**mQTL**	**Total Genes**	**Unique Genes**
GWAS	99	13	8	41	99	49 (49.5%)
Fine-mapping	13	24	2	9	24	8 (33.3%)
eQTL	8	2	19	9	19	6 (31.6%)
mQTL	41	9	9	211	211	162 (76.8%)
					283	225 (79.5%)

Closest protein-coding genes identified by the GWAS and the two fine-mapping methods CAVIARBF and PRFScore, and genes identified by the eQTL and mQTL analyses.

To get a functional overview of the genes implicated by the different methods, we classified them based on their associated gene ontology (GO) terms for biological processes. Before the analysis, GO gene sets were filtered to keep the most reliable associations, namely only those genes were kept in a biological process group, where the supporting evidence was: physical interaction, mutant phenotype, direct assay, expression pattern, or traceable author statement. The final set contained 9,440 genes distributed across 2,833 overlapping categories. Our 283 identified genes were assigned 377 different annotation terms (Table S28). Focusing on 52 annotation terms that contained three or more genes, the most pronounced categories were related to gene regulation, immune system, signal transduction, and cell proliferation. Other highlighted processes were related to metabolism and development terms, as well as skeletal system development represented by five genes (*SOX9*, *BMP2*, *IGFBP4*, *NKX3-2*, and *FBN1*) (Table S28).

The gene sets associated with methylation and expression QTLs yielded 64 different gene ontology annotations with at least two or more genes (Table S29). The most abundant categories were related to immune system, cell proliferation, and gene expression, and there were also ontology terms with clear musculoskeletal consequences, such as skeletal system development, chondrocyte differentiation, and regulation of ossification. These annotations were represented by genes previously identified from genome-wide association studies of anthropometric traits, such as *CDK6*, *GDF5*, *HMGA2*, *IGFBP4*, *FBN1*, and *WNT5A*, which suggests that eQTL and mQTL analyses can contribute to our understanding of the biology underlying complex traits but were also represented by three genes (*PDK1*, *NKX3-2*, *VPS29*) with no previously reported GWAS associations. Looking closely into these genes, we found animal models and other biological information supporting their relevance to anthropometric traits.

Specifically, *PDK1* is the closest protein-coding gene to rs28610092, associated with waist circumference adjusted for BMI in our study, was implicated by fine-mapping, and is a mQTL. Animal models of *PDK1* show abnormal adipose tissue development^87^ and a series of skeletal and ossification abnormalities including abnormal radius^88^ and femur^87^ morphology, as well as abnormal osteoblast differentiation.^87^ *NKX3-2* is a homeobox gene and the closest protein coding gene to rs16888802, associated with height in our study, and identified by the GWAS and mQTL analyses. Although *NKX3-2* has no previous anthropometric associations, it is associated with spondylo-megaepiphyseal-metaphyseal dysplasia, an autosomal-recessive disorder characterized by diverse skeletal abnormalities,^72^ including disproportionate short stature with a short and stiff neck and trunk.^72^ These phenotypic abnormalities were recapitulated in mouse models.^89^, ^90^, ^91^ Finally, *VPS29* was associated to the weight signal rs112540634 by mQTL analysis. The protein product of *VPS29* is part of the retromer complex of the Wnt signaling pathway,^92^, ^93^ which is involved in adipogenesis and adipocyte development.^94^, ^95^

The pronounced representation of immune-related annotations in the gene sets identified by eQTL and mQTL might be explained by the blood-related sources of the studied tissues (mQTL data come explicitly from blood; LCLs, subcutaneous fat, and skin tissues were used for the eQTL data, but the LCL sample size is twice as that of the other two tissues).

**Figure 4:**
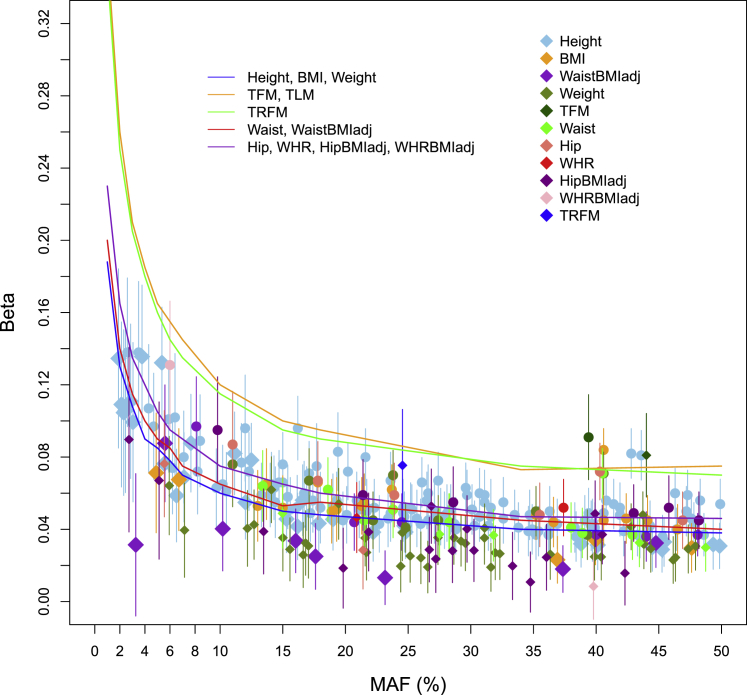
Power to Detect Association in the Discovery Stage, Stage 1 Effect sizes and 95% confidence intervals (absolute value of beta, expressed in standard deviation units) as a function of minor allele frequencies (MAF), based on stage 1 of this study. Newly reported variants are denoted in diamonds, and previously reported variants that reach genome-wide significance (p ≤ 5 × 10^−8^, two-sided) in the discovery stage are denoted in circles. The curves indicate 80% power at the genome-wide significance threshold of p ≤ 5 × 10^−8^, for five representative sample sizes of the discovery stage: (1) height, BMI, weight; (2) TFM, TLM; (3) TRFM; (4) waist circumference, waist circumference adjusted for BMI; (5) hip circumference, waist to hip ratio, hip circumference adjusted for BMI, waist to hip ratio adjusted for BMI. The sample size for height (blue line) had 80% power to detect associations down to 0.1% MAF for betas ≥ 0.19 standard deviations (0.36 and 0.23 for TFM [orange] and waist to hip ratio [purple], respectively; not plotted). Further power calculations for different sample sizes are given in Figure S32. Abbreviations are as follows: BMI, body mass index; WHR, waist to hip ratio; WaistBMIadj, waist circumference adjusted for BMI; HipBMIadj, hip circumference adjusted for BMI; WHRBMIadj, waist to hip ratio adjusted for BMI; TFM, total fat mass; TLM, total lean mass; TRFM, trunk fat mass.

In this study, we set out to identify associations across the full allele frequency spectrum. Consistent with previous studies,^96^, ^97^, ^98^ we find substantial genetic overlap between monogenic and polygenic anthropometric traits, driven primarily by common variants with small effect sizes. Importantly, even though well powered to detect them, we find no evidence of low-frequency variants with strong effect sizes (Figure 4). For example, for height and waist to hip ratio, this study had 80% power to detect associations down to 0.1% MAF for betas ≥ 0.19 and 0.23 standard deviations, respectively, at the genome-wide significance level. It is possible that this picture might change with larger sample sizes sequenced at higher read depths, which would allow researchers to systematically interrogate variants with MAF < 0.1% and increase association power for small effect sizes for low frequency and rare variants. Millions of variants with MAF < 0.1% were not included in this study, many due to imputation accuracy score filters. There may therefore still be true signal to discover in the 0.1%–1% MAF range—even with current sample sizes—if the imputation qualities improve. In addition, within the power constraints of the study, we do not identify any significant association with burdens of rare variants. It is likely that such burdens exist but that the rare variants contributing to them could not be detected by the low read depth of the WGS data generated here. Going forward, deep whole-genome sequencing of large-scale cohorts holds the promise of comprehensively interrogating the allelic architecture of complex traits.

## Web Resources

ALSPAC data dictionary, http://www.bris.ac.uk/alspac/researchers/data-access/data-dictionary/
arcOGEN, https://www.arcogen.org.uk/
ARIES Explorer, http://www.ariesepigenomics.org.uk/ariesexplorer
European Genome-phenome Archive (EGA), https://www.ebi.ac.uk/ega
GWAS Catalog, http://www.ebi.ac.uk/gwas/
HELIC, https://www.helic.org/
METACARPA, https://bitbucket.org/agilly/metacarpa/
OMIM, http://www.omim.org/
PANAMA, https://pypi.python.org/pypi/panama/
PIVUS, http://www.medsci.uu.se/PIVUS/
UK Biobank Protocol, http://www.ukbiobank.ac.uk/wp-content/uploads/2011/11/UK-Biobank-Protocol.pdf
Understanding Society, https://www.understandingsociety.ac.uk/

## Supplemental Data

Document S1. Consortia Information, Acknowledgments and Conflicts of Interest, Cohort Descriptions, Annotations of Identified Variants, Figures S1–S32, and Tables S1, S13, S19, and S21

Table S2. Cohort Information

Table S3. Genome-wide Significant New Associations at Variants Known for a Different Anthropometric Trait

Table S4. Independent and Significant Loci for BMI at p < 1 × 10^-5^ for Sex-Combined Analysis

Table S5. Fine Mapping Results of Newly Identified Variants Given Epigenomic Annotations

Table S6. Pairwise Genetic Correlation Estimates and Their 95% Confidence Intervals across 12 Anthropometric Traits

Table S7. Monogenic and Syndromic Obesity Genes, as Identified through OMIM

Table S8. Lipodystrophy Genes

Table S9. Significant eQTL Associations for Newly Associated SNPs

Table S10. MethQTL Associations for Newly Associated SNPs

Table S11. Enrichment in Discovery Meta-analysis using Independent Variants (r^2^ < 0.2) with MAF ≥ 0.1% (Figure S17A)

Table S12. Previously Identified Variants for Anthropometric Traits with p < 1 × 10-5 in Our Sex-Combined Analysis

Table S14. Reported Associations of Our Top SNPs with Anthropometric Traits within 1 MB

Table S15. Association of Our Newly Identified Genome-wide Loci with Other Anthropometric Traits in Stage1+Stage2

Table S16. Annotations of the Newly Identified Variants

Table S17. Significant Loci in Phase I at p ≤ 5 × 10^-8^ for Sex-Specific Analyses

Table S18. Summary of Results of Genome-wide Rare Variants Tests

Table S20. Meta-analysis Results of Newly Identified Variants Associated with Height using METACARPA and METAL

Table S22. Fine Mapping Results of Newly Identified Variants

Table S23. Fine Mapping Results of Known Variants

Table S24. Enrichment of Discovery Meta-analysis Results in Height Mendelian Genes using Independent Variants (r^2^ < 0.2) with MAF ≥ 0.1% (Figure 2A)

Table S25. Genes with Associated OMIM Morbidity Identifier Located within 1 Mbp of the Newly Identified Variations

Table S26. EQTL Enrichments (p < 0.002) among Anthropometric Traits

Table S27. MethQTL Enrichments (p < 0.002) among Anthropometric Traits

Table S28. Gene Ontology Annotations across 12 Anthropometric Traits and the GWAS, Fine-Mapping, eQTL, and mQTL Results

Table S29. Gene Ontology Annotations Found by the eQTL and mQTL Results

Document S2. Article plus Supplemental Data
