# Supplementary material for: Whole-Genome Sequencing Coupled to Imputation Discovers Genetic Signals for Anthropometric Traits
Source: Am J Hum Genet. 2017 May 25;100(6):865–84. doi: 10.1016/j.ajhg.2017.04.014 (PMC5473732; doi:10.1016/j.ajhg.2017.04.014)
Supplement: Document S1. Consortia Information, Acknowledgments and Conflicts of Interest, Cohort Descriptions, Annotations of Identified Variants, Figures S1–S32, and Tables S1, S13, S19, and S21 [file mmc1.pdf]

The American Journal of Human Genetics, Volume *100*

Supplemental Data

Whole-Genome Sequencing Coupled to Imputation Discovers Genetic Signals for Anthropometric Traits

Ioanna Tachmazidou, Dániel Süveges, Josine L. Min, Graham R.S. Ritchie, Julia Steinberg, Klaudia Walter, Valentina Iotchkova, Jeremy Schwartzentruber, Jie Huang, Yasin Memari, Shane McCarthy, Andrew A. Crawford, Cristina Bombieri, Massimiliano Cocca, Aliki-Eleni Farmaki, Tom R. Gaunt, Pekka Jousilahti, Marjolein N. Kooijman, Benjamin Lehne, Giovanni Malerba, Satu Männistö, Angela Matchan, Carolina Medina-Gomez, Sarah J. Metrustry, Abhishek Nag, Ioanna Ntalla, Lavinia Paternoster, Nigel W. Rayner, Cinzia Sala, William R. Scott, Hashem A. Shihab, Lorraine Southam, Beate St Pourcain, Michela Traglia, Katerina Trajanoska, Gialuigi Zaza, Weihua Zhang, María S. Artigas, Narinder Bansal, Marianne Benn, Zhongsheng Chen, Petr Danecek, Wei-Yu Lin, Adam Locke, Jian'an Luan, Alisa K. Manning, Antonella Mulas, Carlo Sidore, Anne Tybjaerg-Hansen, Anette Varbo, Magdalena Zoledziewska, Chris Finan, Konstantinos Hatzikotoulas, Audrey E. Hendricks, John P. Kemp, Alireza Moayyeri, Kalliope Panoutsopoulou, Michal Szpak, Scott G. Wilson, Michael Boehnke, Francesco Cucca, Emanuele Di Angelantonio, Claudia Langenberg, Cecilia Lindgren, Mark I. McCarthy, Andrew P. Morris, Børge G. Nordestgaard, Robert A. Scott, Martin D. Tobin, Nicholas J. Wareham, SpiroMeta Consortium, GoT2D Consortium, Paul Burton, John C. Chambers, George Davey Smith, George Dedoussis, Janine F. Felix, Oscar H. Franco, Giovanni Gambaro, Paolo Gasparini, Christopher J. Hammond, Albert Hofman, Vincent W.V. Jaddoe, Marcus Kleber, Jaspal S. Kooner, Markus Perola, Caroline Relton, Susan M. Ring, Fernando Rivadeneira, Veikko Salomaa, Timothy D. Spector, Oliver Stegle, Daniela Toniolo, André G. Uitterlinden, arcOGEN Consortium, Understanding Society Scientific Group, UK10K Consortium, Inês Barroso, Celia M.T. Greenwood, John R.B. Perry, Brian R. Walker, Adam S. Butterworth, Yali Xue, Richard Durbin, Kerrin S. Small, Nicole Soranzo, Nicholas J. Timpson, and Eleftheria Zeggini

EXPANDED AUTHOR INFORMATION

UK10K Project Consortium

Saeed Al Turki [1,2], Carl A. Anderson [1], Richard Anney [3], Dinu Antony [4], María Soler Artigas [5], Muhammad Ayub [6], Senduran Bala [1], Jeffrey C. Barrett [1], Inês Barroso [1,7], Phil Beales [4], Jamie Bentham [8], Shoumo Bhattacharya [8], Ewan Birney [9], Douglas Blackwood [10], Martin Bobrow [11], Elena Bochukova [7], Patrick F. Bolton [12,13,14], Rebecca Bounds [7], Chris Boustred [15], Gerome Breen[13,14], Mattia Calissano [16], Keren Carss [1], Ruth Charlton [17], Krishna Chatterjee [7], Lu Chen [1,18], Antonio Ciampi [19], Sebahattin Cirak [16,20], Peter Clapham [1], Gail Clement [21], Guy Coates [1], Massimiliano Cocca [22,23], David A. Collier [14,24], Catherine Cosgrove [8], Tony Cox [1], Nick Craddock [25], Lucy Crooks [1,26], Sarah Curran [12,27,28], David Curtis [29], Allan Daly [1], Petr Danecek [1], Ian N. M. Day [30], Aaron Day-Williams [1,31], Anna Dominiczak [32], Thomas Down [1,33], Yuanping Du [34], Ian Dunham [9], Richard Durbin [1], Sarah Edkins [1], Rosemary Ekong [35], Peter Ellis [1], David M. Evans [36,37], I. Sadaf Farooqi [7], David R. Fitzpatrick [38], Paul Flicek [1,9], James Floyd [1,39], A. Reghan Foley [16], Christopher S. Franklin [1], Marta Futema [40], Louise Gallagher [3], Tom R. Gaunt [36], Matthias Geihs [1], Daniel Geschwind [41], Celia M. T. Greenwood [19,42,43,44], Heather Griffin [45], Detelina Grozeva [11], Xiaosen Guo [34,46], Xueqin Guo [34], Hugh Gurling [47], Deborah Hart [21], Audrey E. Hendricks [1,48], Peter Holmans [25], Bryan Howie [49], Jie Huang [1], Liren Huang [34], Tim Hubbard [1,33], Steve E. Humphries [40], Matthew E. Hurles [1], Pirro Hysi [21], Valentina Iotchkova [1,9], David K. Jackson 1, Yalda Jamshidi [50], Chris Joyce [1], Konrad J. Karczewski [51,52], Jane Kaye [45], Thomas Keane [1], John P. Kemp [36,37], Karen Kennedy [1,53], Alastair Kent [54], Julia Keogh [7], Farrah Khawaja [55], Margriet van Kogelenberg [1], Anja Kolb-Kokocinski [1], Genevieve Lachance [21], Cordelia Langford [1], Daniel Lawson [56], Irene Lee [57], Monkol Lek [51], Rui Li [42,43,58], Yingrui Li [34], Jieqin Liang [34], Hong Lin [34], Ryan Liu [59], Jouko Lönnqvist[60], Luis R. Lopes [61,62], Margarida Lopes [1,63,64], Daniel G. MacArthur [51,52], Massimo Mangino [21,65], Jonathan Marchini [63,66], Gaëlle Marenne [1], John Maslen [1], Iain Mathieson [67], Shane McCarthy [1], Peter McGuffin [14], Andrew M. McIntosh [10], Andrew G. McKechanie [10,68], Andrew McQuillin [47], Yasin Memari [1], Sarah Metrustry [21], Nicola Migone [69], Josine L. Min [36], Hannah M. Mitchison [4], Alireza Moayyeri [21,70], Andrew Morris [71], James Morris [1], Dawn Muddyman [1], Francesco Muntoni[16], Kate Northstone [36], Michael C. O'Donovan [25], Stephen O'Rahilly [7], Alexandros Onoufriadis [33], Karim Oualkacha [72], Michael J. Owen [25], Aarno Palotie [1,73,74], Kalliope Panoutsopoulou [1], Victoria Parker [7], Jeremy R. Parr [75], Lavinia Paternoster [36], Tiina Paunio [60,76], Felicity Payne [1], Stewart J. Payne [77], John R. B. Perry [21,78], Olli Pietilainen [1,60,73], Vincent Plagnol [79], Rebecca C. Pollitt [80], David J. Porteous [81], Sue Povey [35], Michael A. Quail [1], Lydia Quaye [21], F. Lucy Raymond [11], Karola Rehnström [1], J. Brent Richards [19,21,42,43,58], Cheryl K. Ridout [82], Susan Ring [83], Graham R. S. Ritchie [1,9], Nicola Roberts [11], Rachel L. Robinson [17], David B. Savage [7], Peter Scambler [4], Stephan Schiffels [1], Miriam Schmidts [4,84], Nadia Schoenmakers [7], Richard H. Scott [4,85], Robert K. Semple [7], Eva Serra [1], Sally I. Sharp [47], Adam Shaw [86], Hashem A. Shihab [36], So-Youn Shin [1,36], David Skuse [57], Kerrin S. Small [21], Carol Smee [1], Blair H. Smith [87], George Davey Smith [36], Nicole Soranzo [1,18], Lorraine Southam [1,63], Olivera Spasic-Boskovic [11], Timothy D. Spector [21], David St Clair [88], Beate St Pourcain [36,89,90], Jim Stalker [1], Elizabeth Stevens [16], Jianping Sun [19,42], Gabriela Surdulescu [21], Jaana Suvisaari [60], Petros Syrris [61], Ioanna Tachmazidou [1], Rohan Taylor [55], Jing Tian [34], Nicholas J. Timpson [36], Martin D. Tobin [5,91], Ana M. Valdes [21], Anthony M. Vandersteen [92], Parthiban Vijayarangakannan [1], Peter M. Visscher [37,93], Louise V. Wain [5], Klaudia Walter [1], James T. R. Walters [25], Guangbiao Wang [34], Jun Wang [34,46,94,95,96], Yu Wang [34], Kirsten Ward [21], Eleanor Wheeler [1], Tamieka Whyte [16], Hywel J. Williams [25,97], Kathleen A. Williamson [38], Crispian Wilson[11], Scott G. Wilson [21,98,99], Kim Wong [1], ChangJiang Xu [19,42], Jian Yang [37,93], Eleftheria Zeggini [1], Feng Zhang [21], Pingbo Zhang [34], Hou-Feng Zheng [42,43,58]

Affiliations

1. The Wellcome Trust Sanger Institute, Wellcome Trust Genome Campus, Hinxton CB10 1HH, Cambridge, UK.

2. Department of Pathology, King Abdulaziz Medical City, P.O. Box 22490, Riyadh 11426, Saudi Arabia.

3. Department of Psychiatry, Trinity Centre for Health Sciences, St James Hospital, James's Street, Dublin 8, Ireland.

4. Genetics and Genomic Medicine and Birth Defects Research Centre, UCL Institute of Child Health, London WC1N 1EH, UK.
5. Departments of Health Sciences and Genetics, University of Leicester, Leicester LE1 7RH, UK.
6. Division of Developmental Disabilities, Department of Psychiatry, Queen's University, Kingston, Ontario N6C 0A7, Canada.
7. University of Cambridge Metabolic Research Laboratories, and NIHR Cambridge Biomedical Research Centre, Wellcome Trust-MRC Institute of Metabolic Science, Addenbrooke's Hospital, Cambridge CB2 0QQ, UK.
8. Department of Cardiovascular Medicine and Wellcome Trust Centre for Human Genetics, Roosevelt Drive, Oxford OX3 7BN, UK.
9. European Molecular Biology Laboratory, European Bioinformatics Institute, Wellcome Trust Genome Campus, Hinxton, Cambridge CB10 1SD, UK.
10. Division of Psychiatry, The University of Edinburgh, Royal Edinburgh Hospital, Edinburgh EH10 5HF, UK.
11. Academic Laboratory of Medical Genetics, Box 238, Lv 6 Addenbrooke's Treatment Centre, Addenbrooke's Hospital, Cambridge CB2 0QQ, UK
12. Department of Child Psychiatry, Institute of Psychiatry, Psychology and Neuroscience, King's College London, 16 De Crespigny Park, London SE5 8AF, UK.
13. NIHR BRC for Mental Health, Institute of Psychiatry, Psychology and Neuroscience and SLaM NHS Trust, King's College London, 16 De Crespigny Park, London SE5 8AF, UK.
14. MRC Social, Genetic and Developmental Psychiatry Centre, Institute of Psychiatry, Psychology and Neuroscience, King's College London, Denmark Hill, London SE5 8AF, UK.
15. North East Thames Regional Genetics Service, Great Ormond Street Hospital NHS Foundation Trust, London WC1N 3JH, UK.
16. Dubowitz Neuromuscular Centre, UCL Institute of Child Health & Great Ormond Street Hospital, London WC1N 1EH, UK.
17. Leeds Genetics Laboratory, St James University Hospital, Beckett Street, Leeds LS9 7TF, UK.
18. Department of Haematology, University of Cambridge, Long Road, Cambridge CB2 0PT, UK.
19. Department of Epidemiology, Biostatistics and Occupational Health, McGill University, Montreal, Quebec H3A 1A2, Canada.
20. Institut für Humangenetik, Uniklinik Köln, Kerpener Strasse 34, 50931 Köln, Germany.
21. The Department of Twin Research & Genetic Epidemiology, King's College London, St Thomas' Campus, Lambeth Palace Road, London SE1 7EH, UK.
22. Medical Genetics, Institute for Maternal and Child Health IRCCS "Burlo Garofolo", 34100 Trieste, Italy.
23. Department of Medical, Surgical and Health Sciences, University of Trieste, 34100 Trieste, Italy.
24. Lilly Research Laboratories, Eli Lilly & Co. Ltd., Erl Wood Manor, Sunninghill Road, Windlesham GU20 6PH, UK.
25. MRC Centre for Neuropsychiatric Genetics & Genomics, Institute of Psychological Medicine & Clinical Neurosciences, School of Medicine, Cardiff University, Cardiff CF24 4HQ, UK.
26. Sheffield Diagnostic Genetics Service, Sheffield Childrens' NHS Foundation Trust, Western Bank, Sheffield S10 2TH, UK.
27. University of Sussex, Brighton BN1 9RH, UK.
28. Sussex Partnership NHS Foundation Trust, Swandean, Arundel Road, Worthing BN13 3EP, UK.
29. University College London (UCL), UCL Genetics Institute, Darwin Building, Gower Street, London WC1E 6BT, UK.

30. Bristol Genetic Epidemiology Laboratories, School of Social and Community Medicine, University of Bristol, Oakfield House, Oakfield Grove, Clifton, Bristol BS8 2BN, UK.
31. Computational Biology & Genomics, Biogen Idec, 14 Cambridge Center, Cambridge, Massachusetts 02142, USA.
32. Institute of Cardiovascular and Medical Sciences, University of Glasgow, Wolfson Medical School Building, University Avenue, Glasgow, UK G12 8QQ
33. Department of Medical and Molecular Genetics, Division of Genetics and Molecular Medicine, King's College London School of Medicine, Guy's Hospital, London SE1 9RT, UK.
34. BGI-Shenzhen, Shenzhen 518083, China.
35. University College London (UCL) Department of Genetics, Evolution & Environment (GEE), Gower Street, London WC1E 6BT, UK.
36. MRC Integrative Epidemiology Unit, School of Social and Community Medicine, University of Bristol, Oakfield House, Oakfield Grove, Clifton, Bristol BS8 2BN, UK.
37. University of Queensland Diamantina Institute, Translational Research Institute, Brisbane, Queensland 4102, Australia.
38. MRC Human Genetics Unit, MRC Institute of Genetics and Molecular Medicine, at the University of Edinburgh, Western General Hospital, Edinburgh EH4 2XU, UK.
39. The Genome Centre, John Vane Science Centre, Queen Mary, University of London, Charterhouse Square, London EC1M 6BQ, UK.
40. Cardiovascular Genetics, BHF Laboratories, Rayne Building, Institute of Cardiovascular Sciences, University College London, London WC1E 6JJ, UK.
41. UCLA David Geffen School of Medicine, Los Angeles, California 90095, USA.
42. Lady Davis Institute, Jewish General Hospital, Montreal, Quebec H3T 1E2, Canada.
43. Department of Human Genetics, McGill University, Montreal, Quebec H3A 1B1, Canada.
44. Department of Oncology, McGill University, Montreal, Quebec H2W 1S6, Canada.
45. HeLEX – Centre for Health, Law and Emerging Technologies, Nuffield Department of Population Health, University of Oxford, Old Road Campus, Oxford OX3 7LF, UK.
46. Department of Biology, University of Copenhagen, Ole Maaløes Vej 5, DK-2200 Copenhagen, Denmark.
47. University College London (UCL), Molecular Psychiatry Laboratory, Division of Psychiatry, Gower Street, London WC1E 6BT, UK.
48. Department of Mathematical and Statistical Sciences, University of Colorado, Denver, Colorado 80204, USA.
49. Adaptive Biotechnologies Corporation, Seattle, Washington 98102, USA.
50. Human Genetics Research Centre, St George's University of London, London SW17 0RE, UK.
51. Analytic and Translational Genetics Unit, Massachusetts General Hospital, Boston, Massachusetts 02114, USA.
52. Program in Medical and Population Genetics, Broad Institute of Harvard and MIT, Cambridge, Massachusetts 02142, USA.
53. National Cancer Research Institute, Angel Building, 407 St John Street, London EC1V 4AD, UK.
54. Genetic Alliance UK, 4D Leroy House, 436 Essex Road, London N1 3QP, UK.
55. SW Thames Regional Genetics Lab, St George's University, Cranmer Terrace, London SW17 0RE, UK.
56. Schools of Mathematics and Social and Community Medicine, University of Bristol, Oakfield House, Oakfield Grove, Clifton, Bristol BS8 2BN, UK.
57. Behavioural and Brain Sciences Unit, UCL Institute of Child Health, London WC1N 1EH, UK.

58. Department of Medicine, Jewish General Hospital, McGill University, Montreal, Quebec H3A 1B1, Canada.
59. BGI-Europe, London EC2M 4YE, UK.
60. National Institute for Health and Welfare (THL), Helsinki FI-00271, Finland.
61. Institute of Cardiovascular Science, University College London, Gower Street, London WC1E 6BT, UK.
62. Cardiovascular Centre of the University of Lisbon, Faculty of Medicine, University of Lisbon, Avenida Professor Egas Moniz,1649-028 Lisbon, Portugal.
63. Wellcome Trust Centre for Human Genetics, Roosevelt Drive, Oxford OX3 7BN, UK.
64. Illumina Cambridge Ltd, Chesterford Research Park, Cambridge CB10 1XL, UK.
65. National Institute for Health Research (NIHR) Biomedical Research Centre at Guy's and St Thomas' Foundation Trust, London SE1 9RT, UK.
66. Department of Statistics, University of Oxford, 1 South Parks Road, Oxford OX1 3TG, UK.
67. Department of Genetics, Harvard Medical School, Boston, Massachusetts 02115, USA.
68. The Patrick Wild Centre, The University of Edinburgh, Edinburgh EH10 5HF, UK.
69. Department of Medical Sciences, University of Torino, 10124 Torino, Italy.
70. Institute of Health Informatics, Farr Institute of Health Informatics Research, University College London (UCL), 222 Euston Road, London NW1 2DA, UK.
71. Usher Institute of Population Health Sciences and Informatics, University of Edinburgh, 9 Little France Road, Edinburgh, UK EH16 4UX.
72. Department of Mathematics, Université de Québec À Montréal, Montréal, Québec H3C 3P8, Canada.
73. Institute for Molecular Medicine Finland (FIMM), University of Helsinki, Helsinki FI-00014, Finland.
74. Program in Medical and Population Genetics and Genetic Analysis Platform, The Broad Institute of MIT and Harvard, Cambridge, Massachusetts 02132, USA.
75. Institute of Neuroscience, Henry Wellcome Building for Neuroecology, Newcastle University, Framlington Place, Newcastle upon Tyne NE2 4HH, UK.
76. University of Helsinki, Department of Psychiatry, Helsinki FI-00014, Finland.
77. North West Thames Regional Genetics Service, Kennedy-Galton Centre, Northwick Park Hospital, Watford Road, Harrow HA1 3UJ, UK.
78. MRC Epidemiology Unit, University of Cambridge School of Clinical Medicine, Box 285, Institute of Metabolic Science, Cambridge Biomedical Campus, Cambridge CB2 0QQ, UK.
79. University College London (UCL) Genetics Institute (UGI) Gower Street, London WC1E 6BT, UK.
80. Connective Tissue Disorders Service, Sheffield Diagnostic Genetics Service, Sheffield Children's NHS Foundation Trust, Western Bank, Sheffield S10 2TH, UK.
81. Centre for Genomic and Experimental Medicine, Institute of Genetics and Experimental Medicine, University of Edinburgh, Western General Hospital, Crewe Road, Edinburgh, UK EH4 2XU.
82. Molecular Genetics, Viapath at Guy's Hospital, London SE1 9RT, UK.
83. ALSPAC & School of Social and Community Medicine, University of Bristol, Oakfield House, Oakfield Grove, Clifton, Bristol BS8 2BN, UK.
84. Human Genetics Department, Radboudumc and Radboud Institute for Molecular Life Sciences (RIMLS), Geert Grooteplein 25, Nijmegen 6525 HP, The Netherlands.
85. Department of Clinical Genetics, Great Ormond Street Hospital, London, WC1N 3JH, UK.
86. Clinical Genetics, Guy's & St Thomas' NHS Foundation Trust, London SE1 9RT, UK.
87. Mackenzie Building, Kirsty Semple Way, Ninewells Hospital and Medical School, Dundee, UK DD2 4RB
88. Institute of Medical Sciences, University of Aberdeen, Aberdeen AB25 2ZD, UK.
89. School of Oral and Dental Sciences, University of Bristol, Lower Maudlin Street, Bristol BS1 2LY, UK.

90. School of Experimental Psychology, University of Bristol, 12a Priory Road, Bristol BS8 1TU, UK.
91. National Institute for Health Research (NIHR) Leicester Respiratory Biomedical Research Unit, Glenfield Hospital, Leicester LE3 9QP, UK.
92. Maritime Medical Genetics Service, 5850/5980 University Avenue, PO Box 9700, Halifax, Nova Scotia B3K 6R8, Canada.
93. Queensland Brain Institute, University of Queensland, Brisbane, Queensland 4072, Australia.
94. Princess Al Jawhara Albrahim Center of Excellence in the Research of Hereditary Disorders, King Abdulaziz University, P.O. Box 80200, Jeddah 21589, Saudi Arabia.
95. Macau University of Science and Technology, Avenida Wai long, Taipa, Macau 999078, China.
96. Department of Medicine and State Key Laboratory of Pharmaceutical Biotechnology, University of Hong Kong, 21 Sassoon Road, Hong Kong.
97. The Centre for Translational Omics – GOSgene, UCL Institute of Child Health, London WC1N 1EH, UK.
98. School of Medicine and Pharmacology, University of Western Australia, Perth, Western Australia 6009, Australia.
99. Department of Endocrinology and Diabetes, Sir Charles Gairdner Hospital, Nedlands, Western Australia 6009, Australia.

The arcOGEN Consortium

John Loughlin[1], Nigel Arden[2], Fraser Birrell[3,4], Andrew Carr[2], Panos Deloukas[5,6], Michael Doherty[7], Andrew W. McCaskie[8,9], William E. R. Ollier[10], Ashok Rai[11], Stuart H. Ralston[12], Tim D. Spector[13], Ana M. Valdes[7], Gillian A. Wallis[14], J. Mark Wilkinson[15], Eleftheria Zeggini[16].

Affiliations

1. Musculoskeletal Research Group, Institute of Cellular Medicine, Newcastle University, Newcastle-upon-Tyne, NE2 4HH, UK.
2. Botnar Research Centre, University of Oxford, Nuffield Orthopaedic Centre, Oxford, OX3 7LD UK.
3. Musculoskeletal Research Group, Institute of Cellular Medicine, Newcastle University, Newcastle upon-Tyne, NE2 4HH, UK.
4. Northumbria Healthcare NHS Foundation Trust, Wansbeck General Hospital, NE63 9JJ, UK.
5. William Harvey Research Institute, Barts and The London School of Medicine and Dentistry, Queen Mary University, London, EC1M 6BQ, UK.
6. Princess Al-Jawhara Al-Brahim Centre of Excellence in Research of Hereditary Disorders (PACER-HD), King Abdulaziz University Jeddah, 21589, Saudi Arabia.
7. Academic Rheumatology, School of Medicine, University of Nottingham, UK, Nottingham, NG5 1PB, UK.
8. Division of Trauma and Orthopaedic Surgery, Department of Surgery, University of Cambridge Cambridge, CB2 0QQ, UK.
9. Musculoskeletal Research Group, Institute of Cellular Medicine, Newcastle University Newcastle-upon-Tyne, NE2 4HH, UK.
10. Centre for Integrated Genomic Medical Research, University of Manchester, Manchester, M13 9PT, UK.
11. Worcestershire Acute Hospitals NHS Trust, Worcester, UK.
12. Centre for Genomic and Experimental Medicine, Institute of Genetics and Molecular Medicine, University of Edinburgh, Edinburgh, EH4 2XU, UK.
13. Department of Twin Research and Genetic Epidemiology, King's College London, London, SE1 7EH, UK.
14. Wellcome Trust Centre for Cell Matrix Research, University of Manchester, Manchester M13 9PT UK.

15. Department of Oncology and Metabolism, University of Sheffield, Sheffield, UK.
16. Wellcome Trust Sanger Institute, Wellcome Genome Campus, Hinxton, CB10 1HH, UK.

The Understanding Society Scientific Group
Michaela Benzeval[1], Jonathan Burton[1], Nicholas Buck[1], Annette Jäckle[1], Meena Kumari[1], Heather Laurie[1], Peter Lynn[1], Stephen Pudney[1], Birgitta Rabe[1], Dieter Wolke[2].

Affiliations
1. Institute for Social and Economic Research, University of Essex, Colchester, CO4 3SQ, UK.
2. Department of Psychology, University of Warwick, Coventry, CV4 7AL, UK.

The GoT2D Consortium
Jason Flannick[1, 2 ,*], Hyun Min Kang[3,*], Kyle J Gaulton[4,*], Vineeta Agarwala[2, 5,*], Clement Ma[3,*], Davis J McCarthy[4, 6], Loukas Moutsianas[4], Noël P Burtt[2], Pierre Fontanillas[2], Thomas W Blackwell[3], Adam E Locke[3], Richard D Pearson[4], Ashish Kumar[4, 7], Christopher Hartl[2], Michael L Stitzel[8], Stephen C J Parker[9], Yuhui Chen[4], Peter S Chines[10], Jeroen R Huyghe[3], Anne U Jackson[3], Cecilia M Lindgren[2, 4], John R B Perry[4, 11-13], N William Rayner[4, 14, 15], Manuel A Rivas[4], Neil R Robertson[4, 15], Xueling Sim[3], Heather M Stringham[3], Tanya M Teslovich[3], Benjamin F Voight[16], Martijn van de Bunt[4, 15], Anubha Mahajan[4], Todd Green[2], Beverley Balkau[17], Heiner Boeing[18], Erwin P Bottinger[19], Han Chen[20, 21], Pablo Cingolani[22, 23], Josee Dupuis[21, 24], Paul W Franks[25-27], Philippe Froguel[28], Vilmantas Giedraitis[29], Omri Gottesman[19], Thomas Illig[30-32], Erik Ingelsson[4, 33, 34], Phoenix Kwan[3], Claudia Langenberg[13], Lars Lind[35], Ruth J F Loos[19], Yingchang Lu[19], Reedik Mägi[36], James B Meigs[37], Andres Metspalu[36, 38], Evelin Mihailov[36, 38], Martina Müller-Nurasyid[39-42], Carmen Navarro[43-45], Domenico Palli[46], Dennis Rybin[21], Robert A Scott[13], Rob Sladek[22, 47, 48], Ann-Christine Syvänen[49], Dorothée Thuillier[28], Yvonne T van der Schouw[50], Nicholas J Wareham[13], Loïc Yengo[28], Graeme I Bell[51], John Blangero[52], Nancy J Cox[53], Ravindranath Duggirala[52], Craig L Hanis[54], Mark Seielstad[55, 56], Lori L Bonnycastle[10], João Fadista[57], Christopher J Groves[15], Christian Herder[58, 59], Leena Kinnunen[60], Heikki A Koistinen[60-63], Jasmina Kravic[57], Claes Ladenvall[57], Valeri Lyssenko[57], Narisu Narisu[10], Katharine R Owen[15, 64], Wolfgang Rathmann[65], Michael Roden[59, 66], Kerrin S Small[11], Amy Swift[10], Barbara Thorand[67, 68], Richard N Bergman[69], Francis S Collins[10], Timothy M Frayling[12], Christian Gieger[40], Andrew T Hattersley[70], Martin Hrabé de Angelis[67, 71, 72], Christa Meisinger[68], Peter Nilsson[73], Annette Peters[41, 67, 68], Timothy D Spector[11], Tiinamaija Tuomi[63, 74-76], Jaakko Tuomilehto[60, 77-79], Richard M Watanabe[80-82], Eric Banks[2], David Buck[83], Gemma Buck[83], Mark DePristo[2], Timothy Fennell[2], Stacey Gabriel[2], Harald Grallert[30, 67, 68], Cornelia Huth[67, 68], Eric S Lander[84], Jared Maguire[2], Ryan Poplin[2], Janina S Ried[40], Khalid Shakir[2], Joseph Trakalo[83], Konstantin Strauch[39, 40], Andrew D Morris[85], Gilean McVean[4], Jose C Florez[2, 86-88], Karen L Mohlke[89], Peter J Donnelly[4, 6], Tim M Strom[90, 91], Andrew P Morris[4, 36, 92], Inga Prokopenko[4, 15, 93], Gonçalo Abecasis[3], Leif Groop[57, 74, 76], Laura J Scott[3], Thomas Meitinger[90, 91], Mark I McCarthy[4, 15, 64,*], Michael Boehnke[3,*], David Altshuler[1, 2, 86, 88, 94, 95,*], Christian Fuchsberger[3,*]

Affiliations
1. Department of Molecular Biology, Massachusetts General Hospital, Boston, Massachusetts, USA.
2. Program in Medical and Population Genetics, Broad Institute, Cambridge, Massachusetts, USA.
3. Department of Biostatistics and Center for Statistical Genetics, University of Michigan, Ann Arbor, Michigan, USA.
4. Wellcome Trust Centre for Human Genetics, Nuffield Department of Medicine, University of Oxford, Oxford, UK.
5. Harvard-MIT Division of Health Sciences and Technology, Massachusetts Institute of Technology, Cambridge, Massachusetts, USA.
6. Department of Statistics, University of Oxford, Oxford, UK.
7. Chronic Disease Epidemiology, Swiss Tropical and Public Health Institute, University of Basel, Basel, Switzerland.
8. The Jackson Laboratory for Genomic Medicine, Farmington, CT, USA.

9. Departments of Computational Medicine & Bioinformatics and Human Genetics, University of Michigan, Ann Arbor, Michigan, USA.
10. Medical Genomics and Metabolic Genetics Branch, National Human Genome Research Institute, National Institutes of Health, Bethesda, Maryland, USA.
11. Department of Twin Research and Genetic Epidemiology, King's College London, London, UK.
12. Genetics of Complex Traits, University of Exeter Medical School, University of Exeter, Exeter, UK.
13. MRC Epidemiology Unit, Institute of Metabolic Science, University of Cambridge, Cambridge, UK.
14. Department of Human Genetics, Wellcome Trust Sanger Institute, Hinxton, Cambridgeshire, UK.
15. Oxford Centre for Diabetes, Endocrinology and Metabolism, Radcliffe Department of Medicine, University of Oxford, Oxford, UK.
16. Department of Medicine, University of Pennsylvania, Philadelphia, Pennsylvania, USA.
17. Centre for Research in Epidemiology and Population Health, Inserm U1018, Villejuif, France.
18. German Institute of Human Nutrition Potsdam-Rehbruecke, Nuthetal, Germany.
19. The Charles Bronfman Institute for Personalized Medicine, The Icahn School of Medicine at Mount Sinai, New York, NY, USA.
20. Department of Biostatistics, Harvard School of Public Health, Boston, Massachusetts, USA.
21. Department of Biostatistics, Boston University School of Public Health, Boston, Massachusetts, USA.
22. McGill University and Génome Québec Innovation Centre, Montreal, Quebec, Canada.
23. School of Computer Science, McGill University, Montreal, Quebec, Canada.
24. National Heart, Lung, and Blood Institute's Framingham Heart Study, Framingham, Massachusetts, USA.
25. Department of Nutrition, Harvard School of Public Health, Boston, Massachusetts, USA.
26. Department of Public Health and Clinical Medicine, Umeå University, Umeå, Sweden.
27. Department of Clinical Sciences, Lund University Diabetes Centre, Genetic and Molecular Epidemiology Unit, Lund University, Malmö, Sweden.
28. Integrative Genomics and Modelization of Metabolic Diseases CNRS UMR8199, Lille Institute of Biology, E.G.I.D - FR3508 European Genomics Institute of Diabetes, Lille, France.
29. Department of Public Health and Caring Sciences, Geriatrics, Uppsala University, Uppsala, Sweden.
30. Research Unit of Molecular Epidemiology, Helmholtz Zentrum München, German Research Center for Environmental Health, Neuherberg, Germany.
31. Hannover Unified Biobank, Hannover Medical School, Hanover, Germany.
32. Institute for Human Genetics, Hannover Medical School, Hanover, Germany.
33. Division of Cardiovascular Medicine, Department of Medicine, Stanford University School of Medicine, Stanford, CA, USA.
34. Department of Medical Sciences, Molecular Epidemiology and Science for Life Laboratory, Uppsala University, Uppsala, Sweden.
35. Department of Medical Sciences, Uppsala University, Uppsala, Sweden.
36. Estonian Genome Center, University of Tartu, Tartu, Estonia.
37. General Medicine Division, Massachusetts General Hospital and Department of Medicine, Harvard Medical School, Boston, Massachusetts, USA.
38. The Institute of Molecular and Cell Biology, University of Tartu, Tartu, Estonia.
39. Institute of Medical Informatics, Biometry and Epidemiology, Chair of Genetic Epidemiology, Ludwig-Maximilians-Universität, Neuherberg, Germany.
40. Institute of Genetic Epidemiology, Helmholtz Zentrum München, German Research Center for Environmental Health, Neuherberg, Germany.
41. Deutsches Forschungszentrum für Herz-Kreislauferkrankungen (DZHK), Partner Site Munich Heart Alliance, Munich, Germany.
42. Department of Medicine I, University Hospital Grosshadern, Ludwig-Maximilians-Universität, Munich, Germany.
43. Unit of Preventive Medicine and Public Health, School of Medicine, University of Murcia, Spain.
44. Department of Epidemiology, Murcia Regional Health Council, Murcia, Spain.

45. CIBER Epidemiología y Salud Pública (CIBERESP), Spain.
46. Cancer Research and Prevention Institute (ISPO), Florence, Italy.
47. Department of Human Genetics, McGill University, Montreal, Quebec, Canada.
48. Division of Endocrinology and Metabolism, Department of Medicine, McGill University, Montreal, Quebec, Canada.
49. Department of Medical Sciences, Molecular Medicine and Science for Life Laboratory, Uppsala University, Uppsala, Sweden.
50. Julius Center for Health Sciences and Primary Care, University Medical Center Utrecht, Utrecht, Netherlands.
51. Departments of Medicine and Human Genetics, The University of Chicago, Chicago, Illinois, USA.
52. Department of Genetics, Texas Biomedical Research Institute, San Antonio, Texas, USA.
53. Department of Medicine, Section of Genetic Medicine, The University of Chicago, Chicago, Illinois, USA.
54. Human Genetics Center, School of Public Health, The University of Texas Health Science Center at Houston, Houston, Texas, USA.
55. Department of Laboratory Medicine & Institute for Human Genetics, University of California, San Francisco, San Francisco, California, USA.
56. Blood Systems Research Institute, San Francisco, California, USA.
57. Department of Clinical Sciences, Diabetes and Endocrinology, Lund University Diabetes Centre, Malmö, Sweden.
58. German Center for Diabetes Research (DZD), partner Düsseldorf, Düsseldorf, Germany.
59. Institute of Clinical Diabetology, German Diabetes Center, Leibniz Center for Diabetes Research at Heinrich Heine University, Düsseldorf, Germany.
60. Department of Health, National Institute for Health and Welfare, Helsinki, Finland.
61. Department of Medicine, University of Helsinki and Helsinki University Central Hospital, Helsinki, Finland.
62. Minerva Foundation Institute for Medical Research, Helsinki, Finland.
63. Abdominal Center: Endocrinology, University of Helsinki and Helsinki University Central Hospital, Helsinki, Finland.
64. Oxford NIHR Biomedical Research Centre, Oxford University Hospitals Trust, Oxford, UK.
65. Institute for Biometrics and Epidemiology, German Diabetes Center, Leibniz Center for Diabetes Research at Heinrich Heine University, Düsseldorf, Germany.
66. Department of Endocrinology and Diabetology, Medical Faculty, Heinrich-Heine University, Düsseldorf, Germany.
67. German Center for Diabetes Research (DZD), Neuherberg, Germany.
68. Institute of Epidemiology II, Helmholtz Zentrum München, German Research Center for Environmental Health, Neuherberg, Germany.
69. Cedars-Sinai Diabetes and Obesity Research Institute, Los Angeles, California, USA.
70. University of Exeter Medical School, University of Exeter, Exeter, UK.
71. Center of Life and Food Sciences Weihenstephan, Technische Universität München, Freising-Weihenstephan, Germany.
72. Institute of Experimental Genetics, Helmholtz Zentrum München, German Research Center for Environmental Health, Neuherberg, Germany.
73. Department of Clinical Sciences, Medicine, Lund University, Malmö, Sweden.
74. Finnish Institute for Molecular Medicine, University of Helsinki, Helsinki, Finland.
75. Folkhälsan Research Centre, Helsinki, Finland.
76. Research Programs Unit, Diabetes and Obesity, University of Helsinki, Helsinki, Finland.
77. Center for Vascular Prevention, Danube University Krems, Krems, Austria.
78. Instituto de Investigacion Sanitaria del Hospital Universario LaPaz (IdiPAZ), University Hospital LaPaz, Autonomous University of Madrid, Madrid, Spain.
79. Diabetes Research Group, King Abdulaziz University, Jeddah, Saudi Arabia.

80. Department of Physiology & Biophysics, Keck School of Medicine, University of Southern California, Los Angeles, California, USA.
81. Department of Preventive Medicine, Keck School of Medicine, University of Southern California, Los Angeles, California, USA.
82. Diabetes and Obesity Research Institute, Keck School of Medicine, University of Southern California, Los Angeles, California, USA.
83. High Throughput Genomics, Oxford Genomics Centre, Wellcome Trust Centre for Human Genetics, Nuffield Department of Medicine, University of Oxford, Oxford, UK.
84. Broad Institute of MIT and Harvard, Cambridge, Massachusetts, USA.
85. The Usher Institute of Population Health Sciences and Informatics, University of Edinburgh, Edinburgh, UK.
86. Diabetes Research Center (Diabetes Unit), Department of Medicine, Massachusetts General Hospital, Boston, Massachusetts, USA.
87. Center for Human Genetic Research, Department of Medicine, Massachusetts General Hospital, Boston, Massachusetts, USA.
88. Department of Medicine, Harvard Medical School, Boston, Massachusetts, USA.
89. Department of Genetics, University of North Carolina, Chapel Hill, North Carolina, USA.
90. Institute of Human Genetics, Technische Universität München, Munich, Germany.
91. Institute of Human Genetics, Helmholtz Zentrum München, German Research Center for Environmental Health, Neuherberg, Germany.
92. Department of Biostatistics, University of Liverpool, Liverpool, UK.
93. Department of Genomics of Common Disease, School of Public Health, Imperial College London, London, UK.
94. Department of Genetics, Harvard Medical School, Boston, Massachusetts, USA.
95. Department of Biology, Massachusetts Institute of Technology, Cambridge, Massachusetts, USA.

* These authors led the project and the writing of the paper.

Current Addresses
David Altshuler: Vertex Pharmaceuticals, Boston, MA, 02210, USA.

Nancy J Cox: Vanderbilt Genetics Institute, Vanderbilt University Medical Center, Nashville, TN, 37232, USA.

Cecilia M Lindgren: The Big Data Institute at the Li Ka Shing Centre for Health Information and Discovery, University of Oxford, Oxford OX3 7BN, UK.

Ravindranath Duggirala, John Blangero: South Texas Diabetes and Obesity Institute, Edinburg Regional Academic Health Center, University of Texas Health Science Center at San Antonio/University of Texas Rio Grande Valley, Edinburg, TX, 78541, USA.

The SpiroMeta Consortium

Abdul K. Kheirallah[1], Alan F. Wright[2], Alan L. James[3,4,5], Alexander Teumer[6,7], Alexessander C. Alves[8], Anna-Liisa Hartikainen[9], Arthur W. Musk[3,5,10], Åsa Johansson[11,12], Ashish Kumar[13,14,15,16], Beate Koch[17], Caroline Hayward[2], Christian Gieger[18,19,20], David P. Strachan[21], Eva Albrecht[20], Generation Scotland[22], Harald Grallert[18], Harry Campbell[23], Henry Völzke[6], Holger Schulz[24,25], Holly Trochet[2], Ian J. Deary[26,27], Ian P. Hall[1], Ian Sayers[1], Ida Surakka[28,29], Igor Rudan[23,30], Ioanna Ntalla[31], Ivana Kolcic[32], James F. Wilson[23], Janina Ried[20], Jennie Hui[3,33,34,35], Jennifer E. Huffman[2], Jing H. Zhao[36], Joachim Heinrich[24,25,37], John Beilby[3,33,35], John M. Starr[26,38], Jonathan Marten[2], Leo-Pekka Lyytikäinen[39,40], Lorna M. Lopez[26,27], Louise V. Wain[31], Lynne Hocking[41], María S. Artigas[31], Marjo-Riitta Jarvelin[8,29,42,43], Markku Heliövaara[29], Martin D. Tobin[31,44], Medea Imboden[13,14], Mika Kähönen[45], Momoko Horikoshi[15,46], Nicholas J. Wareham[36], Nicole M. Probst-

Hensch[13,14], Nina Hutri-Kähönen[47,48], Olli T. Raitakari[49,50], Ozren Polasek[23,32], Pau Navarro[2], Peter K. Joshi[23], Rajesh Rawal[18,19,20], Robert A. Scott[36], Samuli Ripatti[28,51,52], Sandosh Padmanabhan[53], Sarah E. Harris[26,54,55], Sarah H. Wild[23], Stefan Enroth[11], Stefan Karrasch[24,56,57], Suzanne Miller[1], Sven Gläser[17], Tatijana Zemunik[58], Terho Lehtimäki[39,40], Ulf Gyllensten[11], Veronique Vitart[2], Wendy L. McArdle[59]

Affiliations

1. Division of Respiratory Medicine, Queen's Medical Centre, University of Nottingham, Nottingham, UK
2. MRC Human Genetics Unit, MRC Institute of Genetics and Molecular Medicine, University of Edinburgh, Edinburgh, Scotland, UK
3. Busselton Population Medical Research Institute, Busselton, Western Australia, Australia
4. Department of Pulmonary Physiology and Sleep Medicine, Sir Charles Gairdner Hospital, Western Australia, Australia
5. School of Medicine and Pharmacology, The University of Western Australia, Western Australia, Australia
6. University Medicine Greifswald, Community Medicine, SHIP - Clinical Epidemiological Research, Greifswald, Germany
7. University Medicine Greifswald, Interfaculty Institute for Genetics and Functional Genomics, Department for Genetics and Functional Genomics, Greifswald, Germany
8. Department of Epidemiology and Biostatistics, School of Public Health, Imperial College London, London, UK
9. Department of Obstetrics and Gynecology of Oulu University Hospital ,MRC of Oulu University, Oulu, Finland
10. Department of Respiratory Medicine, Sir Charles Gairdner Hospital, Western Australia, Australia
11. Department of Immunology, Genetics, and Pathology, Biomedical Center, SciLifeLab Uppsala, Uppsala University, Uppsala, Sweden
12. Uppsala Clinical Research Centre, Uppsala University, Uppsala, Sweden
13. Swiss Tropical and Public Health Institute, Basel, Switzerland
14. University of Basel, Switzerland
15. Wellcome Trust Centre for Human Genetics, University of Oxford, Oxford, UK
16. Institute of Environmental Medicine, Karolinska Institutet, Stockholm, Sweden
17. University Medicine Greifswald, Internal Medicine B, Pneumology, Cardiology, Intensive Care, Weaning, Field of Research: Pneumological Epidemiology, Greifswald, Germany
18. Research Unit of Molecular Epidemiology, Helmholtz Zentrum München, German Research Center for Environmental Health, Neuherberg, Germany
19. Institute of Epidemiology II, Helmholtz Zentrum München German research center for environmental health, Neuherberg, Germany
20. Institute of Genetic Epidemiology, Helmholtz Zentrum München German research center for environmental health, Neuherberg, Germany
21. Population Health Research Institute, St George's, University of London, Cranmer Terrace, London, UK
22. Generation Scotland, A Collaboration between the University Medical Schools and NHS, Aberdeen, Dundee, Edinburgh and Glasgow, UK
23. Institute for Population Health Sciences and Informatics, University of Edinburgh, Teviot Place, Edinburgh, Scotland, UK
24. Institute of Epidemiology I, Helmholtz Zentrum München, German Research Center for Environmental Health, Neuherberg, Germany
25. Comprehensive Pneumology Center Munich (CPC-M), Member of the German Center for Lung Research, Munich, Germany
26. Centre for Cognitive Ageing and Cognitive Epidemiology, University of Edinburgh, Edinburgh, UK
27. Psychology, University of Edinburgh, Edinburgh, UK
28. Institute for Molecular Medicine Finland (FIMM), University of Helsinki, Helsinki, Finland
29. The National Institute for Health and Welfare (THL), Helsinki, Finland

30.Centre for Population Health Sciences, Medical School, University of Edinburgh, Edinburgh, Scotland, UK
31.Genetic Epidemiology Group, Department of Health Sciences, University of Leicester, Leicester, UK
32.Department of Public Health, Faculty of Medicine, University of Split, Split, Croatia
33.PathWest Laboratory Medicine WA, Sir Charles Gairdner Hospital, Western Australia, Australia
34.School of Population Health, The University of Western Australia, Western Australia, Australia
35.School of Pathology and Laboratory Medicine, The University of Western Australia, Western Australia, Australia
36.MRC Epidemiology Unit, University of Cambridge School of Clinical Medicine, Institute of Metabolic Science, Cambridge Biomedical Campus, Cambridge, UK
37.University Hospital Munich, Institute and Outpatient Clinic for Occupational, Social and Environmental Medicine, Ludwig-Maximilian University Munich, Munich, Germany
38.Alzheimer Scotland Research Centre, University of Edinburgh, Edinburgh, UK
39.Department of Clinical Chemistry, Fimlab Laboratories, Tampere, Finland
40.Department of Clinical Chemistry, University of Tampere School of Medicine, Tampere, Finland
41.Division of Applied Health Sciences, University of Aberdeen, Aberdeen, Scotland, UK
42.Institute of Health Sciences, University of Oulu, Oulu, Finland
43.Biocenter Oulu, University of Oulu, Oulu, Finland
44.National Institute for Health Research (NIHR) Leicester Respiratory Biomedical Research Unit, Glenfield Hospital, Leicester, UK
45.Department of Clinical Physiology, University of Tampere and Tampere University Hospital, Tampere, Finland
46.Oxford Centre for Diabetes, Endocrinology and Metabolism, University of Oxford, Oxford, UK
47.Department of Pediatrics, Tampere University Hospital, Tampere, Finland
48.Department of Pediatrics, University of Tampere School of Medicine, Tampere, Finland
49.Department of Clinical Physiology and Nuclear Medicine, Turku University Hospital, Turku, Finland
50.Research Centre of Applied and Preventive Cardiovascular Medicine, University of Turku, Turku, Finland
51.Department of Public Health, University of Helsinki, Helsinki, Finland
52.Department of Human Genomics, Wellcome Trust Sanger Institute, Hinxton, Cambridge, UK
53.Division of Cardiovascular and Medical Sciences, University of Glasgow, Glasgow, Scotland, UK
54.Centre for Genomic and Experimental Medicine, University of Edinburgh, Edinburgh, UK
55.Unit of Primary Care, Oulu University Hospital, Oulu, Finland
56.Institute of General Practice, University Hospital Klinikum rechts der Isar, Technische Universität München, Munich, Germany
57.Institute and Outpatient Clinic for Occupational, Social and Environmental Medicine, Ludwig-Maximilians-Universität, Munich, Germany
58.Department of Medical Biology, Faculty of Medicine, University of Split, Croatia, Split, Croatia
59.School of Social and Community Medicine, University of Bristol, Bristol, UK

ACKNOWLEDGEMENTS AND GRANT SUPPORT

AS Butterworth: The Cardiovascular Epidemiology Unit is underpinned by grants from the British Heart Foundation (SP/09/002), the UK Medical Research Council (G0800270), the UK National Institute for Health Research Cambridge Biomedical Centre, the European Research Council (268834) and the European Commission Framework Programme 7 (HEALTH-F2-2012-279233). The National Institute for Health Research Blood and Transplant Unit (NIHR BTRU) in Donor Health and Genomics at the University of Cambridge is supported by the National Institute of Health Research. *J Luan, C Langenberg, RA Scott, NJ Wareham:* The Medical Research Council (MC_UU_12015/1). *E Zeggini:* The Wellcome Trust (WT098051) and the European Research Council (ERC-2011-StG 280559-SEPI). *B Prins:* Economic & Social Research Council (ES/H029745/1) and the Wellcome Trust (WT098051). *V Salomaa:* Academy of Finland

(136895 and 263836), Finnish Foundation for Cardiovascular Research. *V Jaddoe:* The Netherlands Organization for Health Research and Development (NWO, ZonMw-VIDI 016.136.361), European Research Council Consolidator Grant (ERC-2014-CoG-648916). *JF Felix:* European Union Horizon 2020 research and innovation programme, grant agreement number 633595. *K Panoutsopoulou:* Arthritis Research UK Career Development Fellowship (20308). *K Walter:* The Wellcome Trust (WT098051, WT091310). *SG Wilson:* NHMRC (Project Grant 1048216). *AA Crawford:* Wellcome Trust (107049). *BR Walker:* British Heart Foundation (RG/11/4/28734), Scottish Chief Scientist Office (CZB/4/733) and the Wellcome Trust (Senior Investigator Award to BR Walker; 107049/Z/15/Z). *MI McCarthy* is a Wellcome Trust Senior Investigator (Wellcome Trust 098381, 095032). Other grants: Framework VII (HEALTH-F4-2007-201413), Wellcome Trust (090367, 090532), NIDDK (RC2-DK088389, U01-DK085545, R01-DK098032). *L Paternoster* is funded by an MRC Population Health Scientist Fellowship MR/J012165/1. *CM Lindgren* is supported by WT Awards 086596/Z/08/A, 086596/Z/08/Z and Li Ka Shing Funds. *AP Morris* is a Wellcome Trust Senior Fellow in Basic Biomedical Science (under grant WT098017). *N Soranzo* is supported by the Wellcome Trust (Grant Codes WT098051 and WT091310), the EU FP7 (EPIGENESYS Grant Code 257082 and BLUEPRINT Grant Code HEALTH-F5-2011-282510) and the National Institute for Health Research Blood and Transplant Research Unit (NIHR BTRU) in Donor Health and Genomics at the University of Cambridge in partnership with NHS Blood and Transplant (NHSBT). The views expressed are those of the author(s) and not necessarily those of the NHS, the NIHR, the Department of Health or NHSBT. *MD Tobin* and *MS Artigas* are partially funded by the National Institute for Health Research (NIHR). The views expressed are those of the author(s) and not necessarily those of the NHS, the NIHR or the Department of Health. *MD Tobin* holds a Medical Research Council Senior Clinical Fellowship (G0902313). *NJ Timpson, JL Min, TR Gaunt and GD Smith* work within the MRC Integrative Epidemiology Unit at the University of Bristol (MC_UU_12013/1-9). Research at the Sanger Institute is supported by the Wellcome Trust (WT098051).

AS Butterworth has received grants from Pfizer, Novartis and Merck.
R Durbin is a founder and non-executive director of Congenica Ltd.

ALSPAC: This study makes use of data generated by the UK10K Consortium. The Wellcome Trust provided funding for UK10K (WT091310). Medical Research Council (S. Ring, MC_UU_12015/2, MR/J012165/1 to L. Paternoster, MC_UU_12013/1-9 to N. J. Timpson, G. D. Smith, D. Evans, T. Gaunt, H. Shihab). The UK Medical Research Council and the Wellcome Trust (Grant ref: 102215/2/13/2) and the University of Bristol provide core support for ALSPAC. We are extremely grateful to all the families who took part in this study, the midwives for their help in recruiting them, and the whole ALSPAC team, which includes interviewers, computer and laboratory technicians, clerical workers, research scientists, volunteers, managers, receptionists and nurses. The UK Medical Research Council and the Wellcome Trust (Grant ref: 102215/2/13/2) and the University of Bristol provide core support for ALSPAC. This publication is the work of the authors and they will serve as guarantors for the contents of this paper. GWAS data was generated by Sample Logistics and Genotyping Facilities at the Wellcome Trust Sanger Institute and LabCorp (Laboratory Corportation of America) using support from 23andMe.

TwinsUK: TwinsUK receives support from the National Institute for Health Research (NIHR) BioResource Clinical Research Facility and Biomedical Research Centre based at Guy's and St Thomas' NHS Foundation Trust and King's College London, Wellcome Trust Sanger Institute and National Eye Institute. The Wellcome Trust provided funding for UK10K (WT091310). EU grant EU FP7 (257082, HEALTH-F5-2011-282510). We are extremely grateful to all the participants who took part in this study, those who helped recruitment and the whole TUK team.

UKHLS: The UK Household Longitudinal Study was funded by grants from the Economic & Social Research Council (ES/H029745/1) and the Wellcome Trust (WT098051). UKHLS is led by the Institute for Social and Economic Research at the University of Essex and funded by the Economic and Social Research Council.

The survey was conducted by NatCen and the genome-wide scan data were analysed and deposited by the Wellcome Trust Sanger Institute. Information on how to access the data can be found on the Understanding Society website https://www.understandingsociety.ac.uk/.

FINRISK: FINRISK was funded by the Academy of Finland (136895 and 263836), Finnish Foundation for Cardiovascular Research.

LURIC – controls: We extend our appreciation to the participants of the LURIC study; without their collaboration, this article would not have been written. We thank the LURIC study team who were either temporarily or permanently involved in patient recruitment as well as sample and data handling, in addition to the laboratory staff at the Ludwigshafen General Hospital and the Universities of Freiburg and Ulm, Germany.

TEENAGE: This work was funded by the Wellcome Trust (098051) and has been co-financed by the European Union (European Social Fund—ESF) and Greek national funds through the Operational Program "Education and Lifelong Learning" of the National Strategic Reference Framework (NSRF)—Research Funding Program: Heracleitus II. Investing in knowledge society through the European Social Fund. We thank all study participants and their families as well as all volunteers for their contribution in this study. We thank the following staff from the Sample Management and Genotyping Facilities at the Wellcome Trust Sanger Institute for sample preparation, quality control and genotyping: Dave Jones, Doug Simpkin, Emma Gray, Hannah Blackburn, Sarah Edkins.

Rotterdam Study: The Rotterdam Study is funded by Erasmus Medical Center and Erasmus University, Rotterdam, Netherlands Organization for the Health Research and Development (ZonMw), the Research Institute for Diseases in the Elderly (RIDE), the Ministry of Education, Culture and Science, the Ministry for Health, Welfare and Sports, the European Commission (DG XII), and the Municipality of Rotterdam. The authors are grateful to the study participants, the staff from the Rotterdam Study and the participating general practitioners and pharmacists. This study makes use of sequence reference data generated by the UK10K Consortium, derived from samples from the ALSPAC and TwinsUK datasets. A full list of the investigators who contributed to the generation of the data is available from www.UK10K.org. Funding for UK10K was provided by the Wellcome Trust under award WT091310. We thank Jie Huang at the Wellcome Trust's Sanger Institute, at Hinxton, U.K. for the creation of imputed data, with the support of Marijn Verkerk, Carolina Medina-Gomez, MSc, and Anis Abuseiris and their input for the analysis setup. The generation and management of GWAS genotype data for the Rotterdam Study (RS I, RS II, RS III) was executed by the Human Genotyping Facility of the Genetic Laboratory of the Department of Internal Medicine, Erasmus MC, Rotterdam, The Netherlands. The GWAS datasets are supported by the Netherlands Organisation of Scientific Research NWO Investments (nr. 175.010.2005.011, 911-03-012), the Genetic Laboratory of the Department of Internal Medicine, Erasmus MC, the Research Institute for Diseases in the Elderly (014-93-015; RIDE2), the Netherlands Genomics Initiative (NGI)/Netherlands Organisation for Scientific Research (NWO) Netherlands Consortium for Healthy Aging (NCHA), project nr. 050-060-810. We thank Pascal Arp, Mila Jhamai, Marijn Verkerk, Lizbeth Herrera and Marjolein Peters, MSc, and Carolina Medina-Gomez, MSc, for their help in creating the GWAS database, and Karol Estrada, PhD, Yurii Aulchenko, PhD, and Carolina Medina-Gomez, MSc, for the creation and analysis of imputed data.

HELIC: This work was funded by the Wellcome Trust (098051) and the European Research Council (ERC-2011-StG 280559-SEPI). The MANOLIS cohort is named in honour of Manolis Giannakakis, 1978-2010. We thank the residents of the Mylopotamos villages, and of the Pomak villages, for taking part. The HELIC study has been supported by many individuals who have contributed to sample collection (including A. Athanasiadis, O. Balafouti, C. Batzaki, G. Daskalakis, E. Emmanouil, C. Giannakaki, M. Giannakopoulou, A. Kaparou, V. Kariakli, S. Koinaki, D. Kokori, M. Konidari, H. Koundouraki, D. Koutoukidis, V. Mamakou, E.

Mamalaki, E. Mpamiaki, M. Tsoukana, D. Tzakou, K. Vosdogianni, N. Xenaki, E. Zengini), data entry (T. Antonos, D. Papagrigoriou, B. Spiliopoulou), sample logistics (S. Edkins, E. Gray), genotyping (R. Andrews, H. Blackburn, D. Simpkin, S. Whitehead), research administration (A. Kolb-Kokocinski, S. Smee, D. Walker) and informatics (M. Pollard, J. Randall).

INGI-VB: The research was supported by funds from Compagnia di San Paolo, Torino, Italy; Fondazione Cariplo, Italy and Ministry of Health, Ricerca Finalizzata 2008 and CCM 2010, PRIN 2009 and Telethon, Italy to DT. The funders had no role in study design, data collection and analysis, decision to publish, or preparation of the manuscript. We thank the inhabitants of the VB that made this study possible, the local administrations, the Tortona and Genova archdiocese and the ASL-22, Novi Ligure (Al) for support. We also thank Clara Camaschella, Federico Caligaris-Cappio and the Internal Medicine team of the San Raffaele Hospital for clinical data collection, Fiammetta Viganò for technical help, Corrado Masciullo and Massimiliano Cocca for building the analysis platform.

INGI-FVG and INGI-Carl: Project co-financed by the European Regional Development Fund under the Regional Operational Programme of Friuli Venezia Giulia - Objective "Regional Competitiveness and Employment" 2007/2013,Telethon Foundation (GGP09037), Fondo Trieste (2008), Regione FVG (L.26.2008), andItalian Ministry of Health (RC16/06, ART. 13 D.LGS 297/99) (to PG). We would like to thank the people of the Friuli Venezia Giulia Region and of Carlantino for the everlasting support.

arcOGEN: arcOGEN (http://www.arcogen.org.uk/) was funded by a special purpose grant from Arthritis Research UK (grant 18030).

INCIPE: CARIVR Foundation.

1958 Birth Cohort: The provision of data and biosamples from 1958BC is funded jointly by Wellcome Trust and MRC under grant 108439/Z/15/Z.

LURIC – controls: LURIC was supported by the 7th Framework Program (AtheroRemo, grant agreement number 201668 and RiskyCAD, grant agreement number 305739) of the EU and by the INTERREG-IV-Oberrhein-Program (Project A28, Genetic mechanisms of cardiovascular diseases) with support from the European Regional Development Fund (ERDF) and the Wissenschaftsoffensive TMO. We thank the LURIC study team who were either temporarily or permanently involved in patient recruitment as well as sample and data handling, in addition to the laboratory staff at the Ludwigshafen General Hospital and the Universities of Freiburg and Ulm, Germany.

LOLIPOP: The LOLIPOP study is supported by the National Institute for Health Research (NIHR) Comprehensive Biomedical Research Centre Imperial College Healthcare NHS Trust, the British Heart Foundation (SP/04/002), the Medical Research Council (G0601966, G0700931), the Wellcome Trust (084723/Z/08/Z), the NIHR (RP-PG-0407-10371), European Union FP7 (EpiMigrant, 279143) and Action on Hearing Loss (G51). We thank the National Institute for Health Research (NIHR) Comprehensive Biomedical Research Centre Imperial College Healthcare NHS Trust for support. The work was carried out in part at the NIHR/Wellcome Trust Imperial Clinical Research Facility. We thank the participants and research staff who made the study possible.

SardiNIA: This research was supported by National Human Genome Research Institute grants HG005581, HG005552, HG006513, HG007022 and HG007089; by National Heart, Lung, and Blood Institute grant HL117626; by the Intramural Research Program of the US National Institutes of Health, National Institute on Aging, contracts N01-AG-1-2109 and HHSN271201100005C; by Sardinian Autonomous Region (L.R. 7/2009) grant cRP3-154; by the PB05 InterOmics MIUR Flagship Project; by grant FaReBio2011 ‘Farmaci e

Reti Biotecnologiche di Qualità'. We thank all the volunteers who generously participated in this study and made this research possible.

Fenland: The Fenland Study is funded by the Wellcome Trust and the Medical Research Council (MC_U106179471). We are grateful to all the volunteers for their time and help, and to the General Practitioners and practice staff for assistance with recruitment. We thank the Fenland Study Investigators, Fenland Study Co-ordination team and the Epidemiology Field, Data and Laboratory teams. We further acknowledge support from the Medical research council (MC_UU_12015/1).

GoT2D consortium: National Institutes of Health ("Low-Pass Sequencing and High-Density SNP Genotyping for Type 2 Diabetes" RC2DK088389) and the German Center for Diabetes Research (DZD)

FUSION: Support for FUSION was provided by NIH grants R01-DK062370 (to M.B.), R01-DK072193 (to K.L.M.), and intramural project number 1Z01-HG000024 (to F.S.C.). Genome-wide genotyping was conducted by the Johns Hopkins University Genetic Resources Core Facility SNP Center at the Center for Inherited Disease Research (CIDR), with support from CIDR NIH contract no. N01-HG-65403.

EGCUT: EGCUT received financing from European Regional Development Fund, road-map grant no.3.2.0304.11-0312 and grant "Center of Excellence in Genomics (EXCEGEN)". EGCUT studies were covered also by targeted financing from the Estonian Government (IUT24---6, IUT20---60) and CTG grant (SP1GVARENG) from Development Fund of the University of Tartu.

DGI: The Botnia study (DGI) has been financially supported by grants from the Sigrid Juselius Foundation, Folkhälsan Research Foundation, Nordic Center of Excellence in Disease Genetics, an EU grant (EXGENESIS), Signe and Ane Gyllenberg Foundation, Swedish Cultural Foundation in Finland, Finnish Diabetes Research Foundation, Foundation for Life and Health in Finland, Finnish Medical Society, Paavo Nurmi Foundation, Helsinki University Central Hospital Research Foundation, Perklén Foundation, Ollqvist Foundation, Närpes Health Care Foundation and Ahokas Foundation. The study has also been supported by the Ministry of Education in Finland, Municipal Heath Care Center and Hospital in Jakobstad and Health Care Centers in Vasa, Närpes and Korsholm.

METSIM: The METSIM study was supported by the Academy of Finland (contract 124243), the Finnish Heart Foundation, the Finnish Diabetes Foundation, Tekes (contract 1510/31/06), and the Commission of the European Community (HEALTH-F2-2007 201681), and the US National Institutes of Health grants DK093757, DK072193, DK062370, and ZIA- HG000024.

PIVUS and ULSAM: These projects were supported by Knut and Alice Wallenberg Foundation (Wallenberg Academy Fellow), European Research Council (ERC Starting Grant), Swedish Diabetes Foundation (2013-024), Swedish Research Council (2012-1397, 2012-1727, and 2012-2215), Marianne and Marcus Wallenberg Foundation, County Council of Dalarna, Dalarna University, and Swedish Heart-Lung Foundation (20120197). The computations were performed on resources provided by SNIC through Uppsala Multidisciplinary Center for Advanced Computational Science (UPPMAX) under Project b2011036. Genotyping was funded by the Wellcome Trust under award WT064890. Analysis of genetic data was funded by the Wellcome Trust under awards WT098017 and WT090532. We thank the SNP&SEQ Technology Platform in Uppsala (www.genotyping.se) for excellent genotyping.

GenerationR: The general design of Generation R Study is made possible by financial support from the Erasmus Medical Center, Rotterdam, the Erasmus University Rotterdam, the Netherlands Organization for Health Research and Development (ZonMw), the Netherlands Organisation for Scientific Research (NWO), the Ministry of Health, Welfare and Sport and the Ministry of Youth and Families. Vincent W. Jaddoe received an additional grant from the Netherlands Organization for Health Research and Development (VIDI 016.136.361) and a European Research Council Consolidator Grant (ERC-2014-CoG-648916). Janine

F. Felix has received funding from the European Union's Horizon 2020 research and innovation programme under grant agreement No 633595 (DynaHEALTH). Additionally, we received funding from the European Union's Seventh Framework Programme (FP7/2007-2013), project EarlyNutrition under grant agreement n°289346 and an unrestricted grant from Danone Nutritia Research. These sponsors had no role in design and conduct of the study, analysis and interpretation of the data, and preparation, review or approval of the manuscript. The Generation R Study is conducted by the Erasmus Medical Center in close collaboration with the School of Law and Faculty of Social Sciences of the Erasmus University Rotterdam, the Municipal Health Service Rotterdam area, Rotterdam, the Rotterdam Homecare Foundation, Rotterdam and the Stichting Trombosedienst & Artsenlaboratorium Rijnmond (STAR-MDC), Rotterdam. We gratefully acknowledge the contribution of children and parents, general practitioners, hospitals, midwives and pharmacies in Rotterdam. The study protocol was approved by the Medical Ethical Committee of the Erasmus Medical Centre, Rotterdam. Written informed consent was obtained from all participants. The generation and management of GWAS genotype data was executed by the Human Genotyping Facility of the Genetic Laboratory of the Department of Internal Medicine, Erasmus MC, Rotterdam, The Netherlands. We thank Pascal Arp, Mila Jhamai, Marijn Verkerk, Lizbeth Herrera and Marjolein Peters, MSc, and Carolina Medina-Gomez, MSc, for their help in creating the GWAS database, and Karol Estrada, PhD and Carolina Medina-Gomez, MSc, for the creation and analysis of imputed data. This study makes use of sequence reference data generated by the UK10K Consortium, derived from samples from the ALSPAC and TwinsUK datasets. A full list of the investigators who contributed to the generation of the data is available from www.UK10K.org. Funding for UK10K was provided by the Wellcome Trust under award WT091310. We thank Jie Huang at the Wellcome Trust's Sanger Institute, at Hinxton, U.K. for the creation of imputed data, with the support of Marijn Verkerk, Carolina Medina-Gomez, MSc, and Anis Abuseiris and their input for the analysis setup. The Generation R Study received funding from the European Union's Horizon 2020 research and innovation programme (733206, LIFECYCLE).

Copenhagen: We are indebted to staff and participants of the Copenhagen General Population Study, Copenhagen City Heart Study, and Copenhagen Ischemic Heart Disease Study for their important contributions.

Sequenom genotyping: We would like to thank Suzannah Bumpstead, Sam Taylor and Michelle Dignam for coordinating and performing the Sequenom bespoke genotyping for Fenland samples.

This study makes use of data generated by the UK10K Consortium. The Wellcome Trust provided funding for UK10K (WT091310).

This research has been conducted using the UK Biobank Resource.

Supplemental Note

Cohort Descriptions

Cohorts contributing to the discovery phase: Whole-genome sequencing datasets

The Avon Longitudinal Study of Parents and Children (ALSPAC)
ALSPAC is a long-term health research project. More than 14,000 mothers enrolled during pregnancy in 1991 and 1992, and the health and development of their children has been followed in great detail ever since[1,2]. The ALSPAC families have provided a large amount of genetic and environmental information during the course of this longitudinal study. The study website contains details of all the data that is available through a fully searchable data dictionary. Ethical approval for the study was obtained from the ALSPAC Ethics and Law Committee and the Local Research Ethics Committees. Study participants were selected to maximise phenotypic coverage, previous genome-wide array genotyping, coverage with other "-omic" datasets (transcriptomic, metabolomic) and consent to whole genome sequencing, but were otherwise representative of the original population samples. For ALSPAC, the sequenced and imputed samples were combined for phenotype preparation.

The St Thomas' Twin Registry (TwinsUK)
The Department of Twin Research and Genetic Epidemiology (DTR) is the UK's only twin registry of 12,000 identical and non-identical twins between the ages of 16 and 85 years[3]. The database is used to study the genetic and environmental aetiology of age-related complex traits and diseases. Study participants were selected to maximise phenotypic coverage, previous genome-wide array genotyping, coverage with other "-omic" datasets (transcriptomic, metabolomic) and consent to whole genome sequencing, but were otherwise representative of the original population samples.
<u>ALSPAC and TwinsUK WGS data quality control:</u>
Of the 4,030 samples (1,990 TwinsUK and 2,040 ALSPAC) that were submitted for sequencing, 3,910 samples (1,934 TwinsUK and 1,976 ALSPAC) were sequenced and went through the variant calling procedure. Low quality samples were removed for any of the following reasons: high overall discordance to GWAS genotype data, high heterozygosity, no GWAS genotype data available, or sample below 4x mean read-depth. Overall, 3,798 samples (1,870 TwinsUK and 1,928 ALSPAC) were brought forward to the genotype refinement step. After the genotype refinement further samples were removed for any of the following reasons, post-refinement non-reference discordance with GWAS data, multiple relations to other samples, or discordance with manifest gender. This left a final set of 3,781 samples (1,854 TwinsUK and 1,927 ALSPAC). Details on production and quality control of ALSPAC and TwinsUK WGS are described in [4].
<u>TwinsUK anthropometric traits:</u>
Total body and regional fat mass was measured using a dual-energy x-ray absorptiometry (DXA) scanner (Hologic Discovery X-Ray Bone Densitometer; Hologic Model QDR-4500W). Participants were placed with light clothes and without metal objects in a recumbent position on the DXA table.
ALSPAC anthropometric traits:
A Lunar prodigy narrow fan beam densitometer was used to perform a whole body DXA scan where bone content, lean and fat masses are measured. The procedure was clearly explained to the child and parent and parental consent was obtained before proceeding. The child was asked to lie on the Prodigy couch (in light clothing without any metal fastenings), with the parent sitting at least a metre away to comply with the IRMER legislation. The child's height, weight, date of birth, gender and ethnicity (if appropriate) were entered into the computer and the machine was started. The arm of the machine moved over the child and two sources of X-ray scan the child. The child was reassured throughout the scan and encouraged to keep as still as possible. A daily QA was performed using the calibration block in accordance with the

manufacturers recommendations. The radiation protection supervisor or deputy scanned a spine phantom weekly.

FINRISK
The FINRISK study is a series of population-based cardiovascular risk factor surveys carried out every five years in five (or six in 2002) geographical areas of Finland, including North Karelia, Northern Savo (former Kuopio), Southwestern Finland, Oulu Province, Lapland province (in 2002 only) and the region of Helsinki and Vantaa[5]. A stratified random sample was drawn for each survey from the national population register; the age-range was 25-74 years. All individuals enrolled in the study received a physical examination, a self-administered questionnaire, and a blood sample was drawn. The Coordinating Ethical Committee of the Helsinki and Uusimaa Hospital District has approved the FINRISK surveys, which followed the declaration of Helsinki.
<u>Anthropometric traits:</u>
At the study sites, specially trained nurses measured weight, height, waist circumference, and hip circumference using standardized international protocols. All anthropometric measures were assessed with the participant in light clothing and with bare feet. The measurement of weight was rounded to the nearest 0.1 kg and height to the nearest 0.1 cm. BMI was calculated as the weight in kilograms divided by the squared height in meters (kg/m^2). Waist circumference was measured midway between the lower rib margin and iliac crest. Hip circumference was measured at the level of the widest circumference over the buttocks. The measurements of waist and hip circumferences were rounded to the nearest 0.5 cm. Waist to hip ratio was calculated as waist circumference divided by hip circumference.

Cohorts contributing to the discovery phase: GWAS imputed on the 1000 Genomes and UK10K haplotype panels

ALSPAC and TwinsUK GWAS
Additional GWAS data were used for each cohort. For ALSPAC, there were another 6,557 samples available, which were measured on Illumina HumanHap550 arrays 20. For TwinsUK, there were another 2,575 samples that were unrelated to the sequence dataset (IBS>0.125) with genotypes on Illumina HumanHap300 or Illumina Human610 arrays 21.
<u>ALSPAC and TwinsUK GWAS data quality control:</u>
Both datasets passed QC criteria (gender check, heterozygosity, European ancestry, relatedness (ALSPAC) and zygosity (TwinsUK). Variants discovered through WGS of the TwinsUK and ALSPAC cohorts were imputed into the full GWAS genotyped cohorts increasing the sample size for single point association analysis up to 9,132 subjects. The combined UK10K and 1000 Genomes Project reference panel and imputation of it into ALSPAC and TwinsUK GWAS arrays are discussed in [4,6].
<u>TwinsUK anthropometric traits:</u>
Total body and regional fat mass was measured using a dual-energy x-ray absorptiometry (DXA) scanner (Hologic Discovery X-Ray Bone Densitometer; Hologic Model QDR-4500W). Participants were placed with light clothes and without metal objects in a recumbent position on the DXA table.
<u>ALSPAC anthropometric traits:</u>
A Lunar prodigy narrow fan beam densitometer was used to perform a whole body DXA scan where bone content, lean and fat masses are measured. The procedure was clearly explained to the child and parent and parental consent was obtained before proceeding. The child was asked to lie on the Prodigy couch (in light clothing without any metal fastenings), with the parent sitting at least a metre away to comply with the IRMER legislation. The child's height, weight, date of birth, gender and ethnicity (if appropriate) were entered into the computer and the machine was started. The arm of the machine moved over the child and two sources of X-ray scan the child. The child was reassured throughout the scan and encouraged to keep as still as possible. A daily QA was performed using the calibration block in accordance with the manufacturers recommendations. The radiation protection supervisor or deputy scanned a spine phantom weekly.

United Kingdom Household Longitudinal Study (UKHLS)
The UKHLS, also known as Understanding Society is a longitudinal panel survey of 40,000 UK households (England, Scotland, Wales and Northern Ireland) representative of the UK population[7]. Participants are surveyed annually since 2009 and contribute information relating to their socioeconomic circumstances, attitudes, and behaviours via a computer assisted interview. The study includes phenotypical data for a representative sample of participants for a wide range of social and economic indicators as well as a biological sample collection encompassing biometric, physiological, biochemical, and haematological measurements and self-reported medical history and medication use. The UKHLS has been approved by the University of Essex Ethics Committee and informed consent was obtained from every participant.
UKHLS data quality control:
In total, 10,484 samples were genotyped on the Illumina HumanCoreExome chip (v1.0) at the Wellcome Trust Sanger Institute. Genotype calling was performed using GenCall and zCall. We excluded samples with a call rate <98% and <99% for Gencall and zCall respectively, or that were heterozygosity outliers, had sex discrepancies, were duplicates or that were ethnic outliers. Variants were excluded with a call rate below 95% and 99% for GenCall and zCall respectively, with a Hardy-Weinberg equilibrium *P-value* < 10^{-4} or with a cluster separation score < 0.4. Prior to phasing we compared the variants to the 1000 Genomes Project and the UK10K haplotypes and we excluded any variant for which the alleles differed for the same variant at the same position. In addition variants were excluded if they were a duplicate, monomorphic, a singleton or known to have poor clustering after inspecting the intensity data. Samples were phased using SHAPEITv2 and imputed using IMPUTE v2. Unrelated samples were determined by performing identity by descent using the autosomal directly genotyped variants with MAF≥1% and filtered so that variants with a linkage-disequilibrium r^2 <0.2 remained, in total 9175 unrelated samples were included in the analysis.

Rotterdam Study cohort I (RS-I)
The Rotterdam Study is an ongoing prospective population-based cohort study, focused on chronic disabling conditions of the elderly. The study comprises an outbred ethnically homogenous population of Dutch Caucasian origin. The rationale of the study has been described in detail elsewhere[8]. In summary, 7,983 men and women aged 55 years or older, living in Ommoord, a suburb of Rotterdam, the Netherlands, were invited to participate in the first phase. Fasting blood samples were taken during the participant's third visit to the research center.

Rotterdam Study cohort II (RS-II)
The Rotterdam Study cohort II prospective population-based cohort study comprises 3,011 residents aged 55 years and older from the same district of Rotterdam. The rationale and study design of this cohort is similar to that of the RS-I[8]. The baseline measurements took place during the first visit. The Rotterdam Study has been approved by the Medical Ethics Committee of the Erasmus MC and by the Ministry of Health Welfare and Sport of the Netherlands, implementing the "Wet Bevolkingsonderzoek: ERGO (Population Studies Act: Rotterdam Study)". All participants provided written informed consent to participate in the study and to obtain information from their treating physicians.

Rotterdam Study cohort III (RS-III)
The Rotterdam Study is an ongoing prospective population-based cohort study, focused on chronic disabling conditions of the elderly. The study comprises an outbred ethnically homogenous population of Dutch Caucasian origin. In 2006 all residents of Ommoord aged 45 years and over and who had not been invited before to RSI or RSII, were asked to participate following the same rationale that in these studies. A total of 3,932 out 6,057 of men and women entered the study. All participants provided written informed consent to participate in the study and to obtain information from their treating physicians.
Anthropometric traits for Rotterdam Study cohorts I-III:

For all participants dual-energy x-ray absorptiometry (DXA) based bone mineral density (BMD) measurements of the lumbar spine, dual hip and total body BMD, as well as determination of body composition parameters are assessed with a ProdigyTM total body fan-beam densitometer (GE Lunar Corp, Madison, WI, USA). From the total body scan, we measure lean mass and fat mass body composition, including total body, trunk, arm, legs, and android and gynoid regions of interest[9].

The Ludwigshafen Risk and Cardiovascular Health (LURIC) study - controls
The LURIC study is a prospective study of more than 3,300 individuals of German ancestry in whom cardiovascular and metabolic phenotypes (CAD, MI, dyslipidaemia, hypertension, metabolic syndrome and diabetes mellitus) have been defined or ruled out using standardised methodologies in all study participants. A 10-year clinical follow-up for total and cause specific mortality has been completed[10]. From 1997 to 2002 about 3,800 patients were recruited at the Heart Center of Ludwigshafen (Rhein). Inclusion criteria were: German ancestry, clinical stability (except for acute coronary syndromes) and existence of a coronary angiogram. Exclusion criteria were: any acute illness other than acute coronary syndromes, any chronic disease where non-cardiac disease predominated and a history of malignancy within the last five years. The study was approved by the ethics review committee at the Landesarztekammer Rheinland-Pfalz in Mainz, Germany, and written informed consent was obtained from the participants.

1958 Birth Cohort
Participants to the cohort have been followed-up regularly since birth with prospective information collected on a wide range of indicators related to health, health behaviour, lifestyle, growth and development. There have been 9 contacts with the participants since their birth (ages 7, 11, 16, 23, 33, 41, 45, 47, and 50 years). The biomedical survey at age 45 years included collection of blood samples and DNA from about 8000 participants. The survey was approved by the South East multicentre research ethics committee (MREC). There was an informed consent process conducted by the National Centre for Social Research[11].

TEENs of Attica: Genes and Environment (TEENAGE)
Participants were drawn from the TEENAGE study. A random sample of 857 adolescent students attending public secondary schools located in the wider Athens area of Attica in Greece were recruited in the study from 2008 to 2010. Our sample comprised 707 (55.9% females) adolescents of Greek origin aged 13.42 ± 0.88 years. Details of recruitment and data collection have been described elsewhere[12]. Prior to recruitment all study participants gave their verbal assent along with their parents'/guardians' written consent forms. The study was approved by Harokopio University Bioethics Committee and the Greek Ministry of Education, Lifelong Learning and Religious Affairs. DNA samples were genotyped using Illumina HumanOmniExpress BeadChips (Illumina, San Diego, CA, USA) at the Wellcome Trust Sanger Institute, Hinxton, UK. Genotyping and data quality control have been described previously[12].

HELIC MANOLIS
The HELIC (Hellenic Isolated Cohorts) MANOLIS (Minoan Isolates) collection focuses on Anogia and surrounding Mylopotamos villages. Recruitment of this population-based sample was primarily carried out at the village medical centres. All individuals were older than 17 years and had to have at least one parent from the Mylopotamos area. The study includes biological sample collection for DNA extraction and lab-based blood measurements, and interview-based questionnaire filling. The phenotypes collected include anthropometric and biometric measurements, clinical evaluation data, biochemical and haematological profiles, self-reported medical history, demographic, socioeconomic and lifestyle information. The study was approved by the Harokopio University Bioethics Committee and informed consent was obtained from every participant.

HELIC Pomak

The HELIC (Hellenic Isolated Cohorts) Pomak collection focuses on the Pomak villages, a set of isolated mountainous villages in the North of Greece. Recruitment of this population-based sample was primarily carried out at the village medical centres. The study includes biological sample collection for DNA extraction and lab-based blood measurements, and interview-based questionnaire filling. The phenotypes collected include anthropometric and biometric measurements, clinical evaluation data, biochemical and haematological profiles, self-reported medical history, demographic, socioeconomic and lifestyle information. The study was approved by the Harokopio University Bioethics Committee and informed consent was obtained from every participant.

HELIC MANOLIS and HELIC Pomak data quality control:

The HELIC samples were genotyped on both the Illumina HumanOmniExpress and Illumina HumanExome chip at the Wellcome Trust Sanger Institute. For the genotype calling we used Illuminus for OmniExpress and GenCall followed by zCall for the exome chip. We excluded samples with sex discrepancies, that were duplicates or ethnic outliers, that were heterozygosity outliers or that had a call rate <98% for OmniExpress and call rate <98% and <99% for Exome chip for GenCall and zCall respectively. We excluded variants with call rate <95%, if they had a MAF≥5%, and <99%, if they had a MAF<5% for OmniExpress and call rate <95% and <99% for Exome chip for GenCall and zCall respectively or that had a Hardy-Weinberg equilibrium *P-value* $<10^{-4}$. We also excluded variants with a cluster separation score <0.4 for the Exome chip. We combined the genotypes for the OmniExpress and Exome chip into a single dataset. If a variant was present in both the OmniExpress and Exome array then the genotypes for those variants with MAF≥5% were taken from the OmniExpress whilst those with MAF <5% were taken from the Exome chip. Prior to phasing we compared the variants to the 1000 Genomes Project data and the UK10K haplotypes and we excluded any variant for which the alleles differed for the same variant at the same position. Variants were also excluded if they had MAF <5% and were genotyped on the OmniExpress, were monomorphic, a duplicate, a singleton or that were known to have poor clustering after inspecting the intensity data. We phased using SHAPEITv2 and imputed using IMPUTE v2.

INGI-Val Borbera (INGI-VB)

The INGI-Val Borbera population is a collection of 1,785 genotyped samples collected in the Val Borbera Valley, a geographically isolated valley located within the Appennine Mountains in Northwest Italy[13]. The valley is inhabited by about 3,000 descendants from the original population, living in 7 villages along the valley and in the mountains. Participants were healthy people 18-102 years of age that had at least one grandfather living in the valley. A standard battery of tests was performed by the laboratory of ASL 22 - Novi Ligure (AL), on sera from fasting blood collected in the morning. The project was approved by the Ethical committee of the San Raffaele Hospital and of the Piemonte Region. All participants signed an informed consent.

INGI-Friuli Venezia Giulia (INGI-FVG)

The Friuli Venezia Giulia population represents a collection of six villages covering a total area of 7858 km^2 in a hilly part of Friuli-Venezia Giulia (FVG) county located in north-eastern Italy. A recent study[14] characterized this population as a genetic isolate with high level of genomic homozygosity and elevated linkage disequilibrium. The cohort accounts for 1,590 genotyped samples. Participants were randomly selected people 3-92 years of age. Genotyping and phenotyipic data for 1,590 samples are available. People with age <18 were excluded from analyses. A written informed consent for participation was obtained from all subjects. The project was approved by the Ethical committee of the IRCCS Burlo-Garofolo.

INGI-Carlantino (INGI-Carl)

Carlantino is a small village in the Province of Foggia in southern Italy. Genetic analyses of chromosome Y haplotypes as well as mitochondrial DNA show that Carlantino is a genetically homogeneous population and not only a geographically isolated village[14]. Participant were randomly selected in a range of 15 – 90 years of age. Genotyping and phenotypic data are available for 630 individuals. People with age <18 were

excluded from analyses. Subjects gave their written informed consent for participating in these studies. The project was approved by the local administration of Carlantino, the Health Service of Foggia Province, Italy, and ethical committee of the IRCCS Burlo-Garofolo of Trieste.

Arthritis Research UK Osteoarthritis Genetics (arcOGEN)
arcOGEN is a collection of unrelated, UK-based individuals of European ancestry with knee and/or hip osteoarthritis (OA) from the arcOGEN Consortium[15,16]. Cases were ascertained based on clinical evidence of disease to a level requiring joint replacement or radiographic evidence of disease (Kellgren–Lawrence grade ≥2). The arcOGEN study was ethically approved, and all subjects used in this study provided written, informed consent.

INCIPE
For the INCIPE study, 6200 randomly chosen individuals, all of European descent and at least 40 years of age as of 1 January 2006, received a letter inviting them to participate in the study. A total of 3870 subjects (62%) accepted and were enrolled. The ethics committees of the involved institutions approved the study protocol. Two studies were included in the analysis:

1. INCIPE1: Individuals genotyped on HumanOmniExpress-12v1-Multi_B
2. INCIPE2: Individuals genotyped on HumanCoreExome-12v1

London Life Sciences Prospective Population Study (LOLIPOP)
LOLIPOP is an ongoing community prospective cohort of 17,606 Indian Asian and 7,766 European men and women aged 35-75 years, recruited in West London, UK, to study the environmental and genetic factors that contribute to cardiovascular disease among UK Indian Asians[17,18]. Indian Asian participants reported having all four grandparents born on the Indian subcontinent, while European participants are self-classified whites born in Europe. For the current study, only white individuals were included in the primary meta-analysis. All participants provided written consent including for genetic studies. The LOLIPOP study is approved by the local Research Ethics Committees.
Three studies were included in the analysis:
1. LOLIPOP_EW_A: European whites from the general population, genotyped on Affymetrix 500K arrays.
2. LOLIPOP_EW_P: European whites from the general population, enriched by subjects with metabolic syndrome, genotyped on Perlegen custom array.
3. LOLIPOP_EW610: European whites from the general population, genotyped on Illumnia Human610 array.

Cohorts contributing to the follow-up effort: *In silico* follow-up

SardiNIA
The SardiNIA study is a longitudinal population-based cohort study started in 2001 to study quantitative traits of biomedical relevance with a special emphasis on those influencing aging. In a first survey, the project recruited individuals from four towns in the Lanusei Valley (east-central Sardinia) and assessed 98 quantitative traits including over 62% of the eligible population living in the region (age 14-102 years), and at least 96% of the initial cohort have all grandparents born in the same province. The initial group of 6,148 individuals included 4,933 phenotyped sib pairs, 4,266 phenotyped parent-child pairs, >4,069 phenotyped cousin pairs, and >6,459 phenotyped avuncular pairs. Recently, the study recruited 773 additional individuals, involving a total of 6,921 subjects. The longitudinal study, now in its 14th year and in its fourth phase, collected the longitudinal information on more than 1000 quantitative traits, including inflammatory markers and immuno-related traits, that can be scored on a continuous scale[19,20]. A written informed consent was obtained from all participants.
Quality control:
Samples having sex discordance or with call rate lower than 98% were removed from the analyses.

SNPs with call rate lower than 98%, HWE *P-value* $<10^{-6}$, at least 1 mendelian errors in more than 1% of the available families, monomorphic and with more than 1 discordance in 13 twin pairs were removed from the analyses.

GenerationR (GenR)

This study was embedded in the Generation R Study, a population-based prospective cohort study from fetal life onwards in Rotterdam, the Netherlands. The Medical Ethics Committee of the Erasmus MC, University Medical Center, Rotterdam, has approved the study and written informed consent was obtained for all participants. All children were born between April 2002 and January 2006. Enrollment was aimed at early pregnancy, but was allowed until birth of the child. In total, 9,778 mothers and their children were included in the study. Details of recruitment and data collection have been described in detail elsewhere[21,22]. The current analysis includes those children with genome-wide scan data that had a DXA scan around the age of six years.

<u>Anthropometric traits:</u>

Total body and regional fat mass was measured using a dual-energy x-ray absorptiometry (DXA) scanner (iDXA, 2008; GE-Lunar) and analyzed with the enCORE software, version 12.6 (GE-Healthcare). DXA can accurately detect whole-body fat mass within less than 0.25% coefficient of variation. Children were placed without shoes, heavy clothing, and metal objects in supine position on the DXA table. Total fat mass (kilograms) was calculated as a percentage of total body weight (kilograms) measured by DXA. The fat mass index (body fat mass/height^2), and lean mass index (body lean mass/height^2) calculated[23].

<u>Quality Control:</u>

Cord blood for DNA isolation was available in 58% of all live-born participating children. Sex-mismatch rate between genome based sex and midwife-record based sex was low (<0.5%), indicating that possible contamination of maternal DNA was extremely low. Missing cord blood samples were mainly due to logistical constraints at the delivery. GWAS scans were run using the Illumina 610 Quad and 660 platforms. IMPUTE2 software was used to impute genotypes to the combined UK10K-1000 genomes panel. Before imputation, SNPs were excluded if they had high levels of missing data (SNP call rate <98%), strong departures from Hardy-Weinberg equilibrium (*P-value* $<1\times10^{-6}$), or low MAF (<0.1%).

UK Biobank

500,000 participants aged 40-69 years were recruited between 2006 and 2010 in 22 assessment centres throughout the UK[24]. The assessment visit included electronic signed consent; a self-completed touch-screen questionnaire; brief computer-assisted interview; physical and functional measures; and collection of biological samples and genetic data.

<u>Anthropometric traits:</u>

BMI was calculated (kg/m^2) using measured height and weight. Weight (kg) was measured using the Tanita BC-418 MA body composition analyser (accurate to within 0.1kg) after removal of heavy clothing and shoes. Standing height (cm) was measured without shoes using a Seca 202 height measure. Waist circumference (cm) at the level of the umbilicus and hip circumference was measured using a Wessex non-stretchable sprung tape measure.

<u>Data Quality Control:</u>

All the analyses were carried out in Europeans. Subjects with high heterozygosity, low call rate, related participants, and pregnant women were further excluded from analyses. SNP genotypes were called by Affymetrix, and any SNPs failed by Affymetrix batch-specific QC thresholds were set to missing in all subjects from that batch. Additional SNP QC steps were carried out by the UK Biobank team, in which SNPs at certain batch/plates were set to missing if their genotype distributions were significantly different from other batches/plates (*P-value* $<10^{-12}$), or there were significant deviations of genotype frequencies from those expected under Hardy-Weinberg equilibrium (*P-value* $<10^{-12}$). Imputation was carried out, with the combined reference panel of 1000 Genomes phase 3 and UK10K data. Any variants imputed, with minor allele frequency of <0.001% were filtered. Association results from SNPs with imputation quality score <0.3 were discarded.

Cohorts contributing to GoT2D:

WGS

Diabetes Genetics Initiative (DGI)
Details of the samples have been described elsewhere[25]. For sequencing, we selected individuals from the phenotypic extremes using T2D liability scores calculated based on disease status, age, BMI, and sex[26]. We chose individuals from the studies used for the DGI GWAS, the Scania Diabetes Registry[27], and the Malmö Preventive Project[28]. We prioritized early-onset cases with low BMI and older controls with high BMI; we excluded cases with age of diagnosis<35 years to minimize inclusion of individuals with type 1 diabetes (T1D).

Finland-United States Investigation of NIDDM Genetics (FUSION)
Details of the samples have been described elsewhere[29]. For sequencing, we chose T2D cases from FUSION families with ≥2 first-degree relatives with T2D and selected one individual per family with either available GWAS data or earliest age at diagnosis. Remaining cases were chosen from the FUSION replication set, selecting those with earlier age at diagnosis from among those with Metabochip data and age at diagnosis≥35 years. Unrelated normal glucose tolerant (NGT) controls with age≤80 years were frequency matched to the cases by birth province and, within each birth province, controls with the highest age (in years) + 2×BMI (in kg/m^2) were prioritized. All selected individuals had BMI≥18.5 kg/m^2.

Cooperative Health Research in the Region of Augsburg (KORA)
Details of the samples have been described elsewhere[30-32]. For sequencing, we prioritized cases with ≥1 first-degree relative with T2D (self-reported). We then chose individuals with BMI≤30 kg/m^2 and age of diagnosis<65 years, or BMI≤33 kg/m^2 and age of diagnosis≤60 years. We selected controls from KORA F4 who were either >60 years of age with BMI>32 kg/m^2, or >65 years of age with BMI>31 kg/m^2.

United Kingdom T2D Genetics consortium (UKT2D)
Details of the samples have been described elsewhere[33-35]. For sequencing, we chose cases from the Wellcome Trust Case Control Consortium (WTCCC) and controls from the TwinsUK study. For cases, we excluded females with age of diagnosis≥66 years or BMI≥32 kg/m^2, and males with age of diagnosis≥62 years or BMI≥31 kg/m^2. We ranked the remaining samples by age and BMI; we multiplied the two ranks and selected individuals with the lowest product of ranks. For controls, we considered twin pairs (a) with no recorded family history of diabetes; (b) with neither twin ever recorded as impaired glucose tolerant (fasting glucose [FG]>6.1mmol/l); and (c) who had available quantitative trait and GWAS data and no evidence of admixture in analysis of the GWAS data. From qualifying twin pairs, we chose the twin with the lowest ratio of FG level to BMI across all readings, giving priority to unrelated individuals with the lowest FG/[BMI×age] ratio. We performed pairwise sample matching between cases and possible controls using the first two principal components from an analysis of previously available genome-wide genotyping data, with the best control for each case selected.

Imputed GWAS

Diabetes Genetics Initiative (DGI)
Details of the samples and GWAS have been described elsewhere[25]. The current analysis included 899 T2D cases and 1,057 NGT controls from Sweden or Finland. The Finnish samples were predominantly from the Botnia region of Finland and the Swedish samples from Southern Sweden and Skara. T2D cases from both countries met WHO 1999 criteria with FG≥7.0 mmol/l or 2-hour glucose≥11.1 mmol/l during an oral glucose tolerance test. Cases had age of diagnosis >35 years and no detectable anti-GAD antibodies (defined as anti-GAD antibody levels <32 IU/ml in the Finnish samples and <1.3 anti-GAD relative units in

the Swedish). Controls had no first-degree relatives with T2D. Cases and controls were matched on age (within 5 years), sex, BMI, and geographic region. Samples were genotyped using the Affymetrix Human Mapping 500K array.

Estonian Genome Center of the University of Tartu (EGCUT)
Details of the samples have been described elsewhere[36]. The current analysis included 469 T2D cases and 7,781 population-based controls from the Estonian Biobank cohort, a volunteer-based sample of the Estonian resident adult population aged ≥18 years. T2D diagnosis was based on standardized health examination together with questionnaires on health-related topics as described in WHO ICD-10. Data are regularly updated through linkage to national databases and registries. Controls represent a random subset of the Estonian population. Participants were genotyped with either the Illumina HumanHap 370K array (EGCUT-370K, 80 cases and 1,768 controls) or the Illumina OmniExpress array (EGCUT-OMNI, 389 cases and 6,013 controls). GWAS analysis was performed separately in the two subsets.

Finland-United States Investigation of NIDDM Genetics (FUSION)
Details of the samples and GWAS have been described elsewhere[29]. The GWAS sample for imputation included 1,060 T2D cases and 1,090 NGT controls of Finnish origin. 688 T2D cases were selected one per family from T2D affected sibling pairs; 372 were from the population-based Finrisk 2002 study. NGT controls included 272 spouses of FUSION study subjects, 188 individuals who were NGT at ages 65 and 70, and individuals from Finrisk 2002. Cases were defined by WHO 1999 criteria of FG≥7.0 mmol/l or 2-hour plasma glucose≥11.1 mmol/l, by reported diabetes medication use, or based on medical record review. FUSION cases were excluded if they had known or probable T1D among first-degree relatives. Controls were NGT as defined by WHO 1999 criteria. Cases and controls were approximately frequency matched by 5-year age category, sex, and birth province. Samples were genotyped using the Illumina HumanHap300 array.

METabolic Syndrome In Men (METSIM)
The cross-sectional METSIM Study includes 10,197 men, aged from 45 to 73 years, randomly selected from the population register of the Kuopio town, Eastern Finland, and examined in 2005-2010. The aim of the study is to investigate genetic and non-genetic factors associated with the risk of type 2 diabetes (T2D), cardiovascular disease (CVD), and insulin resistance –related traits in a cross-sectional and longitudinal setting[37,38]. Study protocol includes e.g. collection of data on CVD risk factors (smoking, exercise, diet, history of chronic diseases including coronary heart disease, stroke, cardiac failure, medication, history of diabetes or early onset coronary heart disease in the family), questionnaire on the FINDISC Score, measurement of height, weight, waist circumference, hip circumference, blood pressure (3 times), and bioimpedance for the evaluation of fat percentage.

Prospective Investigation of the Vasculature in Uppsala Seniors (PIVUS)
Details of the samples and GWAS have been described elsewhere[39,40]. Participants were randomly sampled from all men and women aged 70 years living in Uppsala County in 2001. This analysis included 111 T2D cases and 838 non-T2D controls of Swedish descent. T2D status was defined by fasting blood glucose>6.1 mmol/l or known diabetes. Controls were non-T2D individuals. All samples were genotyped with the Illumina Metabochip and Illumina OmniExpress array.

Uppsala Longitudinal Study of Adult Men (ULSAM)
All men born between 1920 and 1924 in Uppsala, Sweden were invited to participate at age 50 years in this longitudinal cohort study that was started in 1970. Participants were reinvestigated at ages 60, 70, 77, 82, and 88 years[41]. Our analysis included 166 T2D cases and 953 non-T2D controls of Swedish descent. T2D status was defined as hospital discharge register-defined diabetes before 2002. Controls were non-T2D individuals. All samples were genotyped with the Illumina Metabochip and Illumina HumanOmni2.5 array.

Cohorts contributing to the follow-up effort: *De novo* follow-up

Fenland

The Fenland Study is an ongoing, population-based cohort study (started in 2005) designed to investigate the association between genetic and lifestyle environmental factors and the risk of obesity, insulin sensitivity, hyperglycemia and related metabolic traits in men and women aged 30 to 55 years[42]. Potential volunteers were recruited from General Practice sampling frames in the Fenland, Ely and Cambridge areas of the Cambridgeshire Primary Care Trust in the UK. Exclusion criteria for the study were: prevalent diabetes, pregnant and lactating women, inability to participate due to terminal illness, psychotic illness, or inability to walk unaided. All participants had measurements done at the MRC Epidemiology Unit Clinical Research Facilities in Ely, Wisbech and Cambridge. Participants attended after an overnight fast for a detailed clinical examination, and blood samples were collected. The Local Research Ethics Committee granted ethical approval for the study and all participants gave written informed consent.

<u>Sequenom genotyping:</u>

Genotyping was performed using the iPLEX® Assay and the MassARRAY® System (Agena Bioscience, Inc.). Assays for all SNPs were designed using the eXTEND suite and MassARRAY Assay Design software version 4.0.0.2 (Agena Bioscience, Inc.). Amplification was performed in a total volume of 5µL containing ~10ng genomic DNA, 100nM of each PCR primer, 500µM of each dNTP, 1.25 x PCR buffer (Qiagen), 1.625mM $MgCl_2$ and 1U HotStar Taq® (Qiagen). Reactions were heated to 94 °C for 15 min followed by 45 cycles at 94°C for 20 s, 56°C for 30 s and 72°C for 1 min, then a final extension at 72°C for 3 min. Unincorporated dNTPs were SAP digested prior to iPLEX™ allele specific extension with mass-modified ddNTPs using an iPLEX reagent kit (Agena Bioscience, Inc.). SAP digestion and extension were performed according to the manufacturer's instructions with reaction extension primer concentrations adjusted to between 0.7-1.8µM, dependent upon primer mass. Extension products were desalted and dispensed onto a SpectroCHIP using a MassARRAY Nanodispenser prior to MALDI-TOF analysis with a MassARRAY Analyzer Compact mass spectrometer. Genotypes were automatically assigned and manually confirmed using MassARRAY TyperAnalyzer software version 4.0 (Agena Bioscience, Inc.).

<u>Sequenom Data Quality Control:</u>

Samples were removed if their call rate was <80%. SNPs were removed if their call rate was <80%, HWE *P-value* $<10^{-4}$ and if the gender in the manifest was discordant with the gender in the Sequenom iPLEX assay.

<u>Anthropometric traits:</u>

Body composition measurements of fat mass were measured by total body DXA scans (GE Lunar Prodigy Advanced, GE Medical Systems, Hatfield, UK). Results were acquired and analysed within the enCORE software (Version 10.51.006 to 16, GE Medical Systems) under basic analysis settings. Participants were positioned according to the total body measurement and analysis protocol recommended by the manufacture. Participants body composition results were recalculated by the symmetry and ½ body method when appropriate (replacing omitted left arm or body with right arm or body data). Volunteers were excluded if pregnancy could not be ruled out and if weight exceeded 136kg.

Copenhagen General Population Study (CGPS)

This general population study was initiated in 2003 with ongoing enrolment[43,44]. BMI and ischemic heart disease endpoints have been collected from 1976 to May 2009. Individuals were selected on the basis of the national Danish Civil Registration System to reflect the adult Danish population aged 20–100 y. All participants were white and of Danish descent; this information is available through the national Danish Central Person Registry. Data were obtained from a questionnaire, a physical examination, blood samples, and from DNA. At the time of genotyping 59,883 participants had been included; of these, 5,270 were used as controls in the Copenhagen Ischemic Heart Disease Study, leaving 54,613 for analyses in the CGPS. The study was approved by Danish ethical committees and Herlev Hospital.

Dataset used for mQTL analyses

ARIES Data

The Accessible Resource for Integrative Epigenomic Studies (ARIES) dataset represents genome-wide DNA methylation levels on ALSPAC samples selected from 1,018 mother-child pairs at three time points in children and two time points in their mothers[45]. A DNA sample was extracted from cord blood drawn from the umbilical cord upon delivery or peripheral blood according to standard procedures. Written informed consent has been obtained from all ALSPAC participants. Ethical approval for the study was obtained from the ALSPAC Ethics and Law Committee and the Local Research Ethics Committees. Samples were bisulfite converted using the Zymo EZ DNA Methylation™ kit and genome-wide methylation was measured using the Illumina HumanMethylation450 BeadChip. Methylation data were normalized in R with the wateRmelon package[46] using the Touleimat and Tost[47] algorithm to reduce the non-biological differences between probes. Data were then rank-normalized to remove outliers, and regressed on all covariates, plus bisulphite-converted DNA plate batch to remove potential batch effects (with missing values set to probe mean). Children were genotyped using the Illumina HumanHap550 quad genome-wide SNP genotyping platform by the Wellcome Trust Sanger Institute and the Laboratory Corporation of America. Mothers were genotyped using the Illumina human660W-quad genome-wide SNP genotyping platform at the Centre National de Génotypage. Genotypes were phased together using SHAPEIT, and then imputed against the 1000 Genomes reference panel (phase 1 version 3) using Impute. The final imputed dataset contained 8,074,398 SNPs keeping SNPs that have Hardy-Weinberg equilibrium *P-value* $>5\times10^{-7}$, MAF>1% and imputation quality score >0.8. Each SNP in the imputed datasets was analysed against all CpG sites in the Illumina HM450 with the exception of those failing QC, and those reported to map to more than one location (N 19,834) or to contain a genetic variant at the CpG site (N 74,182)[48]. Association analysis of SNPs with CpG sites was performed using an additive model (rank-normalized CpG methylation on SNP allele count) using Matrix eQTL[49]. SNP effects from this analysis that were *P-value* $<10^{-7}$ were then taken forward for re-analysis in PLINK to perform exact linear regression including covariates. Covariates included in all analyses were age (excluding birth), sex (children only), the top 10 ancestry principal components, bisulfite conversion batch and estimated white blood cell counts (using an algorithm based on differential methylation between cell types[50]).

Annotations of newly reported variants

Variants associated with height:

rs61734601 (stage 1 and 2 weighted effect allele frequency [WEAF] 8.2%, beta=-0.113, *P-value*=1.38×10^{-101}) is 359kb away from the physically closest positive control variant (Table S14). It is located in the intron of *PPP1CA* and a non-coding exon of *CARNS1*, but is reported as significantly associated with expression of *RAD9A,* a DNA repair gene 20kb downstream, in several different tissues in the GTEx[51] portal. DNA repair genes have previously been linked to growth disorders[52]. rs61734601 is in high LD (r^2=0.82) with rs553917782, a 6-nucleotide insertion 10bp upstream of *RAD9A*. The 8 following nucleotides are conserved (mean GERP[53] score 2.2) and occur near the centre of a DNase hypersensitivity peak that coincides with nucleosome depletion in multiple tissues from the Roadmap Epigenomics[54] project, indicating likely transcription factor binding (Figure S15).

rs41271299 (WEAF 5.5%, beta=0.123, *P-value*=1.90×10^{-71}) resides in the intron of *ID4*, in a highly conserved region (the flanking 20 bases are completely conserved in a 17-way mammalian alignment[55] and the GERP score[53] at the variant site is 5.8) (Table S16). The variant resides 23bp from the final acceptor splice site in the gene, therefore potentially disrupting splicing, and the region is annotated as open chromatin in diverse tissues[56] (Figure S15).

rs114976626 (WEAF 2.7%, beta= -0.096, *P-value*=5.00×10^{-20}) is a missense variant in *SSC5D*. *SSC5D* and its secreted protein product are poorly characterized. rs114976626 causes a conservative alanine to valine

mutation in a linker sequence connecting two scavenger receptor cysteine-rich (SRCR) domains[57]. The expression of *SSC5D* can be detected in many tissues[51], and the presence of the protein product throughout the entire body[58]. Proteins belonging to the scavenger receptor family are involved in the innate immune response[59].

rs6930571 (WEAF 17.9%, beta=0.038, *P-value*=6.01x10^{-18}) is a regulatory region variant that overlaps with a CTCF binding site, and has been identified as eQTL for 13 genes in 26 tissues[51]. rs6930571 is associated with the expression of *RNF5*[51], a E3 ubiquitine ligase gene located 231kb downstream. Animal models show that the mutation of this gene causes abnormal muscle regeneration[60]. Aberrant expression of *RNF5* is observed in various human myopathies[60]. rs6930571 is also associated with the expression of *CYP21A2*[51]. The protein product of this gene catalyzes the 21-hydroxylation of steroids, involved in adrenal synthesis of mineralocorticoids and glucocorticoids[61]. Abnormalities of this gene cause congenital adrenal hyperplasia, a common recessive disease due to defective synthesis of cortisol, characterized by androgen excess leading to ambiguous genitalia in affected females, rapid somatic growth during childhood in both sexes with premature closure of the epiphyses and short adult stature. The minor allele of rs6930571 is associated with lower plasma cortisol in the CORNET GWAS meta-analysis[62], although the association is not significant (EAF=18%, beta= -0.023, standard error=0.016, *P-value*=0.168, sample size 12,592).

rs202238847 (WEAF 2.23%, beta=0.095, height *P-value*=3.76x10^{-17}) causes a single base pair deletion in an intron of *CCDC36*, which is expressed in skin/skeletal muscle and testis/ovary in the fetal and adult body, respectively[51]. *LAMB2* is located 93kb upstream of rs202238847 and is involved in growth retardation and decreased body weight in mice[63].

rs4360494 (WEAF 44.7%, beta=0.024, *P-value*=8.98x10^{-13}) is captured by rs4072980 (r^2=0.84) in [64] with *P-value*=3.1x10^{-6}. rs4360494 has an effect on the expression on multiple genes[51]: *FHL3, SF3A3, INPP5B, RP11-109P14.10, UTP11L, MTF1*. The cytoskeleton associated protein product of *FHL3* plays an important role in myogenesis through its binding partner *MyoD*[65]. Overexpression of this gene in mouse myoblast cells results in the retarded myotube formation and decreases the expression of muscle-specific regulatory genes such as myogenin[65]. *INPP5B* is a protein coding gene 43kb upstream involved in PI3K signaling pathway. *INPP5B* might play a role in Lowe's syndrome which is characterized by short stature[66]. *MTF* ,located 130kb upstream from rs4360494, has been associated with hypothyroidism[67], often characterized by slow growth rate and its protein product activates metal response genes.

rs13059073 (WEAF 45.5%, beta=0.022, *P-value*=3.23x10^{-11}) is captured by rs1047898 (r^2=0.98) in [64] with *P-value*=4.6x10^{-6}. rs13059073 is an intergenic variant located 7kb downstream of *WNT5A*, whose secreted product (Wnt-5a) is the primary ligand in the non-canonical Wnt signalling pathway, and a regulator of chondrogenesis[68]. Mutations within this gene were shown to cause the autosomal dominant Robinow syndrome, which is characterized by skeletal dysplasia, limb shortening and other abnormalities[69]. Shortened body length, and various skeletal abnormalities were also described in animal models[70]. Members from the non-canonical Wnt signalling pathway have already implicated in the determination of height (eg. *ROR2*). rs17711489 is associated with the expression of *WNT5A*[51] and is in LD with our signal (r^2=0.25).

rs4303473 (WEAF 38.0%, beta=0.022, *P-value*=4.08x10^{-11}) is an intronic variant in *CRISPLD2*, which is involved in the assembly of the extracellular matrix and has been linked with abnormal embryo size in mice[71].

rs16888802 (WEAF 17.6%, beta=0.028, *P-value*=5.49x10^{-11}) is located 4kb downstream of *NKX3-2*, which encodes a transcription factor with an important role in development and chondrocyte regulation[72]. Rare

frameshift mutations of this gene are observed in spondylo-megaepiphyseal-metaphyseal dysplasia, a skeletal dysplasia characterized by disproportionate short stature [73].

rs183677281 (WEAF 2.4%, beta= 0.071, *P-value*=1.24x10^{-10}) is an intron of the principal[74] transcript of *TGFB2* and a promoter flanking region (ENSR00001598375) active in skeletal muscle myotubes, umbilical vein endothelial cells, astrocytes, fibroblasts[75]. The protein coded by *TGFB2* is a transforming growth factor involved in various developmental processes[76]. Animal models of this gene show diverse phenotypes including defects of musculoskeletal system and morphology[76]. *TGFB2* has already been linked to height by several studies[64,77,78] but the reported associations are independent of our signal (Table S14).

rs1848053 (WEAF 24.8%, beta= -0.024, *P-value*=2.00x10^{-10}) is associated with the expression of *FBN1*[51], which is involved in a series of developmental disorders affecting the musculoskeletal system[79-81].

rs62038850 (WEAF 2.7%, beta= 0.071, *P-value*=2.45x10^{-10}) overlaps with the 3' untranslated region of the principal transcript of the ubiquitously expressed gene *PGP*. Phosphoglycolate phosphatase, the protein product of this gene, regulates the cellular levels of glycerol-3-phosphate a metabolic intermediate of glucose, lipid and energy metabolism[57]. rs62038850 is 1kb away from *BRICD5*, whose integral membrane protein product is mainly found in prostate, pancreas, salivary gland, gastric chief cells, glandular cells in cervix and endometrium[82]. rs62038850 is also 3kb away from *MLST8*, whose protein product is part of the mTOR complex, therefore involved in the regulation of cell growth and survival[57]. The highest levels of the broadly expressed protein product can be detected in skeletal muscle, heart and kidney[83]. Animal models of *MLST8* show growth/body-size phenotypes including embryotic growth retardation and decreased embryo size[84]. *CASKIN1* is located 16kbp away coding a scaffolding protein and is expressed mainly in brain[58]. *MLST8* and *CASKIN1* have already been associated with height[77], but the reported signals are independent from rs62038850 (Table S14). *E4F1* is a ubiquitously expressed protein coding gene 10kb away from rs62038850, the protein product of this gene is a transcriptional repressor regulating cell proliferation and survival[57]. Animal models show mutation of *E4F1* can cause decreased embryo size[85].

rs142854193 (WEAF 2.3%, beta=0.071, *P-value*=1.31x10^{-9}) is a novel height variant overlapping with the 3' untranslated region of the principal[74] transcript of the protein coding gene *FKBP9* and an intron of protein coding gene *AVL9*. *AVL9* is a poorly characterized gene, its protein product is a single pass membrane protein potentially involved in cell migration, endosome trafficking[86]. Misregulation of the expression of this protein causes secretory defects in yeast[86]. *FKBP9* is a chaperone: it mediates the isomerization of peptide bonds during protein synthesis[57]. Mutations of *FKBP9* in mice causes behavioral abnormalities[87] its protein product is expressed throughout the entire body[58].

rs2808290 (WEAF 50%, beta=0.0198, *P-value*=1.34x10^{-9}) is located in an intergenic region, but overlaps with a regulatory feature (ENSR00001421990), a predicted enhancer, which is predicted to be active in osteoblast, myoblast and fibroblast cell lines[75]. The closest gene is protein coding *MKX* located 60kb downstream. The protein product of *MKX* is a transcription factor and regulates collagen expression and tendon development. Animal models of this gene show abnormal tendon and tail morphology[88].

rs116878242 (WEAF 7.5%, beta=0.033, *P-value*=3.14x10^{-8}) resides in an intergenic region flanked by several non-coding genes. rs116878242 overlaps with an annotated promoter flanking region (ENSR00001537939) shown to be active in various cell types including fibroblasts[89]. The nearest protein-coding gene is *SOX9* over 100kb upstream, which is involved in sex determination, and is associated with height (intergenic rs10083886[64], r^2=0.061 with rs116878242, Table S14). *SOX9* is also implicated in various monogenic diseases (Table S25) including campomyelic dysplasia, which includes a skeletal development phenotype[90]. 400bp away from rs116878242, there is a NF-κB transcription binding site, which has been shown to affect the expression of *SOX9*[91].

Variants associated with BMI:

rs62107261 (WEAF 4.7%, beta=-0.075, *P-value*=1.27×10^{-27}) resides in the exon of a long intergenic non-coding RNA (*AC105393.2*). The closest protein-coding gene is *TMEM18*, over 200kb upstream, which has previously been associated with BMI and obesity (Table S14).

rs2003476 (WEAF 40.4%, beta= -0.025, *P-value*=5.89×10^{-13}) resides in an intron of the transcription factor-coding gene *CRTC1*. *Crtc1*-null mice are hyperphagic, obese, and infertile, and the *Creb1- Crtc1* pathway mediates the central effects of hormones and nutrients on energy balance and fertility[92]. rs2003476 is associated with the expression of *CRLF1*[51], a protein coding gene 88kb upstream. *CRLF1* expression changes significantly during human adipogenesis[93]. *CRLF1* also shows differential expression levels in gluteal and abdominal subcutaneous adipose tissue in humans[94].

rs765876 (WEAF 48.8%, beta= -0.020, BMI *P-value*=9.64×10^{-10}) resides in an intron of *HIVEP2*, which codes for a transcription factor that binds to the enhancer sequences of various genes including somatostatin receptor II[95]. Mouse models of this gene show smaller body, with reduced fat mass[96].

Variants associated with hip circumference adjusted for BMI:

rs10044000 (WEAF 39%, beta=0.0157, *P-value*=6.45×10^{-13}) variant overlaps with the coding region of the gene *CATSPER3* , where it causes a synonymous mutation. rs10044000 has been previously associated with height and *CATSPER3* is a known locus for height[78,97] and bulimia[98]. rs10044000 has been shown to be an eQTL for *PITX1*[51] gene located 20kb upstream. The protein product of this gene is a transcription factor and involved in skeletal development[99]. Various congenital diseases are associated to this gene and all characterized by skeletal abnormalities[100,101]. Animal models also highlight the effect of this gene on skeletal development[102].

rs35874463 (WEAF 58.2%, beta=0.0374, *P-value*=9.26×10^{-17}) results in an isoleucine to valine substitution in *SMAD3*. The substitution caused by this variant is predicted to be benign (Polyphen score = 0.007). Position 65 is directly adjacent to the metal binding site, which is required for the RNA binding function of the MH1 domain, valine is frequently found in the homolog position. Mutations of this gene were implicated in the aneurysms-osteoarthritis syndrome[103], characterized by early onset osteoarthritis in the knees, hands and spine. Animal models of this gene shows a series of skeletal phenotypes[104] highlighting the gene's role in ossification and skeletal development. rs35874463 is also associated with height[64] and heart developmental failures[105].

Variants associated with waist circumference adjusted for BMI:

rs28610092 (WEAF 17%, beta= -0.021, *P-value*=8.92×10^{-16}) resides in the promoter flanking region of *PKD1*, which has been found to be active in myoblasts, fibroblasts and osteoblasts[75]. rs28610092 is associated with the expression of *PKD1*[51]. Although the primary function of this gene is the regulation of the development of the renal tubulogenesis[106] and is the primary causal gene for adult type-1 polycystic kidney disease[107], animal models show diverse phenotypes including defects in the myoskeletal development[108].

rs577721086 (WEAF 5.1%, beta=0.056, *P-value*=2.54×10^{-39}) is located in the 5' untranslated region of *RSPO3*, its position is highly conserved (GERP score=3.77) and has a number of epigenetic marks indicative of being an active promoter in several tissues[55], GWAVA[109] score=0.63. Intronic variation at this locus has previously been associated with waist circumference and waist to hip ratio adjusted for BMI (Table S14) and whilst collider bias might have complicated the interpretation of this signal[110], it appears

to be a genuine contributor to variance in waist circumference relative to trunk and not BMI (stage 1 *P-value*=0.14) (Figure S27). rs577721086 has been previously associated with waist to hip ratio adjusted for BMI (Table S14).

Variants associated with other anthropometric traits:

rs11042397 is associated with hip circumference (WEAF 56.4%, beta= 0.047, *P-value*=$5.20x10^{-11}$) and is located in an intron of *ZNF143*, which codes for a transcription factor involved in early developmental processes in animal models[111]. rs11042397 is tagged in HapMap by rs2290424 (r^2=0.963) with *P-value*=0.02[112]. rs11042397 is located 161kb upstream of *SWAP70* , known for affecting bone mass and osteoclast function through modulating f-actin[113] and 187kb away from *TMEM49B*, implicated in metabolic processes in animal models[114].

rs62065847 is associated with waist circumference (WEAF 48.6%, beta= -0.022, *P-value*=$2.86x10^{-11}$). This intergenic variant resides in a reported enhancer[115] and is associated with the expression of many genes in close proximity including *HOXB2*, which is involved in skeletal abnormalities in animal models[116,117].

rs2082881 is a novel TRFM signal (WEAF 24.4%, beta=0.0834, *P-value*=$9.91x10^{-9}$), but it is a known variant for BMI and height. rs2082881 overlaps with an intron of *CENPO*, which has been associated with height[64]. Animal models of this gene shows increased body length[71]. rs2082881 is an eQTL for other genes including *NCOA1* and *ADCY3*[51]. *NCOA1* is a nuclear receptor coactivator, involved in the coactivation of steroid hormone receptors and activates the expression of a series of genes involved in development. Mouse model of this gene shows diverse phenotypes including obesity[118]. Several obesity and obesity related signals were associated to *ADCY3*, whose protein product, through its adenyl-cyclase activity, is an important regulator of energy balance.

rs6901225 is a novel association for weight (WEAF 12%, beta=-0.0377, *P-value*=$4.20x10^{-13}$) and is a known variant for height. Although the variant is located in the intergenic region, it is associated with the expression of multiple transcription factors[51] such as *ZNF322* and *ABT1*. *ZNF322*, a zinc-finger protein, is responsible for the regulation of many embryonic genes[119]. *ABT1* interacts with *IGHMBP2*, which is important for skeletal phenotypes[120].

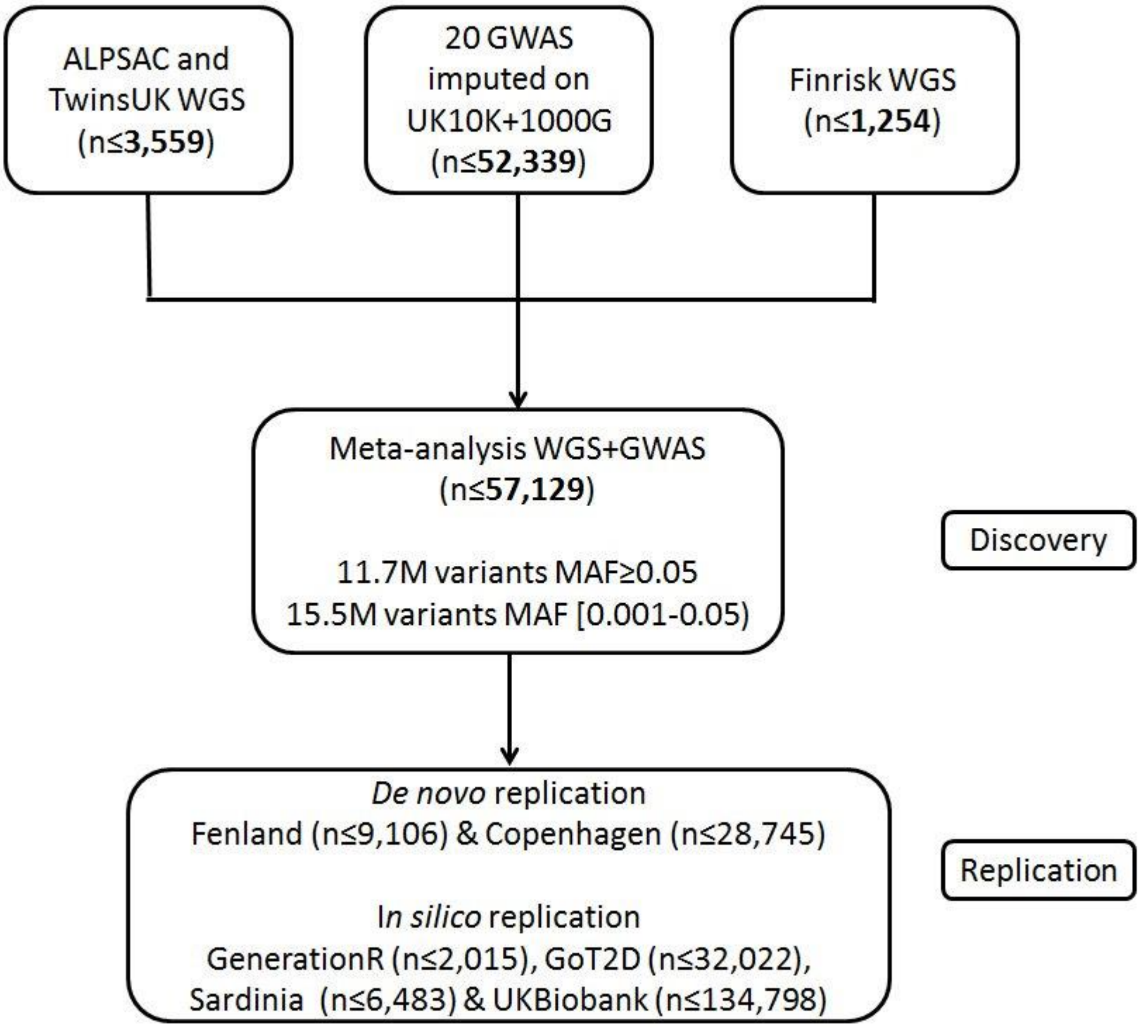

Figure S1: Study design for single marker tests.

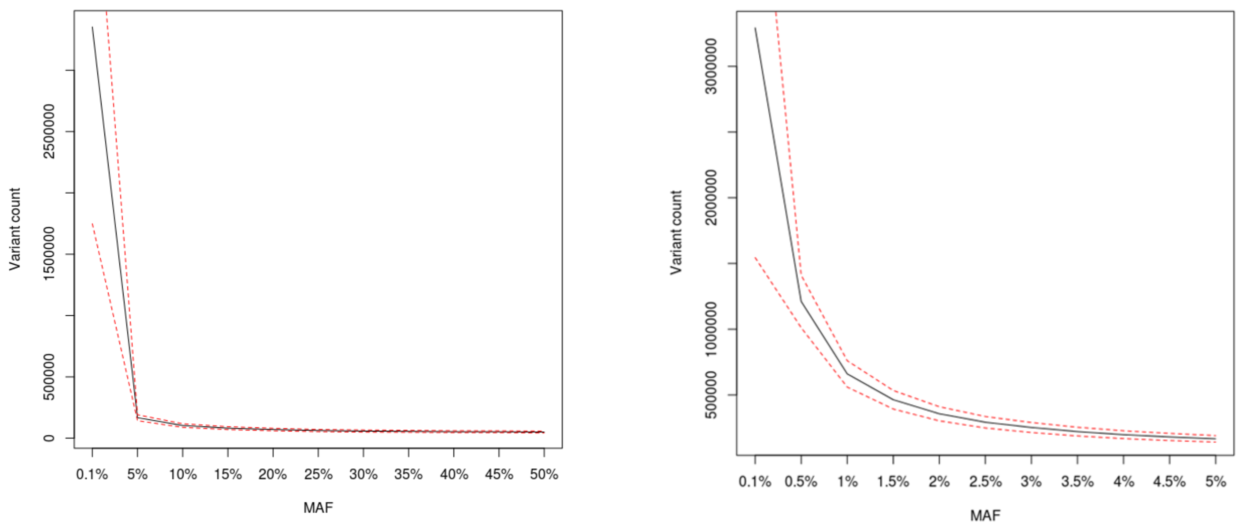

Figure S2: A minor allele frequency (MAF) histogram of variants that have passed the imputation quality threshold (0.4) across the imputed datasets that are available genome-wide.
In the left graph we plotted the whole MAF range (0.01% -50%) and in the right graph we zoomed in MAF between 0.01% -5%. The y-axis is the average number of variants across our genome-wide imputed datasets (arcOGEN, UKHLS, ALSPAC, TwinsUK, 1958 Birth Cohort, INGI-Carl, INGI-FVG, HELIC MANOLIS, HELIC Pomak, INCIPE1, INCIPE2, LURIC, Rotterdam Study-1, Rotterdam Study-2, Rotterdam Study-3, TEENAGE, INGI-VB and UK Biobank).

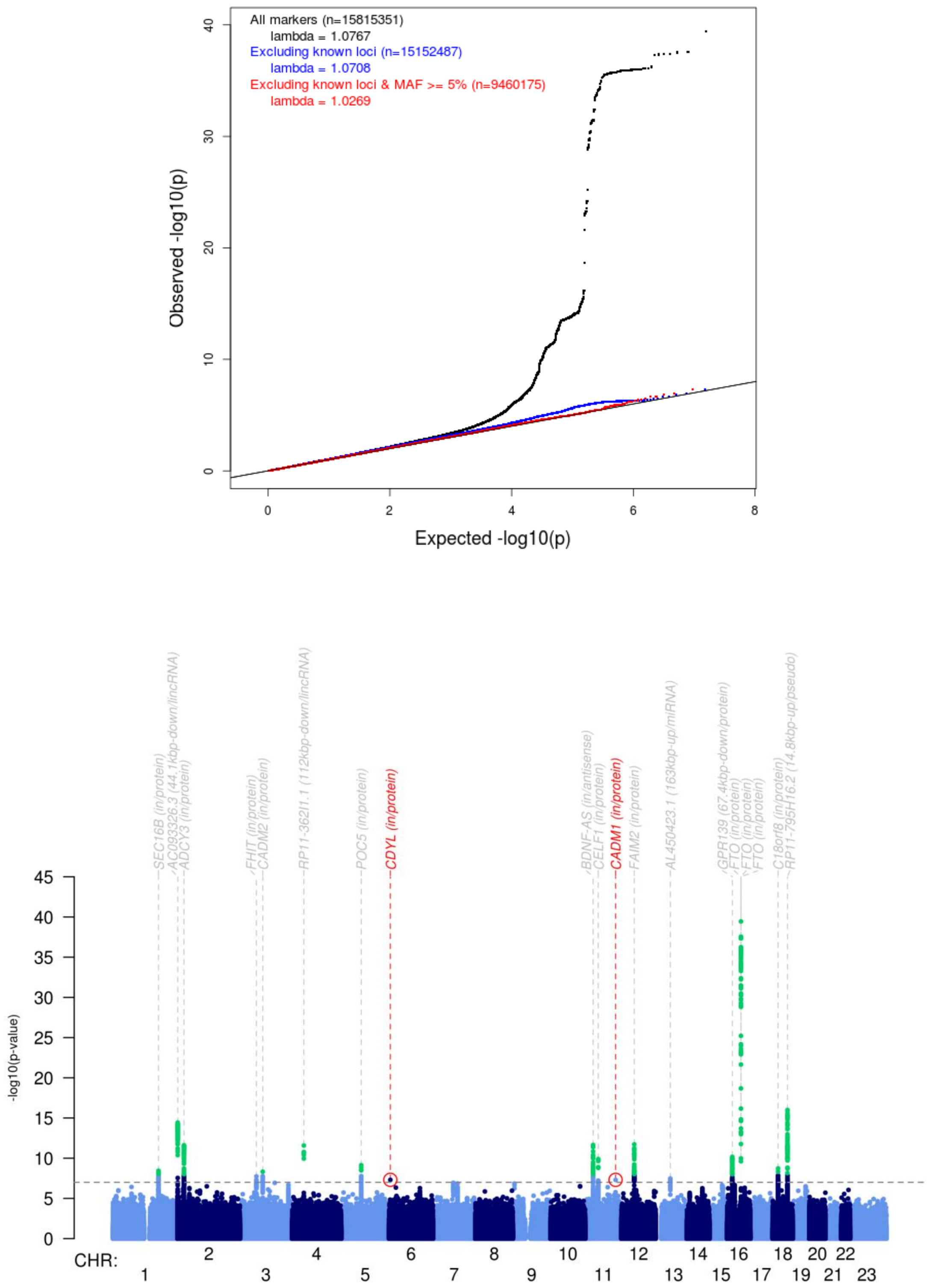

Figure S3: Summary plots of body mass index (BMI) sex-combined meta-analysis.
Quantile-quantile plot of SNP associations. All SNPs are plotted in black, after excluding previously known loci (±500 kb) in blue, and after excluding previously known and common loci (±500 kb) in red. Manhattan plot showing in green loci with $P \leq 10^{-8}$. Loci with $P \leq 10^{-7}$ are labeled with the nearest protein coding gene in grey if they are known and in red if they are novel. The reported gene is the closest in physical distance. The horizontal line is drawn at 10^{-7}.

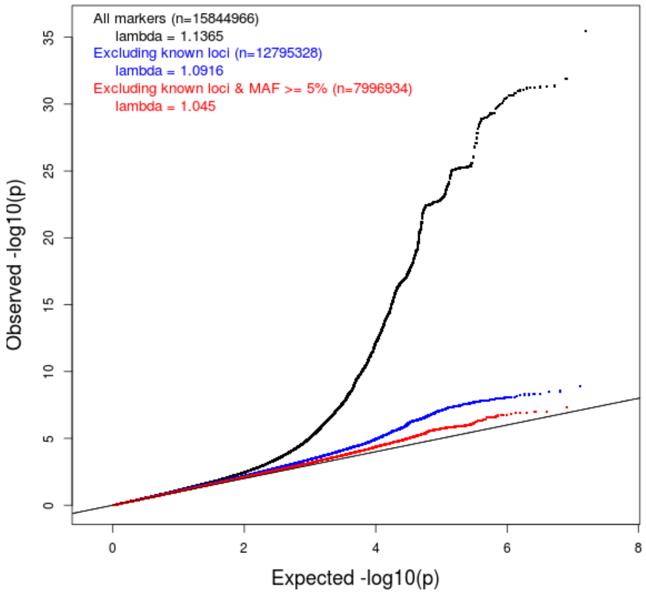

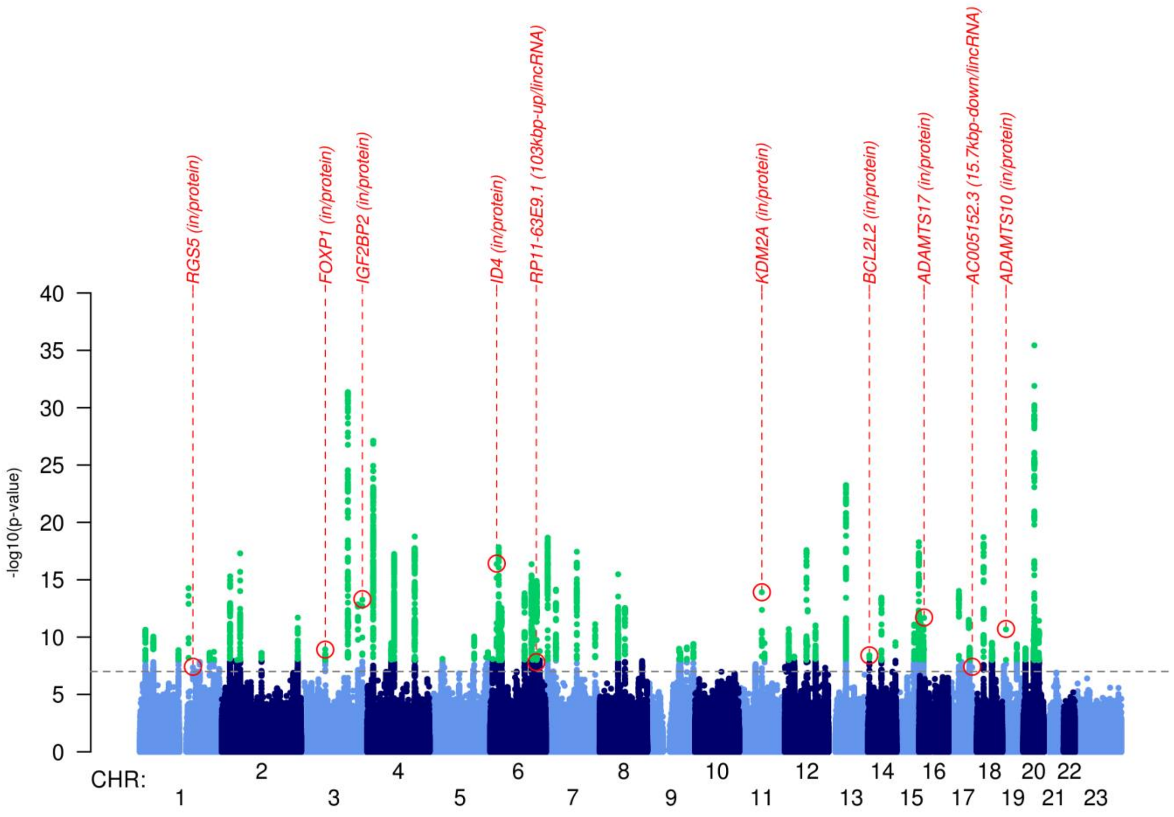

Figure S4: Summary plots of height sex-combined meta-analysis.
Quantile-quantile plot of SNP associations. All SNPs are plotted in black, after excluding previously known loci (±500 kb) in blue, and after excluding previously known and common loci (±500 kb) in red. Manhattan plot showing in green loci with $P \leq 10^{-8}$. Loci with $P \leq 10^{-7}$ are labeled with the nearest protein coding gene in red if they are novel. The reported gene is the closest in physical distance. The horizontal line is drawn at 10^{-7}. Only novel signals are annotated for Height, to avoid overcrowding in the graph.

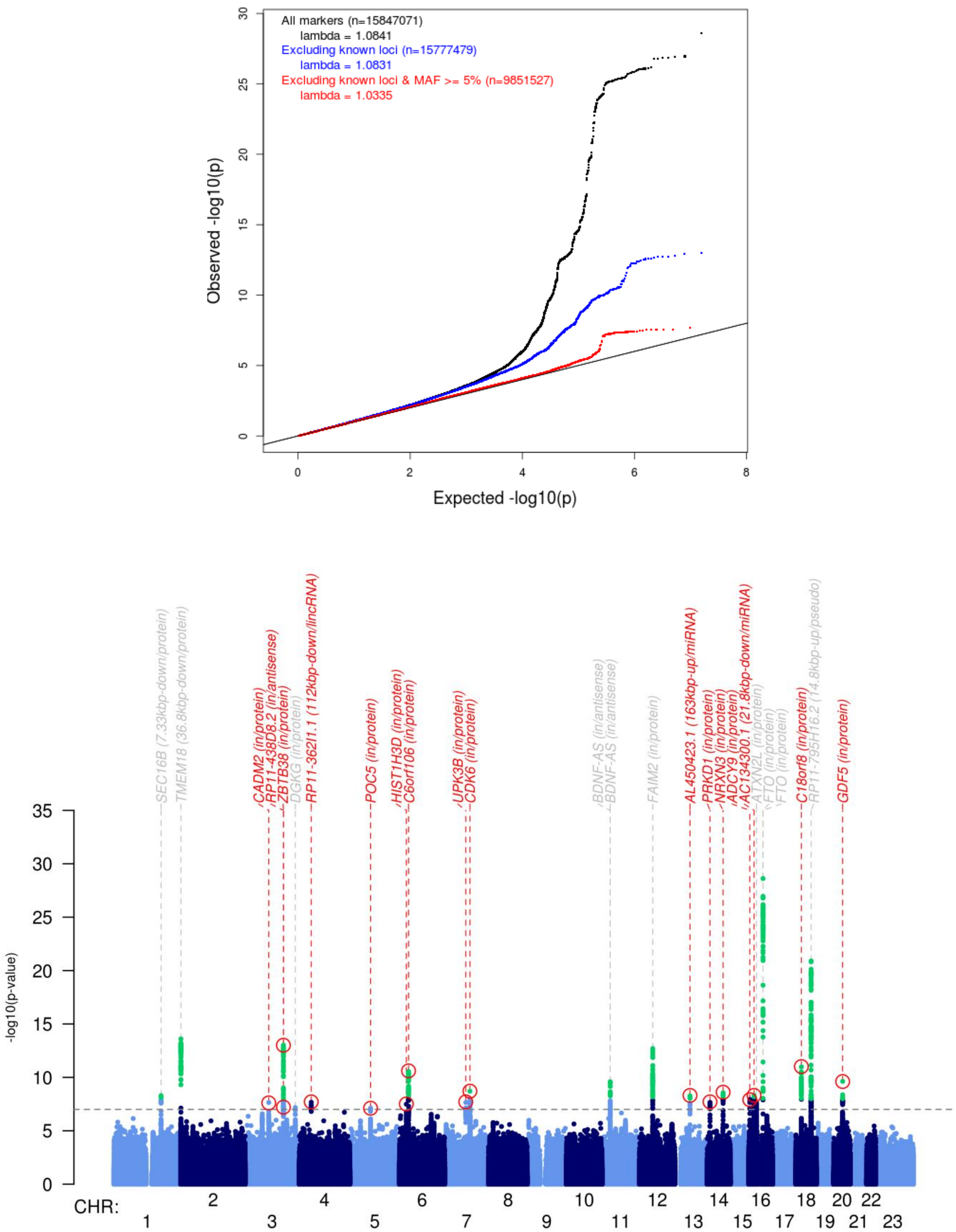

Figure S5: Summary plots of weight sex-combined meta-analysis.
Quantile-quantile plot of SNP associations. All SNPs are plotted in black, after excluding previously known loci (±500 kb) in blue, and after excluding previously known and common loci (±500 kb) in red. Manhattan plot showing in green loci with $P \leq 10^{-8}$. Loci with $P \leq 10^{-7}$ are labeled with the nearest protein coding gene in grey if they are known and in red if they are novel. The reported gene is the closest in physical distance. The horizontal line is drawn at 10^{-7}.

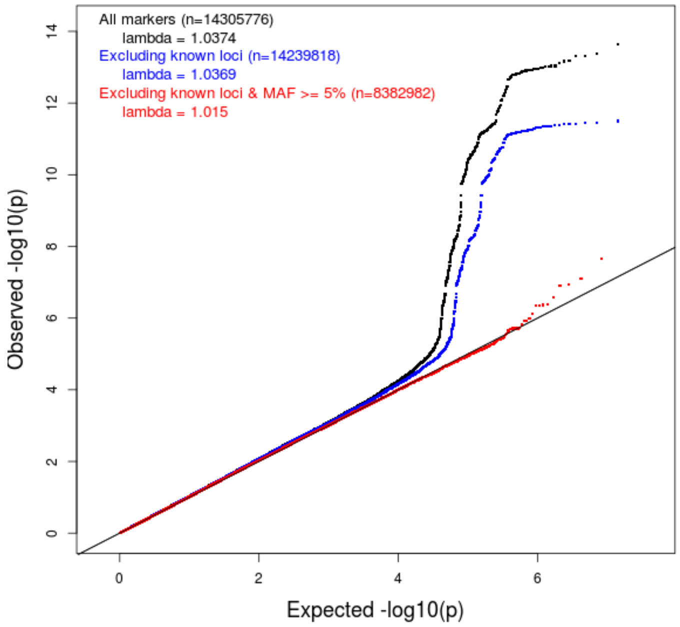

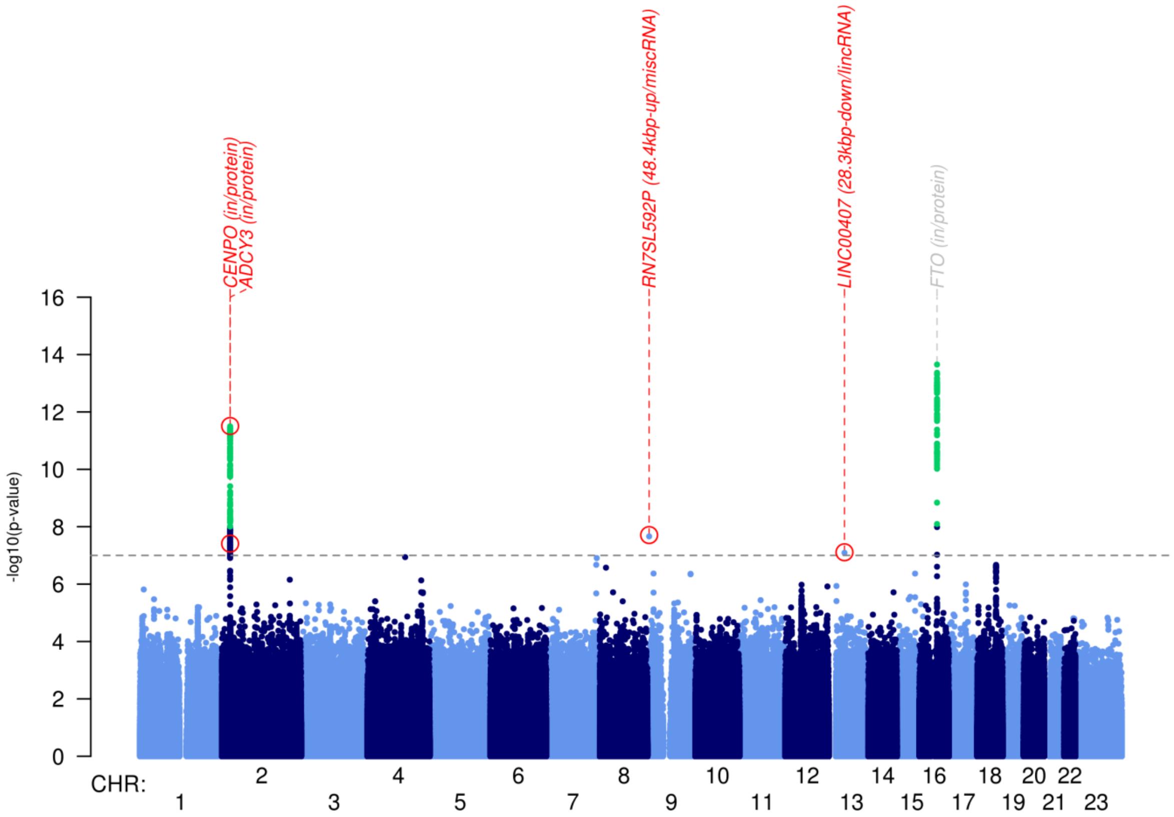

Figure S6: Summary plots of total fat mass (TFM) sex-combined meta-analysis.
Quantile-quantile plot of SNP associations. All SNPs are plotted in black, after excluding previously known loci (±500 kb) in blue, and after excluding previously known and common loci (±500 kb) in red. Manhattan plot showing in green loci with $P \leq 10^{-8}$. Loci with $P \leq 10^{-7}$ are labeled with the nearest protein coding gene in grey if they are known and in red if they are novel. The reported gene is the closest in physical distance. The horizontal line is drawn at 10^{-7}.

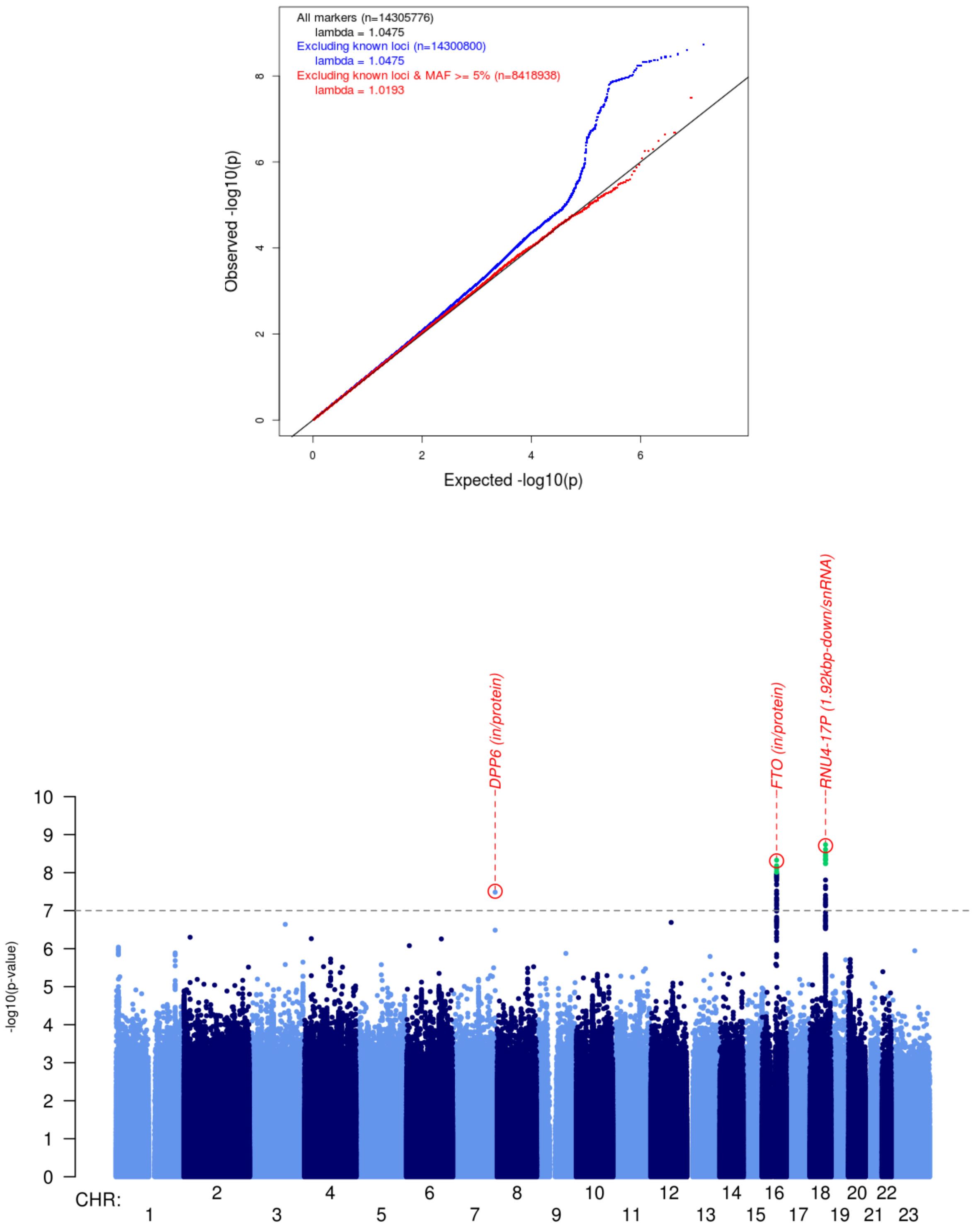

Figure S7: Summary plots of total lean mass (TLM) sex-combined meta-analysis.
Quantile-quantile plot of SNP associations. All SNPs are plotted in black, after excluding previously known loci (±500 kb) in blue, and after excluding previously known and common loci (±500 kb) in red. Manhattan plot showing in green loci with $P \leq 10^{-8}$. Loci with $P \leq 10^{-7}$ are labeled with the nearest protein coding gene in grey if they are known and in red if they are novel. The reported gene is the closest in physical distance. The horizontal line is drawn at 10^{-7}.

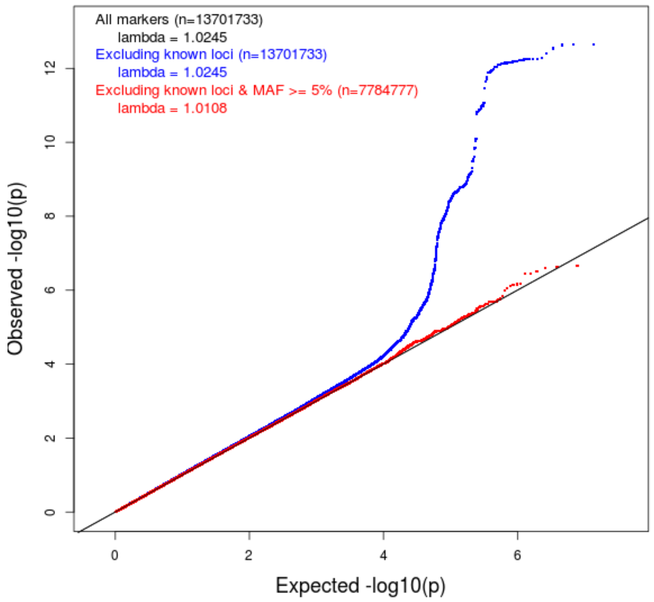

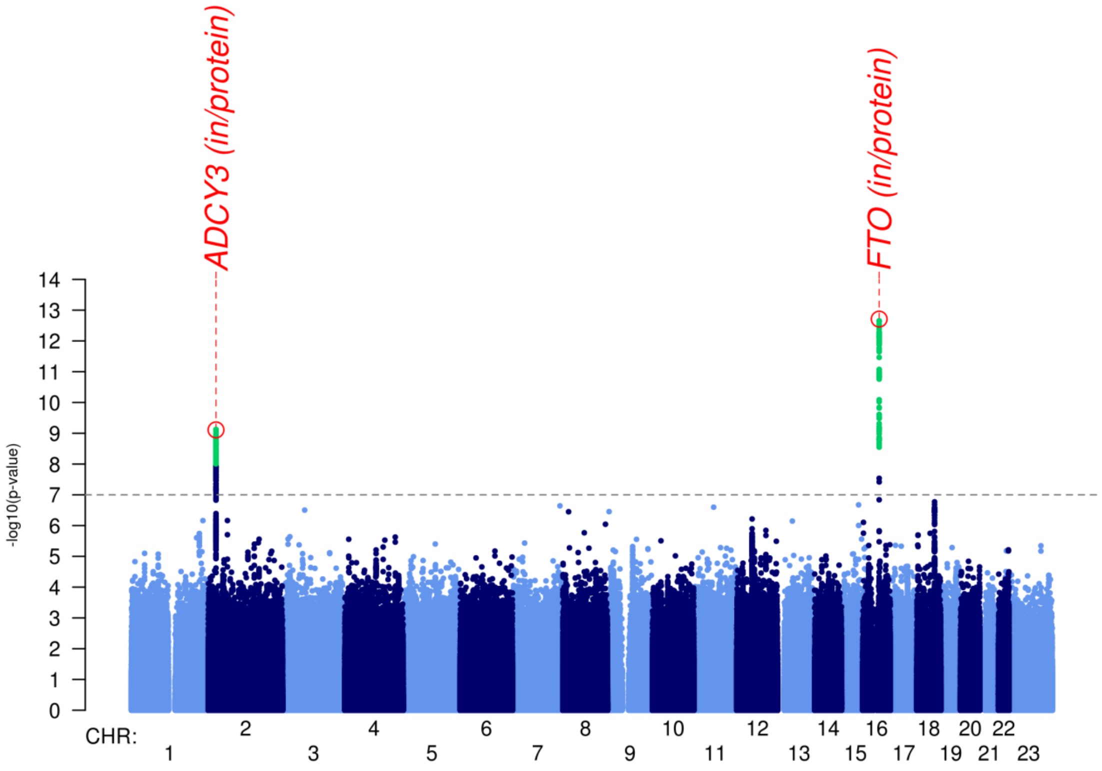

Figure S8: Summary plots of trunk fat mass (TRFM) sex-combined meta-analysis.
Quantile-quantile plot of SNP associations. All SNPs are plotted in black, after excluding previously known loci (±500 kb) in blue, and after excluding previously known and common loci (±500 kb) in red. Manhattan plot showing in green loci with $P \leq 10^{-8}$. Loci with $P \leq 10^{-7}$ are labeled with the nearest protein coding gene in grey if they are known and in red if they are novel. The reported gene is the closest in physical distance. The horizontal line is drawn at 10^{-7}.

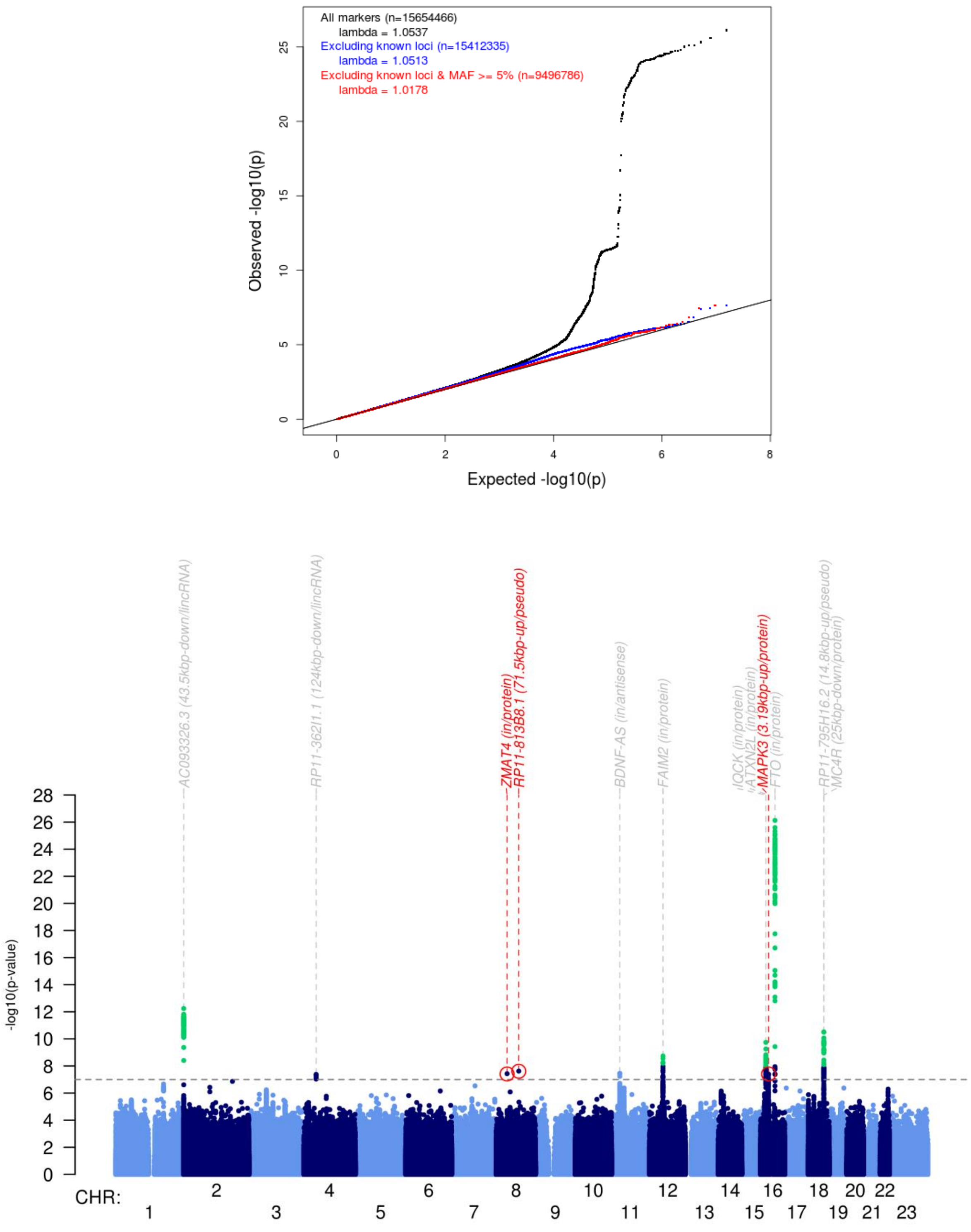

Figure S9: Summary plots of waist circumference sex-combined meta-analysis.

Quantile-quantile plot of SNP associations. All SNPs are plotted in black, after excluding previously known loci (±500 kb) in blue, and after excluding previously known and common loci (±500 kb) in red. Manhattan plot showing in green loci with $P \leq 10^{-8}$. Loci with $P \leq 10^{-7}$ are labeled with the nearest protein coding gene in grey if they are known and in red if they are novel. The reported gene is the closest in physical distance. The horizontal line is drawn at 10^{-7}.

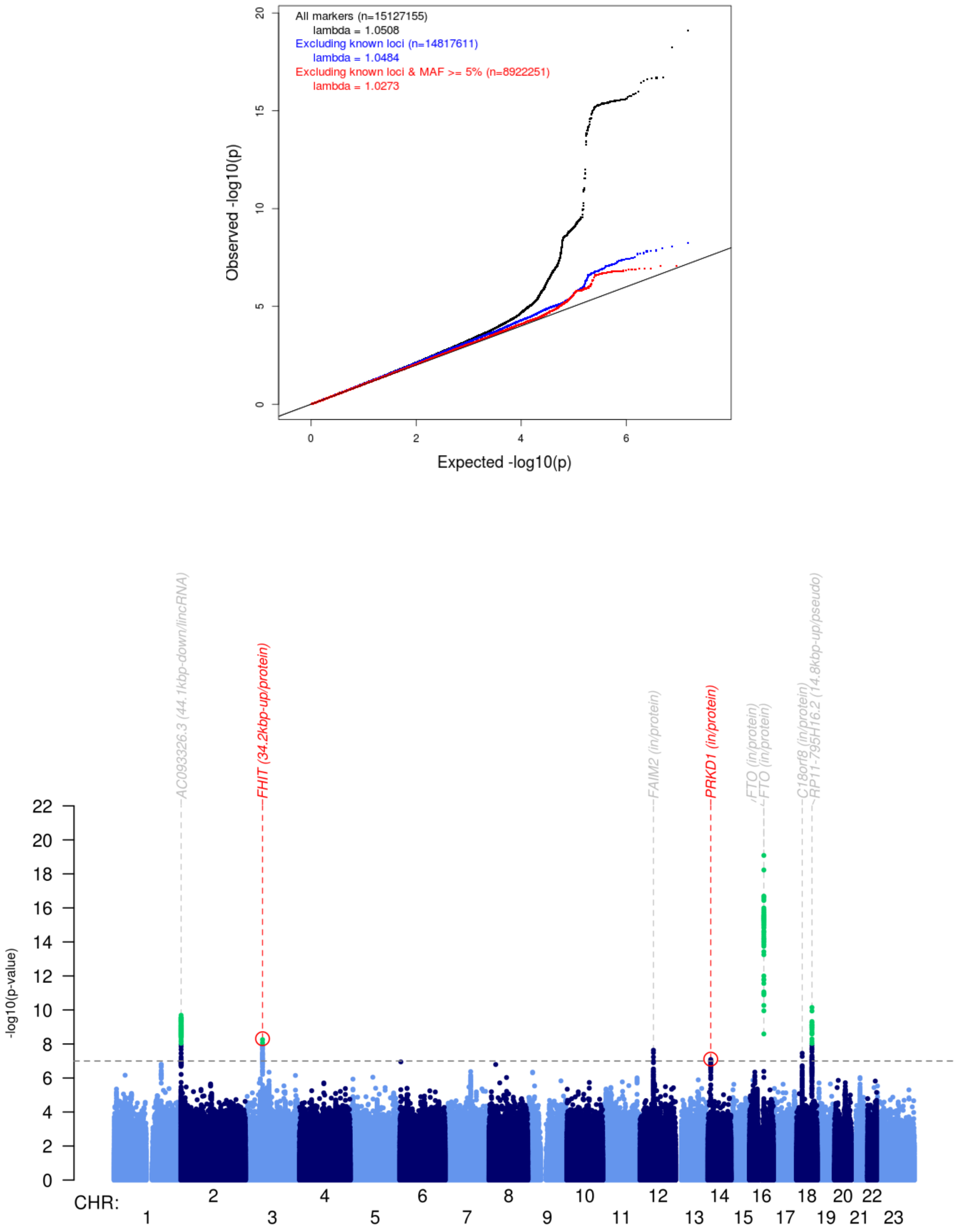

Figure S10: Summary plots of hip circumference sex-combined meta-analysis.
Quantile-quantile plot of SNP associations. All SNPs are plotted in black, after excluding previously known loci (±500 kb) in blue, and after excluding previously known and common loci (±500 kb) in red. Manhattan plot showing in green loci with $P \leq 10^{-8}$. Loci with $P \leq 10^{-7}$ are labeled with the nearest protein coding gene in grey if they are known and in red if they are novel. The reported gene is the closest in physical distance. The horizontal line is drawn at 10^{-7}.

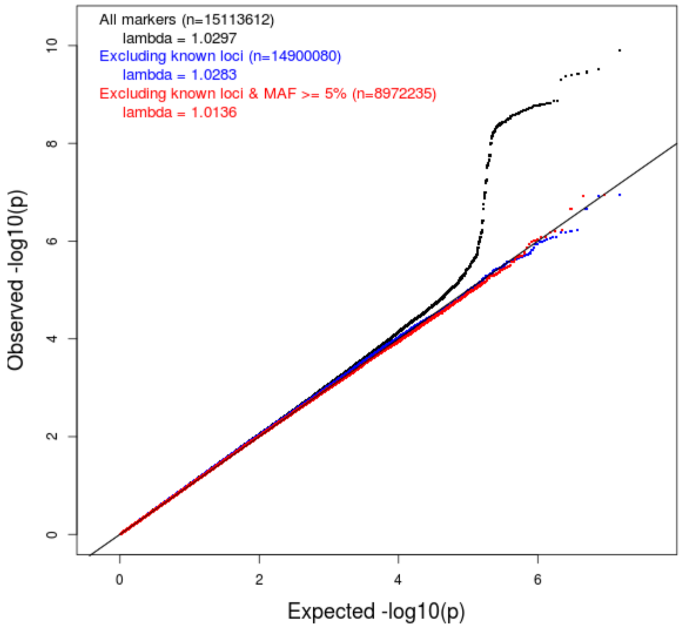

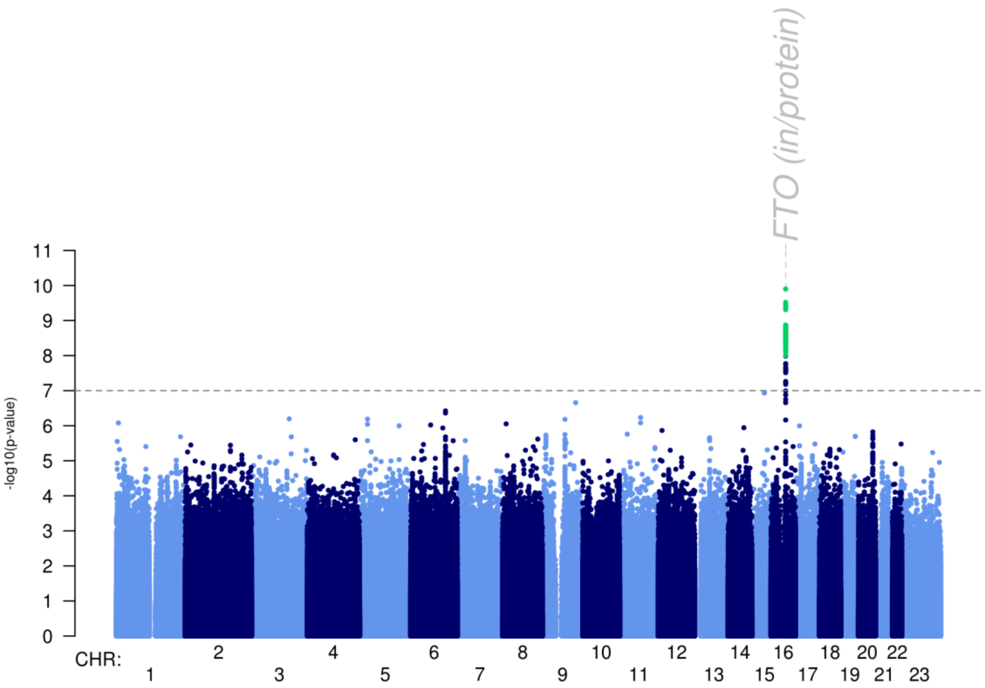

Figure S11: Summary plots of waist-to-hip ratio (WHR) sex-combined meta-analysis.
Quantile-quantile plot of SNP associations. All SNPs are plotted in black, after excluding previously known loci (±500 kb) in blue, and after excluding previously known and common loci (±500 kb) in red. Manhattan plot showing in green loci with $P \leq 10^{-8}$. Loci with $P \leq 10^{-7}$ are labeled with the nearest protein coding gene in grey if they are known and in red if they are novel. The reported gene is the closest in physical distance. The horizontal line is drawn at 10^{-7}.

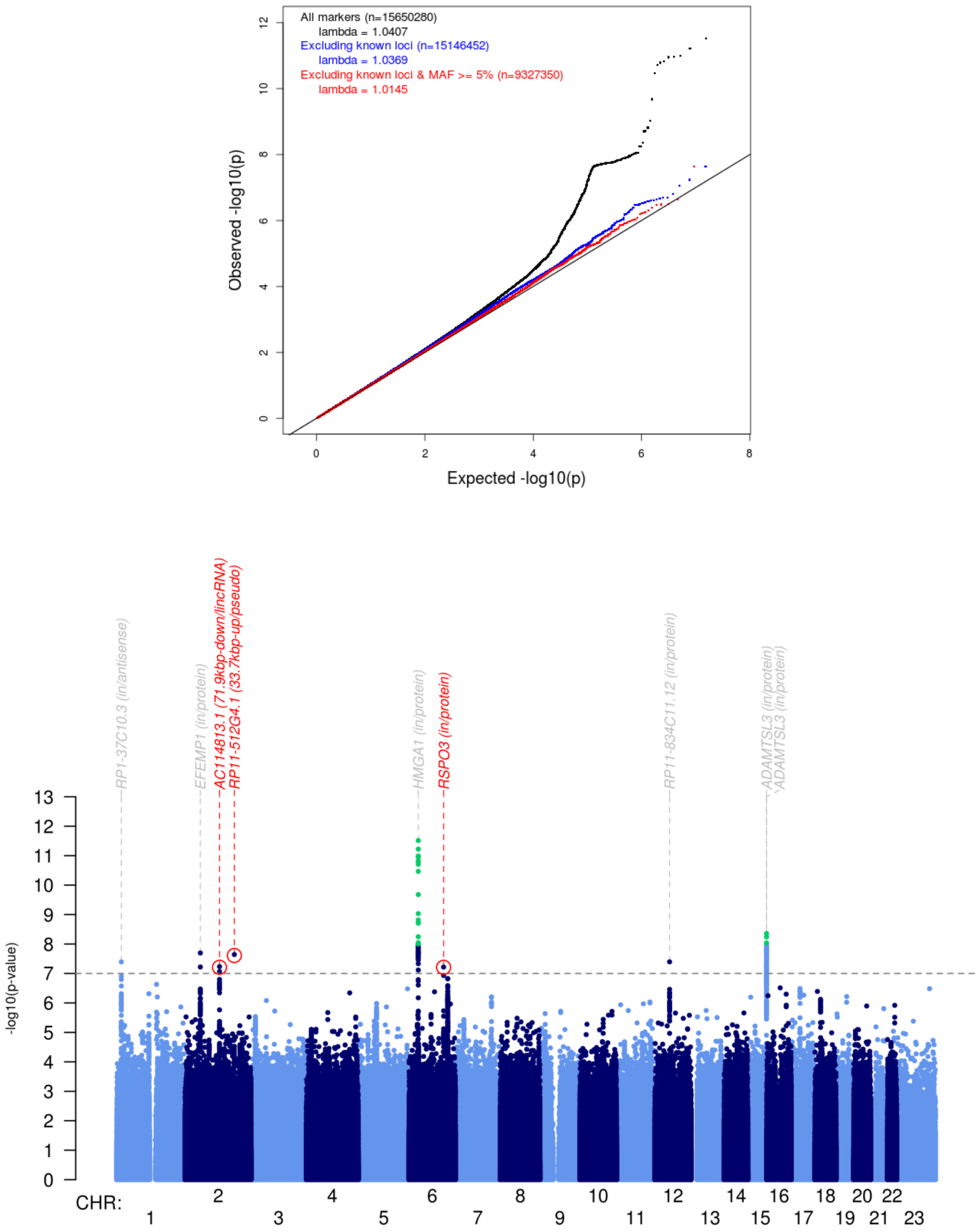

Figure S12: Summary plots of waist circumference adjusted for BMI sex-combined meta-analysis. Quantile-quantile plot of SNP associations. All SNPs are plotted in black, after excluding previously known loci (±500 kb) in blue, and after excluding previously known and common loci (±500 kb) in red. Manhattan plot showing in green loci with $P \leq 10^{-8}$. Loci with $P \leq 10^{-7}$ are labeled with the nearest protein coding gene in grey if they are known and in red if they are novel. The reported gene is the closest in physical distance. The horizontal line is drawn at 10^{-7}.

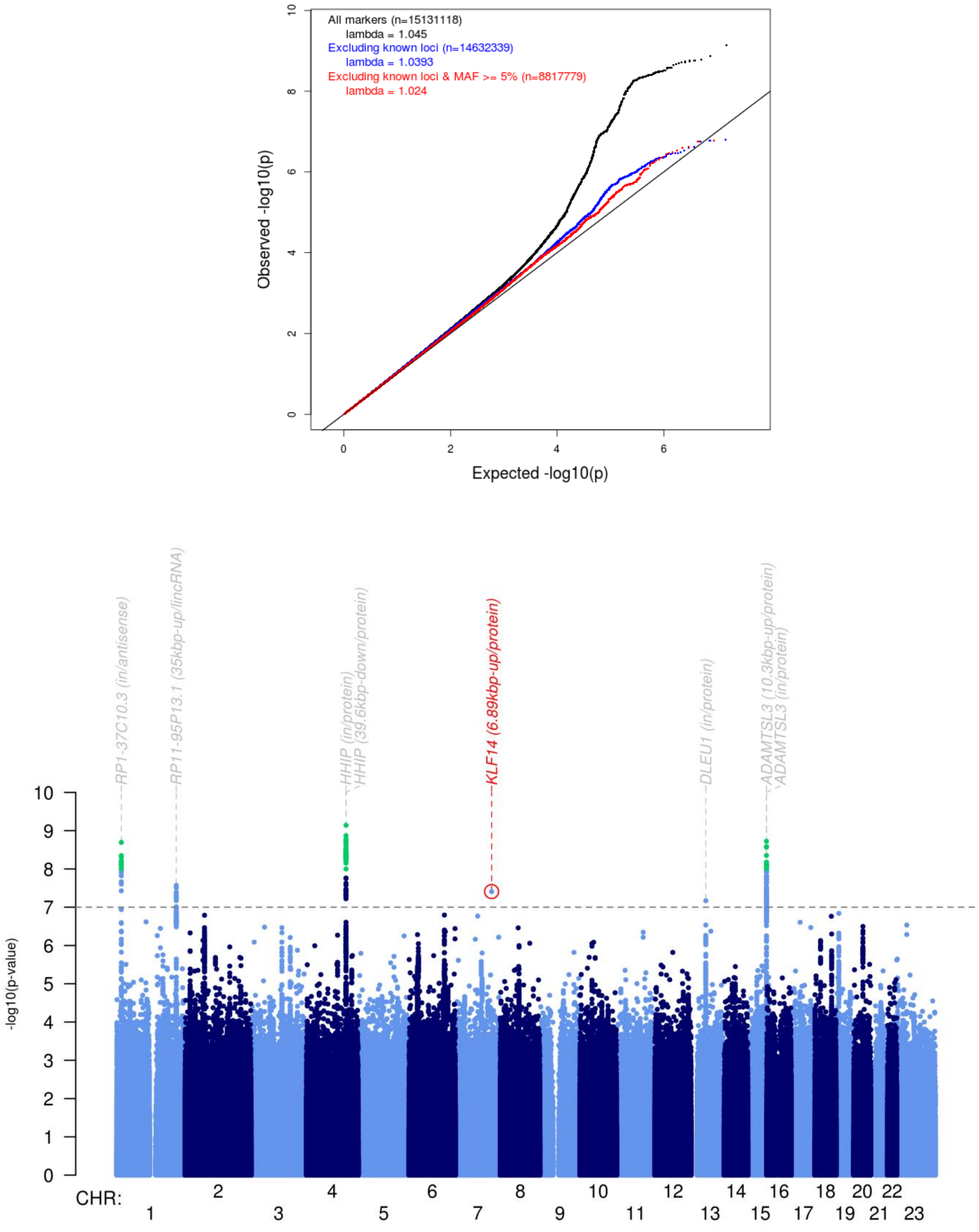

Figure S13: Summary plots of hip circumference adjusted for BMI sex-combined meta-analysis. Quantile-quantile plot of SNP associations. All SNPs are plotted in black, after excluding previously known loci (±500 kb) in blue, and after excluding previously known and common loci (±500 kb) in red. Manhattan plot showing in green loci with $P \leq 10^{-8}$. Loci with $P \leq 10^{-7}$ are labeled with the nearest protein coding gene in grey if they are known and in red if they are novel. The reported gene is the closest in physical distance. The horizontal line is drawn at 10^{-7}.

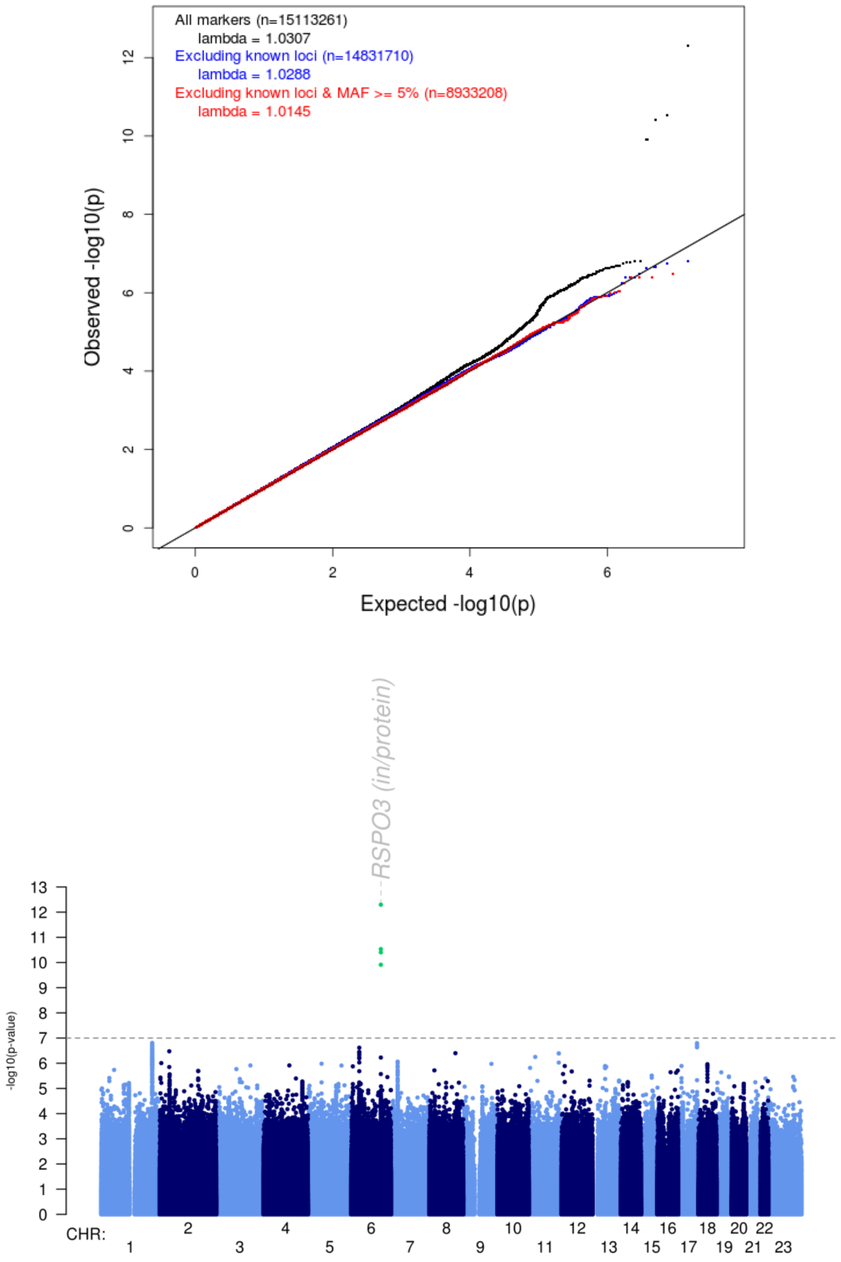

Figure S14: Summary plots of wait-to-hip ratio (WHR) adjusted for BMI sex-combined meta-analysis. Quantile-quantile plot of SNP associations. All SNPs are plotted in black, after excluding previously known loci (±500 kb) in blue, and after excluding previously known and common loci (±500 kb) in red. Manhattan plot showing in green loci with $P \leq 10^{-8}$. Loci with $P \leq 10^{-7}$ are labeled with the nearest protein coding gene in grey if they are known and in red if they are novel. The reported gene is the closest in physical distance. The horizontal line is drawn at 10^{-7}.

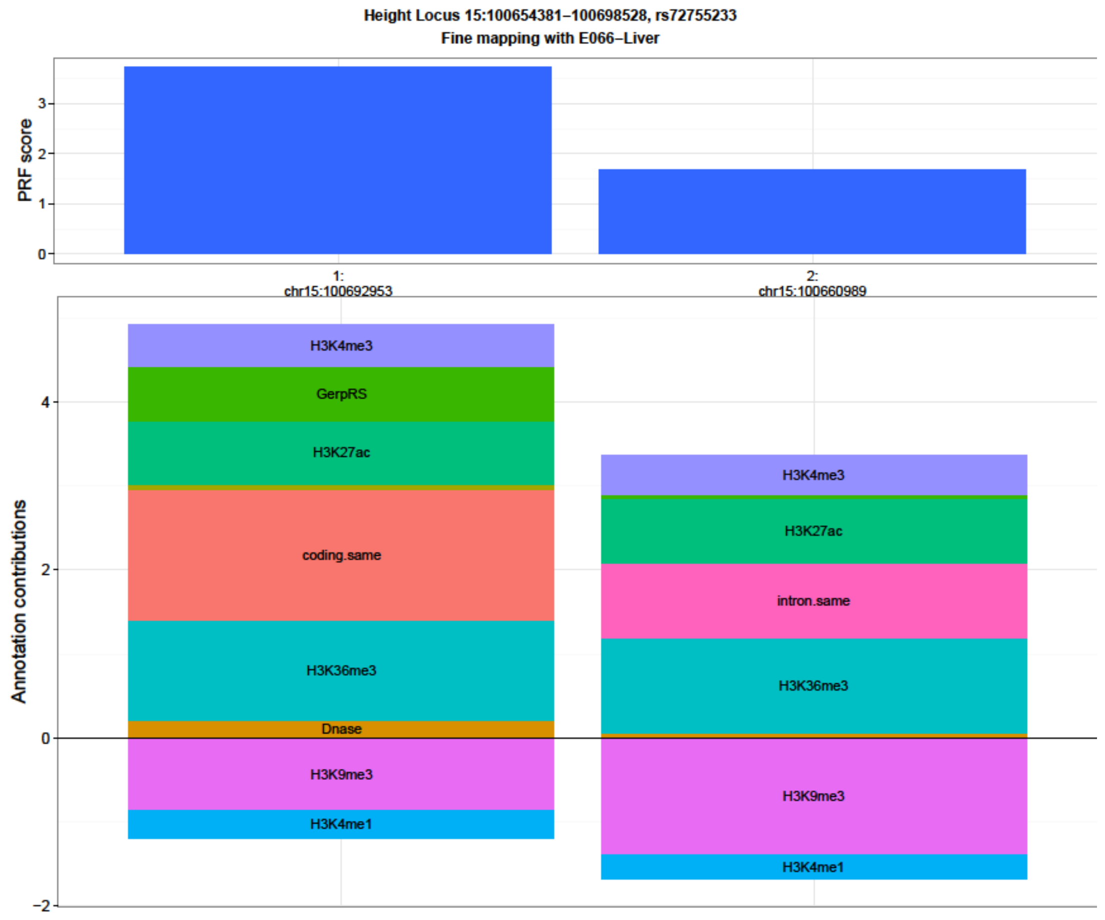

Height Locus 15:100654381–100698528, rs72755233
Fine mapping with E066–Liver
PRF score
0
1
2
3
1:
chr15:100692953
2:
chr15:100660989
Annotation contributions
−2
0
2
4
H3K4me3
GerpRS
H3K27ac
coding.same
H3K36me3
Dnase
H3K9me3
H3K4me1
H3K4me3
H3K27ac
intron.same
H3K36me3
H3K9me3
H3K4me1

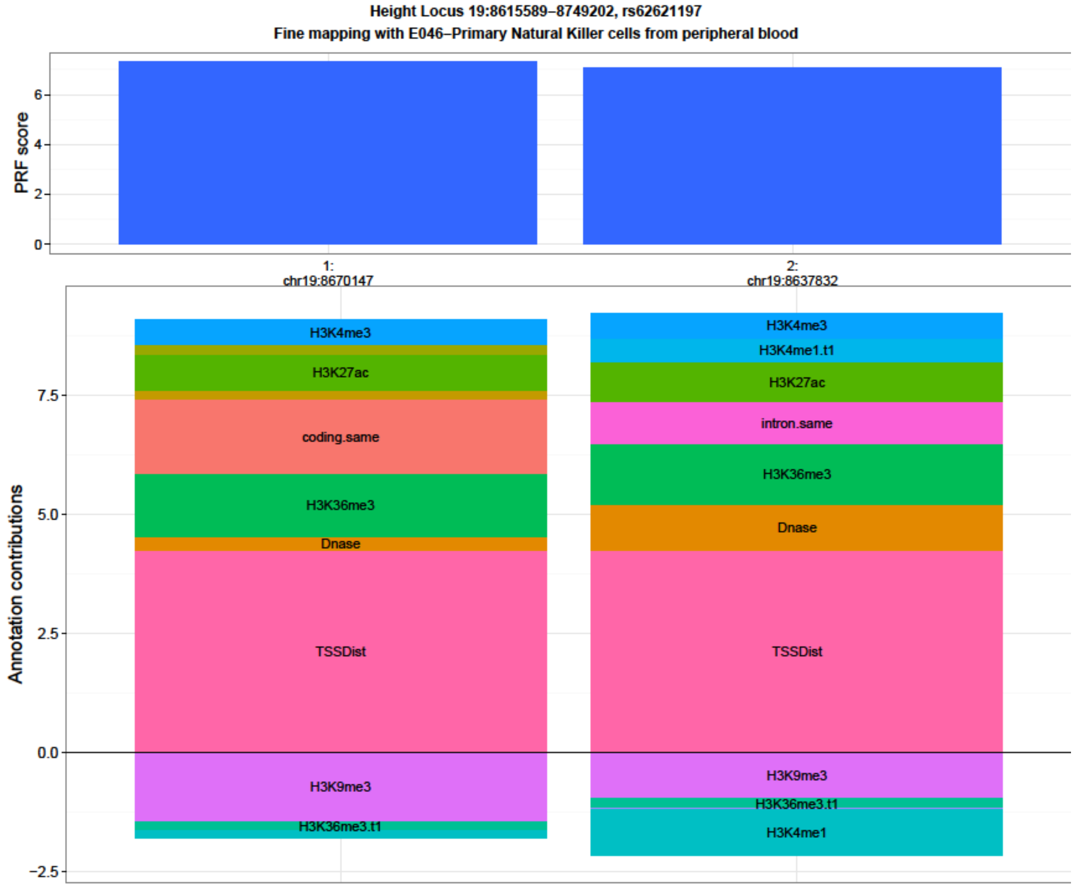
Height Locus 19:8615589–8749202, rs62621197
Fine mapping with E046–Primary Natural Killer cells from peripheral blood
PRF score
0
2
4
6
1:
chr19:8670147
2:
chr19:8637832
Annotation contributions
−2.5
0.0
2.5
5.0
7.5
H3K4me3
H3K27ac
coding.same
H3K36me3
Dnase
TSSDist
H3K9me3
H3K36me3.t1
H3K4me3
H3K4me1.t1
H3K27ac
intron.same
H3K36me3
Dnase
TSSDist
H3K9me3
H3K36me3.t1
H3K4me1

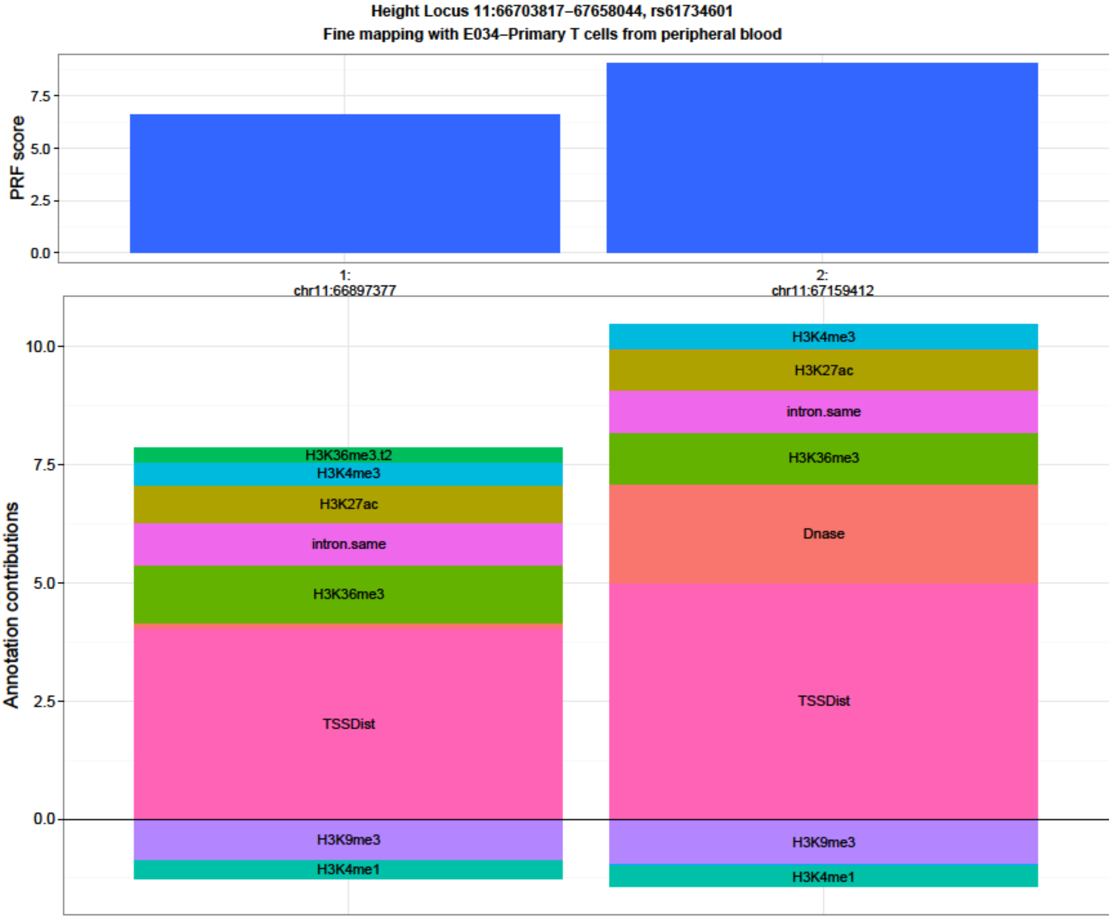
Height Locus 11:66703817–67658044, rs61734601
Fine mapping with E034–Primary T cells from peripheral blood
PRF score
0.0
2.5
5.0
7.5
1:
chr11:66897377
2:
chr11:67159412
Annotation contributions
0.0
2.5
5.0
7.5
10.0
H3K36me3.t2
H3K4me3
H3K27ac
intron.same
H3K36me3
TSSDist
H3K9me3
H3K4me1
H3K4me3
H3K27ac
intron.same
H3K36me3
Dnase
TSSDist
H3K9me3
H3K4me1

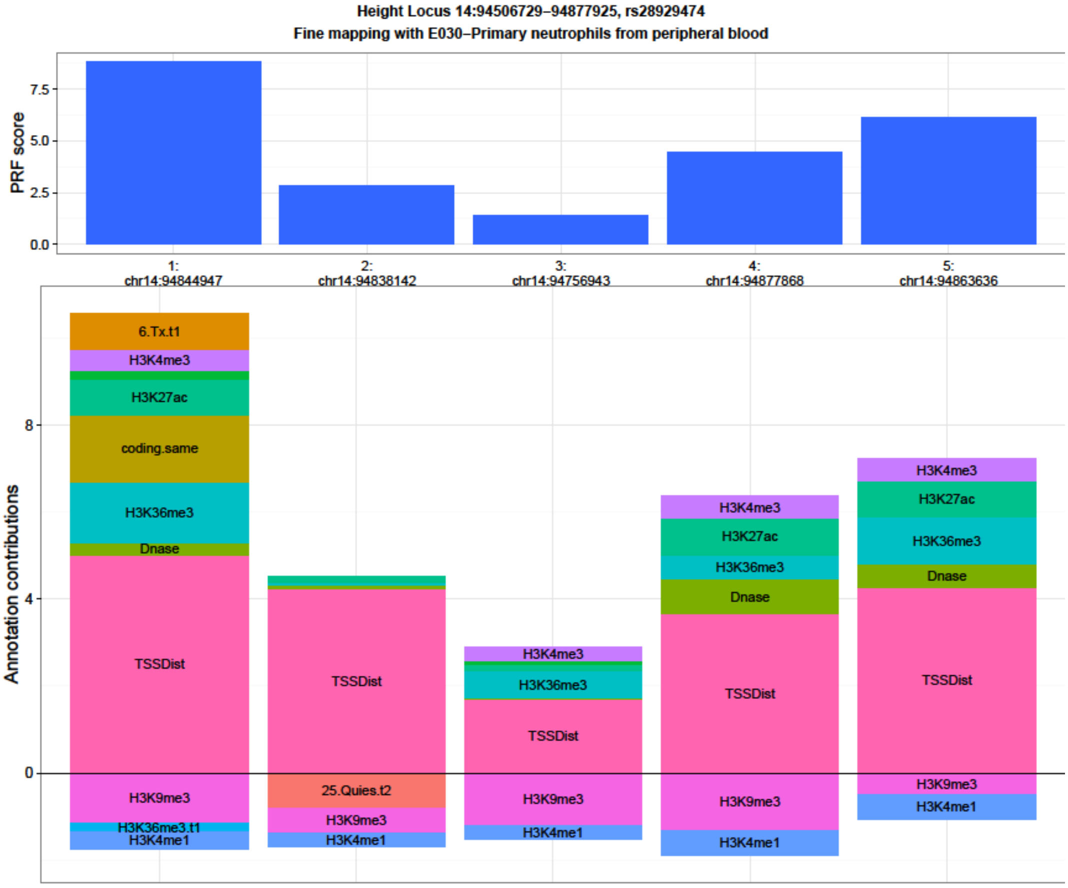

Height Locus 14:94506729–94877925, rs28929474
Fine mapping with E030–Primary neutrophils from peripheral blood
PRF score
7.5
5.0
2.5
0.0
1:
chr14:94844947
2:
chr14:94838142
3:
chr14:94756943
4:
chr14:94877868
5:
chr14:94863636
Annotation contributions
8
4
0
6.Tx.t1
H3K4me3
H3K27ac
coding.same
H3K36me3
Dnase
TSSDist
H3K9me3
H3K36me3.t1
H3K4me1
25.Quies.t2

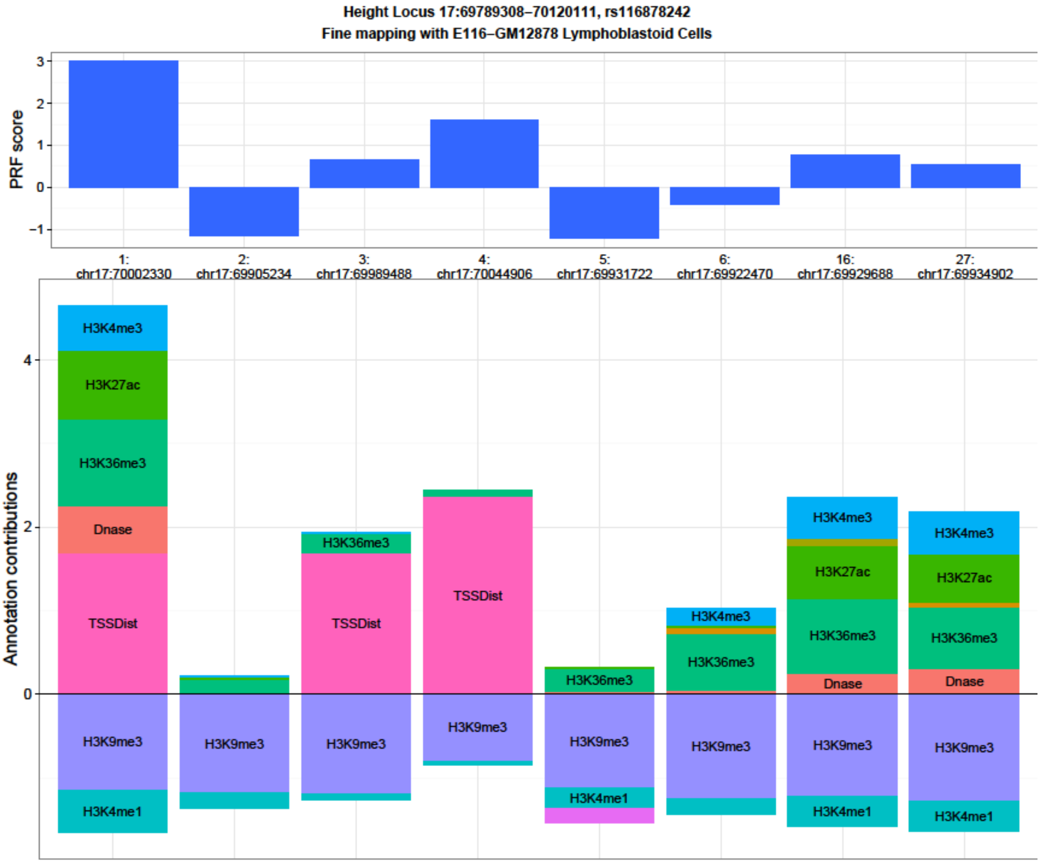

Height Locus 17:69789308–70120111, rs116878242
Fine mapping with E116–GM12878 Lymphoblastoid Cells
PRF score
3
2
1
0
−1
1:
chr17:70002330
2:
chr17:69905234
3:
chr17:69989488
4:
chr17:70044906
5:
chr17:69931722
6:
chr17:69922470
16:
chr17:69929688
27:
chr17:69934902
Annotation contributions
4
2
0
H3K4me3
H3K27ac
H3K36me3
Dnase
TSSDist
H3K9me3
H3K4me1

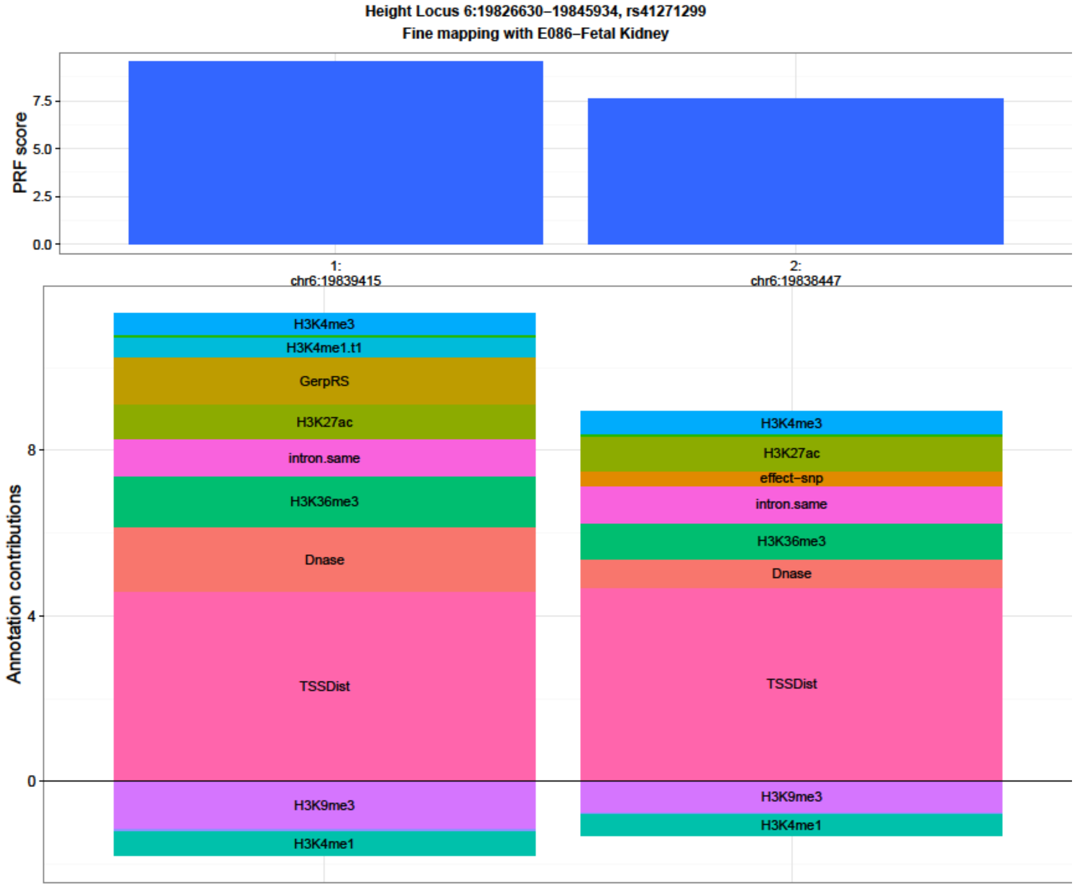
Height Locus 6:19826630–19845934, rs41271299
Fine mapping with E086–Fetal Kidney
PRF score
Annotation contributions
1:
chr6:19839415
2:
chr6:19838447
H3K4me3
H3K4me1.t1
GerpRS
H3K27ac
intron.same
H3K36me3
Dnase
TSSDist
H3K9me3
H3K4me1
effect–snp

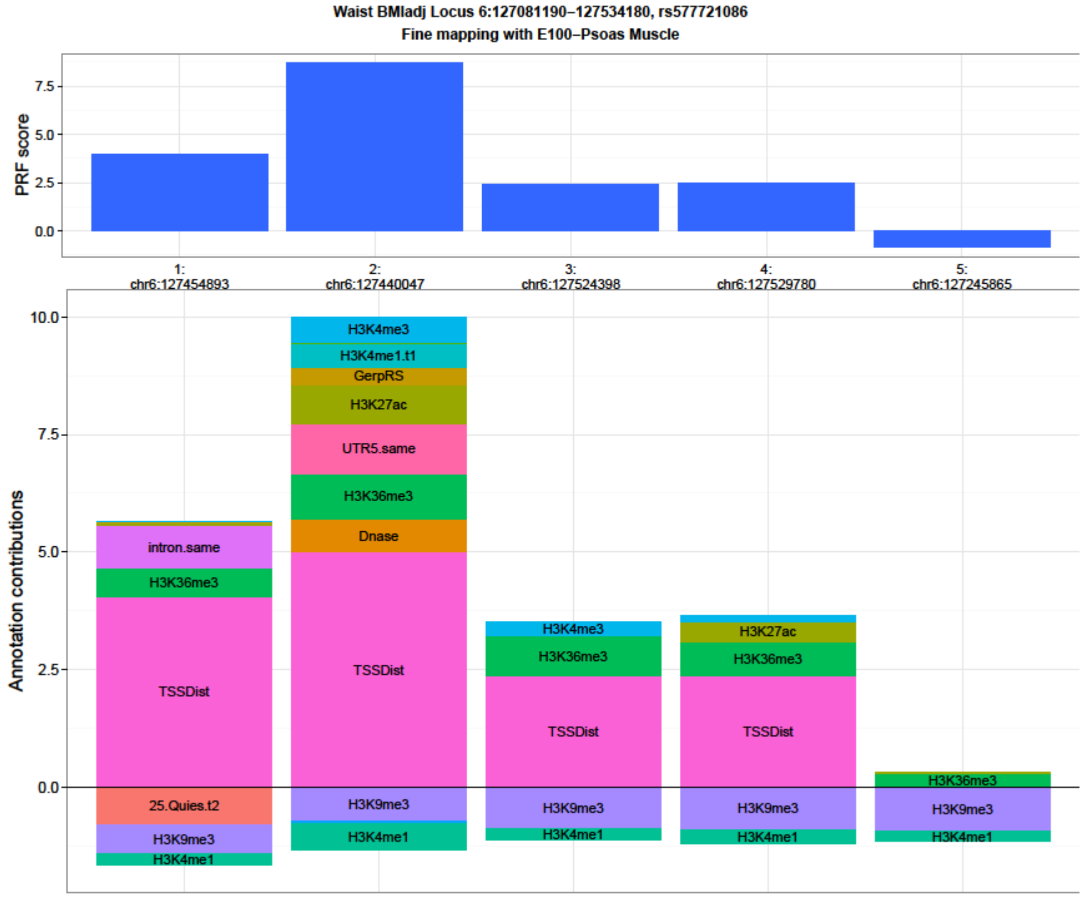
Waist BMIadj Locus 6:127081190–127534180, rs577721086
Fine mapping with E100–Psoas Muscle
PRF score
Annotation contributions
1:
chr6:127454893
2:
chr6:127440047
3:
chr6:127524398
4:
chr6:127529780
5:
chr6:127245865
intron.same
H3K36me3
TSSDist
25.Quies.t2
H3K9me3
H3K4me1
H3K4me3
H3K4me1.t1
GerpRS
H3K27ac
UTR5.same
Dnase

Height Locus 4:13281120–13749254, rs16888802
Fine mapping with E121–HSMM cell derived Skeletal Muscle Myotubes Cells

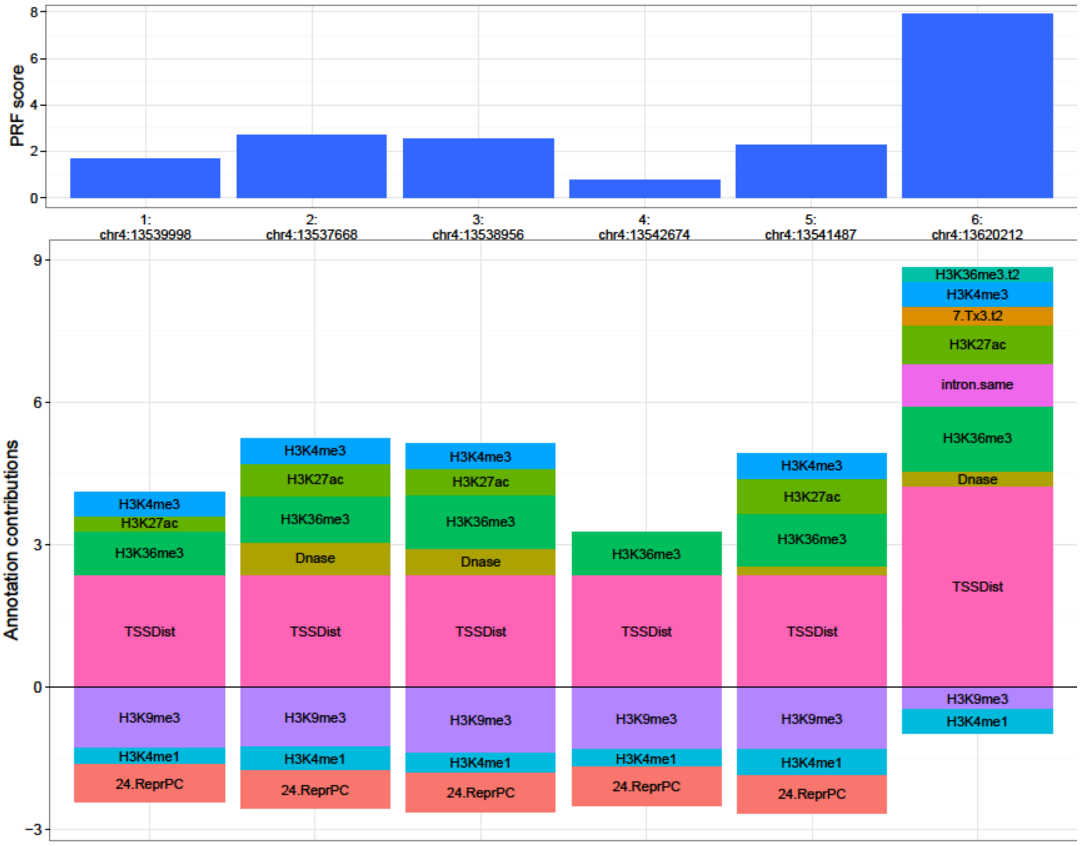

Height Locus 3:48394419–50532006, rs202238847
Fine mapping with E059–Foreskin Melanocyte Primary Cells skin01

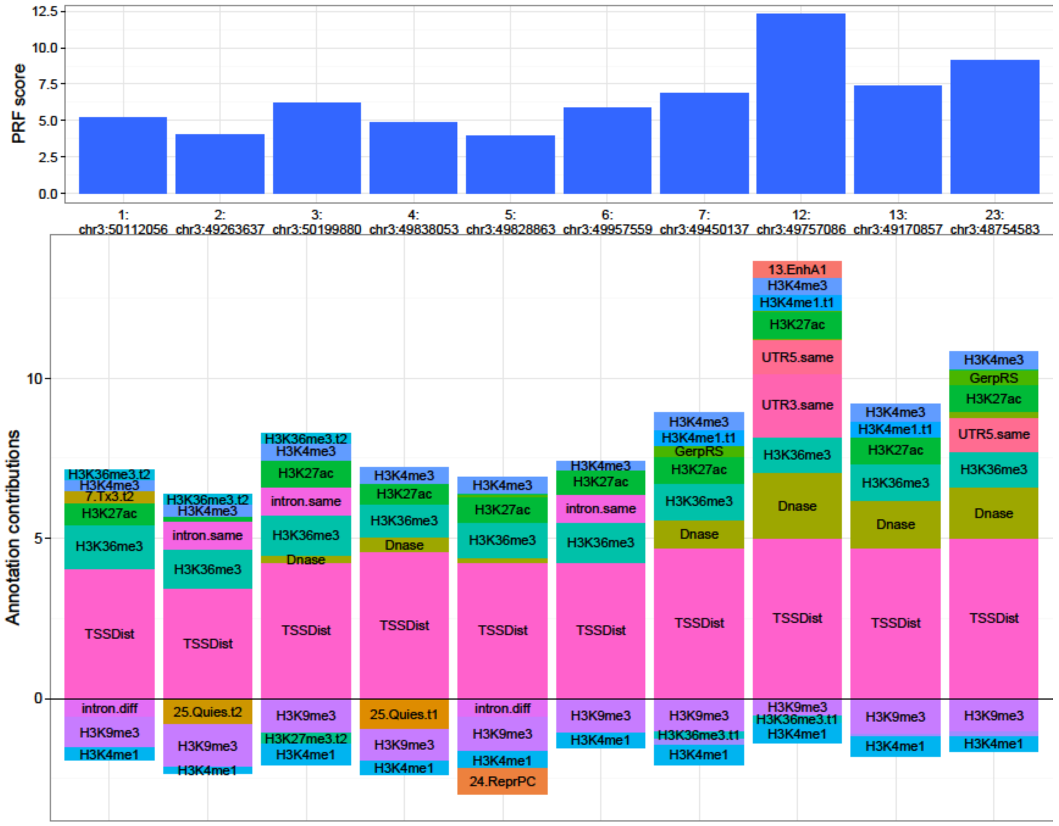

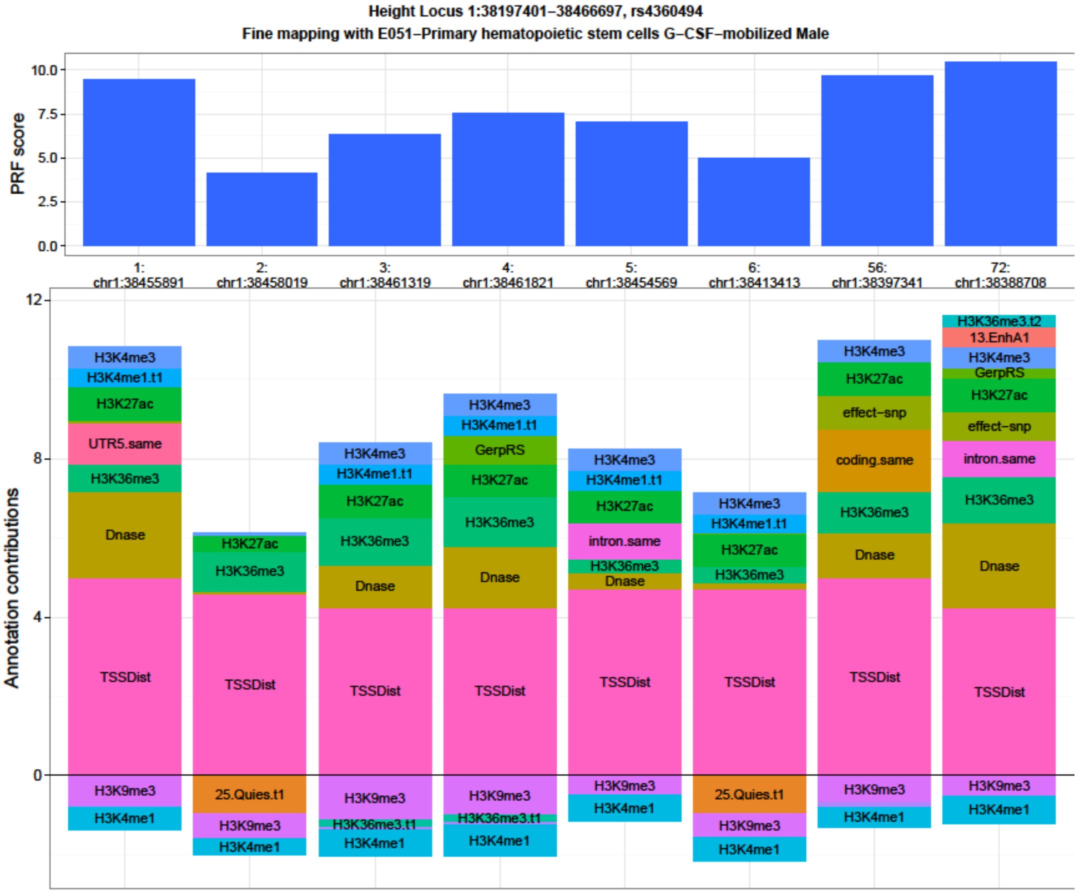
Height Locus 1:38197401–38466697, rs4360494
Fine mapping with E051–Primary hematopoietic stem cells G–CSF–mobilized Male
PRF score
Annotation contributions
1: chr1:38455891
2: chr1:38458019
3: chr1:38461319
4: chr1:38461821
5: chr1:38454569
6: chr1:38413413
56: chr1:38397341
72: chr1:38388708

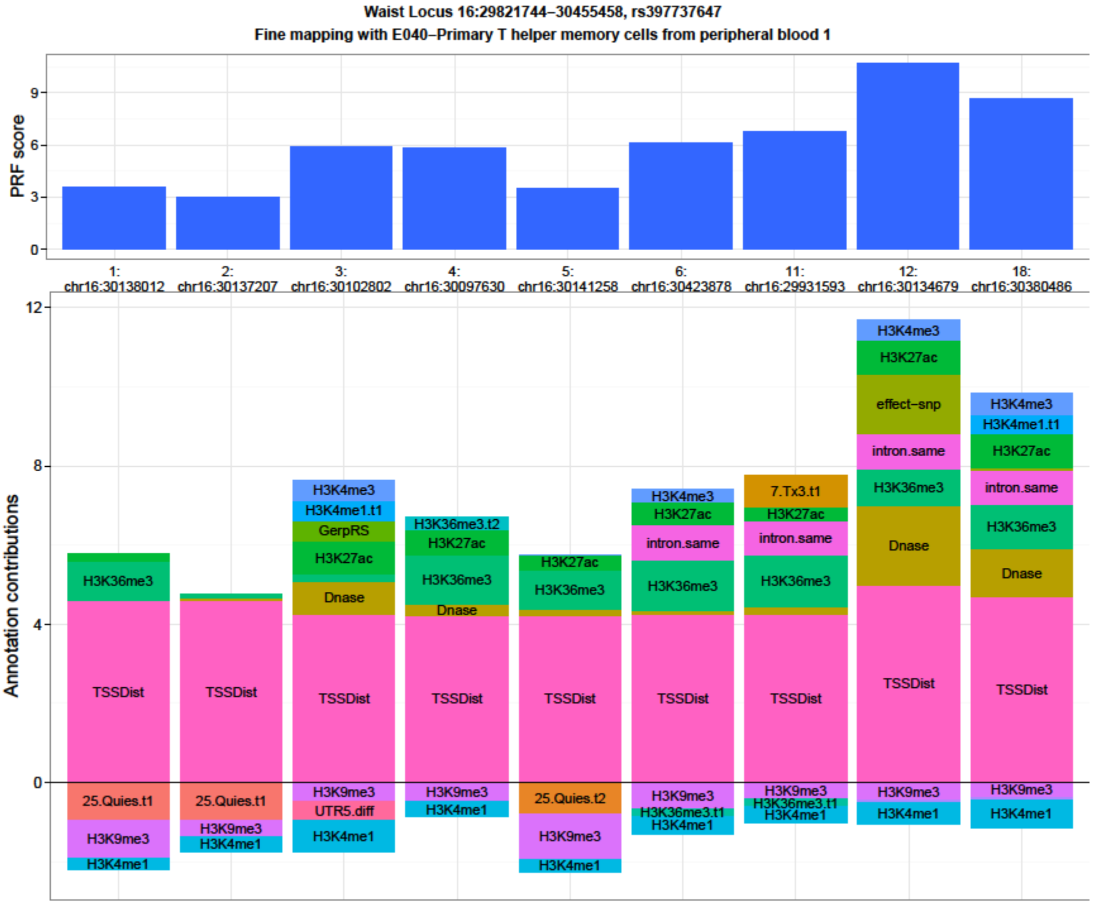
Waist Locus 16:29821744–30455458, rs397737647
Fine mapping with E040–Primary T helper memory cells from peripheral blood 1
PRF score
Annotation contributions
1: chr16:30138012
2: chr16:30137207
3: chr16:30102802
4: chr16:30097630
5: chr16:30141258
6: chr16:30423878
11: chr16:29931593
12: chr16:30134679
18: chr16:30380486

Hip BMIadj Locus 2:71334122–71703945, rs4852777
Fine mapping with E066–Liver

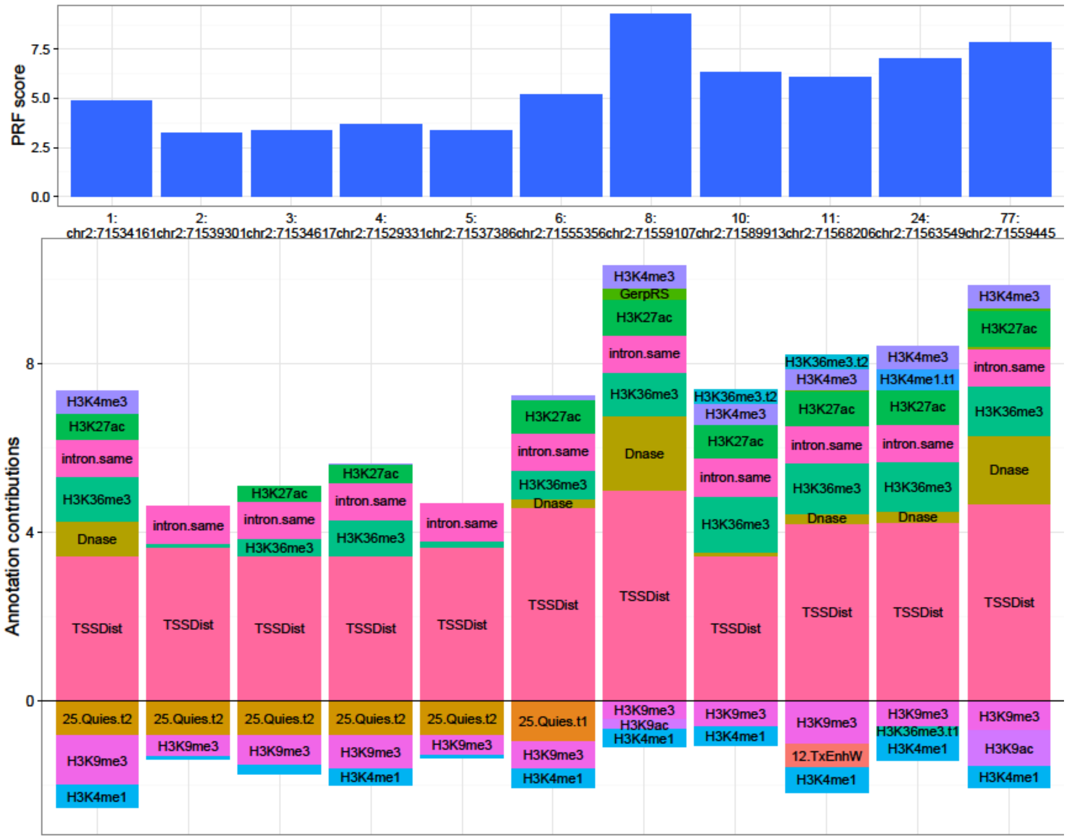

Weight Locus 7:92057968–92563344, rs42377
Fine mapping with E006–H1 Derived Mesenchymal Stem Cells

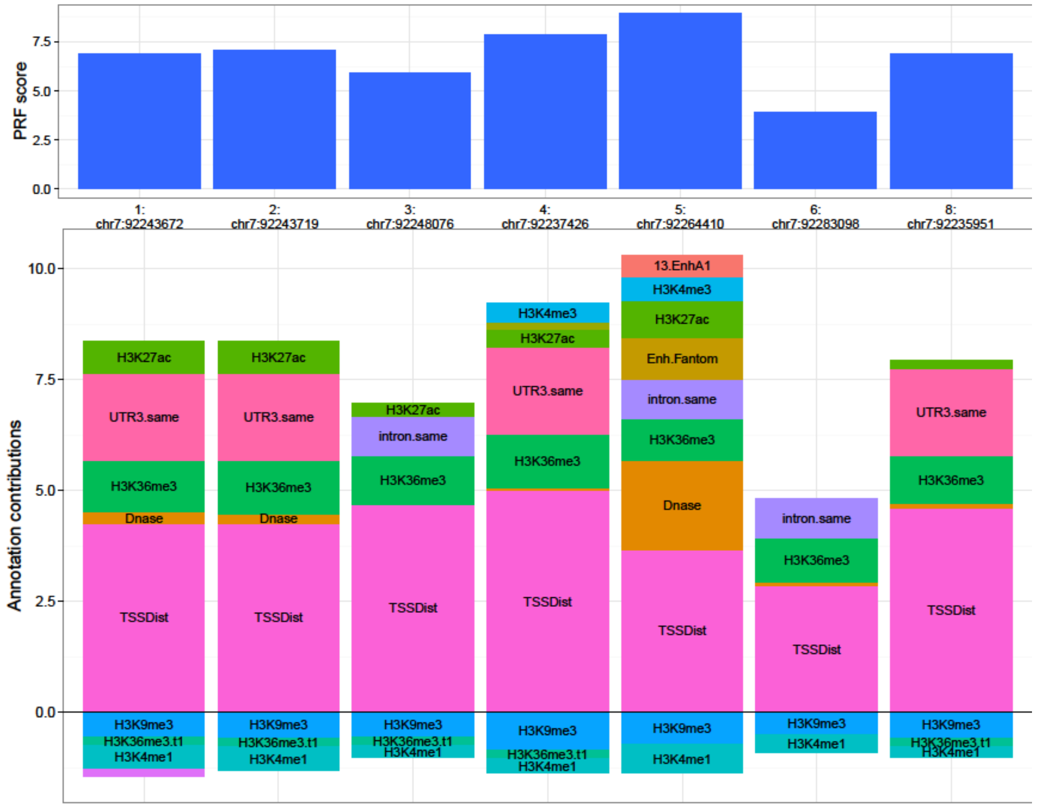

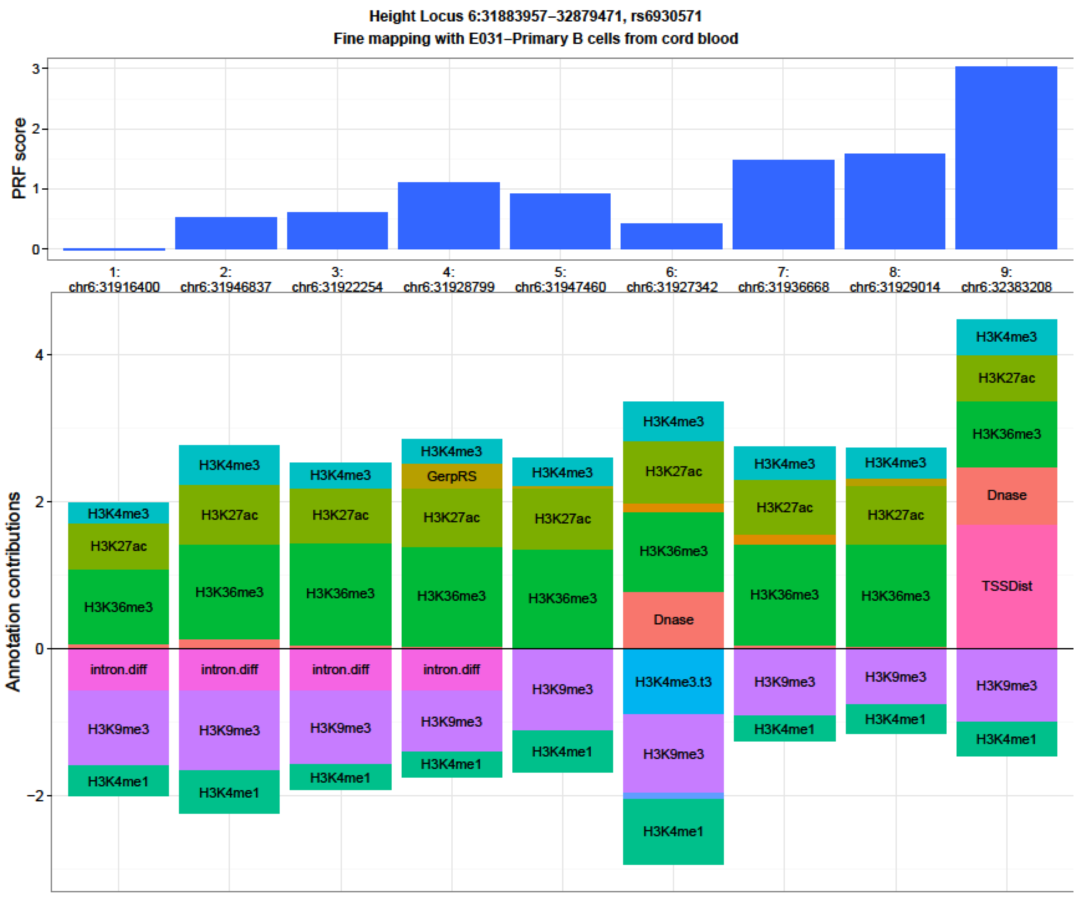

Figure S15: Probability of regulatory function (PRF) plots for the newly identified loci.
For each of the newly identified loci, we attempted to fine-map the association signal using epigenomic annotations (Methods). At each of the regions around the loci, the plots show the PRF scores for variants with high posterior probability of association, after combining association statistics with the PRF scores. Each variant is labeled with its association statistic rank, followed by the variant position (e.g. 1: chr2:407713). The height of the blue bar represents the PRF score itself, which is the sum of the contributions from individual annotations shown below. Annotations with negative enrichments (depletion of causal variants, e.g. H3K9me3) appear below zero in the PRF score breakdown. Regions with high confidence in fine-mapping are shown below (posterior probability of causality for the best SNP > 0.5).

A)

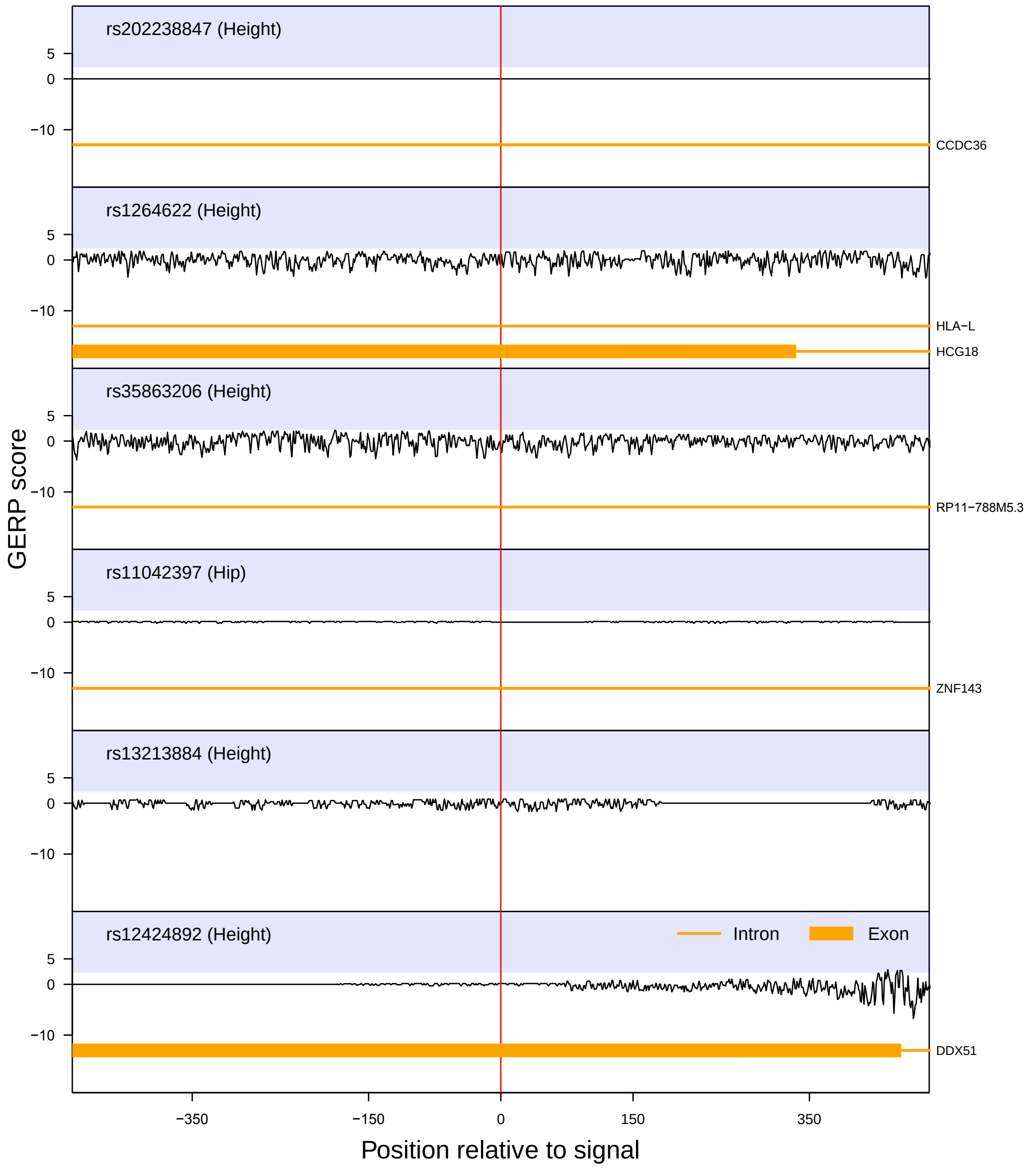

rs202238847 (Height)
CCDC36
rs1264622 (Height)
HLA−L
HCG18
rs35863206 (Height)
RP11−788M5.3
rs11042397 (Hip)
ZNF143
rs13213884 (Height)
rs12424892 (Height)
Intron
Exon
DDX51
GERP score
5
0
−10
−350
−150
0
150
350
Position relative to signal

B)

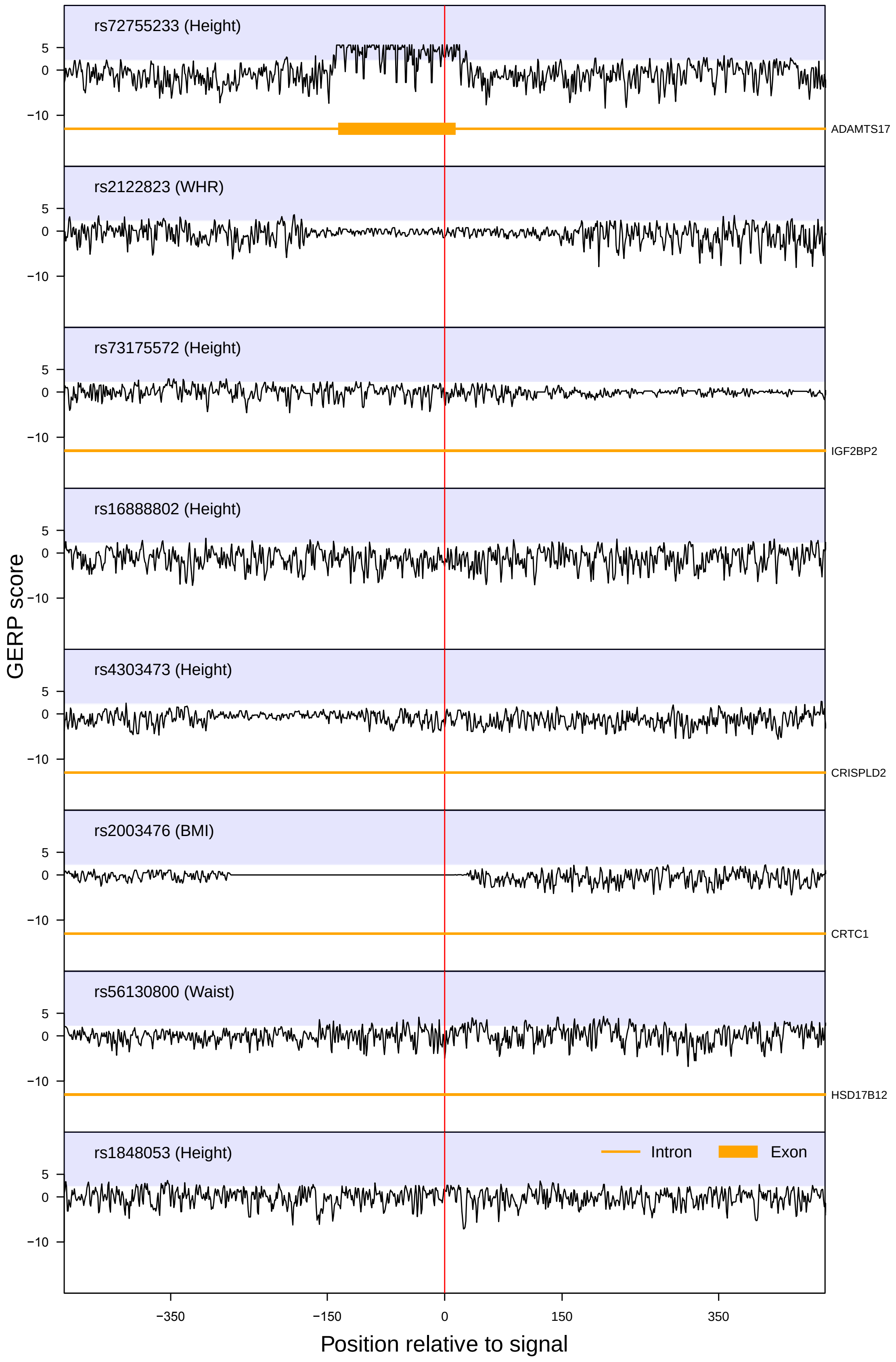

rs72755233 (Height)
ADAMTS17
rs2122823 (WHR)
rs73175572 (Height)
IGF2BP2
rs16888802 (Height)
rs4303473 (Height)
CRISPLD2
rs2003476 (BMI)
CRTC1
rs56130800 (Waist)
HSD17B12
rs1848053 (Height)
Intron
Exon
5
0
−10
GERP score
−350
−150
0
150
350
Position relative to signal

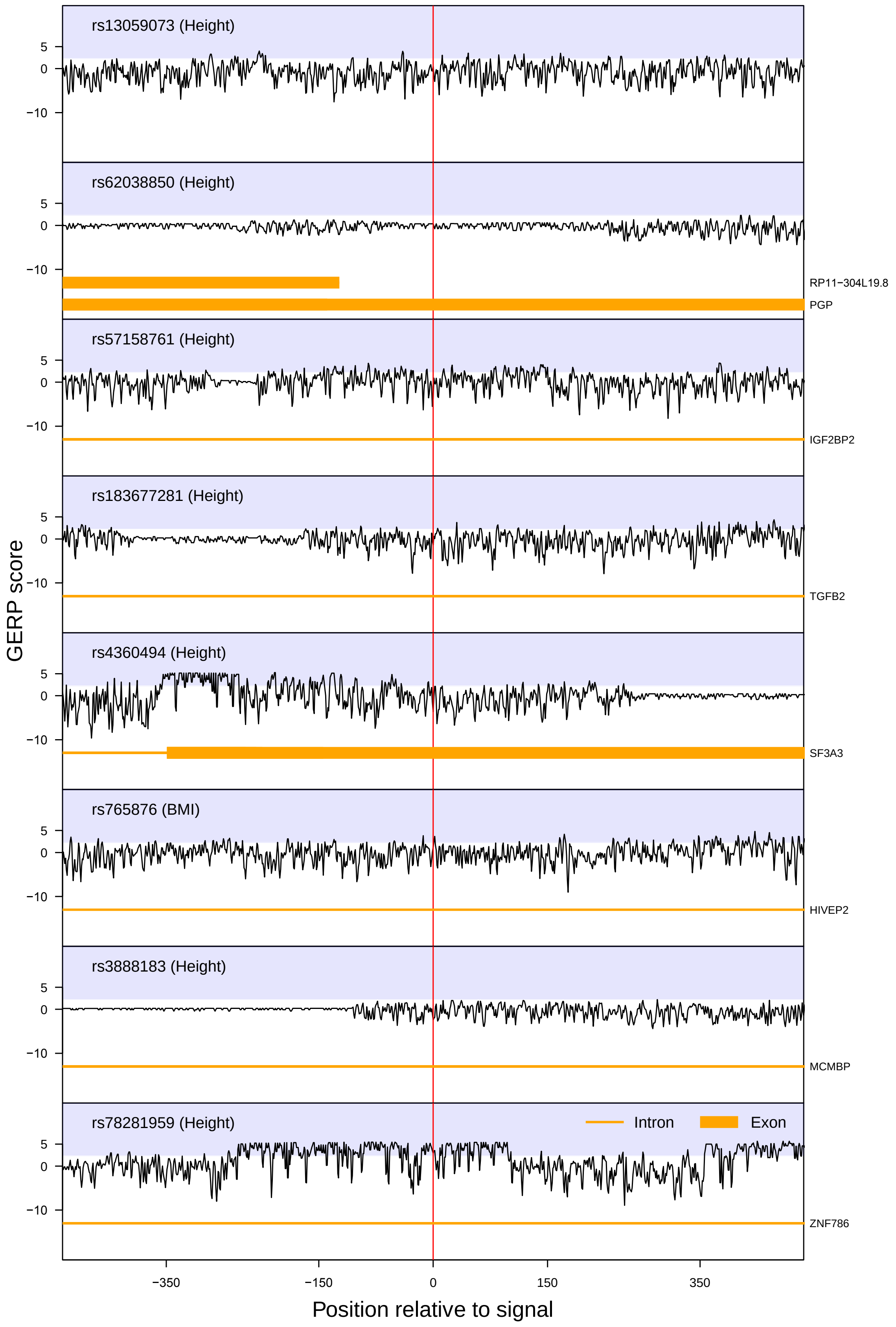

rs13059073 (Height)
rs62038850 (Height)
rs57158761 (Height)
rs183677281 (Height)
rs4360494 (Height)
rs765876 (BMI)
rs3888183 (Height)
rs78281959 (Height)
RP11−304L19.8
PGP
IGF2BP2
TGFB2
SF3A3
HIVEP2
MCMBP
ZNF786
Intron
Exon
GERP score
5
0
−10
−350
−150
0
150
350
Position relative to signal

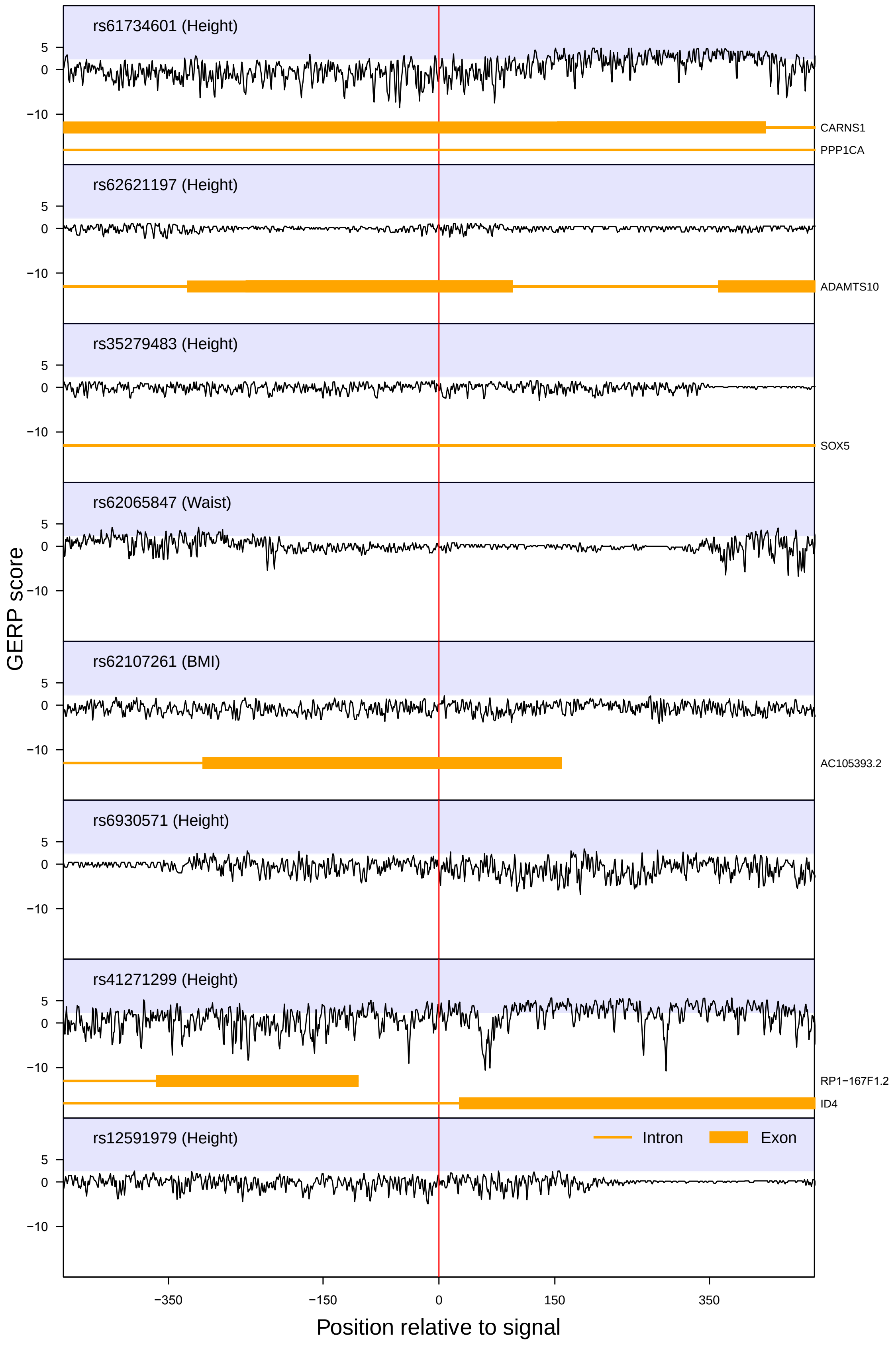
rs61734601 (Height)
CARNS1
PPP1CA
rs62621197 (Height)
ADAMTS10
rs35279483 (Height)
SOX5
rs62065847 (Waist)
rs62107261 (BMI)
AC105393.2
rs6930571 (Height)
rs41271299 (Height)
RP1−167F1.2
ID4
rs12591979 (Height)
Intron
Exon
5
0
−10
GERP score
−350
−150
0
150
350
Position relative to signal

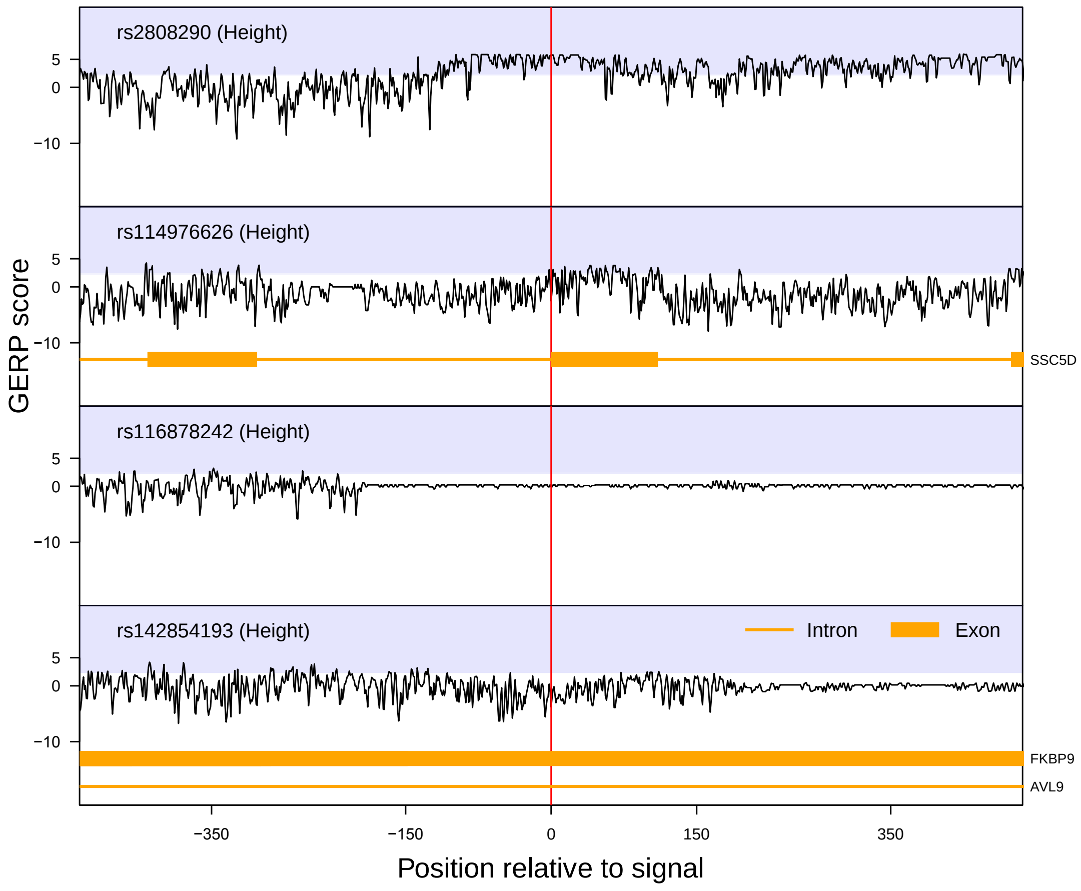

Figure S16: Genomic Evolutionary Rate Profiling (GERP) score as a measure of cross-species conservation of the sequences around each newly identified association.

A – variants listed in Table 1; B – variants listed in Table 2. The GERP score is based on the analysis of the alignment of sequences from 29 mammalian species and captures substitution deficits indicating sequence conservation. A score above zero indicates substitution deficit and thus indicates that a site may be under evolutionary constraint. Negative scores indicate substitution surplus. Stretches of scores close to zero indicate regions where the alignment is too shallow to get a meaningful estimate of the constraint. In practice, a position with GERP score above two is considered to be conserved (this threshold is indicated by the light blue background on the plots). To put the conservation pattern in a genic context, the transcripts of genes located within 500bp of the signals are also shown (annotation from GENCODE release 19). To make the trends in conservation more visible in the plots, GERP scores were averaged in two base pairs long sliding window. The red line indicates the position of the variant.

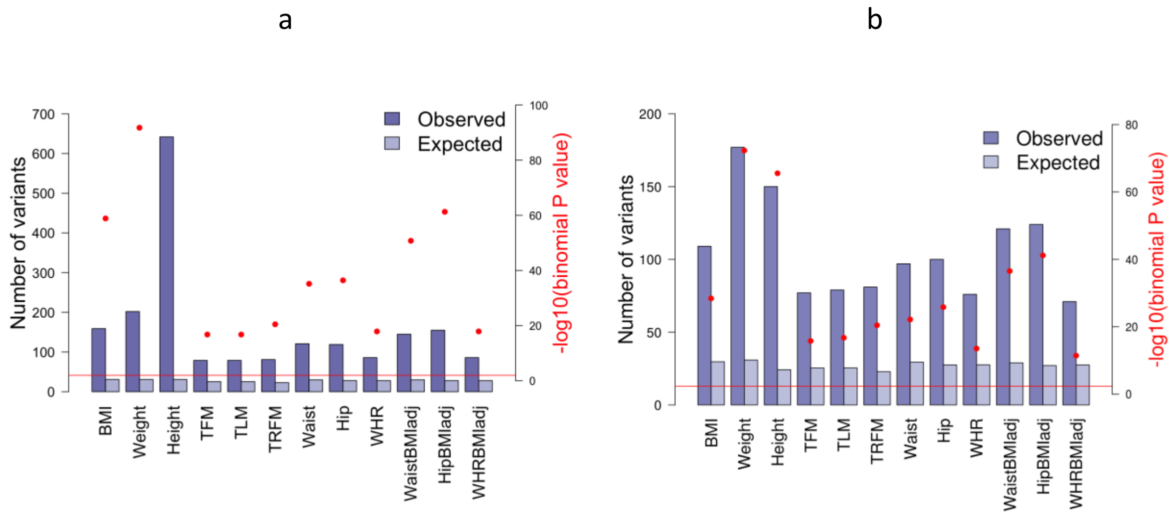

Figure S17: Enrichment in discovery meta-analysis using independent variants (r^2<0.2) with MAF ≥ 0.1% (a) and after excluding previously known loci (±500kb) (b).
Enrichment of signal is observed if the *P-value* (one-sided, denoted by the red dot) from the binomial test of observed versus expected number of variants with $P \leq 10^{-5}$ is less than 0.05/4.482 (5% significance level Bonferroni corrected for the effective number of independent traits; horizontal red line). The enrichment in height using all variants in (a) is too significant to be calculated with precision (~3M independent variants with MAF≥0.1%, 642 of which have $P \leq 10^{-5}$, 31 expected). Observed and expected counts, Bonferroni corrected *P-values* and FDR *q-values* are given in Table S11.
WaistBMIadj: waist circumference adjusted for BMI; HipBMIadj: hip circumference adjusted for BMI; WHRBMIadj: waist to hip ratio adjusted for BMI; TFM: total fat mass; TLM: total lean mass; TRFM: trunk fat mass.

All	Novel
0.1%≤MAF≤1%	
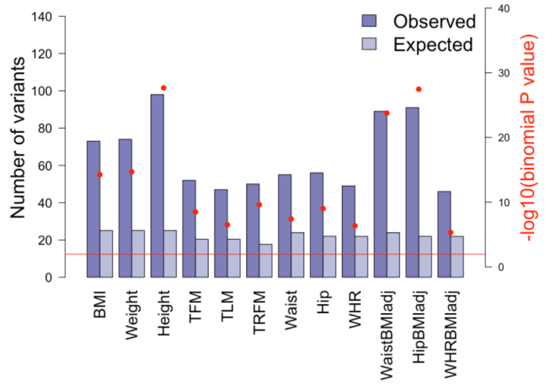	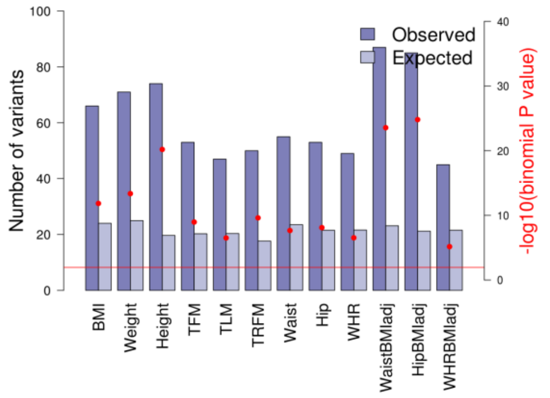
1%<MAF≤5%	
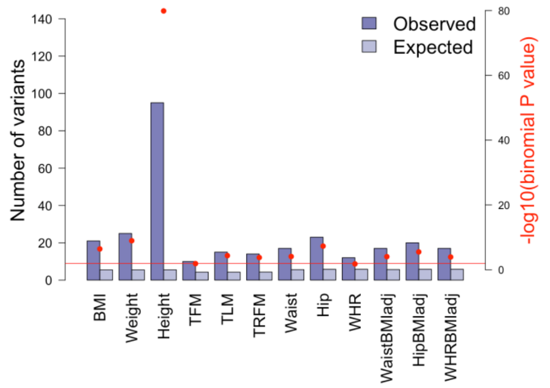	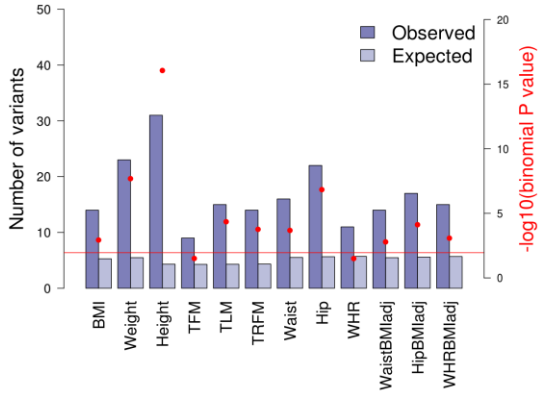
MAF>5%	
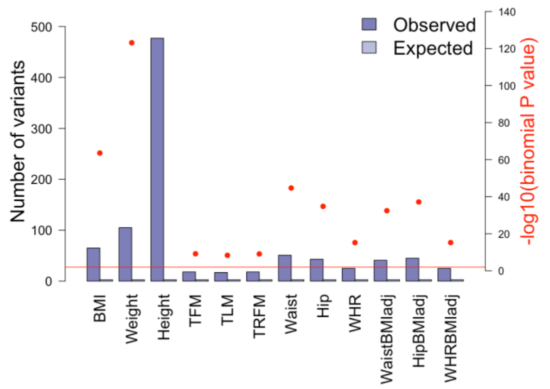	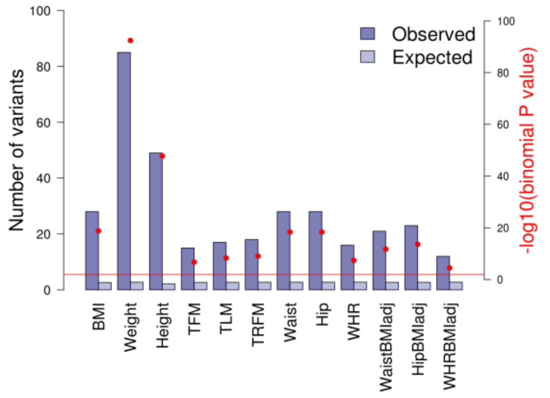

BMI: body mass index; WHR: waist to hip ratio; WaistBMIadj: waist circumference adjusted for BMI; HipBMIadj: hip circumference adjusted for BMI; WHRBMIadj: waist to hip ratio adjusted for BMI; TFM: total fat mass; TLM: total lean mass; TRFM: trunk fat mass

Figure S18: Enrichment in discovery meta-analysis results. Using independent variants (r^2<0.2) within different Minor Allele Frequency (MAF) bins (left) and after excluding previously known loci (±500 kb) (right). Enrichment of signal is observed if the *P-value* from the binomial test of observed versus expected number of variants with P≤10^{-5} is less than 0.05/4.482 (5% significance level Bonferroni corrected for the effective number of independent traits (horizontal red line).

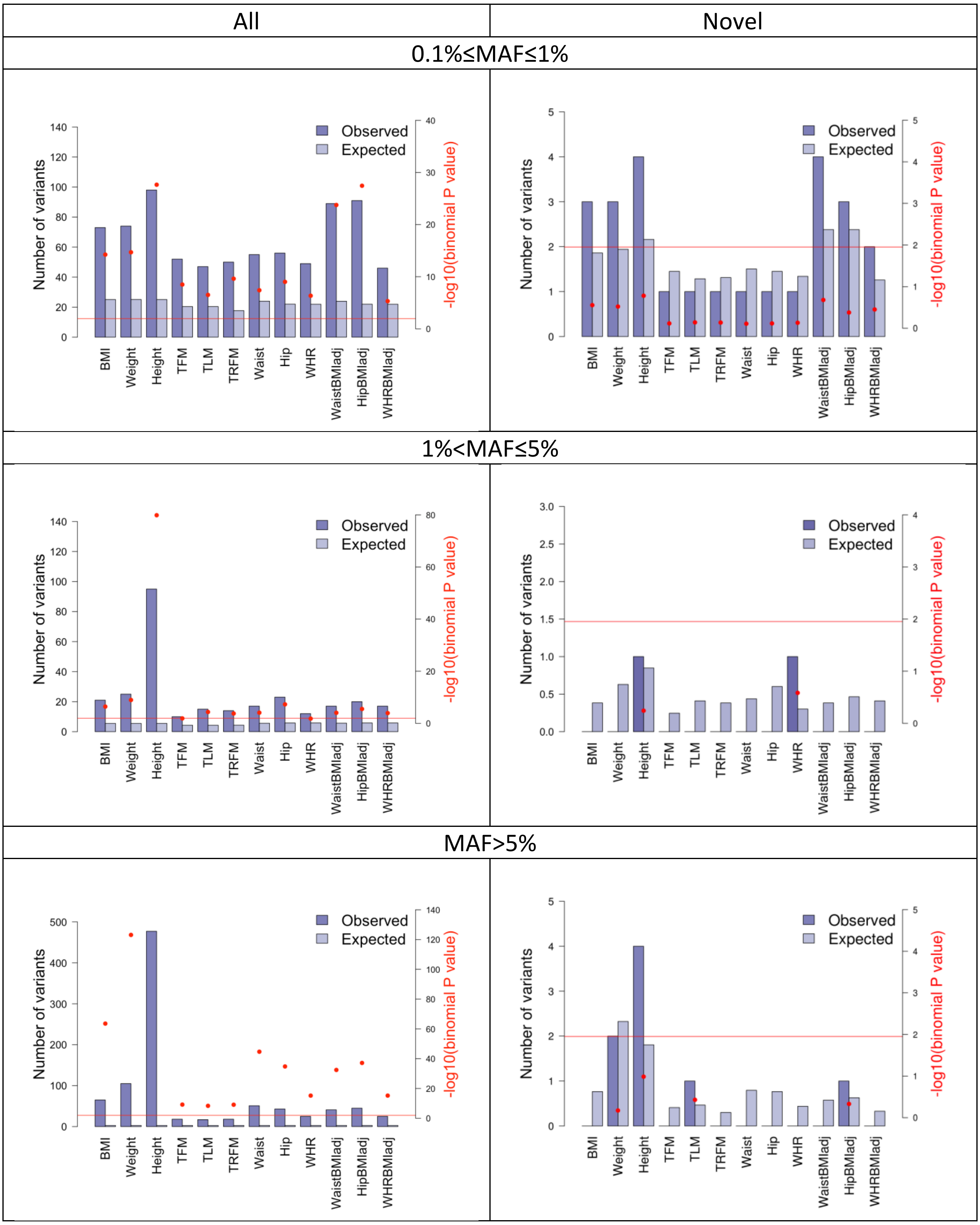

BMI: body mass index; WHR: waist to hip ratio; WaistBMIadj: waist circumference circumference adjusted for BMI; HipBMIadj: hip circumference adjusted for BMI; WHRBMIadj: waist to hip ratio adjusted for BMI; TFM: total fat mass; TLM: total lean mass; TRFM: trunk fat mass

Figure S19: Enrichment of discovery meta-analysis results in Mendelian genes for height.
We used independent variants ($r^2<0.2$) within different Minor Allele Frequency (MAF) bins (left) and after excluding previously known loci (±500 kb) (right). Enrichment of signal is observed if the *P-value* from the binomial test of observed versus expected number of variants with $P \leq 10^{-5}$ in Mendelian genes for height (as calculated by GREAT) is less than 0.05/4.482 (5% significance level Bonferroni corrected for the effective number of independent traits (horizontal red line).

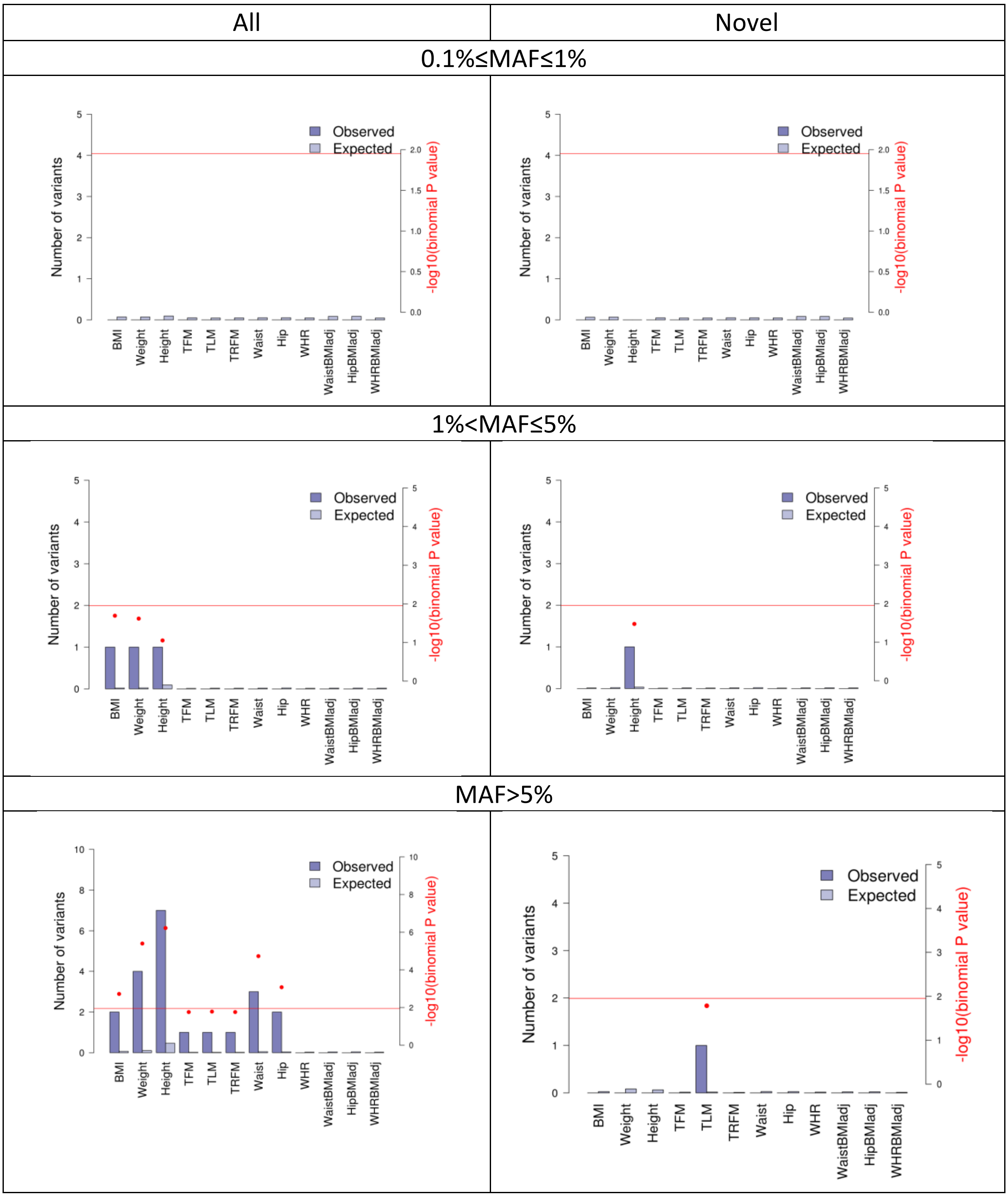

BMI: body mass index; WHR: waist to hip ratio; WaistBMIadj: waist circumference adjusted for BMI; HipBMIadj: hip circumference adjusted for BMI; WHRBMIadj: waist to hip ratio adjusted for BMI; TFM: total fat mass; TLM: total lean mass; TRFM: trunk fat mass

Figure S20: Enrichment of discovery meta-analysis results in monogenic obesity genes. We used independent variants (r^2<0.2) within different Minor Allele Frequency (MAF) bins (left) and after excluding previously known loci (±500 kb) (right). Enrichment of signal is observed if the *P-value* from the binomial test of observed versus expected number of variants with $P \leq 10^{-5}$ in Mendelian genes for obesity (as calculated by GREAT) is less than 0.05/4.482 (5% significance level Bonferroni corrected for the effective number of independent traits (horizontal red line).

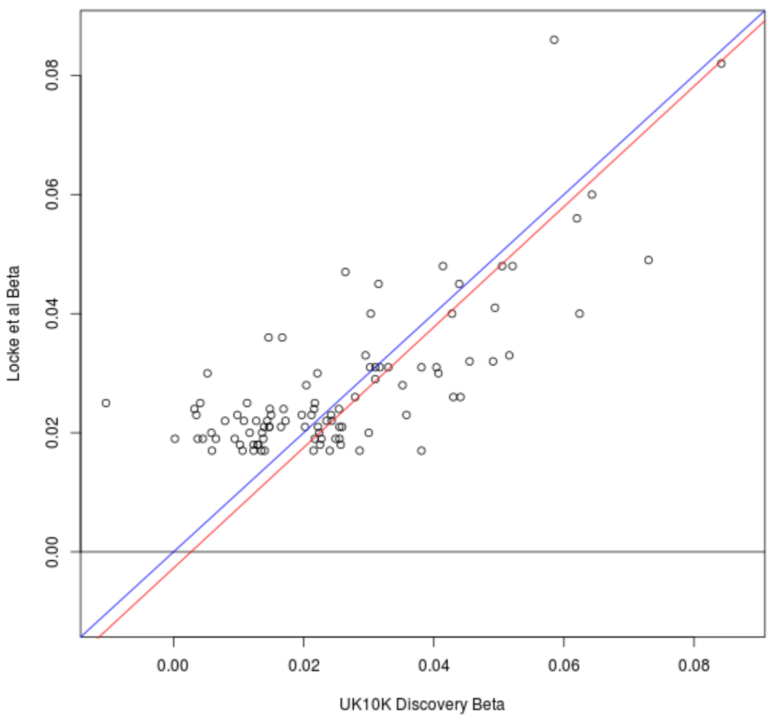

0.00
0.02
0.04
0.06
0.08
Locke et al Beta
UK10K Discovery Beta

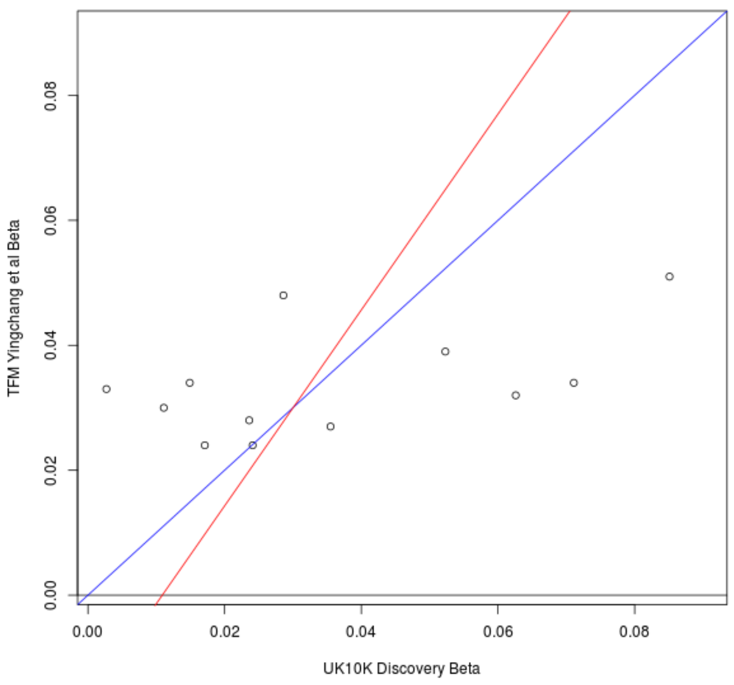

0.00
0.02
0.04
0.06
0.08
TFM Yingchang et al Beta
UK10K Discovery Beta

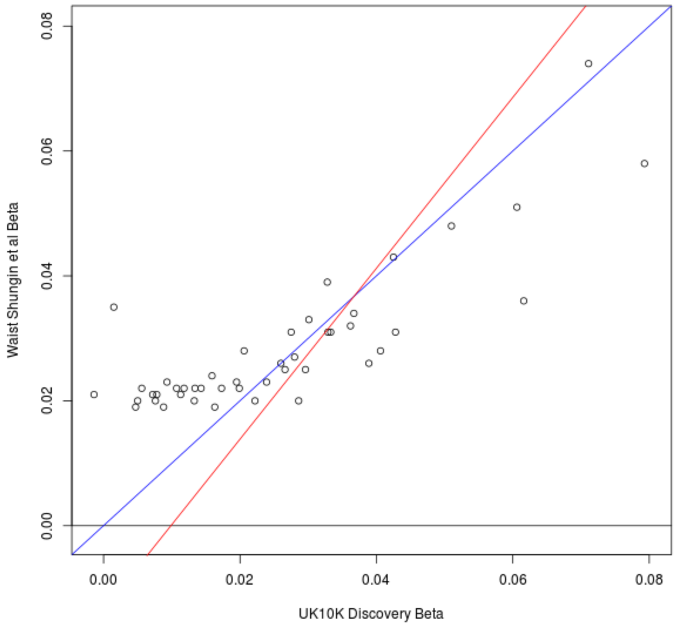

Waist Shungin et al Beta
UK10K Discovery Beta
0.00
0.02
0.04
0.06
0.08

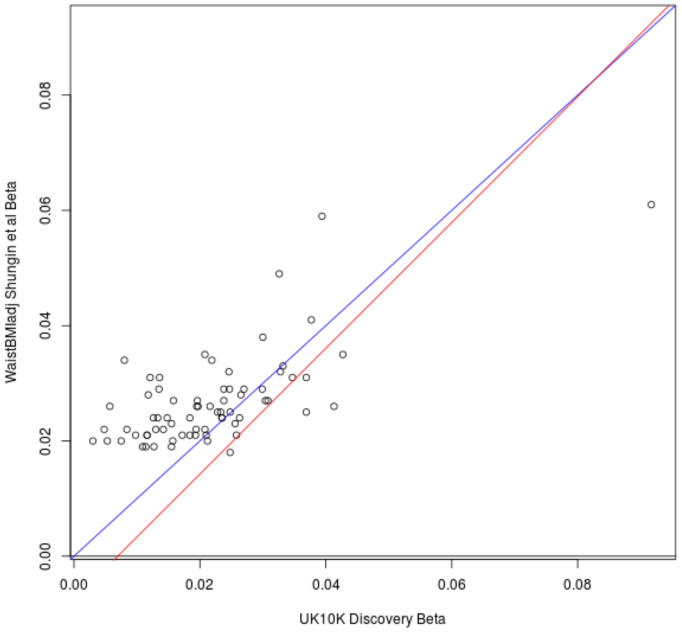

WaistBMIadj Shungin et al Beta
UK10K Discovery Beta
0.00
0.02
0.04
0.06
0.08

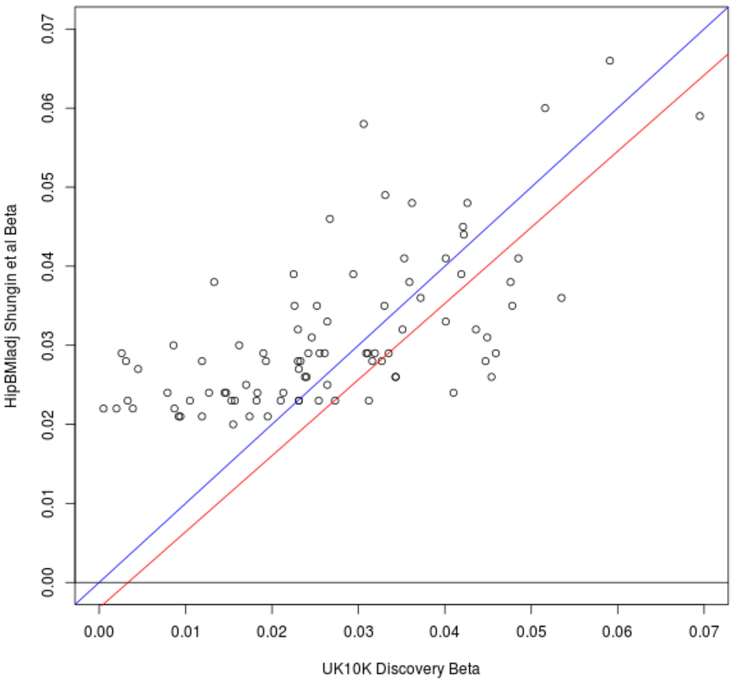
HipBMIadj Shungin et al Beta
UK10K Discovery Beta
0.00
0.01
0.02
0.03
0.04
0.05
0.06
0.07

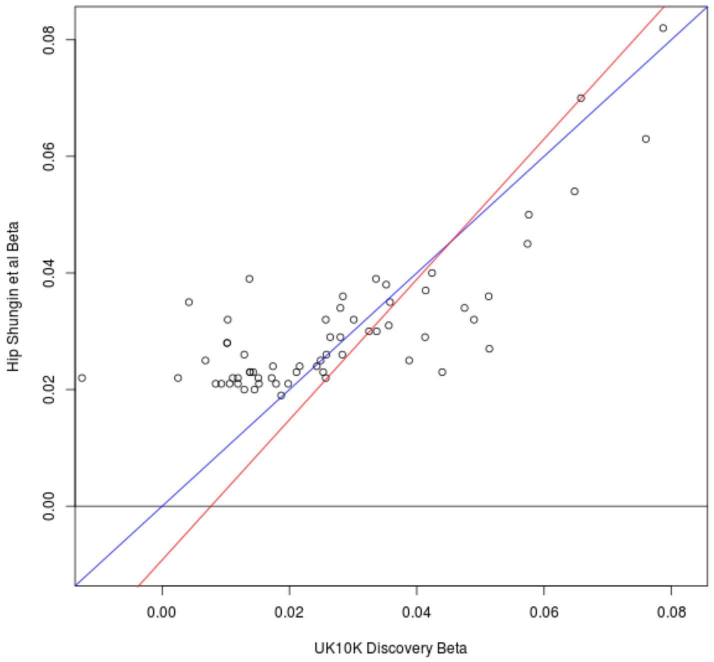
Hip Shungin et al Beta
UK10K Discovery Beta
0.00
0.02
0.04
0.06
0.08

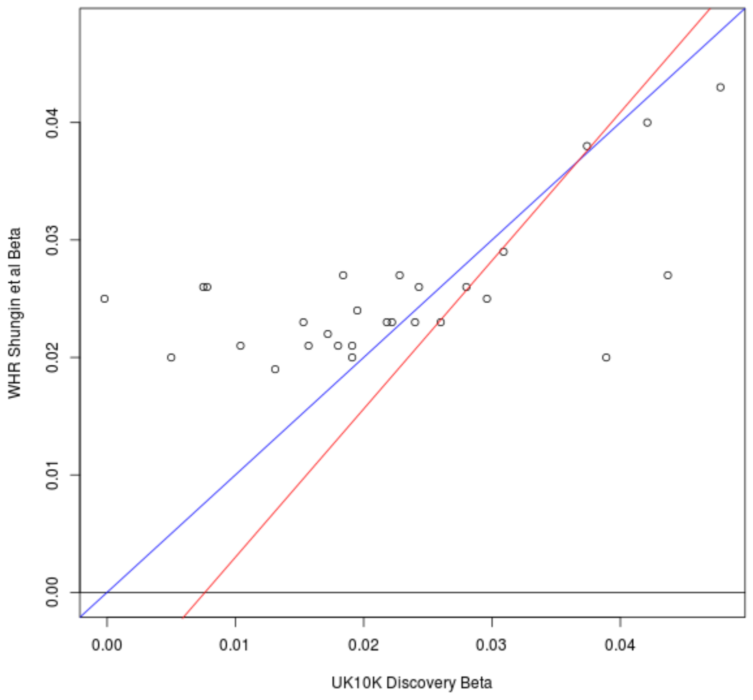

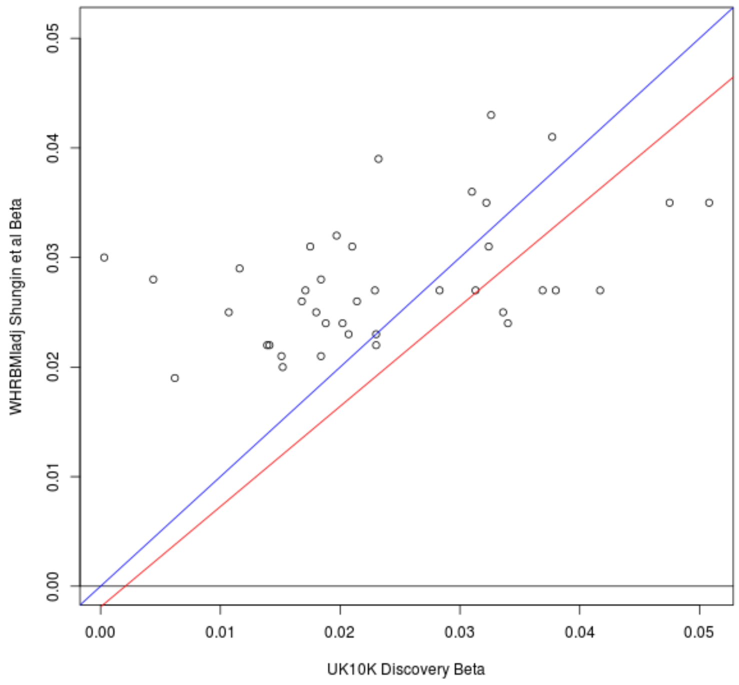

Figure S21: Beta-beta plots.
Effect sizes in our discovery phase (x-axis) versus effect sizes of previously published associations (y-axis) for variants associated with: A. BMI from Locke et al, R-squared: 0.563; B. TFM from Lu et al, R-squared: 0.267; C. waist circumference from Shungin et al, R-squared: 0.701; D. waist circumference adjusted for BMI from Shungin et al, R-squared: 0.489; E. hip circumference from Shungin et al, R-squared: 0.623; F. hip circumference adjusted for BMI from Shungin et al, R-squared: 0.427; G. WHR from Shungin et al, R-squared: 0.397; H. WHR adjusted for BMI from Shungin et al, R-squared: 0.215. The blue line is drawn at y=x. The red line is the observed correlation coefficient of x and y.

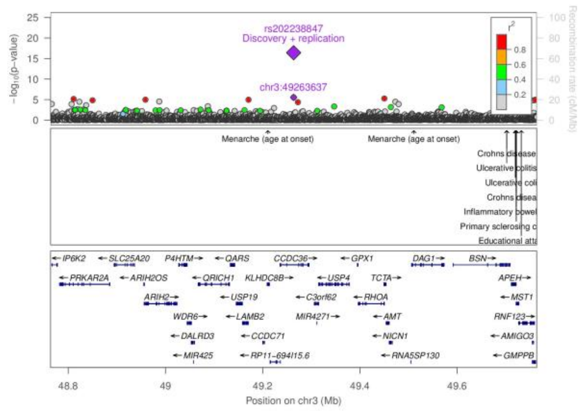

Cohort	EAF	Beta	P-value	N	Weight
ALSPAC WGS	0.024	0.023	8.27e-01	1794	89.59
TwinsUK WGS	0.021	0.299	1.17e-02	1747	71.15
arcOGEN	0.02	-0.006	9.54e-01	2748	103.09
UKHLS	0.021	0.014	7.95e-01	8700	330.98
FINRISK	0.008	0.658	2.95e-03	1249	20.68
ALSPAC GWA	0.022	0.039	6.17e-01	4103	165.71
TwinsUK GWA	0.021	0.215	5.82e-02	3540	77.89
1958 Birth Cohort	0.021	0.214	1.44e-03	5721	221.97
INGI Carlantino	0.038	0.083	6.83e-01	471	25.41
INGI Friuli Venezia Giulia	0.03	0.246	5.55e-02	1197	60.95
HELIC MANOLIS	0.009	0.186	4.77e-01	1058	14.54
HELIC Pomak	0.004	-0.295	4.11e-01	949	7.76
INCIPE 1	0.017	0.082	6.56e-01	937	29.52
INCIPE 2	0.012	0.35	1.79e-02	2057	45.87
LURIC	0.022	-0.029	8.32e-01	1427	54.11
Rotterdam 1	0.021	0.089	1.86e-01	5961	219.15
Rotterdam 2	0.025	0.195	5.45e-02	2151	97.78
Rotterdam 3	0.022	0.054	5.58e-01	3018	119.3
TEENAGE	0.018	0.257	2.21e-01	703	22.72
INGI Val Borbera	0.021	0.063	6.16e-01	1778	63.54
UK BioBank	0.023	0.091	2.05e-12	134797	5994.75
**Overall**	**0.022**	**0.095**	**3.76e-17**	**186106**	**7831.47**

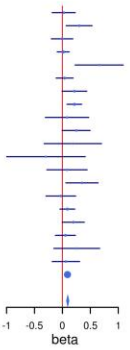

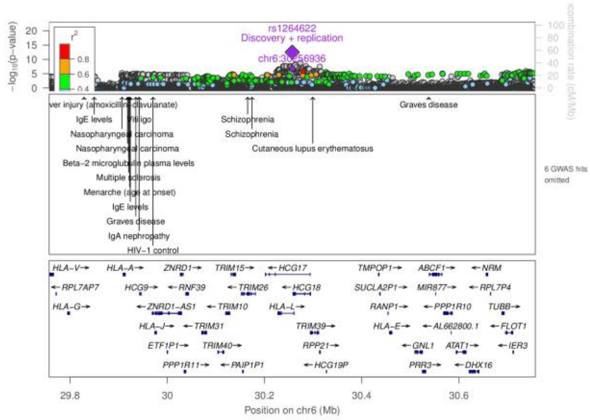

Cohort	EAF	Beta	P-value	N	Weight
ALSPAC WGS	0.198	-0.002	9.51e-01	1794	599.58
TwinsUK WGS	0.211	-0.016	6.97e-01	1747	580.58
arcOGEN	0.194	0.064	5.80e-02	2748	868.62
UKHLS	0.2	0.025	1.91e-01	8700	2787.37
FINRISK	0.138	0.118	5.15e-02	1249	276.17
ALSPAC GWA	0.205	0.093	7.31e-04	4103	1332.66
TwinsUK GWA	0.206	0.035	3.51e-01	3540	695.82
1958 Birth Cohort	0.199	0.02	3.99e-01	5721	1848.02
INGI Carlantino	0.092	0.204	8.29e-02	471	72.8
INGI Friuli Venezia Giulia	0.112	-0.02	7.90e-01	1197	185.26
HELIC MANOLIS	0.13	0.086	2.20e-01	1058	200.8
HELIC Pomak	0.155	-0.035	6.18e-01	949	194.02
INCIPE 2	0.167	0.094	2.55e-02	2057	565.44
LURIC	0.177	0.01	8.36e-01	1427	416.03
Rotterdam 1	0.188	0.073	1.50e-02	5961	1118.18
Rotterdam 2	0.198	0.024	6.20e-01	2151	431.94
Rotterdam 3	0.186	0.094	2.55e-02	3018	561.85
TEENAGE	0.127	0.07	3.86e-01	703	155.59
INGI Val Borbera	0.153	0.111	4.92e-02	1778	311.54
UK BioBank	0.201	0.026	4.62e-08	134797	45102.42
**Overall**	**0.199**	**0.03**	**3.05e-13**	**185169**	**59488.4**

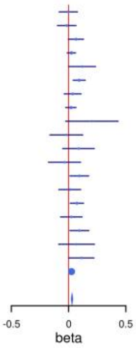

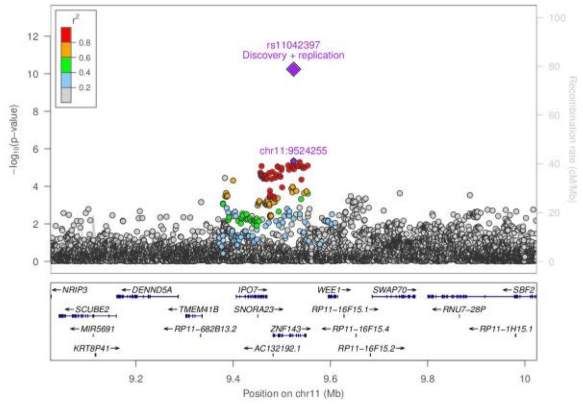

Cohort	EAF	Beta	P-value	N	Weight
ALSPAC WGS	0.457	0.069	3.66e-02	1808	918.66
TwinsUK WGS	0.455	-0.011	7.76e-01	1266	638.5
FINRISK	0.32	-0.025	5.59e-01	1254	530.94
ALSPAC GWA	0.451	0.047	3.43e-02	4115	2059.74
TwinsUK GWA	0.452	0.065	7.06e-02	2582	765.84
1958 Birth Cohort	0.445	0.035	6.40e-02	5700	2763.97
INGI Carlantino	0.576	-0.01	9.00e-01	400	164.39
INGI Friuli Venezia Giulia	0.548	0.062	2.58e-01	701	335.98
HELIC MANOLIS	0.557	0.024	6.06e-01	1065	474.98
HELIC Pomak	0.514	0.054	2.83e-01	899	389.44
LURIC	0.461	0.037	3.31e-01	1404	699.91
Rotterdam 1	0.445	0.059	1.97e-03	5660	2797.4
Rotterdam 2	0.446	0.032	3.24e-01	1937	953.13
Rotterdam 3	0.452	-0.009	7.34e-01	2931	1378.68
TEENAGE	0.549	0.063	2.56e-01	701	329.16
UK BioBank	0.444	0.02	4.11e-07	134650	66203.42
**Overall**	**0.446**	**0.023**	**5.74e-11**	**167073**	**81632.65**

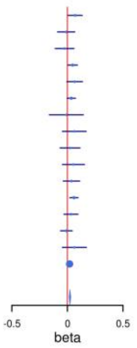

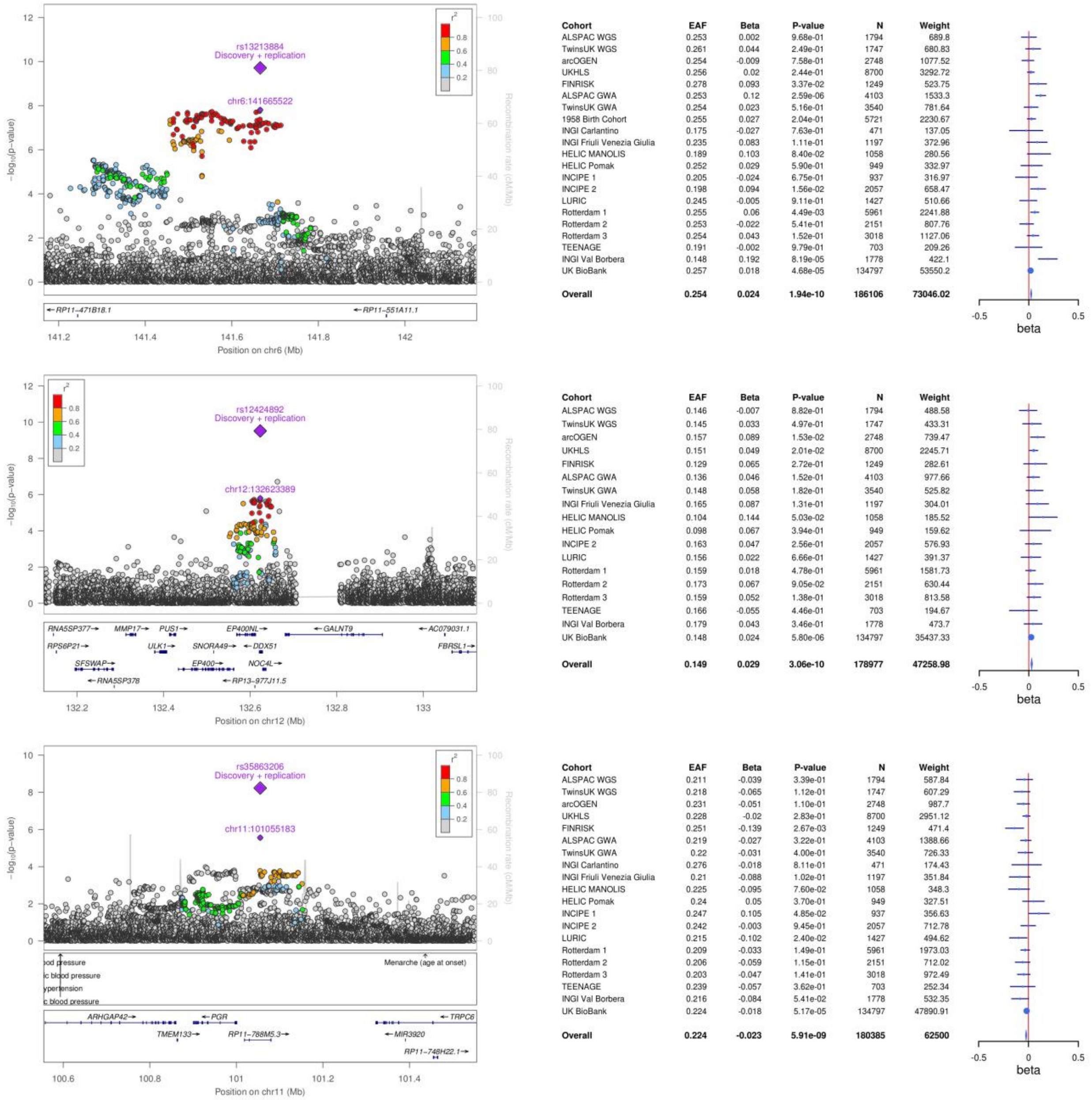

Cohort	EAF	Beta	P-value	N	Weight
ALSPAC WGS	0.253	0.002	9.68e-01	1794	689.8
TwinsUK WGS	0.261	0.044	2.49e-01	1747	680.83
arcOGEN	0.254	-0.009	7.58e-01	2748	1077.52
UKHLS	0.256	0.02	2.44e-01	8700	3292.72
FINRISK	0.278	0.093	3.37e-02	1249	523.75
ALSPAC GWA	0.253	0.12	2.59e-06	4103	1533.3
TwinsUK GWA	0.254	0.023	5.16e-01	3540	781.64
1958 Birth Cohort	0.255	0.027	2.04e-01	5721	2230.67
INGI Carlantino	0.175	-0.027	7.63e-01	471	137.05
INGI Friuli Venezia Giulia	0.235	0.083	1.11e-01	1197	372.96
HELIC MANOLIS	0.189	0.103	8.40e-02	1058	280.56
HELIC Pomak	0.252	0.029	5.90e-01	949	332.97
INCIPE 1	0.205	-0.024	6.75e-01	937	316.97
INCIPE 2	0.198	0.094	1.56e-02	2057	658.47
LURIC	0.245	-0.005	9.11e-01	1427	510.66
Rotterdam 1	0.255	0.06	4.49e-03	5961	2241.88
Rotterdam 2	0.253	-0.022	5.41e-01	2151	807.76
Rotterdam 3	0.254	0.043	1.52e-01	3018	1127.06
TEENAGE	0.191	-0.002	9.79e-01	703	209.26
INGI Val Borbera	0.148	0.192	8.19e-05	1778	422.1
UK BioBank	0.257	0.018	4.68e-05	134797	53550.2
**Overall**	**0.254**	**0.024**	**1.94e-10**	**186106**	**73046.02**

Cohort	EAF	Beta	P-value	N	Weight
ALSPAC WGS	0.146	-0.007	8.82e-01	1794	488.58
TwinsUK WGS	0.145	0.033	4.97e-01	1747	433.31
arcOGEN	0.157	0.089	1.53e-02	2748	739.47
UKHLS	0.151	0.049	2.01e-02	8700	2245.71
FINRISK	0.129	0.065	2.72e-01	1249	282.61
ALSPAC GWA	0.136	0.046	1.52e-01	4103	977.66
TwinsUK GWA	0.148	0.058	1.82e-01	3540	525.82
INGI Friuli Venezia Giulia	0.165	0.087	1.31e-01	1197	304.01
HELIC MANOLIS	0.104	0.144	5.03e-02	1058	185.52
HELIC Pomak	0.098	0.067	3.94e-01	949	159.62
INCIPE 2	0.163	0.047	2.56e-01	2057	576.93
LURIC	0.156	0.022	6.66e-01	1427	391.37
Rotterdam 1	0.159	0.018	4.78e-01	5961	1581.73
Rotterdam 2	0.173	0.067	9.05e-02	2151	630.44
Rotterdam 3	0.159	0.052	1.38e-01	3018	813.58
TEENAGE	0.166	-0.055	4.46e-01	703	194.67
INGI Val Borbera	0.179	0.043	3.46e-01	1778	473.7
UK BioBank	0.148	0.024	5.80e-06	134797	35437.33
**Overall**	**0.149**	**0.029**	**3.06e-10**	**178977**	**47258.98**

Cohort	EAF	Beta	P-value	N	Weight
ALSPAC WGS	0.211	-0.039	3.39e-01	1794	587.84
TwinsUK WGS	0.218	-0.065	1.12e-01	1747	607.29
arcOGEN	0.231	-0.051	1.10e-01	2748	987.7
UKHLS	0.228	-0.02	2.83e-01	8700	2951.12
FINRISK	0.251	-0.139	2.67e-03	1249	471.4
ALSPAC GWA	0.219	-0.027	3.22e-01	4103	1388.66
TwinsUK GWA	0.22	-0.031	4.00e-01	3540	726.33
INGI Carlantino	0.276	-0.018	8.11e-01	471	174.43
INGI Friuli Venezia Giulia	0.21	-0.088	1.02e-01	1197	351.84
HELIC MANOLIS	0.225	-0.095	7.60e-02	1058	348.3
HELIC Pomak	0.24	0.05	3.70e-01	949	327.51
INCIPE 1	0.247	0.105	4.85e-02	937	356.63
INCIPE 2	0.242	-0.003	9.45e-01	2057	712.78
LURIC	0.215	-0.102	2.40e-02	1427	494.62
Rotterdam 1	0.209	-0.033	1.49e-01	5961	1973.03
Rotterdam 2	0.206	-0.059	1.15e-01	2151	712.02
Rotterdam 3	0.203	-0.047	1.41e-01	3018	972.49
TEENAGE	0.239	-0.057	3.62e-01	703	252.34
INGI Val Borbera	0.216	-0.084	5.41e-02	1778	532.35
UK BioBank	0.224	-0.018	5.17e-05	134797	47890.91
**Overall**	**0.224**	**-0.023**	**5.91e-09**	**180385**	**62500**

Figure S22: Locus zoom and forest plots for the novel loci reported in Table 1.
Plots display 500kb each side of the top variant, where the smaller diamond represents the discovery *P-value* and the bigger diamond the overall *P-value* (meta-analysis across discovery and follow-up cohorts). LD is calculated from the combined WGS UK10K cohorts (ALSPAC and TwinsUK). Previously reported variants are denoted by large circles.

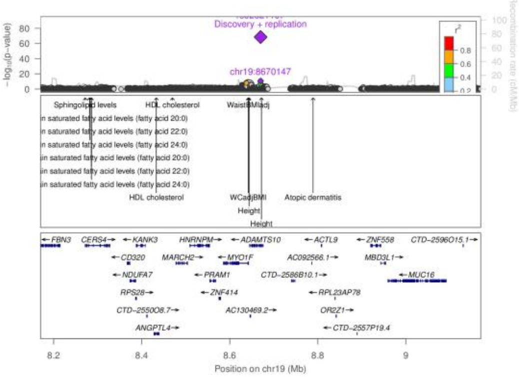

Cohort	EAF	Beta	P-value	N	Weight
ALSPAC WGS	0.034	-0.271	2.93e-03	1794	120.42
TwinsUK WGS	0.036	-0.143	1.06e-01	1747	128.15
LOLIPOP_EW610	0.037	-0.074	5.98e-01	927	50.77
LOLIPOP_EW_A	0.036	-0.033	8.58e-01	582	28.68
LOLIPOP_EW_P	0.033	0.175	4.34e-01	642	19.97
arcOGEN	0.031	-0.024	7.92e-01	2748	124.46
UKHLS	0.031	-0.156	2.04e-03	8700	389.28
FINRISK	0.015	-0.052	7.47e-01	1249	37.39
ALSPAC GWA	0.035	-0.177	1.01e-02	4103	211.93
TwinsUK GWA	0.036	-0.263	2.86e-03	3540	129.11
INGI Carlantino	0.054	-0.386	3.14e-02	471	31.42
INGI Friuli Venezia Giulia	0.046	-0.252	5.11e-02	1197	60.37
HELIC MANOLIS	0.062	-0.112	3.29e-01	1058	75.33
HELIC Pomak	0.057	-0.001	9.97e-01	949	76.96
INCIPE 1	0.042	-0.258	6.57e-02	937	51.14
INCIPE 2	0.045	-0.163	8.75e-02	2057	110.25
LURIC	0.037	-0.119	3.34e-01	1427	65.5
Rotterdam 1	0.034	-0.067	2.47e-01	5961	298.46
Rotterdam 2	0.041	-0.051	5.70e-01	2151	125.82
Rotterdam 3	0.035	-0.195	1.31e-02	3018	162.27
TEENAGE	0.056	-0.079	5.74e-01	703	50.73
INGI Val Borbera	0.042	-0.051	6.29e-01	1778	90.77
Fenland	0.039	-0.171	8.00e-06	8942	680.63
GoT2D	0.029	-0.106	6.99e-04	25750	1033.9
Copenhagen	0.033	-0.164	2.21e-12	28495	1825.82
SardiNIA	0.11	-0.129	3.90e-06	6477	1275.51
UK BioBank	0.036	-0.138	7.26e-38	134797	8710.44
**Overall**	**0.042**	**-0.139**	**3.22e-69**	**252200**	**16023.07**

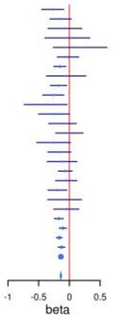

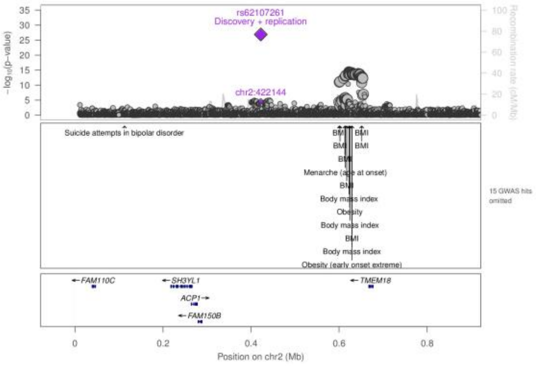

Cohort	EAF	Beta	P-value	N	Weight
ALSPAC WGS	0.05	-0.21	6.54e-03	1791	168.3
TwinsUK WGS	0.049	-0.044	5.71e-01	1747	165.59
LOLIPOP_EW610	0.058	-0.024	8.22e-01	915	86.31
LOLIPOP_EW_A	0.06	-0.157	2.80e-01	580	47.63
LOLIPOP_EW_P	0.061	-0.077	5.66e-01	640	56.02
arcOGEN	0.046	-0.146	3.65e-02	2733	205.55
UKHLS	0.048	-0.037	3.46e-01	8553	654.04
FINRISK	0.014	0.12	4.83e-01	1249	32.89
ALSPAC GWA	0.048	-0.152	6.32e-03	4101	324.45
TwinsUK GWA	0.055	-0.206	3.90e-03	3539	196.55
INGI Carlantino	0.042	-0.083	6.80e-01	472	24.74
INGI Friuli Venezia Giulia	0.044	-0.087	4.61e-01	1170	72.61
HELIC MANOLIS	0.035	0.024	8.73e-01	1033	46.41
HELIC Pomak	0.032	0.128	4.58e-01	948	33.84
INCIPE 1	0.054	-0.103	3.70e-01	934	76.16
INCIPE 2	0.063	0.168	2.71e-02	2050	173.23
LURIC	0.05	-0.016	8.70e-01	1427	107.83
Rotterdam 1	0.048	-0.039	4.05e-01	5954	467.33
Rotterdam 2	0.042	-0.044	5.92e-01	2148	150.51
Rotterdam 3	0.051	-0.074	2.28e-01	3017	262.74
TEENAGE	0.053	0.021	8.82e-01	701	49.92
INGI Val Borbera	0.041	-0.279	7.00e-03	1774	93.72
Fenland	0.05	-0.09	9.34e-03	8769	837.92
GoT2D	0.028	-0.027	3.41e-01	29996	1257.48
Copenhagen	0.039	-0.07	1.09e-03	28642	2149.47
SardiNIA	0.074	-0.085	1.20e-02	6481	865.05
UK BioBank	0.048	-0.081	3.80e-19	134509	12234.64
**Overall**	**0.047**	**-0.075**	**1.27e-27**	**255873**	**21003.99**

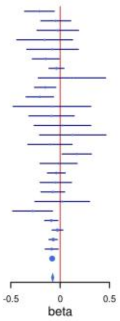

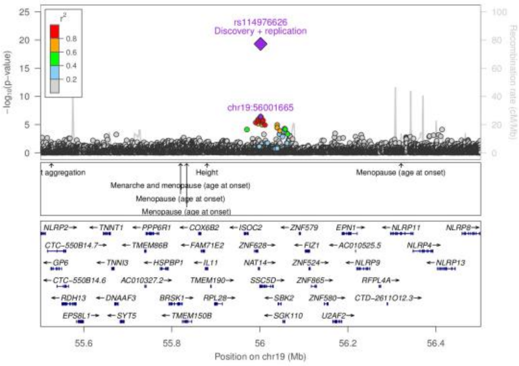

Cohort	EAF	Beta	P-value	N	Weight
ALSPAC WGS	0.03	-0.232	1.26e-02	1794	115.7
TwinsUK WGS	0.025	-0.232	3.14e-02	1747	86.26
arcOGEN	0.027	-0.141	9.90e-02	2748	137.1
UKHLS	0.025	-0.135	5.63e-03	8700	419.81
FINRISK	0.005	-0.227	4.23e-01	1249	12.4
ALSPAC GWA	0.027	-0.064	3.52e-01	4103	210.36
TwinsUK GWA	0.031	-0.238	9.12e-03	3540	120.11
INGI Friuli Venezia Giulia	0.011	-0.216	3.05e-01	1197	22.63
HELIC MANOLIS	0.047	-0.072	5.00e-01	1058	87.27
HELIC Pomak	0.033	-0.111	4.18e-01	949	57.87
INCIPE 2	0.048	-0.088	2.13e-01	2057	202.1
LURIC	0.025	-0.189	1.56e-01	1427	56.55
Rotterdam 1	0.022	-0.017	7.87e-01	5961	240.05
Rotterdam 2	0.022	0.019	8.61e-01	2151	83.49
Rotterdam 3	0.021	-0.116	2.10e-01	3018	117.18
TEENAGE	0.039	-0.152	2.88e-01	703	49.16
INGI Val Borbera	0.027	0.065	5.69e-01	1778	78.17
UK BioBank	0.026	-0.092	1.74e-14	134797	7019.35
**Overall**	**0.027**	**-0.096**	**5.00e-20**	**178977**	**9070.29**

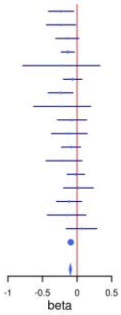

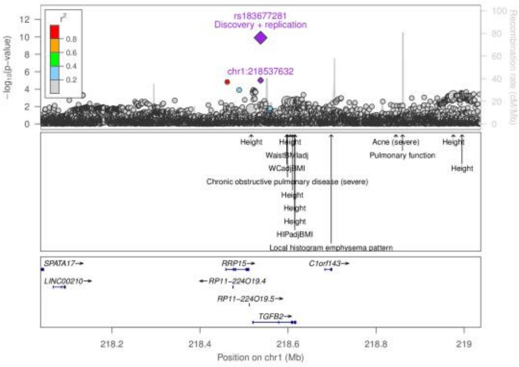

Cohort	EAF	Beta	P-value	N	Weight
ALSPAC WGS	0.025	0.075	4.70e-01	1794	91.61
TwinsUK WGS	0.033	0.061	5.18e-01	1747	113.44
arcOGEN	0.026	0.064	4.74e-01	2748	124.19
UKHLS	0.027	0.151	3.19e-03	8700	382.05
FINRISK	0.007	0.173	4.68e-01	1249	17.62
ALSPAC GWA	0.027	0.11	1.34e-01	4103	187.21
TwinsUK GWA	0.023	0.274	1.16e-02	3540	84.65
INGI Carlantino	0.013	0.139	6.85e-01	471	9.01
INGI Friuli Venezia Giulia	0.016	-0.052	7.84e-01	1197	26.59
HELIC MANOLIS	0.016	0.222	3.09e-01	1058	20.94
INCIPE 1	0.029	0.203	2.02e-01	937	39.68
INCIPE 2	0.023	0.167	1.84e-01	2057	63.63
LURIC	0.028	0.083	5.12e-01	1427	63.05
Rotterdam 1	0.039	0.018	7.20e-01	5961	379.44
Rotterdam 2	0.035	0.113	1.96e-01	2151	131.91
Rotterdam 3	0.041	0.104	1.36e-01	3018	206.11
TEENAGE	0.02	0.059	8.06e-01	703	17.19
INGI Val Borbera	0.014	-0.079	6.94e-01	1778	24.81
UK BioBank	0.026	0.062	9.81e-07	134797	6269.62
**Overall**	**0.027**	**0.071**	**1.24e-10**	**179436**	**8264.46**

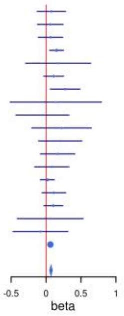

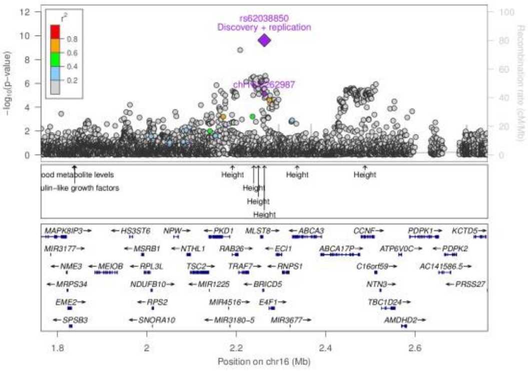

Cohort	EAF	Beta	P-value	N	Weight
ALSPAC WGS	0.025	0.119	2.59e-01	1794	90.46
TwinsUK WGS	0.021	0.372	1.64e-03	1747	71.78
arcOGEN	0.022	0.185	5.32e-02	2748	109.46
UKHLS	0.025	0.071	1.65e-01	8700	385.78
FINRISK	0.016	0.12	4.35e-01	1249	42.71
ALSPAC GWA	0.026	0.124	9.23e-02	4103	183.2
TwinsUK GWA	0.023	0.16	1.45e-01	3540	82.78
1958 Birth Cohort	0.027	0.069	2.63e-01	5721	264.25
INGI Carlantino	0.015	-0.044	9.07e-01	471	7.42
INGI Friuli Venezia Giulia	0.017	0.059	7.68e-01	1197	24.81
HELIC MANOLIS	0.013	0.084	6.87e-01	1058	22.8
HELIC Pomak	0.027	0.222	1.30e-01	949	46.08
INCIPE 1	0.014	0.322	1.30e-01	937	22.07
INCIPE 2	0.016	-0.052	6.95e-01	2057	55.86
LURIC	0.022	0.281	1.01e-01	1427	34.19
Rotterdam 1	0.018	-0.011	8.82e-01	5961	173.37
Rotterdam 2	0.019	0.254	3.77e-02	2151	67.12
Rotterdam 3	0.028	0.131	1.56e-01	3018	116.42
TEENAGE	0.012	-0.372	2.20e-01	703	10.92
INGI Val Borbera	0.009	-0.271	2.06e-01	1778	21.81
UK BioBank	0.025	0.061	1.84e-06	122318	6207.24
**Overall**	**0.024**	**0.071**	**2.44e-10**	**173627**	**7971.94**

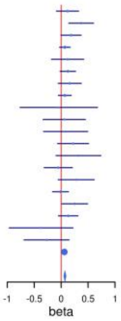

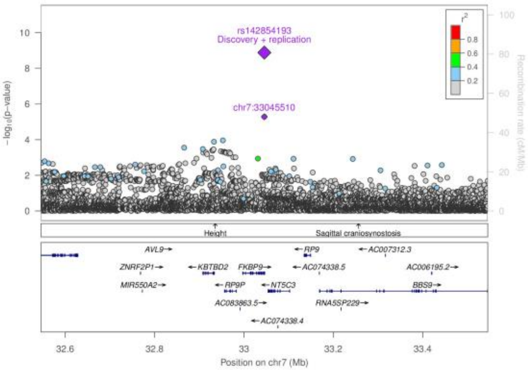

Cohort	EAF	Beta	P-value	N	Weight
ALSPAC WGS	0.027	0.066	5.30e-01	1794	91.63
TwinsUK WGS	0.027	0.116	2.69e-01	1747	91.17
arcOGEN	0.02	0.067	5.15e-01	2748	93.95
UKHLS	0.023	0.046	4.53e-01	8700	263.49
FINRISK	0.019	0.127	3.68e-01	1249	50.48
ALSPAC GWA	0.025	0.061	4.22e-01	4103	174.56
TwinsUK GWA	0.019	-0.001	9.88e-01	3540	76.18
1958 Birth Cohort	0.021	0.12	8.66e-02	5721	203.56
INGI Carlantino	0.03	0.014	9.52e-01	471	15.74
INGI Friuli Venezia Giulia	0.028	-0.065	6.73e-01	1197	42.32
HELIC MANOLIS	0.024	-0.176	3.53e-01	1058	27.95
HELIC Pomak	0.037	-0.08	6.23e-01	949	39.26
INCIPE 1	0.025	0.161	3.96e-01	937	27.84
INCIPE 2	0.029	0.234	4.80e-02	2057	71.65
LURIC	0.027	0.128	3.64e-01	1427	50.59
Rotterdam 1	0.026	0.197	1.59e-03	5961	257.62
Rotterdam 2	0.023	0.297	1.04e-02	2151	74.57
Rotterdam 3	0.026	0.196	2.06e-02	3018	139.02
TEENAGE	0.037	0.056	7.62e-01	703	29
INGI Val Borbera	0.018	0.024	8.90e-01	1778	34.53
UK BioBank	0.022	0.06	1.36e-05	134797	5262.74
**Overall**	**0.022**	**0.072**	**1.31e-09**	**186106**	**7061.65**

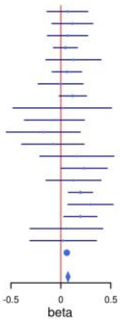

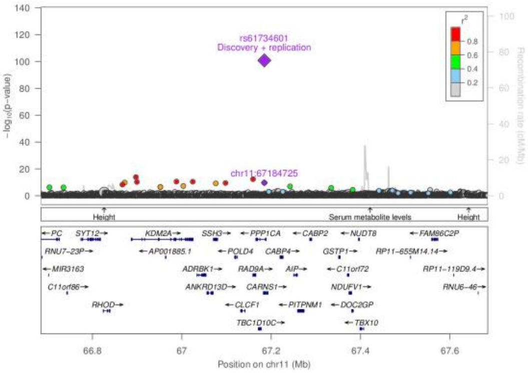

Cohort	EAF	Beta	P-value	N	Weight
Overall	0.068	0.037	6.07e-08	179850	21626.3
TwinsUK WGS	0.033	-0.001	9.89e-01	1747	115.17
arcOGEN	0.033	0.179	2.26e-02	2733	162.06
UKHLS	0.035	-0.024	5.85e-01	8553	538
ALSPAC GWA	0.031	-0.132	4.71e-02	4101	225.31
TwinsUK GWA	0.032	0.091	3.07e-01	3539	125.14
INGI Carlantino	0.069	0.266	5.51e-02	472	52.06
INGI Friuli Venezia Giulia	0.063	0.138	1.42e-01	1170	113.79
HELIC MANOLIS	0.055	0.047	6.66e-01	1033	84.84
HELIC Pomak	0.039	-0.195	1.28e-01	948	62.22
INCIPE 1	0.059	-0.034	7.42e-01	934	94.04
INCIPE 2	0.059	0.016	8.13e-01	2050	221.57
LURIC	0.042	0.079	4.13e-01	1427	108.24
Rotterdam 1	0.039	-0.035	4.74e-01	5954	411.06
Rotterdam 2	0.04	0.059	4.55e-01	2148	158.42
Rotterdam 3	0.037	-0.117	9.08e-02	3017	207.57
TEENAGE	0.061	0.123	2.86e-01	701	75.93
INGI Val Borbera	0.058	0.035	6.50e-01	1774	170.92
**Overall**	**0.068**	**0.037**	**6.07e-08**	**179850**	**21626.3**

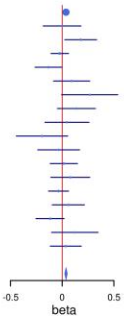

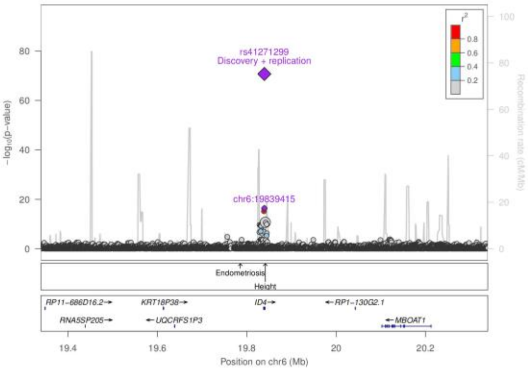

Cohort	EAF	Beta	P-value	N	Weight
ALSPAC WGS	0.047	0.092	2.27e-01	1794	174.21
TwinsUK WGS	0.053	0.259	5.40e-04	1747	178.86
arcOGEN	0.05	0.169	1.23e-02	2748	220.08
UKHLS	0.051	0.174	1.03e-05	8700	646.05
FINRISK	0.017	0.53	6.64e-04	1249	41.85
ALSPAC GWA	0.054	0.021	6.97e-01	4103	349.6
TwinsUK GWA	0.053	0.167	1.59e-02	3540	207.49
1958 Birth Cohort	0.057	0.154	4.24e-04	5721	526.41
INGI Carlantino	0.064	-0.179	2.95e-01	471	35.21
INGI Friuli Venezia Giulia	0.077	0.043	6.30e-01	1197	124.43
HELIC MANOLIS	0.041	-0.004	9.76e-01	1058	55.91
HELIC Pomak	0.034	0.352	3.08e-02	949	37.69
INCIPE 1	0.058	0.194	7.51e-02	937	84.7
INCIPE 2	0.049	0.249	7.01e-03	2057	117.35
LURIC	0.043	-0.092	3.93e-01	1427	86.08
Rotterdam 1	0.057	0.049	2.57e-01	5961	546.26
Rotterdam 2	0.053	0.233	2.09e-03	2151	175.17
Rotterdam 3	0.048	0.226	6.72e-04	3018	227.15
TEENAGE	0.061	0.091	5.00e-01	703	54.63
INGI Val Borbera	0.064	0.029	7.18e-01	1778	156.6
Fenland	0.055	0.118	2.87e-04	8990	944.08
GoT2D	0.026	0.112	4.67e-04	25750	982.69
SardiNIA	0.12	0.083	2.00e-03	6477	1371.74
UK BioBank	0.051	0.126	5.25e-49	134627	13750
**Overall**	**0.055**	**0.123**	**1.90e-71**	**227153**	**21003.99**

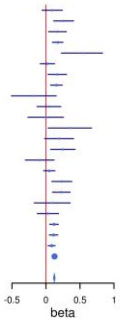

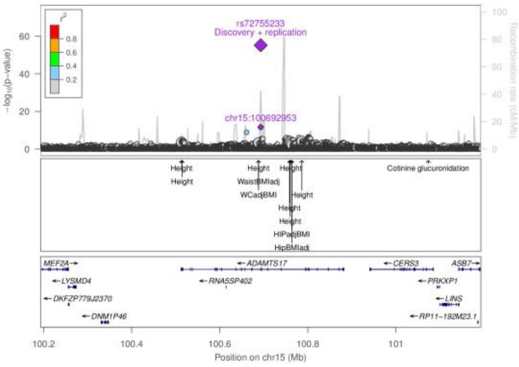

Cohort	EAF	Beta	P-value	N	Weight
ALSPAC WGS	0.115	-0.065	2.09e-01	1794	373.01
TwinsUK WGS	0.107	-0.007	9.05e-01	1747	328.77
arcOGEN	0.116	-0.101	2.92e-02	2748	468.99
UKHLS	0.117	-0.097	3.70e-05	8700	1800.19
FINRISK	0.112	-0.092	1.42e-01	1249	258.1
ALSPAC GWA	0.116	-0.064	9.99e-02	4103	655.91
TwinsUK GWA	0.121	-0.1	4.08e-02	3540	419.14
INGI Friuli Venezia Giulia	0.085	-0.051	6.03e-01	1197	101.66
HELIC MANOLIS	0.072	-0.047	5.86e-01	1058	129.42
HELIC Pomak	0.095	-0.25	1.34e-03	949	165.24
INCIPE 2	0.088	-0.064	2.28e-01	2057	354.03
LURIC	0.117	-0.171	9.84e-03	1427	229.33
Rotterdam 1	0.11	-0.033	2.99e-01	5961	973.27
Rotterdam 2	0.115	-0.062	2.57e-01	2151	338.08
Rotterdam 3	0.116	-0.094	3.40e-02	3018	513.23
TEENAGE	0.089	-0.172	1.68e-01	703	64.41
INGI Val Borbera	0.095	-0.111	1.51e-01	1778	168.03
UK BioBank	0.112	-0.084	3.40e-45	134635	28105.65
**Overall**	**0.112**	**-0.084**	**5.42e-56**	**178815**	**35599.86**

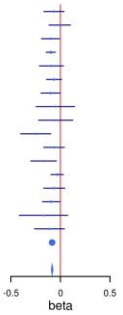

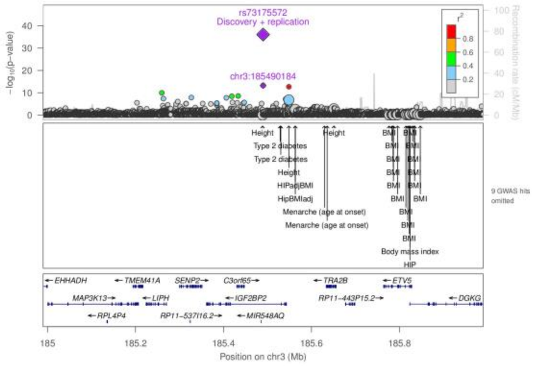

Cohort	EAF	Beta	P-value	N	Weight
ALSPAC WGS	0.114	-0.009	8.56e-01	1794	378.68
TwinsUK WGS	0.113	0.096	7.21e-02	1747	354.26
arcOGEN	0.111	0.07	1.03e-01	2748	547.61
UKHLS	0.112	0.12	8.71e-07	8700	1679.52
FINRISK	0.11	0.055	3.83e-01	1249	254.47
ALSPAC GWA	0.114	-0.003	9.39e-01	4103	785.86
TwinsUK GWA	0.109	0.178	3.00e-04	3540	415.29
INGI Carlantino	0.174	0.186	3.90e-02	471	122.33
INGI Friuli Venezia Giulia	0.133	0.071	2.70e-01	1197	236.19
HELIC MANOLIS	0.173	0.033	5.96e-01	1058	261.78
HELIC Pomak	0.158	0.031	6.45e-01	949	228.84
INCIPE 1	0.137	0.164	1.63e-02	937	216.02
INCIPE 2	0.146	0.043	3.29e-01	2057	510.62
LURIC	0.133	0.055	3.38e-01	1427	304.38
Rotterdam 1	0.125	0.128	4.78e-06	5961	1274.14
Rotterdam 2	0.124	0.033	5.03e-01	2151	424.51
Rotterdam 3	0.126	0.05	2.07e-01	3018	648.35
TEENAGE	0.144	0.104	1.67e-01	703	176.81
INGI Val Borbera	0.145	0.022	6.68e-01	1778	403.44
UK BioBank	0.112	0.063	8.26e-25	134797	27020.47
**Overall**	**0.115**	**0.067**	**8.27e-37**	**180385**	**35599.86**

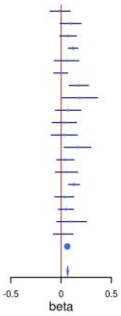

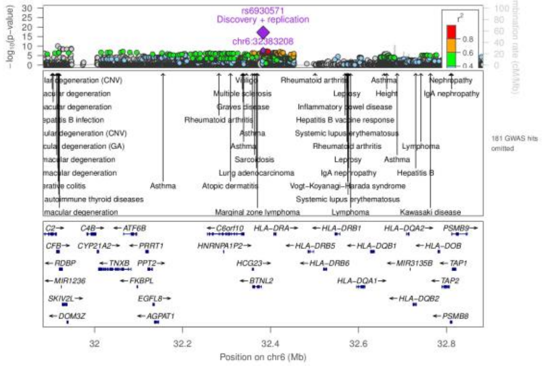

Cohort	EAF	Beta	P-value	N	Weight
ALSPAC WGS	0.155	0.06	1.79e-01	1794	507.15
TwinsUK WGS	0.15	0.065	1.58e-01	1747	467.9
arcOGEN	0.186	0.058	9.26e-02	2748	845.94
UKHLS	0.16	0.078	3.28e-04	8700	2117.58
FINRISK	0.174	0.075	1.46e-01	1249	375
ALSPAC GWA	0.178	0.03	2.95e-01	4103	1189.8
TwinsUK GWA	0.188	0.039	3.12e-01	3540	670.96
INGI Carlantino	0.043	-0.142	4.36e-01	471	30.1
INGI Friuli Venezia Giulia	0.116	-0.061	4.32e-01	1197	166.44
HELIC MANOLIS	0.035	0.114	3.64e-01	1058	63.55
HELIC Pomak	0.122	0.02	8.12e-01	949	155.07
INCIPE 2	0.111	0.077	1.18e-01	2057	407.56
LURIC	0.132	0.073	2.09e-01	1427	294.91
Rotterdam 1	0.184	0.055	4.00e-02	5961	1378.17
Rotterdam 2	0.191	-0.021	6.27e-01	2151	517.3
Rotterdam 3	0.179	0.09	1.77e-02	3018	700.76
TEENAGE	0.094	0.152	9.37e-02	703	121.64
UK BioBank	0.182	0.034	6.63e-12	134462	41846.7
**Overall**	**0.179**	**0.038**	**6.01e-18**	**177335**	**51652.89**

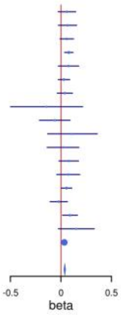

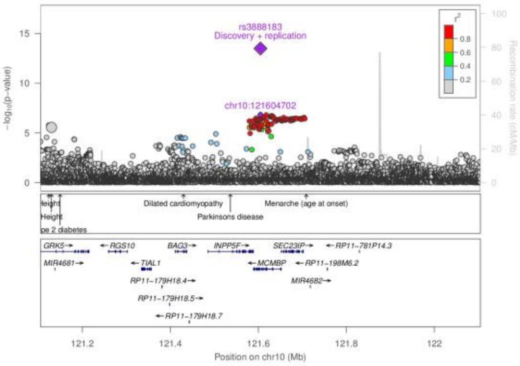

Cohort	EAF	Beta	P-value	N	Weight
ALSPAC WGS	0.104	-0.041	4.49e-01	1794	339.19
TwinsUK WGS	0.119	-0.113	3.19e-02	1747	363.99
arcOGEN	0.127	-0.034	3.92e-01	2748	622.76
UKHLS	0.122	-0.066	4.54e-03	8700	1843.26
FINRISK	0.059	0.011	8.97e-01	1249	143.38
ALSPAC GWA	0.117	-0.065	5.91e-02	4103	832.18
TwinsUK GWA	0.128	0.027	5.61e-01	3540	473.04
INGI Carlantino	0.18	-0.052	5.72e-01	471	125.13
INGI Friuli Venezia Giulia	0.139	-0.061	3.24e-01	1197	263.83
HELIC MANOLIS	0.138	0.014	8.39e-01	1058	229.44
HELIC Pomak	0.119	-0.052	4.66e-01	949	191.25
INCIPE 1	0.117	-0.056	4.33e-01	937	197.22
INCIPE 2	0.119	-0.056	2.43e-01	2057	431.4
LURIC	0.126	0.034	5.40e-01	1427	319.96
Rotterdam 1	0.118	-0.079	5.32e-03	5961	1243.86
Rotterdam 2	0.116	-0.05	2.91e-01	2151	445.03
Rotterdam 3	0.12	-0.088	2.49e-02	3018	645.1
TEENAGE	0.123	-0.086	2.93e-01	703	150.95
INGI Val Borbera	0.102	-0.066	2.42e-01	1778	308.52
UK BioBank	0.118	-0.034	8.88e-09	134797	29094.28
**Overall**	**0.118**	**-0.039**	**3.29e-14**	**180385**	**38446.75**

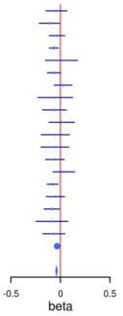

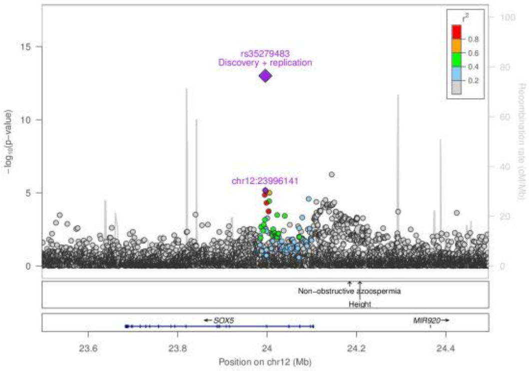

Cohort	EAF	Beta	P-value	N	Weight
ALSPAC WGS	0.402	-0.031	3.61e-01	1794	868.98
TwinsUK WGS	0.403	0.009	7.80e-01	1747	897.86
arcOGEN	0.39	-0.007	7.95e-01	2748	1314.75
UKHLS	0.405	-0.061	1.05e-04	8700	4011.86
FINRISK	0.335	0.01	8.02e-01	1249	574.8
ALSPAC GWA	0.395	0.003	9.09e-01	4103	1888.55
TwinsUK GWA	0.398	-0.027	4.00e-01	3540	953.56
INGI Carlantino	0.451	0.001	9.85e-01	471	194.8
INGI Friuli Venezia Giulia	0.421	-0.101	2.30e-02	1197	512.56
HELIC MANOLIS	0.477	-0.059	2.03e-01	1058	472.25
HELIC Pomak	0.469	0.007	8.82e-01	949	447.24
INCIPE 1	0.395	-0.006	9.00e-01	937	486.9
INCIPE 2	0.405	-0.049	1.26e-01	2057	964.11
LURIC	0.407	-0.047	2.20e-01	1427	690.7
Rotterdam 1	0.393	-0.023	2.18e-01	5961	2812.55
Rotterdam 2	0.391	-0.044	1.67e-01	2151	1000.18
Rotterdam 3	0.385	-0.033	2.15e-01	3018	1387.94
TEENAGE	0.429	-0.015	7.79e-01	703	335.58
INGI Val Borbera	0.412	-0.048	1.72e-01	1778	822.81
UK BioBank	0.402	-0.023	1.83e-09	134797	67445.03
**Overall**	**0.402**	**-0.025**	**1.00e-13**	**180385**	**86505.19**

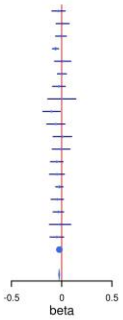

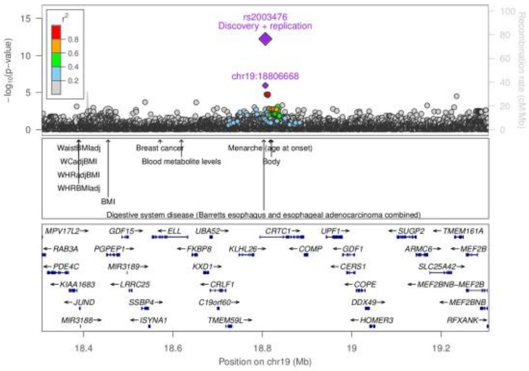

Cohort	EAF	Beta	P-value	N	Weight
ALSPAC WGS	0.393	-0.04	2.35e-01	1791	880.42
TwinsUK WGS	0.399	-0.018	6.09e-01	1747	844.66
arcOGEN	0.399	-0.01	7.38e-01	2733	1235.05
UKHLS	0.399	-0.034	2.76e-02	8553	4089.72
FINRISK	0.5	-0.058	1.43e-01	1249	629.13
ALSPAC GWA	0.409	-0.074	9.83e-04	4101	1983.76
TwinsUK GWA	0.406	0.012	7.08e-01	3539	912.06
INGI Carlantino	0.283	-0.043	5.79e-01	472	169.22
INGI Friuli Venezia Giulia	0.375	-0.133	3.21e-03	1170	497.89
HELIC MANOLIS	0.283	-0.071	1.73e-01	1033	371.59
HELIC Pomak	0.408	0.009	8.50e-01	948	425.03
INCIPE 1	0.355	-0.013	7.91e-01	934	416.54
INCIPE 2	0.35	0.019	5.66e-01	2050	905.01
LURIC	0.388	0.02	5.98e-01	1427	693.1
Rotterdam 1	0.419	-0.04	3.55e-02	5954	2833.85
Rotterdam 2	0.436	-0.049	1.12e-01	2148	1067.55
Rotterdam 3	0.414	-0.017	5.22e-01	3017	1382.47
TEENAGE	0.344	-0.028	6.15e-01	701	320.6
INGI Val Borbera	0.355	-0.077	4.45e-02	1774	685.98
UK BioBank	0.406	-0.022	3.32e-08	134509	64144.57
**Overall**	**0.404**	**-0.025**	**5.89e-13**	**179850**	**86505.19**

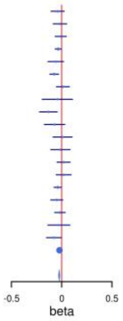

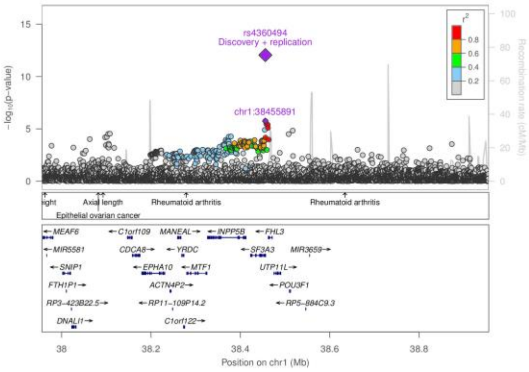

Cohort	EAF	Beta	P-value	N	Weight
ALSPAC WGS	0.449	0.091	5.31e-03	1794	949.84
TwinsUK WGS	0.461	0.005	8.86e-01	1747	886.3
arcOGEN	0.448	0.041	1.41e-01	2748	1310.75
UKHLS	0.452	0.031	4.70e-02	8700	4155.36
FINRISK	0.398	0.094	1.85e-02	1249	632.74
ALSPAC GWA	0.443	0.036	1.13e-01	4103	1926.54
TwinsUK GWA	0.449	0.071	2.53e-02	3540	1004.56
INGI Carlantino	0.526	0.055	4.57e-01	471	182.59
INGI Friuli Venezia Giulia	0.485	-0.056	2.16e-01	1197	487.1
HELIC MANOLIS	0.513	-0.021	6.54e-01	1058	466.72
HELIC Pomak	0.467	-0.043	3.58e-01	949	455.86
INCIPE 1	0.493	0.058	2.06e-01	937	467.33
INCIPE 2	0.481	0.052	9.67e-02	2057	1012.76
LURIC	0.467	0.053	1.62e-01	1427	698.91
Rotterdam 1	0.441	0.032	9.55e-02	5961	2780.32
Rotterdam 2	0.429	0.053	9.75e-02	2151	988.88
Rotterdam 3	0.442	0.028	2.89e-01	3018	1407.38
TEENAGE	0.5	-0.043	4.14e-01	703	353.17
INGI Val Borbera	0.485	-0.022	5.55e-01	1778	739.68
UK BioBank	0.445	0.021	3.24e-08	134797	69302.77
**Overall**	**0.447**	**0.024**	**8.98e-13**	**180385**	**91827.36**

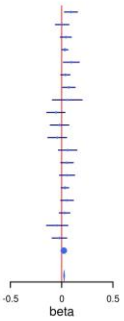

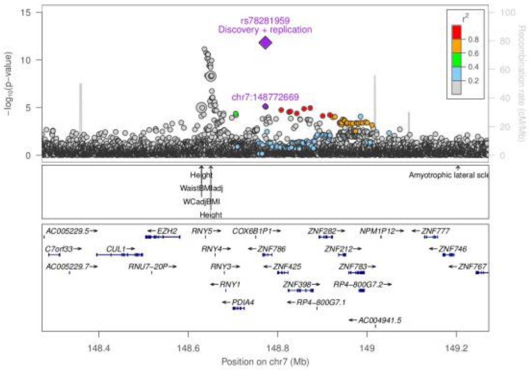

Cohort	EAF	Beta	P-value	N	Weight
ALSPAC WGS	0.055	0.053	4.59e-01	1794	194.93
TwinsUK WGS	0.058	0.066	3.47e-01	1747	201.12
arcOGEN	0.069	0.017	7.40e-01	2748	364.27
UKHLS	0.063	0.065	3.91e-02	8700	1000.3
FINRISK	0.064	0.103	1.93e-01	1249	160.81
ALSPAC GWA	0.065	0.063	1.74e-01	4103	471.83
TwinsUK GWA	0.068	-0.051	4.10e-01	3540	266.26
1958 Birth Cohort	0.065	0.111	3.96e-03	5721	679.51
INGI Carlantino	0.036	-0.007	9.73e-01	471	30.61
INGI Friuli Venezia Giulia	0.057	0.098	2.85e-01	1197	117.15
HELIC MANOLIS	0.023	-0.009	9.54e-01	1058	40.83
HELIC Pomak	0.101	0.02	8.17e-01	949	157.82
INCIPE 1	0.058	0.214	2.79e-02	937	105.49
INCIPE 2	0.057	0.115	9.34e-02	2057	214.51
LURIC	0.067	-0.056	4.54e-01	1427	179.43
Rotterdam 1	0.068	0.036	3.27e-01	5961	732
Rotterdam 2	0.072	0.109	6.99e-02	2151	276.08
Rotterdam 3	0.068	-0.004	9.37e-01	3018	381.26
TEENAGE	0.031	0.452	4.16e-03	703	40.49
INGI Val Borbera	0.066	0.069	3.34e-01	1778	197.88
UK BioBank	0.062	0.044	2.77e-08	134797	16021.94
**Overall**	**0.062**	**0.048**	**1.56e-12**	**186106**	**21626.3**

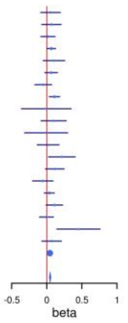

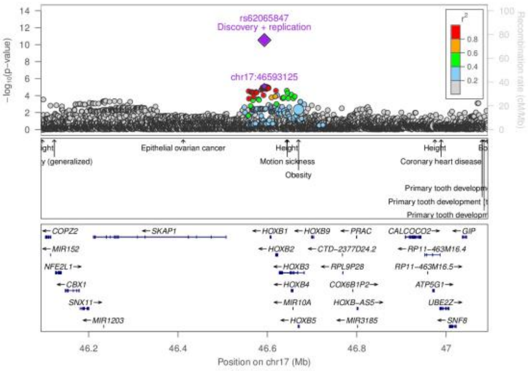

Cohort	EAF	Beta	P-value	N	Weight
ALSPAC WGS	0.476	-0.028	3.85e-01	1807	931.15
TwinsUK WGS	0.482	-0.057	1.55e-01	1265	622.88
UKHLS	0.492	-0.031	4.26e-02	8727	4333.38
FINRISK	0.431	-0.008	8.49e-01	1254	607.14
ALSPAC GWA	0.473	-0.035	1.13e-01	4121	2105.75
TwinsUK GWA	0.494	-0.076	3.77e-02	2585	753.41
1958 Birth Cohort	0.482	-0.04	3.09e-02	5713	2845.96
INGI Carlantino	0.552	-0.042	5.85e-01	397	170.8
INGI Friuli Venezia Giulia	0.54	-0.157	2.92e-03	791	360.2
HELIC MANOLIS	0.476	-0.048	2.92e-01	1075	487.05
HELIC Pomak	0.542	0.005	9.20e-01	903	402.58
INCIPE 1	0.496	0.066	1.57e-01	934	455.13
INCIPE 2	0.483	-0.02	5.08e-01	2050	1042.67
LURIC	0.467	-0.037	3.30e-01	1403	707.86
Rotterdam 1	0.505	-0.002	9.01e-01	5665	2812.85
Rotterdam 2	0.511	-0.024	4.61e-01	1938	922.69
Rotterdam 3	0.504	-0.043	1.00e-01	2930	1465.5
TEENAGE	0.451	-0.066	2.27e-01	698	333.79
INGI Val Borbera	0.438	-0.005	8.77e-01	1740	920.46
UK BioBank	0.485	-0.02	3.23e-07	134798	67319.79
**Overall**	**0.486**	**-0.022**	**2.86e-11**	**180794**	**91827.36**

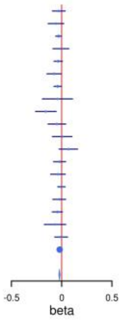

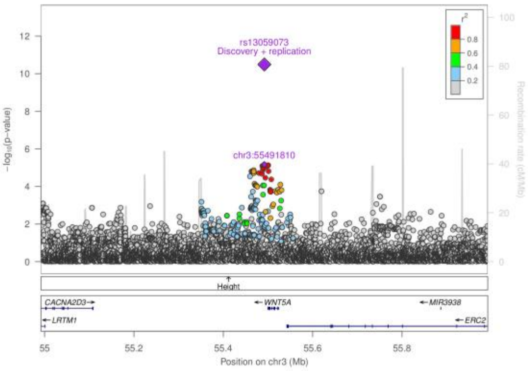

Cohort	EAF	Beta	P-value	N	Weight
ALSPAC WGS	0.457	0.045	1.76e-01	1794	917.72
TwinsUK WGS	0.443	0.013	6.97e-01	1747	884.66
arcOGEN	0.455	-0.013	6.23e-01	2748	1335.78
UKHLS	0.451	0.031	4.65e-02	8700	4240.76
FINRISK	0.408	0.042	2.96e-01	1249	625.24
ALSPAC GWA	0.456	0.017	4.39e-01	4103	1979
TwinsUK GWA	0.448	0.023	4.48e-01	3540	1044.77
1958 Birth Cohort	0.451	0.02	2.87e-01	5721	2863.96
INGI Carlantino	0.419	0.031	6.41e-01	471	223.23
INGI Friuli Venezia Giulia	0.488	0.022	6.17e-01	1197	521.96
HELIC MANOLIS	0.471	0.057	2.10e-01	1058	484.92
HELIC Pomak	0.425	0.012	8.17e-01	949	435.76
INCIPE 1	0.44	0.066	1.51e-01	937	470.05
INCIPE 2	0.459	0.026	3.98e-01	2057	1078.23
LURIC	0.441	0.038	3.21e-01	1427	687.23
Rotterdam 1	0.454	0.038	3.97e-02	5961	2989.98
Rotterdam 2	0.438	0.078	1.08e-02	2151	1063.86
Rotterdam 3	0.46	0.04	1.26e-01	3018	1473.39
TEENAGE	0.455	-0.003	9.61e-01	703	334.62
INGI Val Borbera	0.494	0.009	7.86e-01	1778	819.4
UK BioBank	0.456	0.019	4.52e-07	134797	69380.92
**Overall**	**0.455**	**0.022**	**3.23e-11**	**186106**	**91827.36**

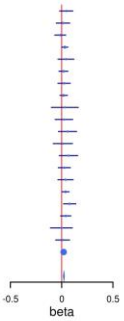

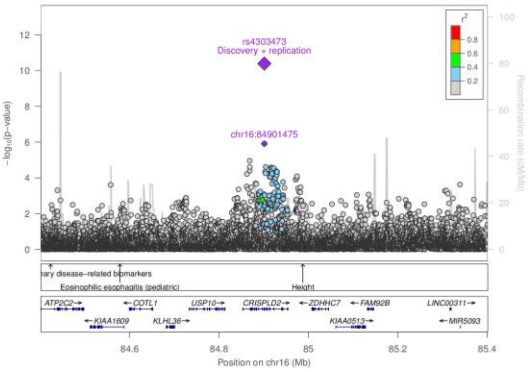

Cohort	EAF	Beta	P-value	N	Weight
ALSPAC WGS	0.377	0.03	3.71e-01	1794	872.01
TwinsUK WGS	0.382	0.024	4.81e-01	1747	843.38
arcOGEN	0.387	0.034	2.39e-01	2748	1226.58
UKHLS	0.374	0.046	3.77e-03	8700	3981.04
FINRISK	0.362	0.025	5.42e-01	1249	610.22
ALSPAC GWA	0.381	0.03	2.02e-01	4103	1858.55
TwinsUK GWA	0.374	0.025	4.37e-01	3540	934.69
1958 Birth Cohort	0.378	0.049	1.09e-02	5721	2674.65
INGI Carlantino	0.437	-0.081	2.49e-01	471	203.76
INGI Friuli Venezia Giulia	0.418	-0.014	7.54e-01	1197	466.76
HELIC MANOLIS	0.459	0.033	4.65e-01	1058	479.21
HELIC Pomak	0.368	0.075	1.26e-01	949	414.25
INCIPE 1	0.436	0.073	1.12e-01	937	473.35
INCIPE 2	0.41	-0.006	8.50e-01	2057	979.99
LURIC	0.378	0.022	5.68e-01	1427	660.44
Rotterdam 1	0.383	0.043	2.37e-02	5961	2766.88
Rotterdam 2	0.386	0.003	9.18e-01	2151	1012.37
Rotterdam 3	0.389	0.016	5.56e-01	3018	1362.84
TEENAGE	0.444	-0.051	3.50e-01	703	338.13
INGI Val Borbera	0.45	0.056	1.16e-01	1778	778.97
UK BioBank	0.376	0.019	1.60e-06	134797	65252.38
**Overall**	**0.38**	**0.022**	**4.08e-11**	**186106**	**86505.19**

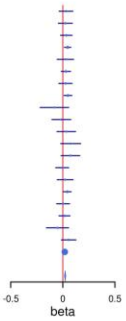

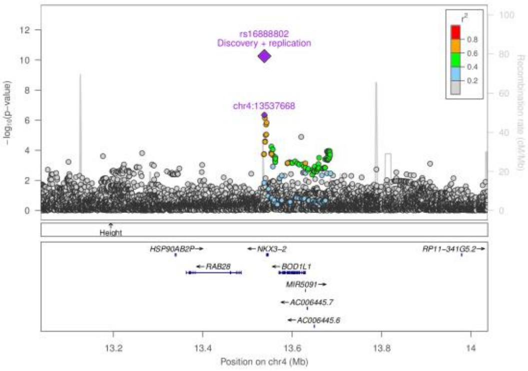

Cohort	EAF	Beta	P-value	N	Weight
ALSPAC WGS	0.172	0.05	2.57e-01	1794	513.35
TwinsUK WGS	0.182	-0.093	3.56e-02	1747	514.14
arcOGEN	0.17	0.091	1.43e-02	2748	720.38
UKHLS	0.177	0.063	2.01e-03	8700	2368.66
FINRISK	0.167	0.019	7.24e-01	1249	352.91
ALSPAC GWA	0.176	0.034	2.56e-01	4103	1116.91
TwinsUK GWA	0.174	0.01	8.13e-01	3540	578.92
1958 Birth Cohort	0.174	0.049	5.21e-02	5721	1587.15
INGI Carlantino	0.184	0.294	2.11e-03	471	109.59
INGI Friuli Venezia Giulia	0.18	0.008	8.94e-01	1197	281.62
HELIC MANOLIS	0.199	0.06	3.07e-01	1058	287.93
HELIC Pomak	0.202	0.074	2.40e-01	949	255.8
INCIPE 1	0.186	0.012	8.51e-01	937	248.81
INCIPE 2	0.18	0.106	1.10e-02	2057	575.66
LURIC	0.17	0.079	1.47e-01	1427	338.08
Rotterdam 1	0.176	0.025	3.12e-01	5961	1586.52
Rotterdam 2	0.181	0.01	8.05e-01	2151	605.47
Rotterdam 3	0.183	0.052	1.32e-01	3018	836.28
TEENAGE	0.196	0.023	7.45e-01	703	206.06
INGI Val Borbera	0.202	0.02	6.58e-01	1778	484.89
UK BioBank	0.175	0.023	3.19e-06	134615	40631.54
**Overall**	**0.176**	**0.028**	**5.49e-11**	**185924**	**54083.29**

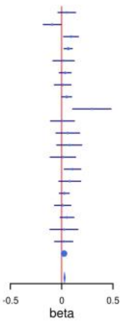

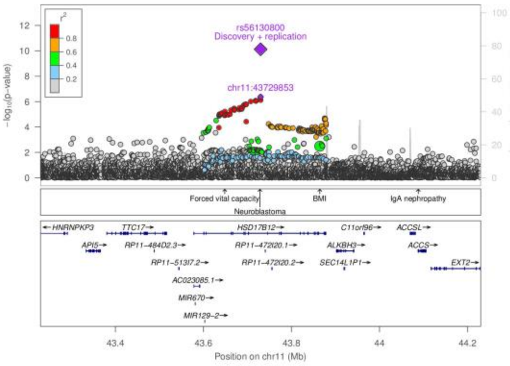

Cohort	EAF	Beta	P-value	N	Weight
ALSPAC WGS	0.324	-0.009	7.93e-01	1807	837.73
TwinsUK WGS	0.308	0.036	4.02e-01	1265	549.23
UKHLS	0.317	0.043	8.73e-03	8727	3781.86
ALSPAC GWA	0.32	0.03	1.95e-01	4121	1815.1
TwinsUK GWA	0.311	0.026	5.14e-01	2585	650.42
1958 Birth Cohort	0.312	0.034	8.76e-02	5713	2472.73
INGI Carlantino	0.377	0.016	8.25e-01	397	182.35
INGI Friuli Venezia Giulia	0.414	0.009	8.65e-01	791	360.53
HELIC MANOLIS	0.314	0.049	3.16e-01	1075	413.13
HELIC Pomak	0.332	0.032	5.37e-01	903	381.6
INCIPE 1	0.336	0.008	8.65e-01	934	411.62
INCIPE 2	0.334	0.05	1.18e-01	2050	964.83
LURIC	0.309	0.041	3.21e-01	1403	594.3
Rotterdam 1	0.311	0.039	6.06e-02	5665	2370.74
Rotterdam 2	0.306	0.059	8.81e-02	1938	823.61
Rotterdam 3	0.308	0.047	9.84e-02	2930	1250.2
TEENAGE	0.352	0.05	3.72e-01	698	320.12
INGI Val Borbera	0.3	0.047	1.85e-01	1740	807.04
UK BioBank	0.317	0.019	4.09e-06	134798	58319.9
**Overall**	**0.317**	**0.023**	**7.52e-11**	**179540**	**77160.49**

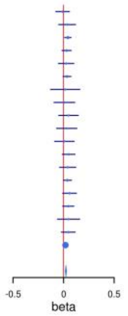

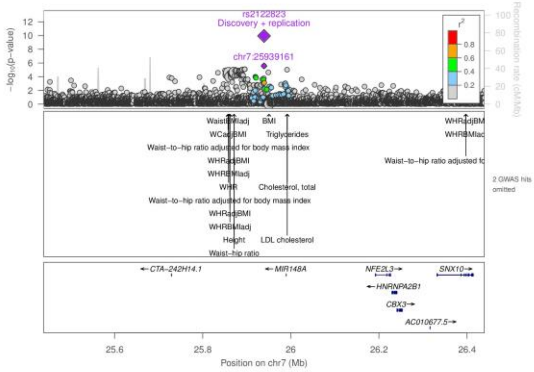

Cohort	EAF	Beta	P-value	N	Weight
ALSPAC WGS	0.203	0.062	1.35e-01	1806	585.11
TwinsUK WGS	0.212	0.033	4.99e-01	1265	430.4
FINRISK	0.246	0.017	7.19e-01	1254	461.97
ALSPAC GWA	0.21	0.055	4.72e-02	4116	1313.23
TwinsUK GWA	0.217	-0.034	4.42e-01	2582	502.5
1958 Birth Cohort	0.208	0.043	6.08e-02	5720	1890.03
INGI Carlantino	0.224	-0.024	7.82e-01	388	127.95
INGI Friuli Venezia Giulia	0.199	0.019	7.73e-01	791	227.51
HELIC MANOLIS	0.205	0.084	1.43e-01	1068	303.33
HELIC Pomak	0.182	-0.025	7.05e-01	895	226.76
LURIC	0.212	0.115	1.72e-02	1400	427.34
Rotterdam 1	0.198	0.047	4.25e-02	5660	1829.72
Rotterdam 2	0.215	0.085	2.86e-02	1937	664.43
Rotterdam 3	0.203	0.059	6.53e-02	2928	961.66
TEENAGE	0.251	0.037	5.53e-01	697	256.14
UK BioBank	0.211	0.023	9.98e-07	134795	43727.03
**Overall**	**0.21**	**0.028**	**1.13e-10**	**167302**	**54083.29**

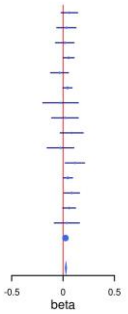

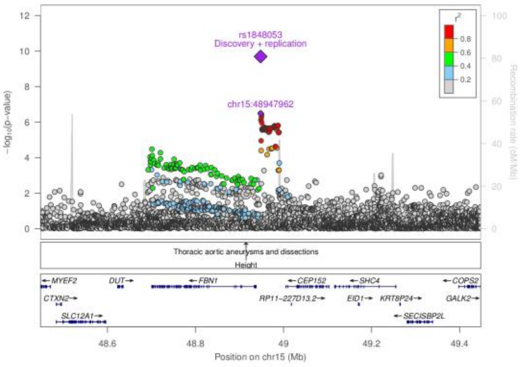

Cohort	EAF	Beta	P-value	N	Weight
ALSPAC WGS	0.252	-0.015	6.91e-01	1794	707.33
TwinsUK WGS	0.251	-0.023	5.58e-01	1747	663.4
arcOGEN	0.261	-0.053	8.70e-02	2748	1032.24
UKHLS	0.251	-0.026	1.54e-01	8700	3102.95
FINRISK	0.282	-0.057	1.93e-01	1249	525.76
ALSPAC GWA	0.249	-0.03	2.37e-01	4103	1525.88
TwinsUK GWA	0.247	-0.086	1.86e-02	3540	750.83
1958 Birth Cohort	0.245	-0.037	9.38e-02	5721	2002.81
INGI Carlantino	0.222	0.065	4.43e-01	471	136.96
INGI Friuli Venezia Giulia	0.262	-0.042	4.17e-01	1197	372.95
HELIC MANOLIS	0.256	0.001	9.79e-01	1058	356.14
HELIC Pomak	0.301	0.019	7.10e-01	949	376.9
INCIPE 1	0.234	-0.106	6.19e-02	937	313.14
INCIPE 2	0.236	-0.045	2.38e-01	2057	678.66
LURIC	0.236	-0.055	2.19e-01	1427	498.58
Rotterdam 1	0.232	-0.049	2.76e-02	5961	2017.23
Rotterdam 2	0.242	-0.055	1.40e-01	2151	733.23
Rotterdam 3	0.238	-0.033	2.97e-01	3018	1025.51
TEENAGE	0.225	-0.129	4.93e-02	703	234.73
INGI Val Borbera	0.258	-0.018	6.62e-01	1778	575.85
UK BioBank	0.248	-0.019	1.24e-05	134797	50817.47
**Overall**	**0.248**	**-0.024**	**2.00e-10**	**186106**	**69252.08**

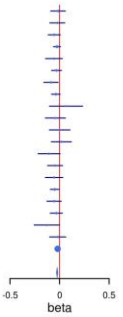

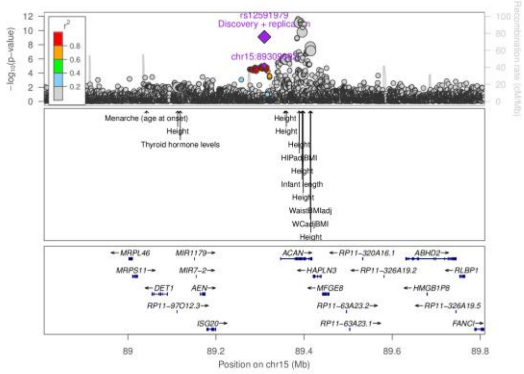

Cohort	EAF	Beta	P-value	N	Weight
ALSPAC WGS	0.163	-0.06	1.77e-01	1794	500.77
TwinsUK WGS	0.163	-0.035	4.47e-01	1747	462.14
arcOGEN	0.161	-0.09	1.52e-02	2748	735.86
UKHLS	0.17	-0.033	1.05e-01	8700	2343.5
FINRISK	0.222	-0.004	9.36e-01	1249	455.69
ALSPAC GWA	0.16	-0.059	5.72e-02	4103	1038.44
TwinsUK GWA	0.159	-0.046	2.67e-01	3540	569.6
INGI Carlantino	0.152	-0.143	1.40e-01	471	108.17
INGI Friuli Venezia Giulia	0.17	0.014	8.21e-01	1197	275.32
HELIC MANOLIS	0.112	-0.1	1.79e-01	1058	181.24
HELIC Pomak	0.157	-0.056	3.90e-01	949	229.55
INCIPE 1	0.135	-0.044	5.09e-01	937	229.42
INCIPE 2	0.144	-0.101	2.98e-02	2057	464.48
LURIC	0.16	-0.052	3.16e-01	1427	370.38
Rotterdam 1	0.155	-0.033	1.92e-01	5961	1527.79
Rotterdam 2	0.167	-0.044	2.86e-01	2151	584.74
Rotterdam 3	0.169	0.013	7.18e-01	3018	813.4
TEENAGE	0.132	0.05	5.37e-01	703	155.13
INGI Val Borbera	0.124	-0.045	4.10e-01	1778	333.32
UK BioBank	0.165	-0.024	4.86e-06	134797	37438.46
**Overall**	**0.164**	**-0.028**	**8.06e-10**	**180385**	**49382.72**

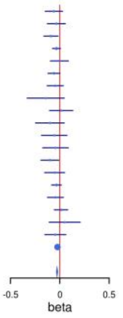

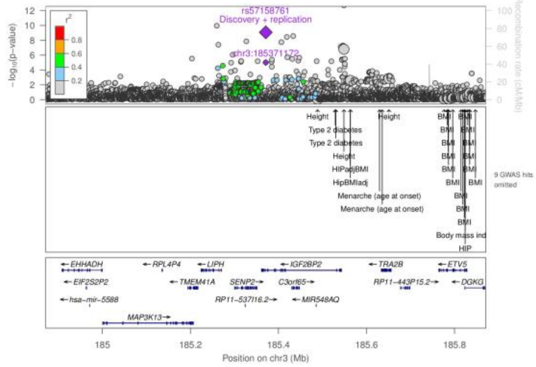

Cohort	EAF	Beta	P-value	N	Weight
ALSPAC WGS	0.426	-0.039	2.40e-01	1794	908.67
TwinsUK WGS	0.454	-0.091	6.22e-03	1747	903.05
arcOGEN	0.441	-0.016	5.61e-01	2748	1360.83
UKHLS	0.44	-0.027	8.20e-02	8700	4219.3
FINRISK	0.388	-0.057	1.53e-01	1249	618.57
ALSPAC GWA	0.431	-0.057	1.05e-02	4103	2001.2
TwinsUK GWA	0.432	-0.04	2.04e-01	3540	1024.89
INGI Carlantino	0.455	-0.064	3.45e-01	471	225.64
INGI Friuli Venezia Giulia	0.45	-0.033	4.50e-01	1197	534.3
HELIC MANOLIS	0.496	-0.019	6.77e-01	1058	474.5
HELIC Pomak	0.471	-0.05	2.95e-01	949	435.69
INCIPE 1	0.476	-0.004	9.27e-01	937	484.82
INCIPE 2	0.471	-0.011	7.11e-01	2057	1067.55
LURIC	0.446	-0.034	3.59e-01	1427	708.88
Rotterdam 1	0.441	-0.005	7.89e-01	5961	2938.02
Rotterdam 2	0.444	-0.034	2.73e-01	2151	1062.33
Rotterdam 3	0.432	-0.015	5.69e-01	3018	1438.83
TEENAGE	0.485	-0.102	6.04e-02	703	342.36
INGI Val Borbera	0.503	0.003	9.33e-01	1778	787.33
UK BioBank	0.435	-0.017	5.20e-06	134797	68416.66
**Overall**	**0.437**	**-0.02**	**8.35e-10**	**180385**	**91827.36**

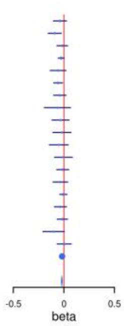

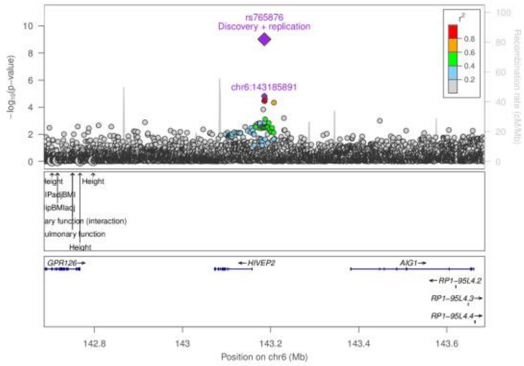

Cohort	EAF	Beta	P-value	N	Weight
ALSPAC WGS	0.477	-0.045	1.66e-01	1791	933.2
TwinsUK WGS	0.499	0.006	8.54e-01	1747	894.48
arcOGEN	0.48	-0.055	4.89e-02	2733	1296.45
UKHLS	0.489	-0.04	7.57e-03	8553	4370.13
ALSPAC GWA	0.492	-0.059	7.26e-03	4101	2071.2
TwinsUK GWA	0.477	-0.092	3.22e-03	3539	1035.19
INGI Carlantino	0.437	0.095	1.68e-01	472	211.12
INGI Friuli Venezia Giulia	0.48	-0.062	1.54e-01	1170	534.7
HELIC MANOLIS	0.36	0.014	7.78e-01	1033	419.76
HELIC Pomak	0.517	0.018	7.11e-01	948	438.5
INCIPE 1	0.475	-0.027	5.54e-01	934	474.92
INCIPE 2	0.461	-0.031	3.17e-01	2050	1054.69
LURIC	0.47	0.037	3.17e-01	1427	730.58
Rotterdam 1	0.462	-0.03	1.04e-01	5954	2977.26
Rotterdam 2	0.471	-0.005	8.58e-01	2148	1084.77
Rotterdam 3	0.463	-0.044	8.71e-02	3017	1529.46
TEENAGE	0.461	-0.001	9.82e-01	701	337.85
INGI Val Borbera	0.494	0.061	7.76e-02	1774	834.32
UK BioBank	0.491	-0.018	4.56e-06	134509	67300.93
**Overall**	**0.488**	**-0.021**	**9.64e-10**	**178601**	**86505.19**

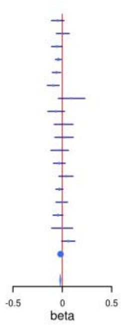

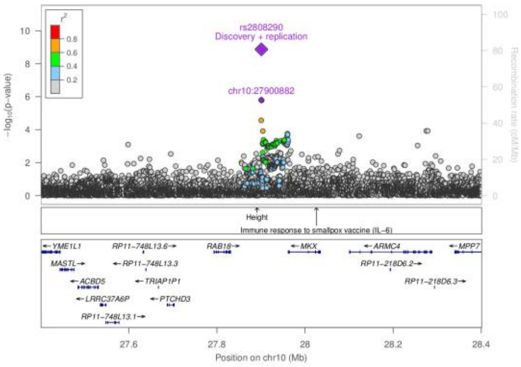

Cohort	EAF	Beta	P-value	N	Weight
ALSPAC WGS	0.502	0.031	3.44e-01	1794	939.5
TwinsUK WGS	0.513	0.034	2.99e-01	1747	913.56
arcOGEN	0.51	0.054	4.26e-02	2748	1387.01
UKHLS	0.502	0.037	1.57e-02	8700	4214.92
FINRISK	0.375	0.001	9.85e-01	1249	610.78
ALSPAC GWA	0.502	-0.002	9.42e-01	4103	1995.48
TwinsUK GWA	0.504	0.048	1.25e-01	3540	1031.82
1958 Birth Cohort	0.508	0.036	5.29e-02	5721	2892.68
INGI Carlantino	0.445	-0.026	7.21e-01	471	198.72
INGI Friuli Venezia Giulia	0.514	0.039	3.58e-01	1197	551.44
HELIC MANOLIS	0.495	0.09	5.55e-02	1058	459.53
HELIC Pomak	0.485	0.083	7.71e-02	949	461.55
INCIPE 1	0.481	-0.011	8.21e-01	937	445.95
INCIPE 2	0.488	0.072	2.57e-02	2057	965.19
LURIC	0.492	0.098	1.00e-02	1427	695.81
Rotterdam 1	0.5	0.022	2.24e-01	5961	2967.54
Rotterdam 2	0.502	0.067	3.34e-02	2151	1014.89
Rotterdam 3	0.505	-0.024	3.62e-01	3018	1474.86
TEENAGE	0.48	-0.017	7.53e-01	703	337.14
INGI Val Borbera	0.512	-0.021	5.54e-01	1778	766.12
UK BioBank	0.503	0.016	2.64e-05	134797	69336.72
**Overall**	**0.502**	**0.02**	**1.34e-09**	**186106**	**91827.36**

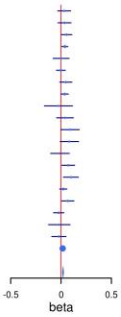

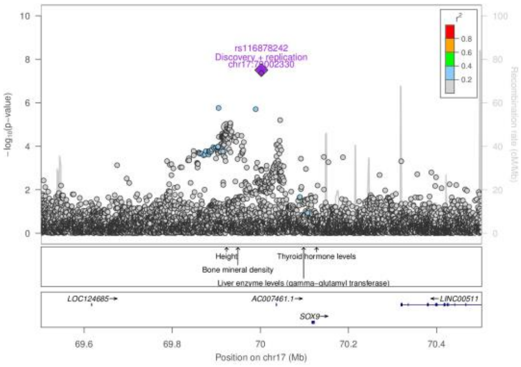

Cohort	EAF	Beta	P-value	N	Weight
ALSPAC WGS	0.067	0.086	1.93e-01	1794	228.31
TwinsUK WGS	0.069	0.035	6.01e-01	1747	226.06
arcOGEN	0.071	0.08	1.26e-01	2748	362.47
UKHLS	0.073	0.036	2.31e-01	8700	1131.99
FINRISK	0.069	0.028	7.16e-01	1249	164.54
ALSPAC GWA	0.075	0.021	6.15e-01	4103	549.9
TwinsUK GWA	0.079	0.113	5.45e-02	3540	290.52
1958 Birth Cohort	0.07	0.099	5.87e-03	5721	774.53
INGI Carlantino	0.02	0.482	5.74e-02	471	15.54
INGI Friuli Venezia Giulia	0.042	0.142	2.01e-01	1197	80.79
HELIC MANOLIS	0.067	0.103	2.72e-01	1058	113.87
HELIC Pomak	0.072	0.043	6.47e-01	949	123.04
INCIPE 1	0.068	0.011	9.10e-01	937	110.11
INCIPE 2	0.069	0.124	4.59e-02	2057	258.32
LURIC	0.082	0.126	7.82e-02	1427	196.94
Rotterdam 1	0.073	0.022	5.39e-01	5961	808.73
Rotterdam 2	0.071	0.045	4.60e-01	2151	265.6
Rotterdam 3	0.07	0.134	7.90e-03	3018	391.47
TEENAGE	0.066	0.13	2.44e-01	703	79.76
INGI Val Borbera	0.057	0.161	3.88e-02	1778	164.98
GoT2D	0.085	0.016	3.24e-01	25750	3628.97
SardiNIA	0.014	-0.042	5.70e-01	6477	187.65
UK BioBank	0.076	0.024	9.41e-04	134797	18688.83
**Overall**	**0.075**	**0.033**	**3.14e-08**	**218333**	**28727.38**

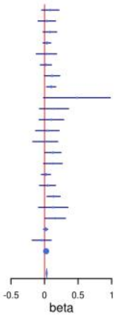

Figure S23: Locus zoom and forest plots for the novel loci reported in Table 2.
Plots display 500kb each side of the top variant, where the smaller diamond represents the discovery *P-value* and the bigger diamond the overall *P-value* (meta-analysis across discovery and follow-up cohorts). LD is calculated from the combined WGS UK10K cohorts (ALSPAC and TwinsUK). Previously reported variants are denoted by large circles.

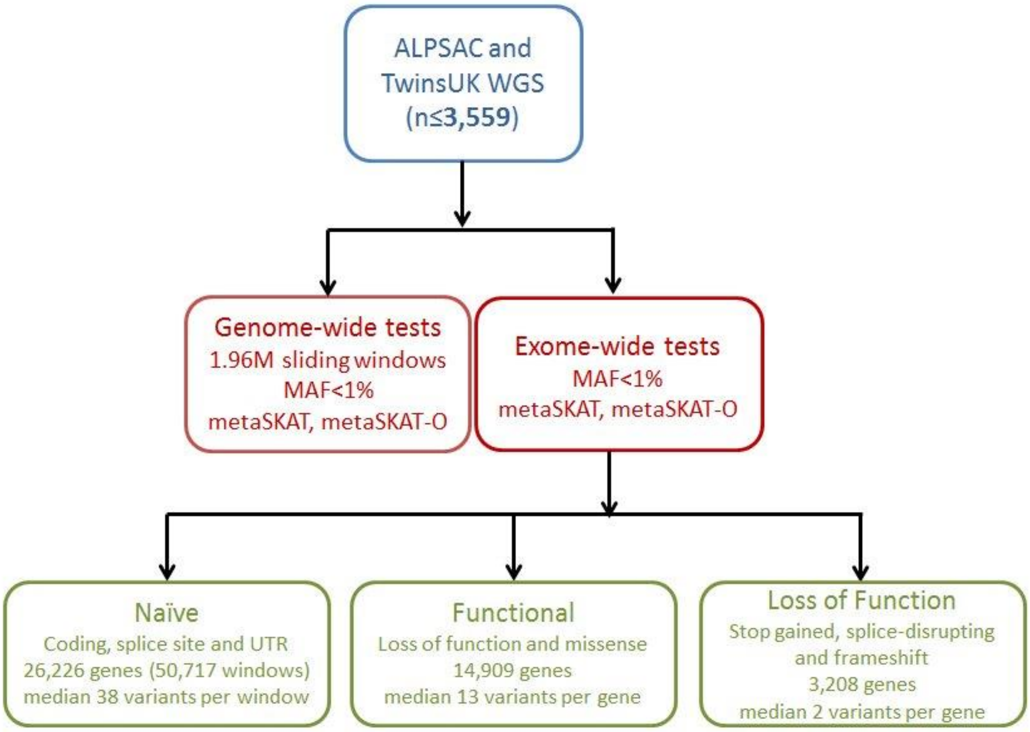

Figure S24: Study design for rare variants tests.

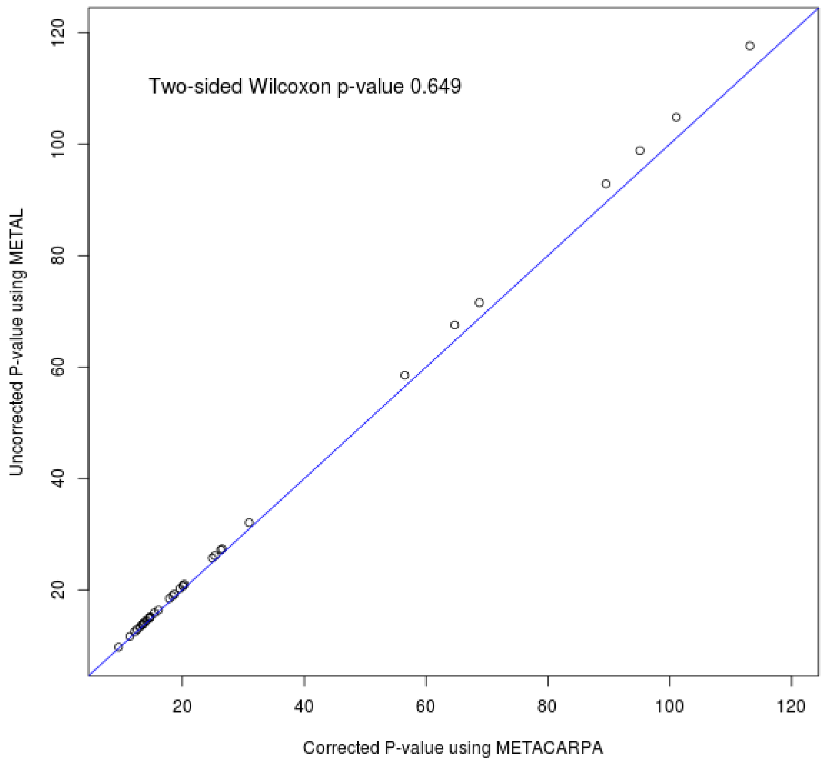

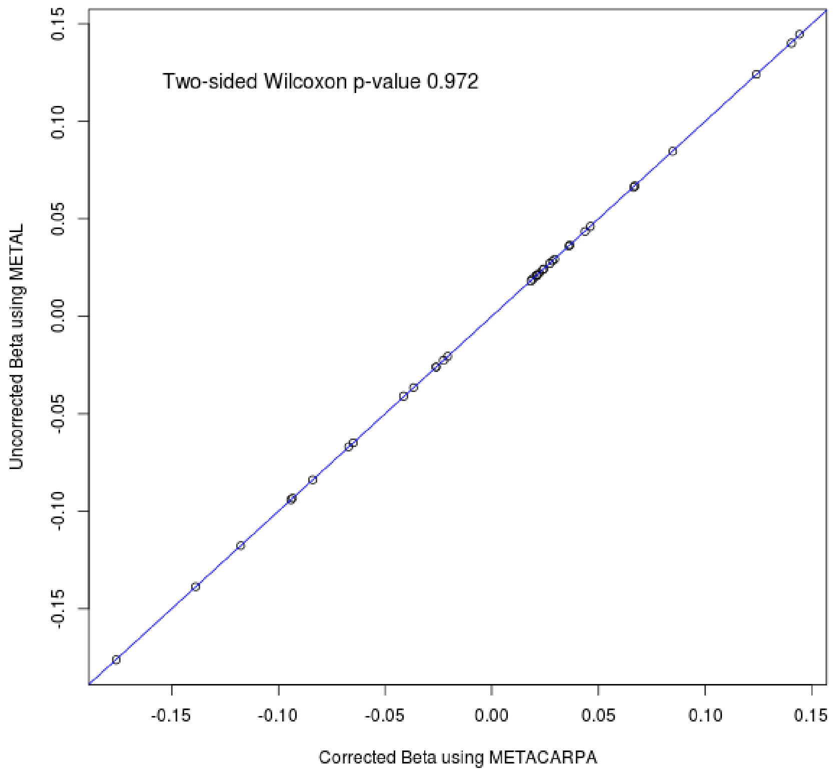

Figure S25: Meta-analysis *P-values* (top) and effect sizes (bottom) of variants associated with height across discovery cohorts and UKBiobank using METACARPA and METAL.

A

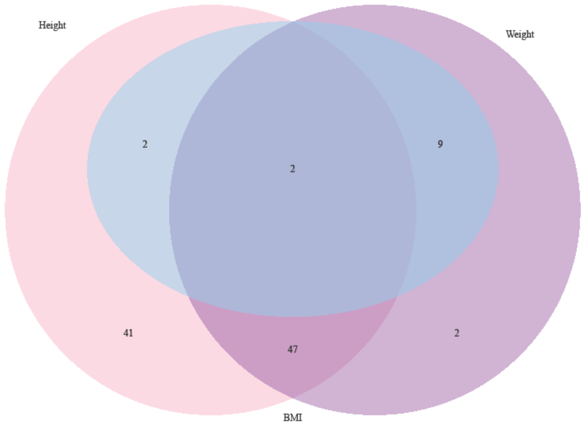
Height
Weight
BMI
2
2
9
41
47
2

B

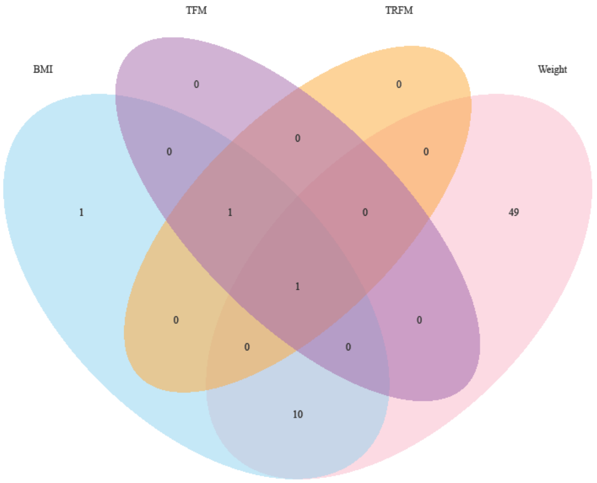
TFM
TRFM
BMI
Weight
0
0
0
0
0
1
1
0
49
1
0
0
0
0
10

C

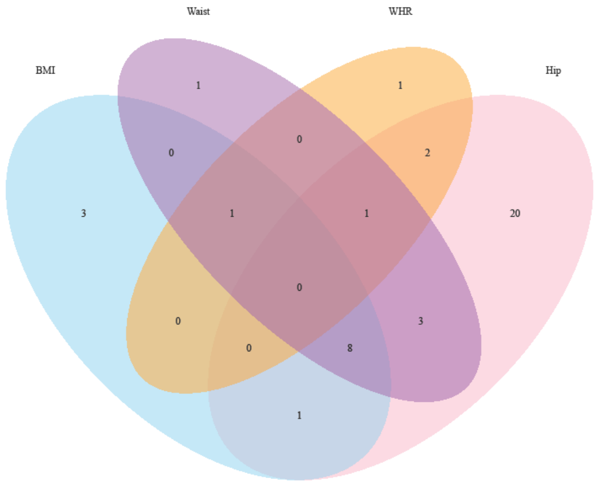
Waist
WHR
BMI
Hip
1
1
0
0
2
3
1
1
20
0
0
0
8
3
1

D

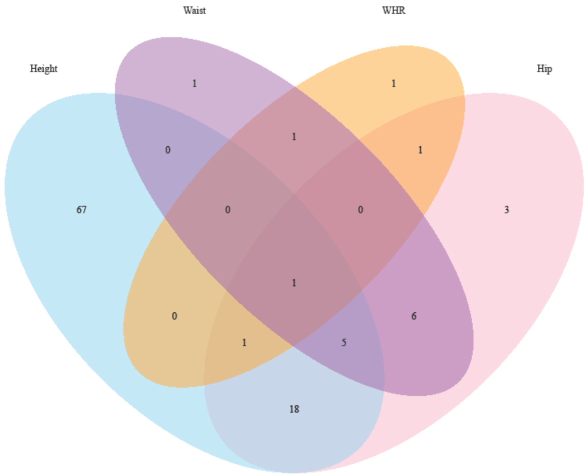
Waist
WHR
Height
Hip
1
1
1
0
1
67
0
0
3
1
0
1
5
6
18

E

F

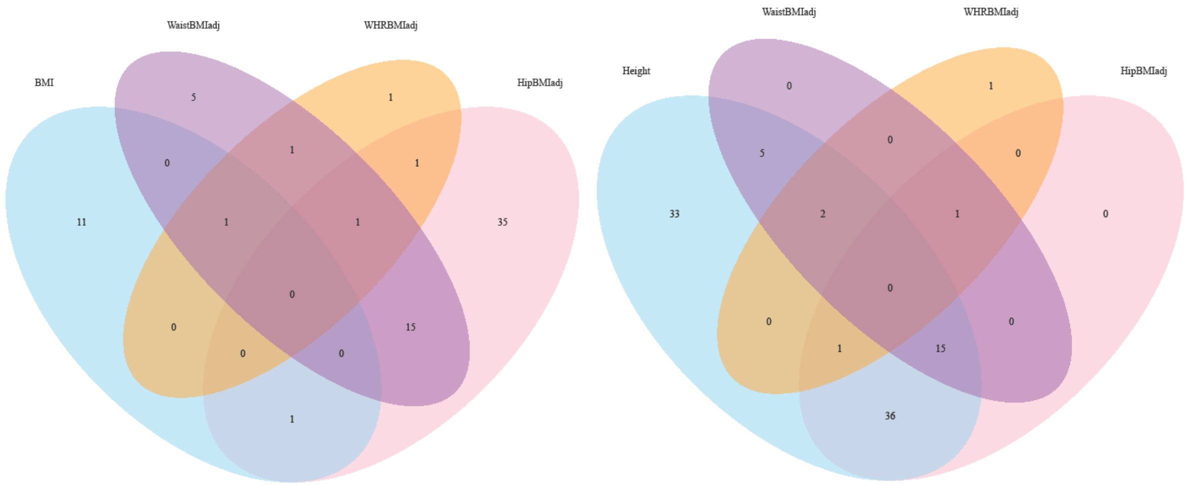

Figure S26: Venn diagrams showing overlap of the 106 signals from Tables 1, 2 and S3 robustly associated with an anthropometric trait at *P-value* ≤ 5x10^{-8} in stage1+stage2 with other anthropometric traits also associated at *P-value* ≤ 5x10^{-8} in stage1+stage2.

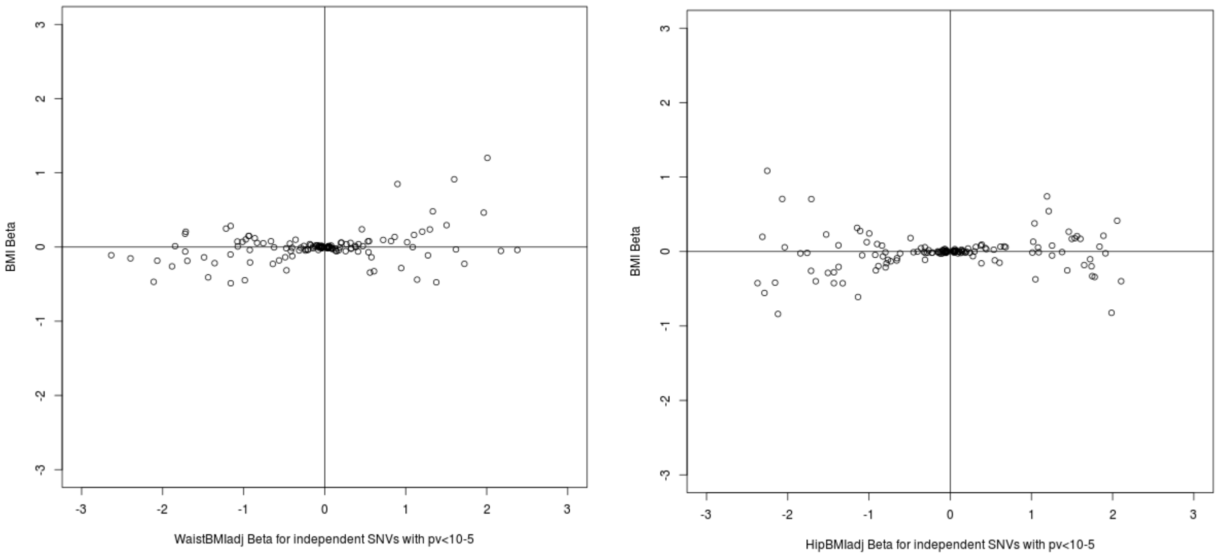

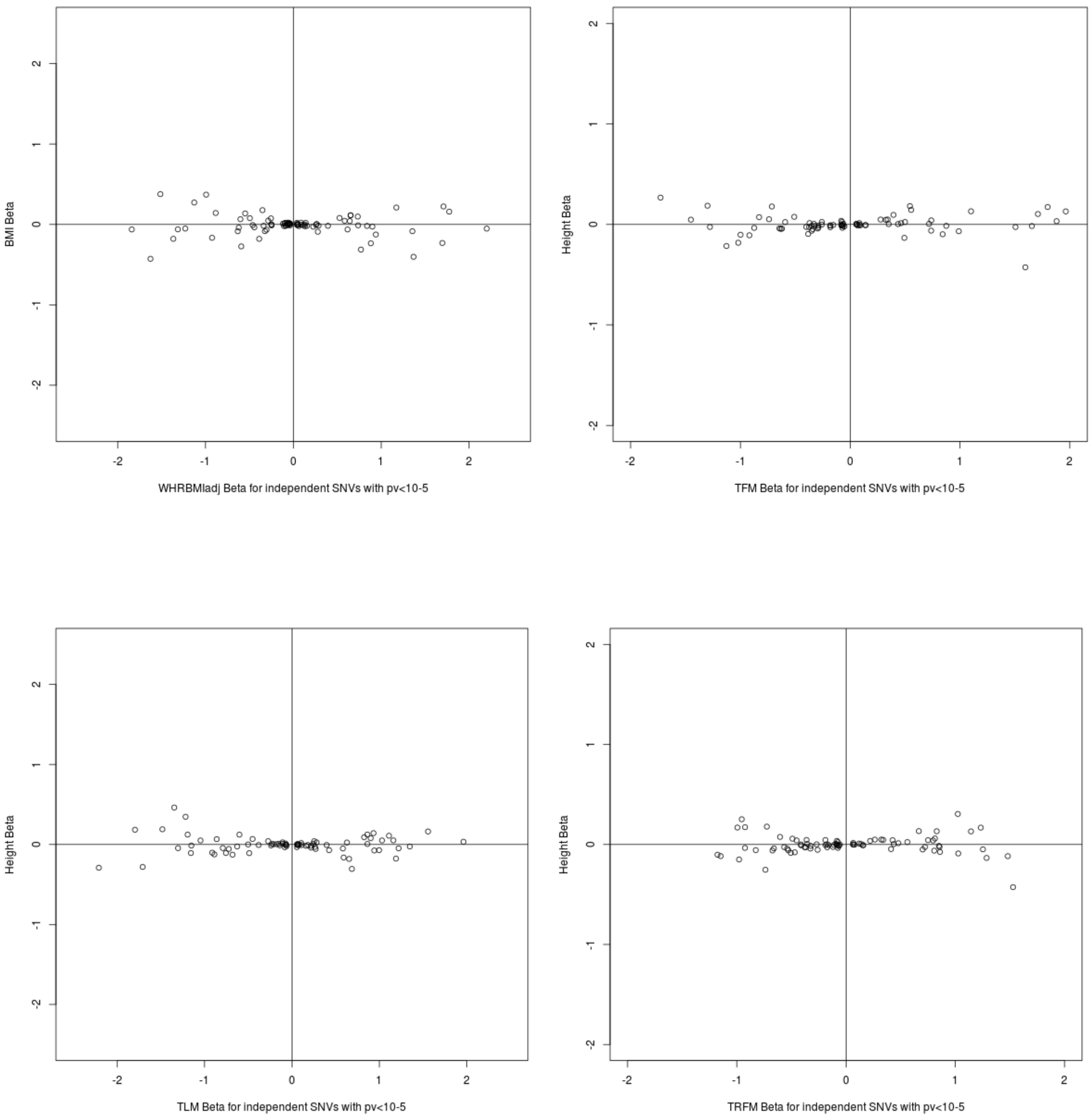

Figure S27: No evidence of collider bias for traits adjusted for BMI or Height.
Effect sizes of independent (pairwise $r^2<0.2$ and further than 500kb) SNPs suggestive ($P<10^{-5}$) for waist circumference, hip circumference and waist to hip ratio adjusted for BMI (R-squared 0.11, 0.0051, 0.004 respectively) versus effect sizes for BMI. Similarly, effect sizes of independent SNPs suggestive for total fat mass, total lean mass and trunk fat mass (R-squared 0.0003, 0.0007, 0.0011 respectively) versus effect sizes for height.

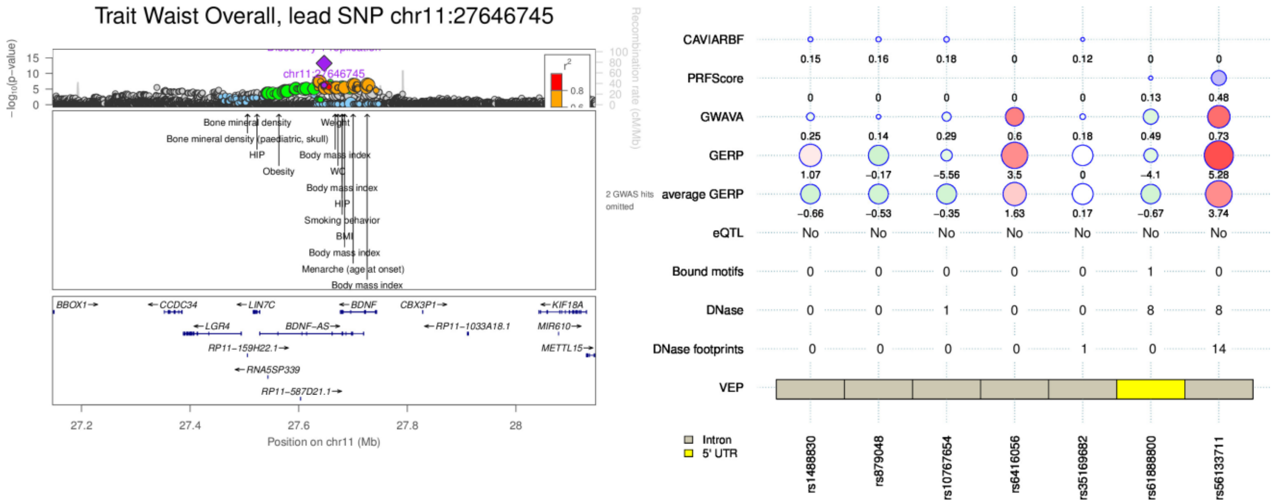
Trait Waist Overall, lead SNP chr11:27646745
Position on chr11 (Mb)
2 GWAS hits omitted
CAVIARBF
PRFScore
GWAVA
GERP
average GERP
eQTL
Bound motifs
DNase
DNase footprints
VEP
Intron
5' UTR
rs1488830
rs879048
rs10767654
rs6416056
rs35169682
rs61888800
rs56133711

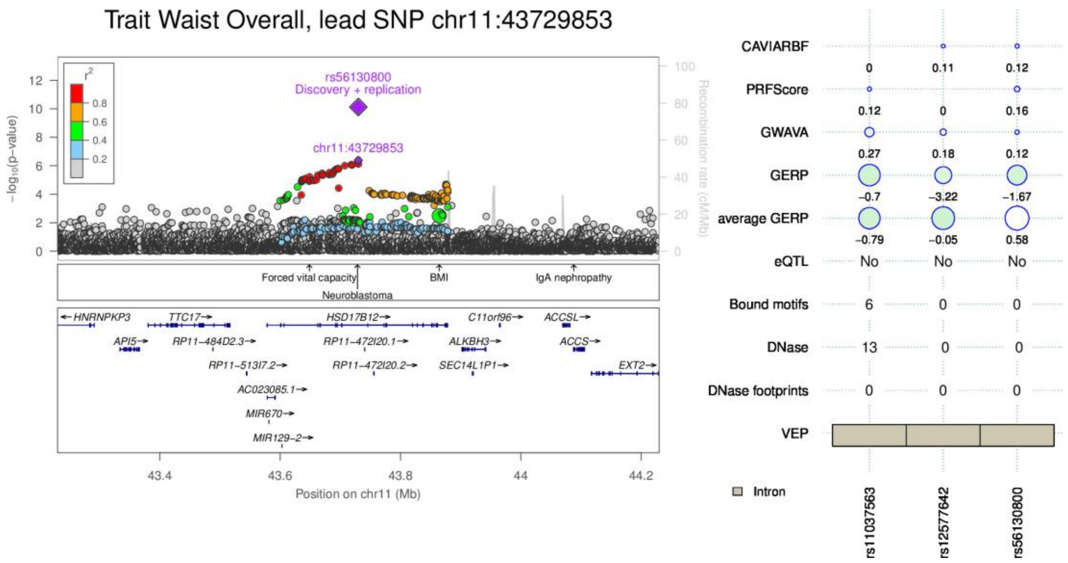
Trait Waist Overall, lead SNP chr11:43729853
rs56130800
Discovery + replication
chr11:43729853
Forced vital capacity
Neuroblastoma
BMI
IgA nephropathy
Position on chr11 (Mb)
CAVIARBF
PRFScore
GWAVA
GERP
average GERP
eQTL
Bound motifs
DNase
DNase footprints
VEP
Intron
rs11037563
rs12577642
rs56130800

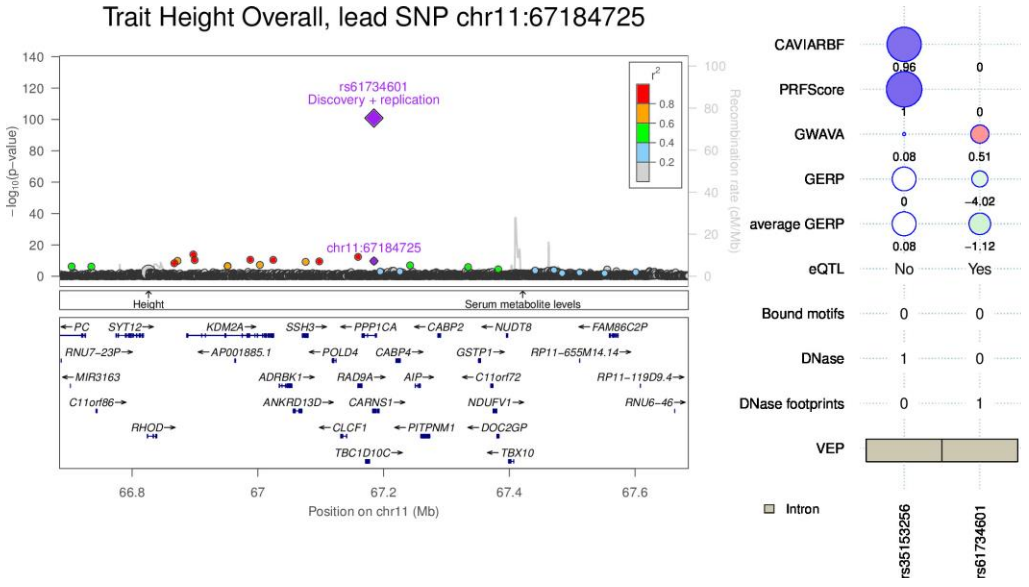
Trait Height Overall, lead SNP chr11:67184725
rs61734601
Discovery + replication
chr11:67184725
Height
Serum metabolite levels
Position on chr11 (Mb)
CAVIARBF
PRFScore
GWAVA
GERP
average GERP
eQTL
Bound motifs
DNase
DNase footprints
VEP
Intron
rs35153256
rs61734601

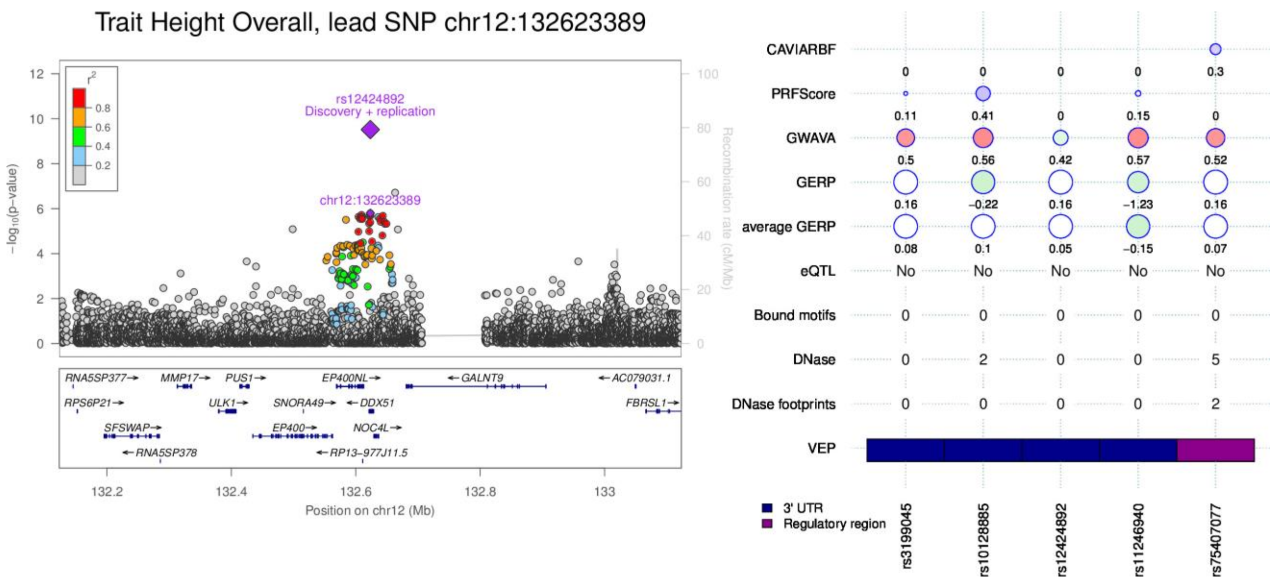
Trait Height Overall, lead SNP chr12:132623389
rs12424892
Discovery + replication
chr12:132623389
−log10(p-value)
Recombination rate (cM/Mb)
Position on chr12 (Mb)
CAVIARBF
PRFScore
GWAVA
GERP
average GERP
eQTL
Bound motifs
DNase
DNase footprints
VEP
3' UTR
Regulatory region
rs3199045
rs10128885
rs12424892
rs11246940
rs75407077

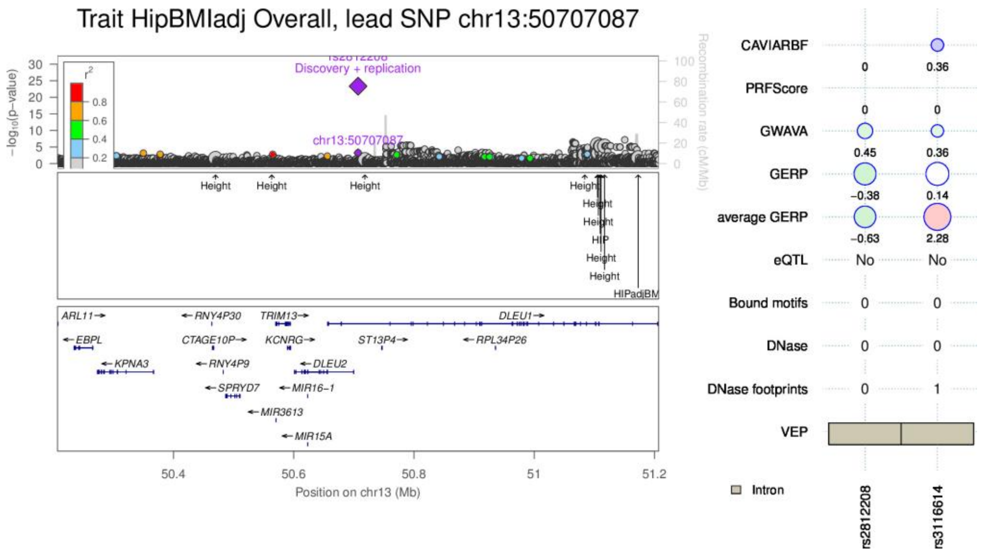
Trait HipBMIadj Overall, lead SNP chr13:50707087
Discovery + replication
chr13:50707087
−log10(p-value)
Recombination rate (cM/Mb)
Position on chr13 (Mb)
CAVIARBF
PRFScore
GWAVA
GERP
average GERP
eQTL
Bound motifs
DNase
DNase footprints
VEP
Intron
rs2812208
rs3116614

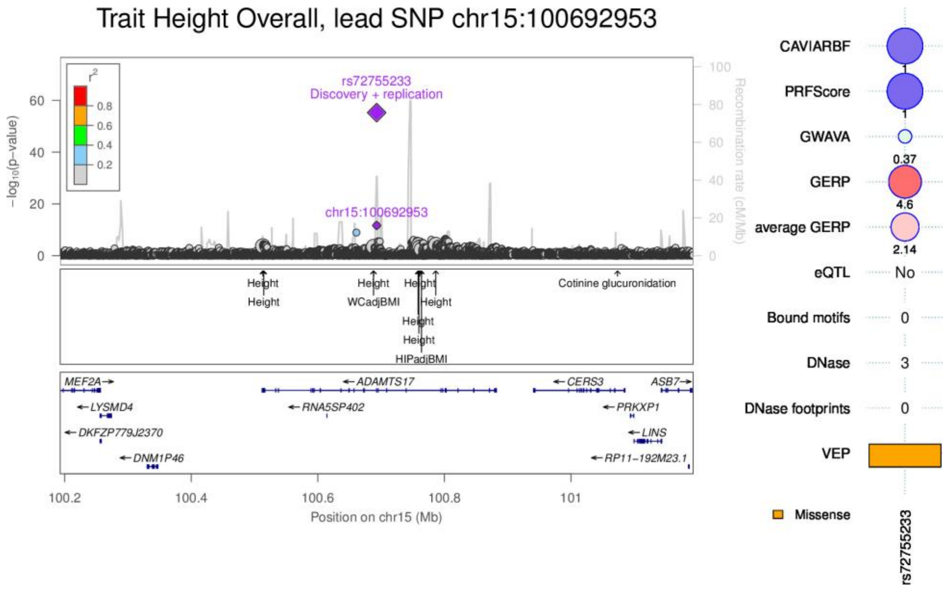
Trait Height Overall, lead SNP chr15:100692953
rs72755233
Discovery + replication
chr15:100692953
−log10(p-value)
Recombination rate (cM/Mb)
Position on chr15 (Mb)
Cotinine glucuronidation
CAVIARBF
PRFScore
GWAVA
GERP
average GERP
eQTL
Bound motifs
DNase
DNase footprints
VEP
Missense
rs72755233

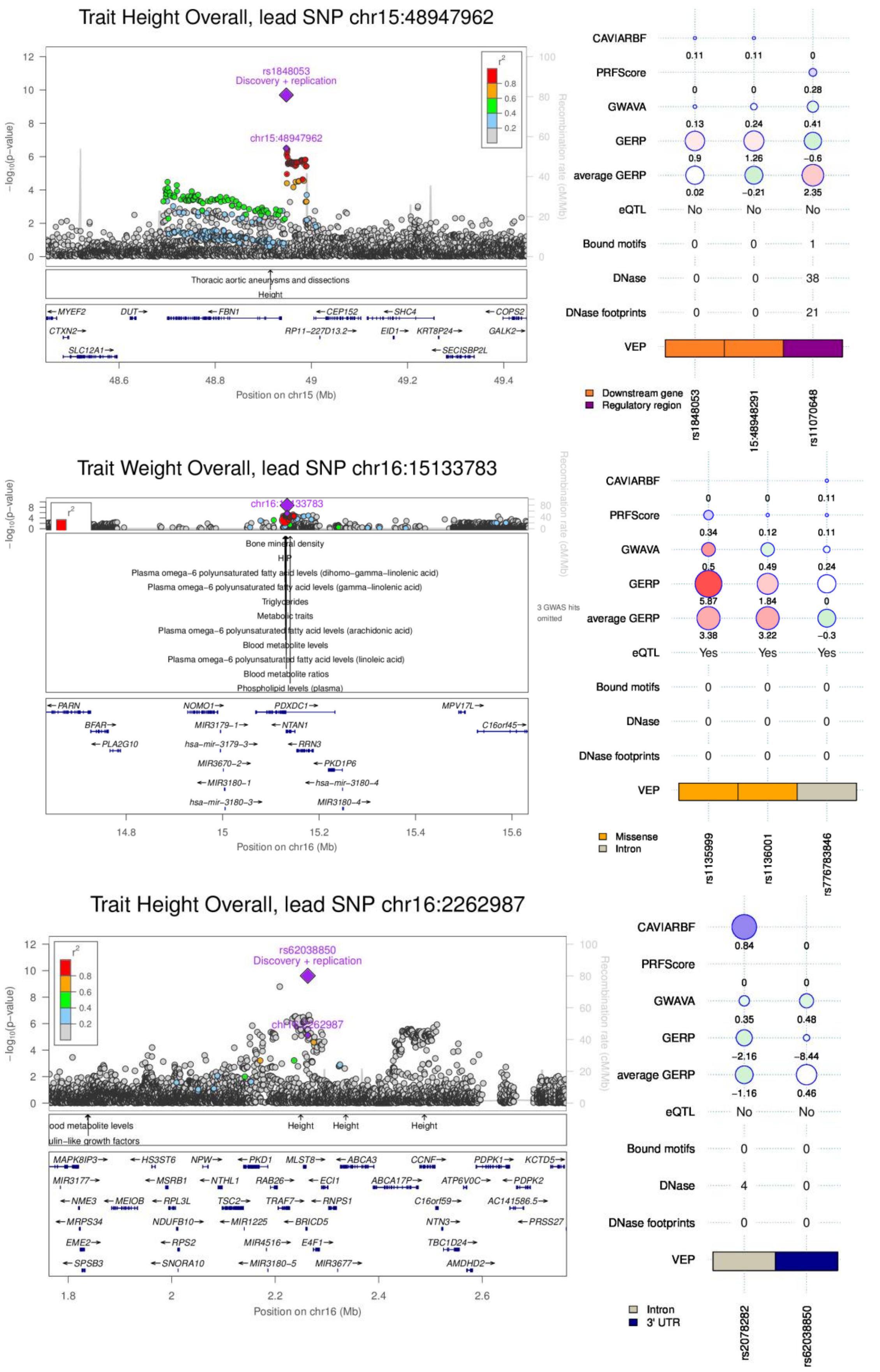

Trait Height Overall, lead SNP chr15:48947962
rs1848053
Discovery + replication
chr15:48947962
Thoracic aortic aneurysms and dissections
Height
Position on chr15 (Mb)
−log10(p−value)
Recombination rate (cM/Mb)
CAVIARBF
PRFScore
GWAVA
GERP
average GERP
eQTL
Bound motifs
DNase
DNase footprints
VEP
Downstream gene
Regulatory region
rs1848053
15:48948291
rs11070648
Trait Weight Overall, lead SNP chr16:15133783
Bone mineral density
HIP
Plasma omega−6 polyunsaturated fatty acid levels (dihomo−gamma−linolenic acid)
Plasma omega−6 polyunsaturated fatty acid levels (gamma−linolenic acid)
Triglycerides
Metabolic traits
Plasma omega−6 polyunsaturated fatty acid levels (arachidonic acid)
Blood metabolite levels
Plasma omega−6 polyunsaturated fatty acid levels (linoleic acid)
Blood metabolite ratios
Phospholipid levels (plasma)
3 GWAS hits omitted
Position on chr16 (Mb)
Missense
Intron
rs1135999
rs1136001
rs776783846
Trait Height Overall, lead SNP chr16:2262987
rs62038850
Discovery + replication
Height
Position on chr16 (Mb)
Intron
3' UTR
rs2078282
rs62038850

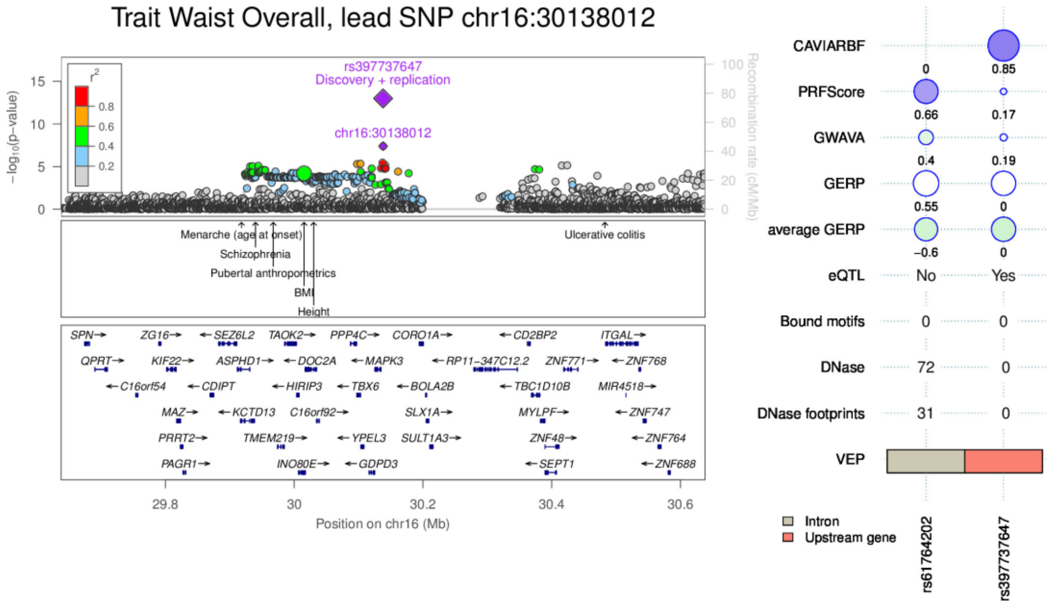

Trait Waist Overall, lead SNP chr16:30138012
rs397737647
Discovery + replication
chr16:30138012
−log10(p−value)
Recombination rate (cM/Mb)
Menarche (age at onset)
Schizophrenia
Pubertal anthropometrics
BMI
Height
Ulcerative colitis
Position on chr16 (Mb)
CAVIARBF
PRFScore
GWAVA
GERP
average GERP
eQTL
Bound motifs
DNase
DNase footprints
VEP
Intron
Upstream gene
rs61764202
rs397737647

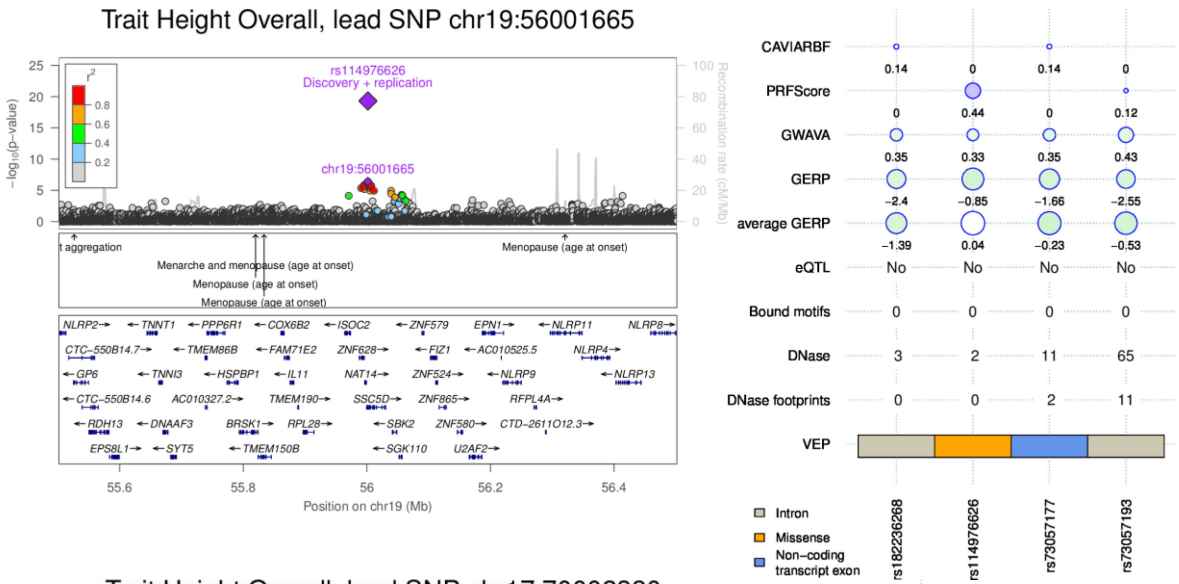

Trait Height Overall, lead SNP chr19:56001665
rs114976626
Discovery + replication
chr19:56001665
−log10(p−value)
Recombination rate (cM/Mb)
Menarche and menopause (age at onset)
Menopause (age at onset)
Position on chr19 (Mb)
CAVIARBF
PRFScore
GWAVA
GERP
average GERP
eQTL
Bound motifs
DNase
DNase footprints
VEP
Intron
Missense
Non−coding transcript exon
rs182236268
rs114976626
rs73057177
rs73057193

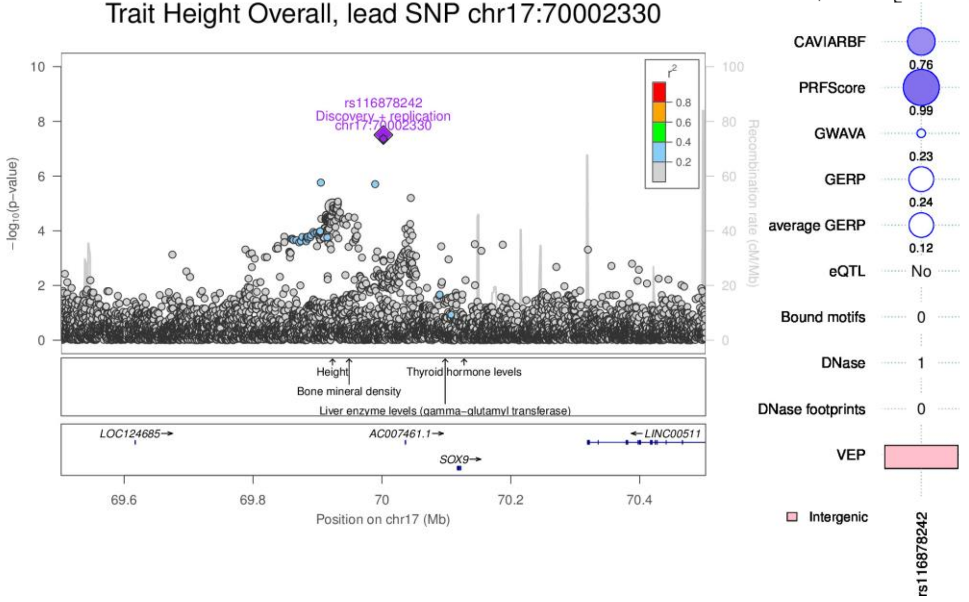

Trait Height Overall, lead SNP chr17:70002330
rs116878242
Discovery + replication
chr17:70002330
−log10(p−value)
Recombination rate (cM/Mb)
Height
Bone mineral density
Thyroid hormone levels
Liver enzyme levels (gamma−glutamyl transferase)
Position on chr17 (Mb)
CAVIARBF
PRFScore
GWAVA
GERP
average GERP
eQTL
Bound motifs
DNase
DNase footprints
VEP
Intergenic
rs116878242

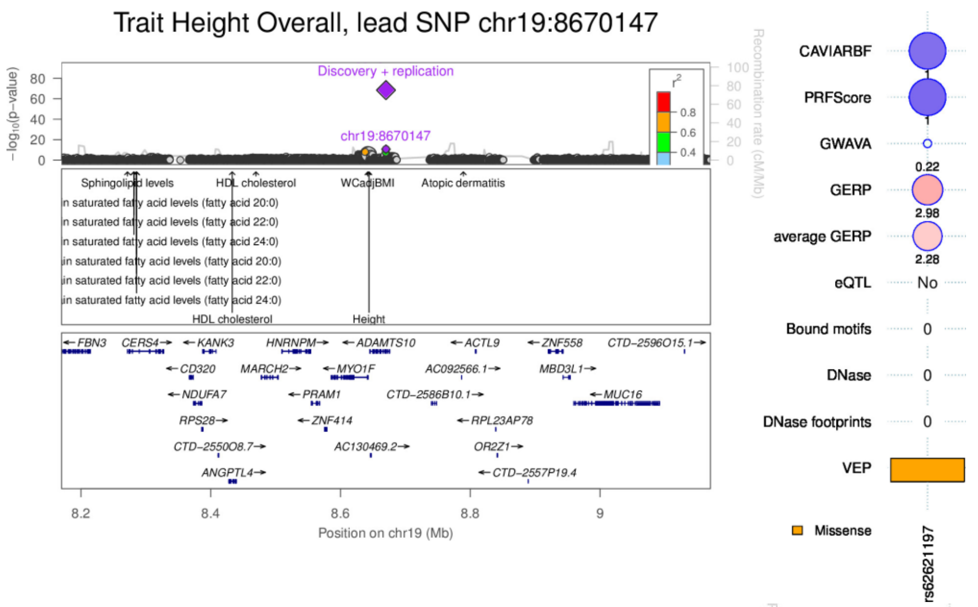
Trait Height Overall, lead SNP chr19:8670147
Discovery + replication
chr19:8670147
−log10(p−value)
Recombination rate (cM/Mb)
Sphingolipid levels
HDL cholesterol
WCadjBMI
Atopic dermatitis
HDL cholesterol
Height
Position on chr19 (Mb)
CAVIARBF
PRFScore
GWAVA
GERP
average GERP
eQTL
Bound motifs
DNase
DNase footprints
VEP
Missense
rs62621197

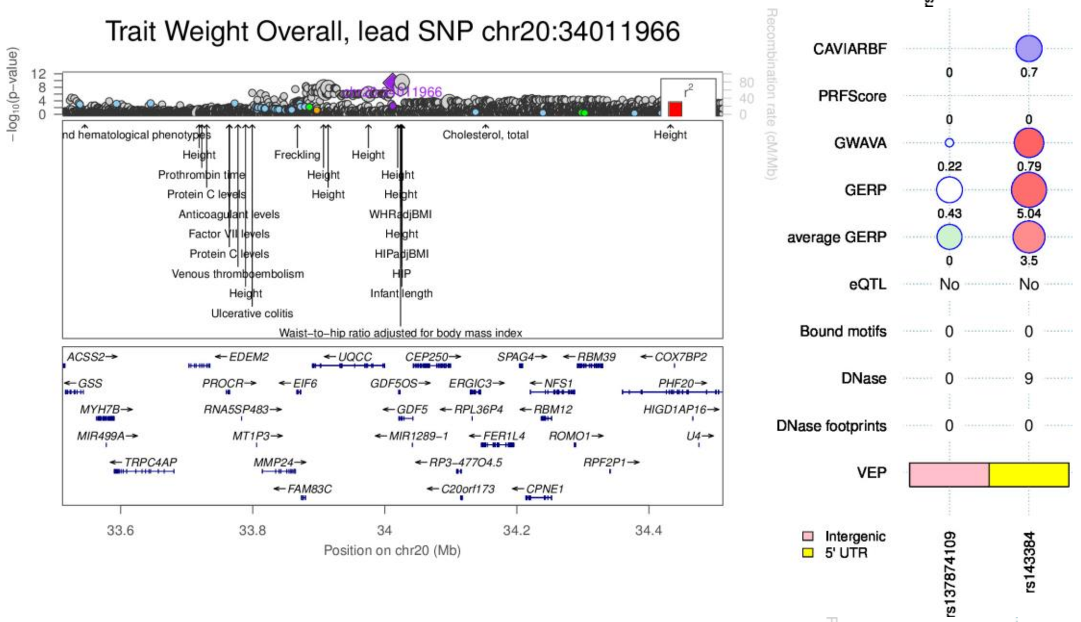
Trait Weight Overall, lead SNP chr20:34011966
−log10(p−value)
Recombination rate (cM/Mb)
Freckling
Height
Prothrombin time
Protein C levels
Anticoagulant levels
Factor VII levels
Venous thromboembolism
Ulcerative colitis
Cholesterol, total
WHRadjBMI
HIPadjBMI
HIP
Infant length
Waist−to−hip ratio adjusted for body mass index
Position on chr20 (Mb)
CAVIARBF
PRFScore
GWAVA
GERP
average GERP
eQTL
Bound motifs
DNase
DNase footprints
VEP
Intergenic
5' UTR
rs137874109
rs143384

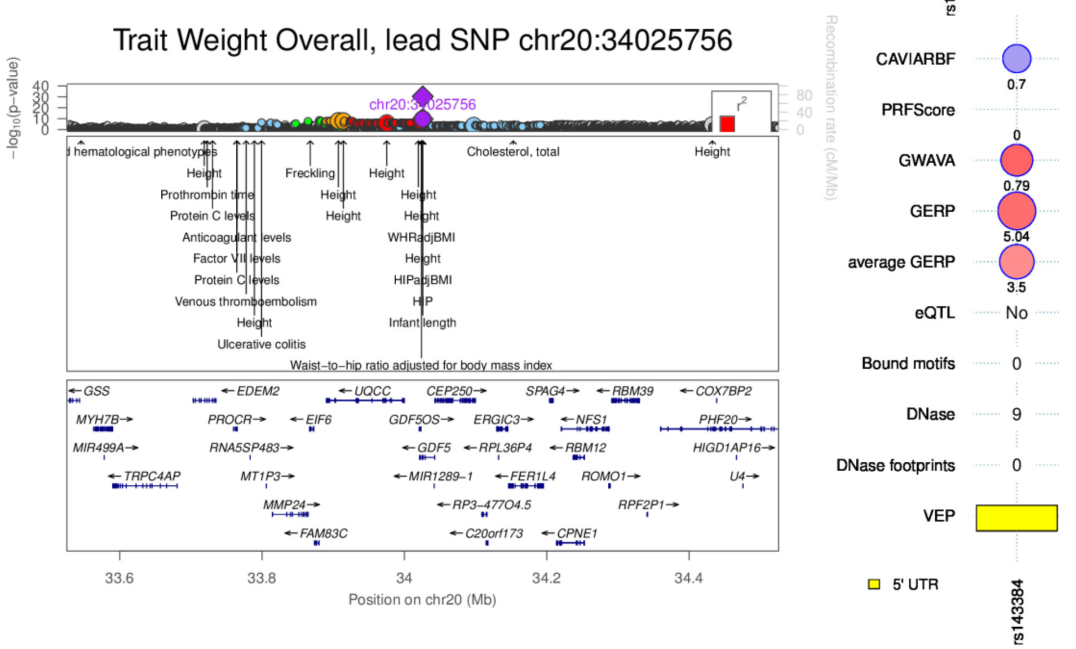
Trait Weight Overall, lead SNP chr20:34025756
−log10(p−value)
Recombination rate (cM/Mb)
Freckling
Height
Prothrombin time
Protein C levels
Anticoagulant levels
Factor VII levels
Venous thromboembolism
Ulcerative colitis
Cholesterol, total
WHRadjBMI
HIPadjBMI
HIP
Infant length
Waist−to−hip ratio adjusted for body mass index
Position on chr20 (Mb)
CAVIARBF
PRFScore
GWAVA
GERP
average GERP
eQTL
Bound motifs
DNase
DNase footprints
VEP
5' UTR
rs143384

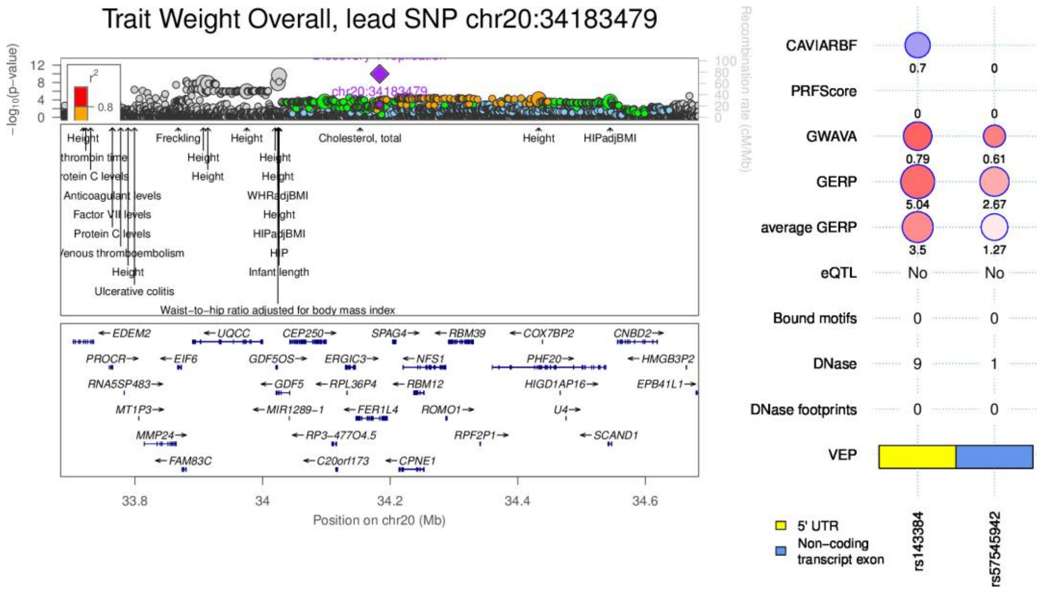
Trait Weight Overall, lead SNP chr20:34183479
chr20:34183479
Position on chr20 (Mb)
CAVIARBF
PRFScore
GWAVA
GERP
average GERP
eQTL
Bound motifs
DNase
DNase footprints
VEP
5' UTR
Non-coding transcript exon
rs143384
rs57545942

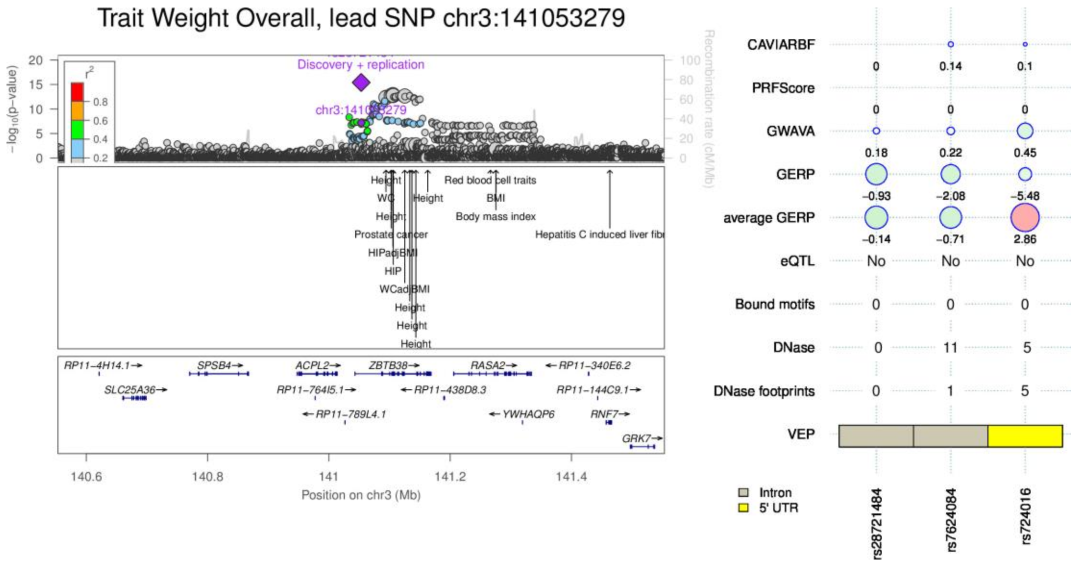
Trait Weight Overall, lead SNP chr3:141053279
Discovery + replication
Position on chr3 (Mb)
CAVIARBF
PRFScore
GWAVA
GERP
average GERP
eQTL
Bound motifs
DNase
DNase footprints
VEP
Intron
5' UTR
rs28721484
rs7624084
rs724016

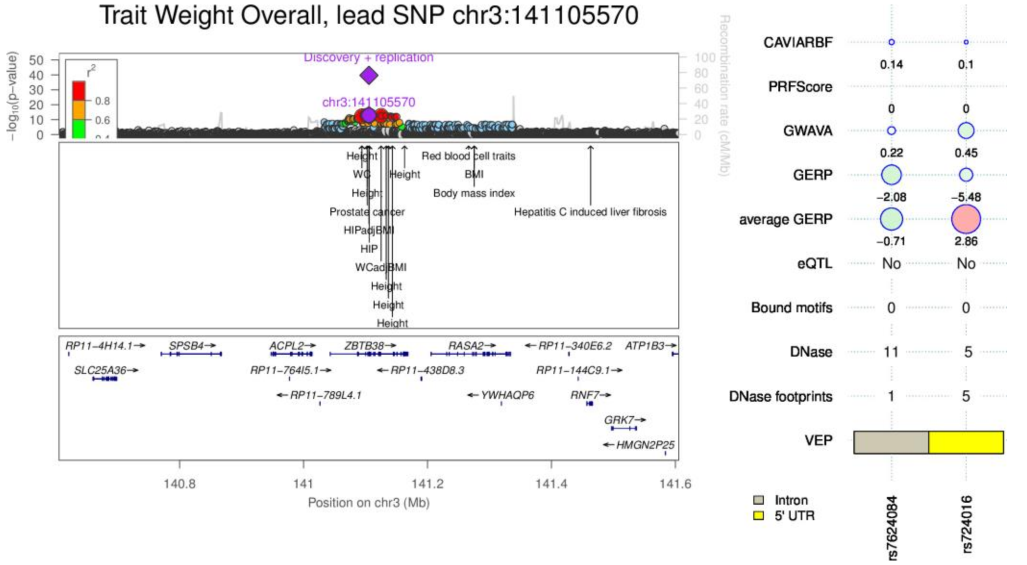
Trait Weight Overall, lead SNP chr3:141105570
Discovery + replication
chr3:141105570
Position on chr3 (Mb)
CAVIARBF
PRFScore
GWAVA
GERP
average GERP
eQTL
Bound motifs
DNase
DNase footprints
VEP
Intron
5' UTR
rs7624084
rs724016

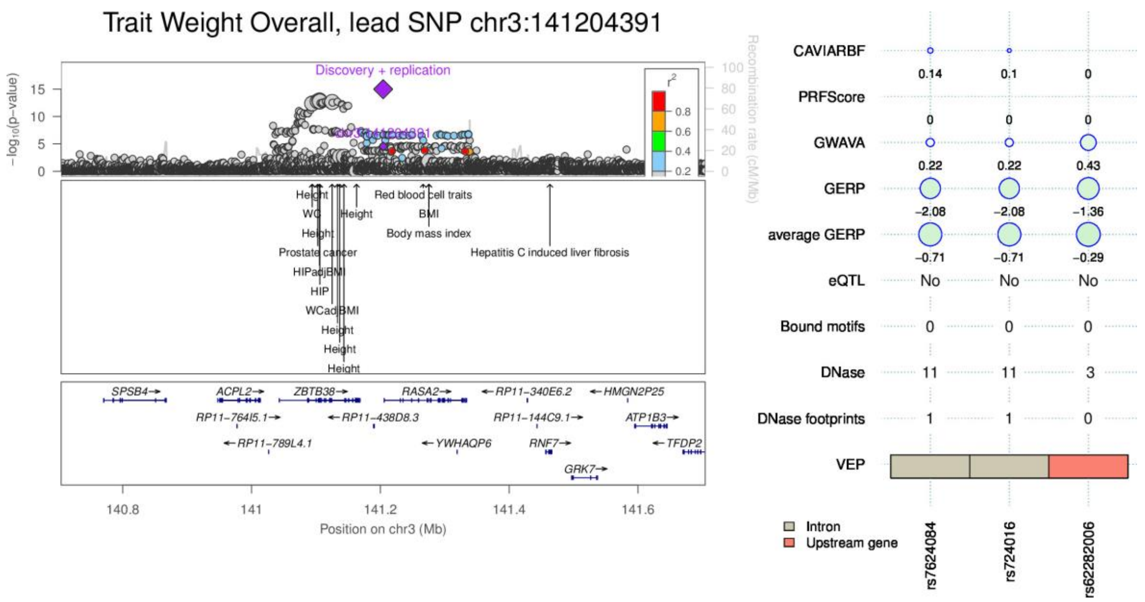

Trait Weight Overall, lead SNP chr3:141204391
Discovery + replication
−log10(p−value)
Recombination rate (cM/Mb)
Position on chr3 (Mb)
CAVIARBF
PRFScore
GWAVA
GERP
average GERP
eQTL
Bound motifs
DNase
DNase footprints
VEP
Intron
Upstream gene
rs7624084
rs724016
rs62282006

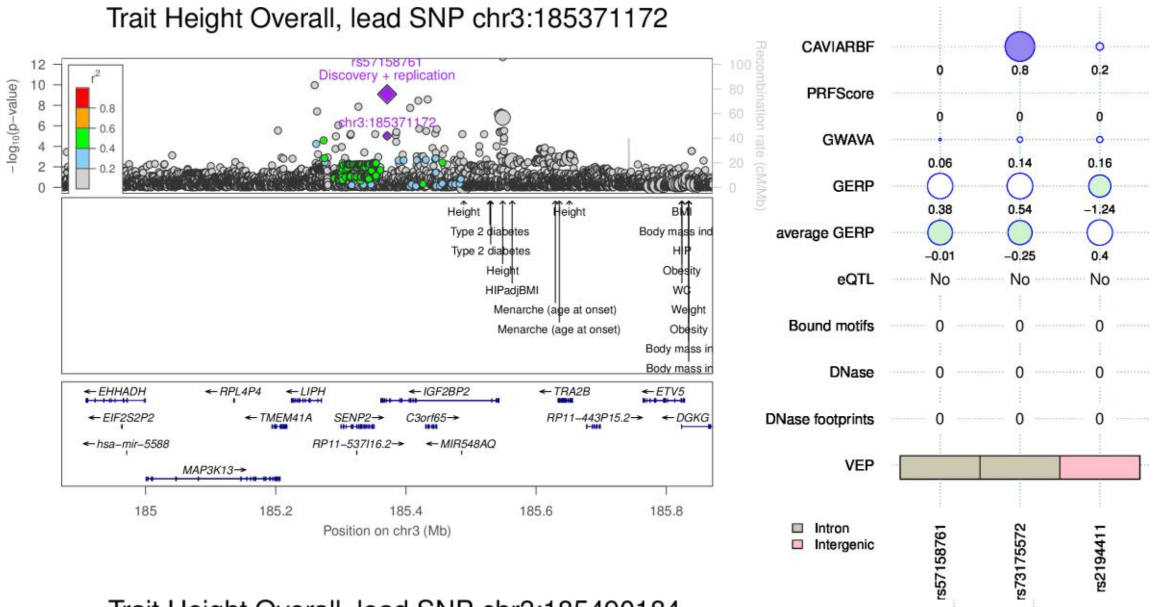

Trait Height Overall, lead SNP chr3:185371172
rs57158761
Discovery + replication
chr3:185371172
−log10(p−value)
Recombination rate (cM/Mb)
Position on chr3 (Mb)
CAVIARBF
PRFScore
GWAVA
GERP
average GERP
eQTL
Bound motifs
DNase
DNase footprints
VEP
Intron
Intergenic
rs57158761
rs73175572
rs2194411

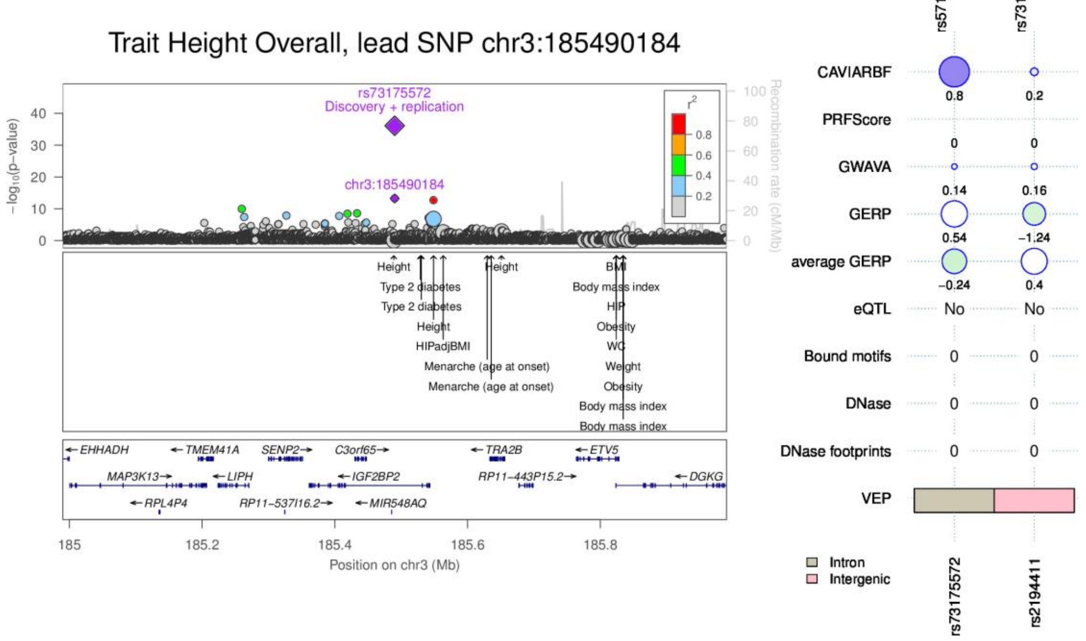

Trait Height Overall, lead SNP chr3:185490184
rs73175572
Discovery + replication
chr3:185490184
−log10(p−value)
Recombination rate (cM/Mb)
Position on chr3 (Mb)
CAVIARBF
PRFScore
GWAVA
GERP
average GERP
eQTL
Bound motifs
DNase
DNase footprints
VEP
Intron
Intergenic
rs73175572
rs2194411

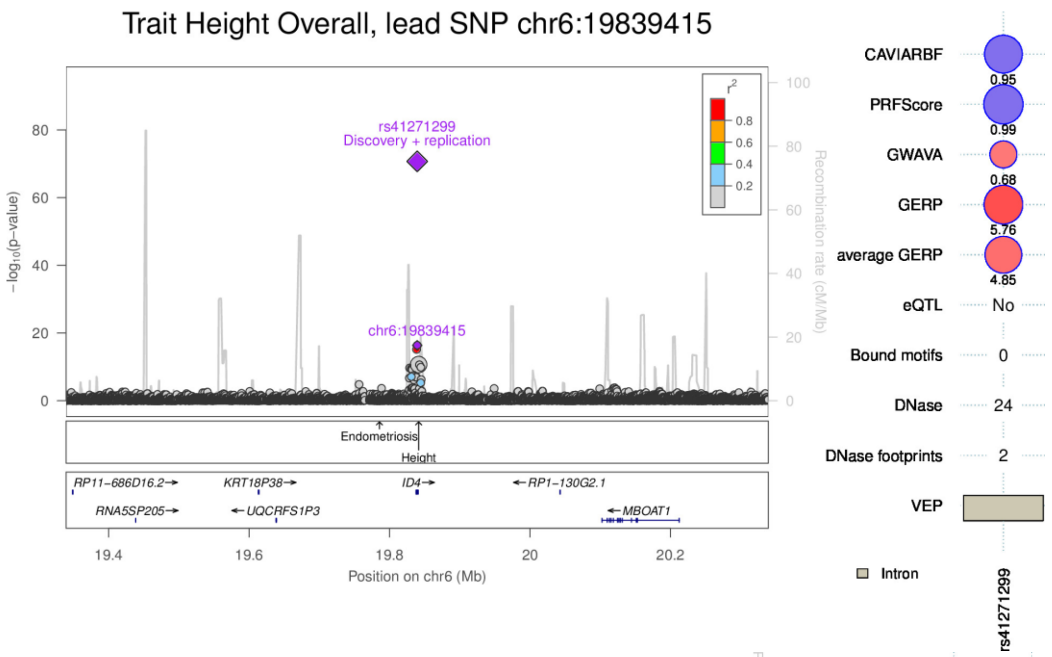
Trait Height Overall, lead SNP chr6:19839415
rs41271299
Discovery + replication
chr6:19839415
−log10(p−value)
Recombination rate (cM/Mb)
Endometriosis
Height
RP11−686D16.2
KRT18P38
ID4
RP1−130G2.1
RNA5SP205
UQCRFS1P3
MBOAT1
Position on chr6 (Mb)
CAVIARBF
PRFScore
GWAVA
GERP
average GERP
eQTL
Bound motifs
DNase
DNase footprints
VEP
0.95
0.99
0.68
5.76
4.85
No
0
24
2
Intron
rs41271299

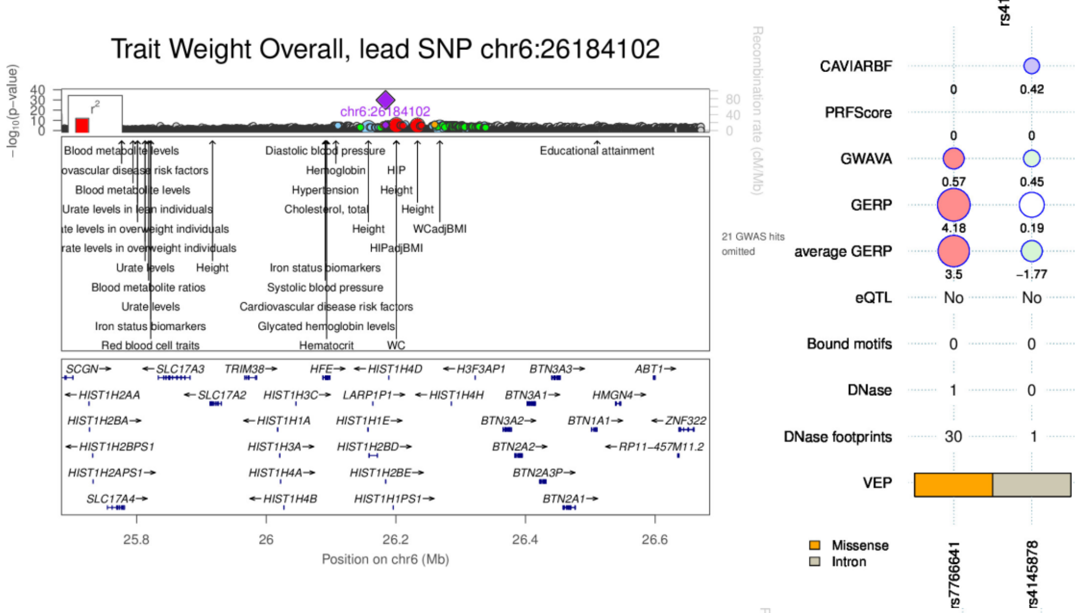
Trait Weight Overall, lead SNP chr6:26184102
chr6:26184102
−log10(p−value)
Recombination rate (cM/Mb)
21 GWAS hits omitted
Blood metabolite levels
Diastolic blood pressure
Educational attainment
Hemoglobin
HIP
Hypertension
Height
Cholesterol, total
WCadjBMI
HIPadjBMI
Iron status biomarkers
Blood metabolite ratios
Systolic blood pressure
Urate levels
Cardiovascular disease risk factors
Glycated hemoglobin levels
Red blood cell traits
Hematocrit
WC
Position on chr6 (Mb)
CAVIARBF
PRFScore
GWAVA
GERP
average GERP
eQTL
Bound motifs
DNase
DNase footprints
VEP
Missense
Intron
rs7766641
rs4145878

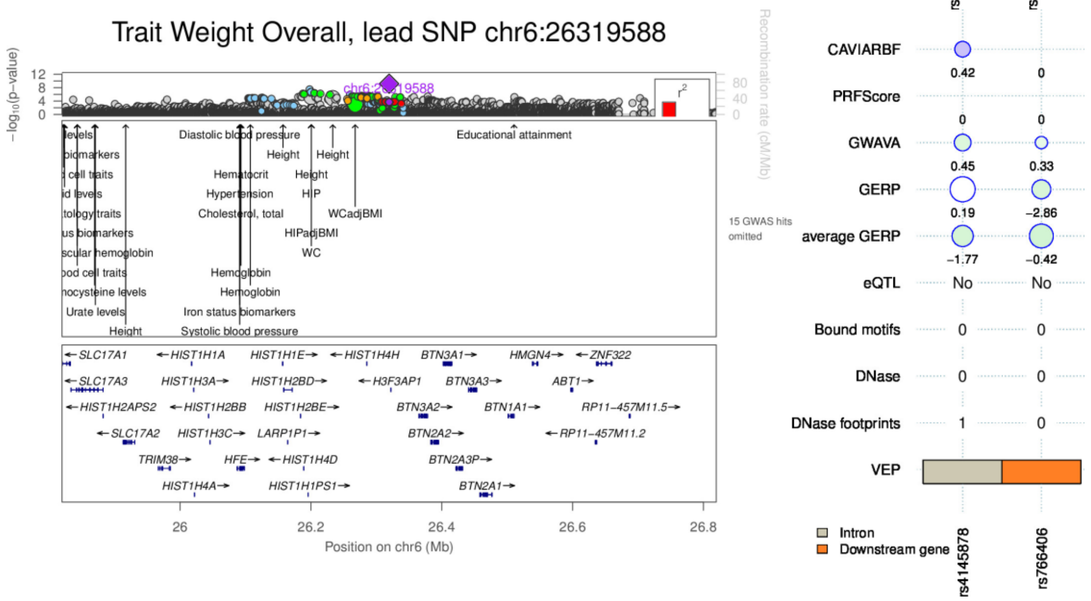
Trait Weight Overall, lead SNP chr6:26319588
−log10(p−value)
Recombination rate (cM/Mb)
15 GWAS hits omitted
Diastolic blood pressure
Educational attainment
Height
Hematocrit
Hypertension
HIP
Cholesterol, total
WCadjBMI
HIPadjBMI
WC
Hemoglobin
Iron status biomarkers
Systolic blood pressure
Urate levels
Position on chr6 (Mb)
CAVIARBF
PRFScore
GWAVA
GERP
average GERP
eQTL
Bound motifs
DNase
DNase footprints
VEP
Intron
Downstream gene
rs4145878
rs766406

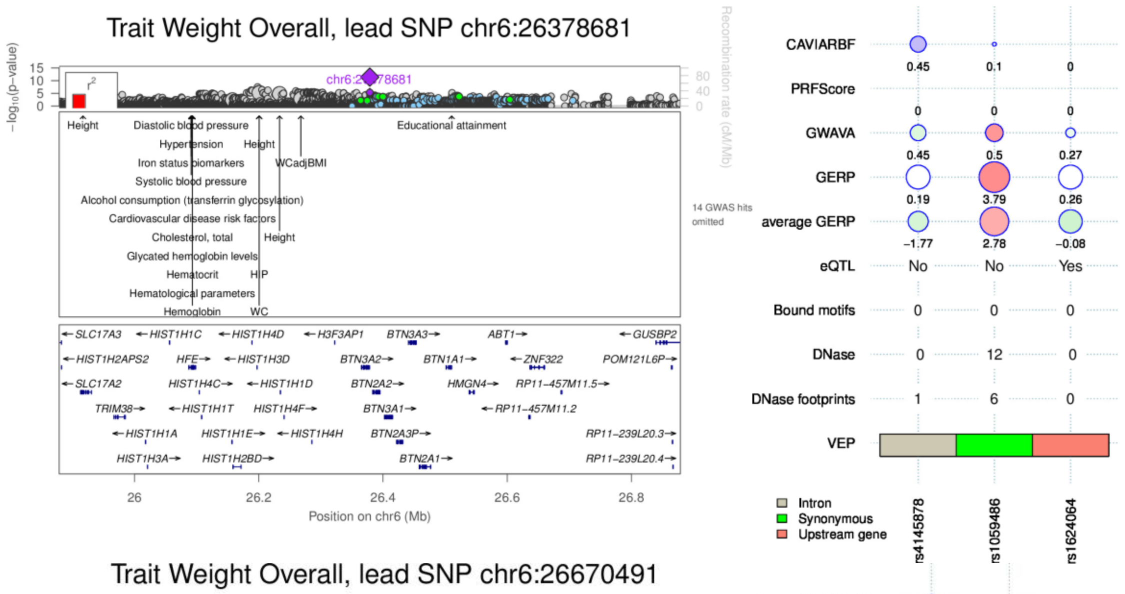
Trait Weight Overall, lead SNP chr6:26378681
chr6:26378681
−log10(p−value)
Recombination rate (cM/Mb)
14 GWAS hits omitted
Height
Diastolic blood pressure
Hypertension
Height
Iron status biomarkers
WCadjBMI
Systolic blood pressure
Alcohol consumption (transferrin glycosylation)
Cardiovascular disease risk factors
Cholesterol, total
Height
Glycated hemoglobin levels
Hematocrit
HIP
Hematological parameters
Hemoglobin
WC
Educational attainment
SLC17A3
HIST1H1C
HIST1H4D
H3F3AP1
BTN3A3
ABT1
GUSBP2
HIST1H2APS2
HFE
HIST1H3D
BTN3A2
BTN1A1
ZNF322
POM121L6P
SLC17A2
HIST1H4C
HIST1H1D
BTN2A2
HMGN4
RP11−457M11.5
TRIM38
HIST1H1T
HIST1H4F
BTN3A1
RP11−457M11.2
HIST1H1A
HIST1H1E
HIST1H4H
BTN2A3P
RP11−239L20.3
HIST1H3A
HIST1H2BD
BTN2A1
RP11−239L20.4
26
26.2
26.4
26.6
26.8
Position on chr6 (Mb)
CAVIARBF
PRFScore
GWAVA
GERP
average GERP
eQTL
Bound motifs
DNase
DNase footprints
VEP
0.45
0.1
0
0
0
0
0.45
0.5
0.27
0.19
3.79
0.26
−1.77
2.78
−0.08
No
No
Yes
0
0
0
0
12
0
1
6
0
Intron
Synonymous
Upstream gene
rs4145878
rs1059486
rs1624064

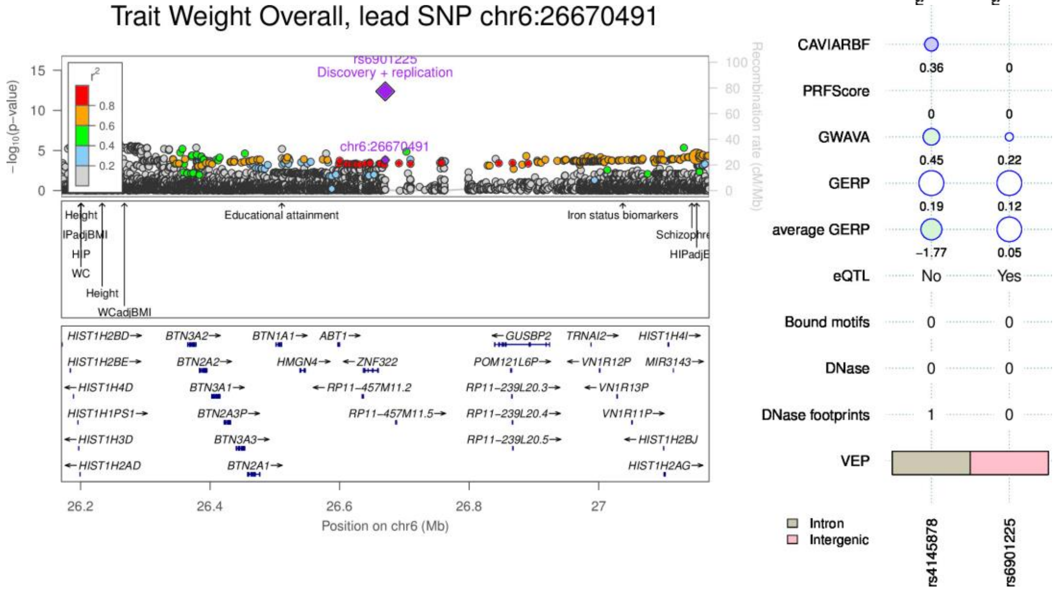
Trait Weight Overall, lead SNP chr6:26670491
rs6901225
Discovery + replication
chr6:26670491
−log10(p−value)
Recombination rate (cM/Mb)
Height
IPadjBMI
HIP
WC
Height
WCadjBMI
Educational attainment
Iron status biomarkers
Schizophre
HIPadjB
HIST1H2BD
BTN3A2
BTN1A1
ABT1
GUSBP2
TRNAI2
HIST1H4I
HIST1H2BE
BTN2A2
HMGN4
ZNF322
POM121L6P
VN1R12P
MIR3143
HIST1H4D
BTN3A1
RP11−457M11.2
RP11−239L20.3
VN1R13P
HIST1H1PS1
BTN2A3P
RP11−457M11.5
RP11−239L20.4
VN1R11P
HIST1H3D
BTN3A3
RP11−239L20.5
HIST1H2BJ
HIST1H2AD
BTN2A1
HIST1H2AG
26.2
26.4
26.6
26.8
27
Position on chr6 (Mb)
CAVIARBF
PRFScore
GWAVA
GERP
average GERP
eQTL
Bound motifs
DNase
DNase footprints
VEP
0.36
0
0
0
0.45
0.22
0.19
0.12
−1.77
0.05
No
Yes
0
0
0
0
1
0
Intron
Intergenic
rs4145878
rs6901225

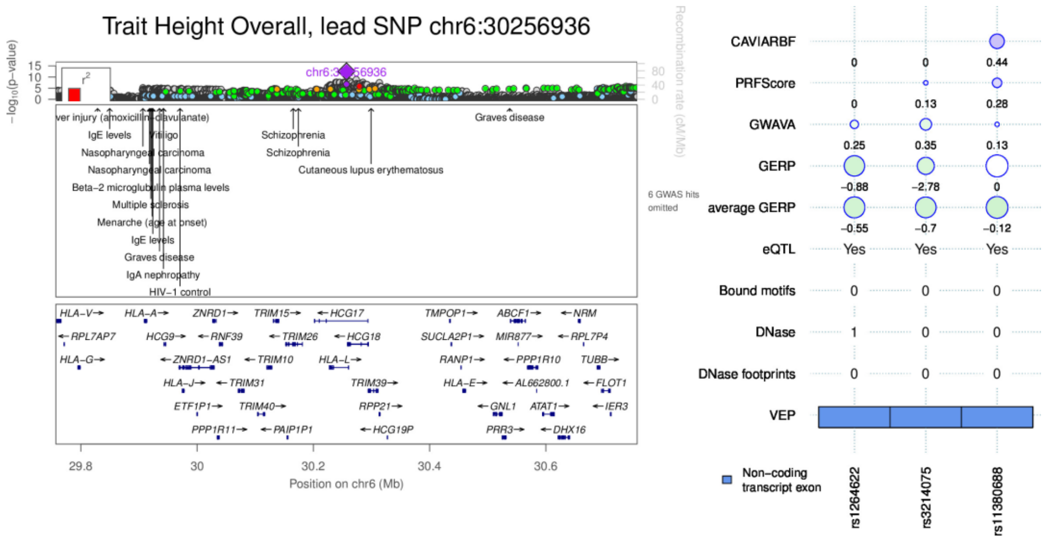
Trait Height Overall, lead SNP chr6:30256936
chr6:30256936
−log10(p−value)
Recombination rate (cM/Mb)
6 GWAS hits omitted
ver injury (amoxicillin-clavulanate)
IgE levels
Vitiligo
Nasopharyngeal carcinoma
Nasopharyngeal carcinoma
Beta−2 microglubulin plasma levels
Multiple sclerosis
Menarche (age at onset)
IgE levels
Graves disease
IgA nephropathy
HIV−1 control
Schizophrenia
Schizophrenia
Cutaneous lupus erythematosus
Graves disease
HLA−V
HLA−A
ZNRD1
TRIM15
HCG17
TMPOP1
ABCF1
NRM
RPL7AP7
HCG9
RNF39
TRIM26
HCG18
SUCLA2P1
MIR877
RPL7P4
HLA−G
ZNRD1−AS1
TRIM10
HLA−L
RANP1
PPP1R10
TUBB
HLA−J
TRIM31
TRIM39
HLA−E
AL662800.1
FLOT1
ETF1P1
TRIM40
RPP21
GNL1
ATAT1
IER3
PPP1R11
PAIP1P1
HCG19P
PRR3
DHX16
29.8
30
30.2
30.4
30.6
Position on chr6 (Mb)
CAVIARBF
PRFScore
GWAVA
GERP
average GERP
eQTL
Bound motifs
DNase
DNase footprints
VEP
0
0
0.44
0
0.13
0.28
0.25
0.35
0.13
−0.88
−2.78
0
−0.55
−0.7
−0.12
Yes
Yes
Yes
0
0
0
1
0
0
0
0
0
Non-coding transcript exon
rs1264622
rs3214075
rs11380688

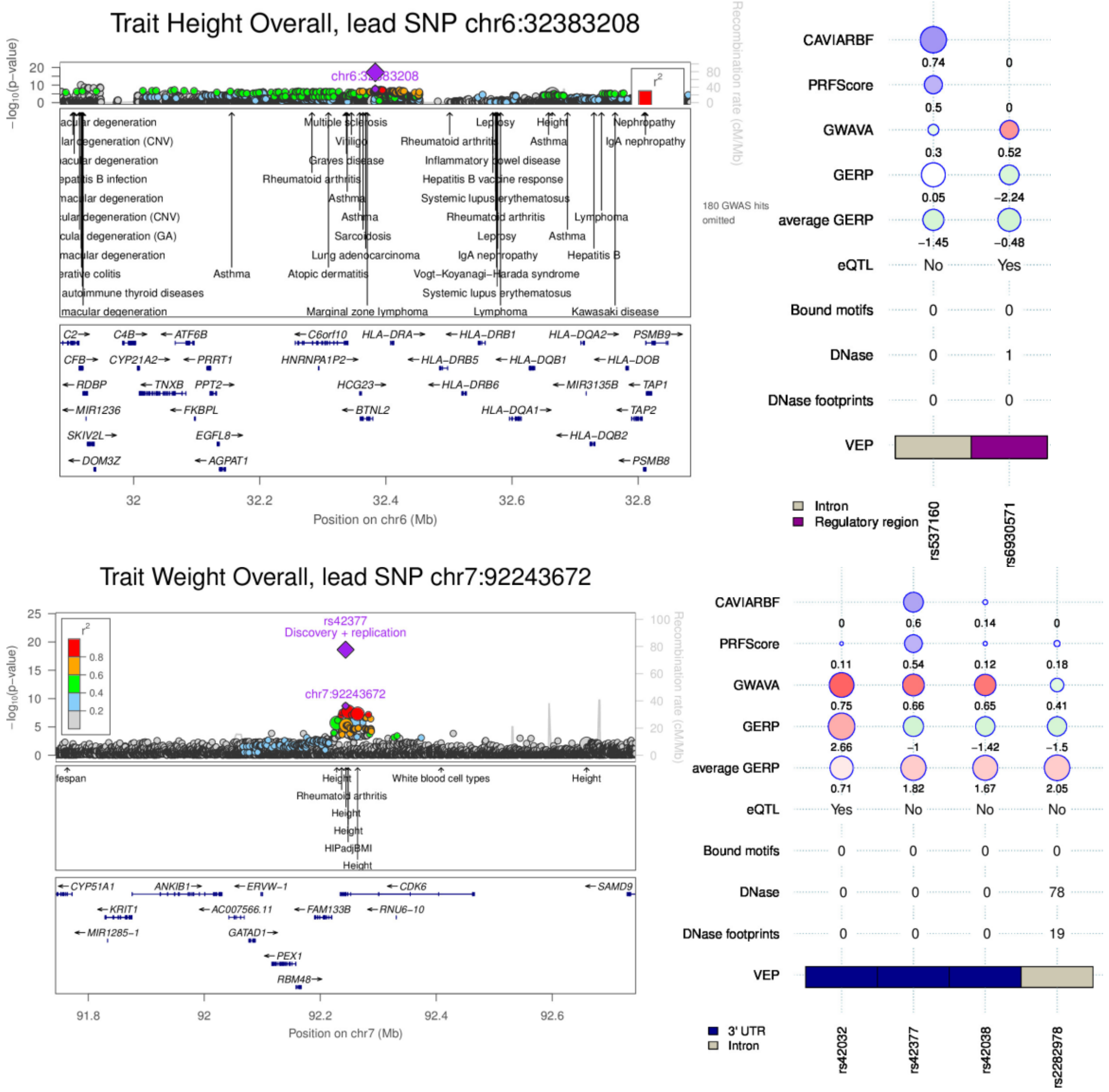

Figure S28: Combined information from two fine-mapping methods, functional prediction scores and eQTL analysis to assess the overall evidence supporting functional and causal interpretation at 30 fine-mapped regions (Table S5) of the 106 newly indentifind variants.

The panels (from top to bottom) show the LocusZoom regional association plot; posterior probability (PP) statistics from the fine-mapping methods CAVIARBF and PRFScore (only variants with PP>0.1 in either methods are shown); Genome Wide Annotation of Variants (GWAVA) scores ; Genomic Evolutionary Rate Profiling (GERP) scores; average GERP (in a 100bp window around each variant) scores; if the variant is an eQTL signal; number of cell lines in which the variant overlaps with a DNase footprints (peak calls from ENCODE); number of overlapping transcriptional factor binding sites based on ENCODE and JASPAR ChIP-seq; number of cell lines in which the queried locus overlaps with a DNase hypersensitivity site (ENCODE data, peaks from Ensembl); and Variant Effect Predictor (VEP) genic annotation. Circle sizes and colors for all scores are scaled with respect to score type and numbers are plotted below each circle. GWAVA scores range between [0,1] and scores over 0.5 indicate functionality (coloured in shades of green for scores <0.5 and red for scores >0.5). GERP scores range between [-12.3,6.17] and scores above zero indicate constrain (coloured in shades of green for scores <0 and red for scores >0).

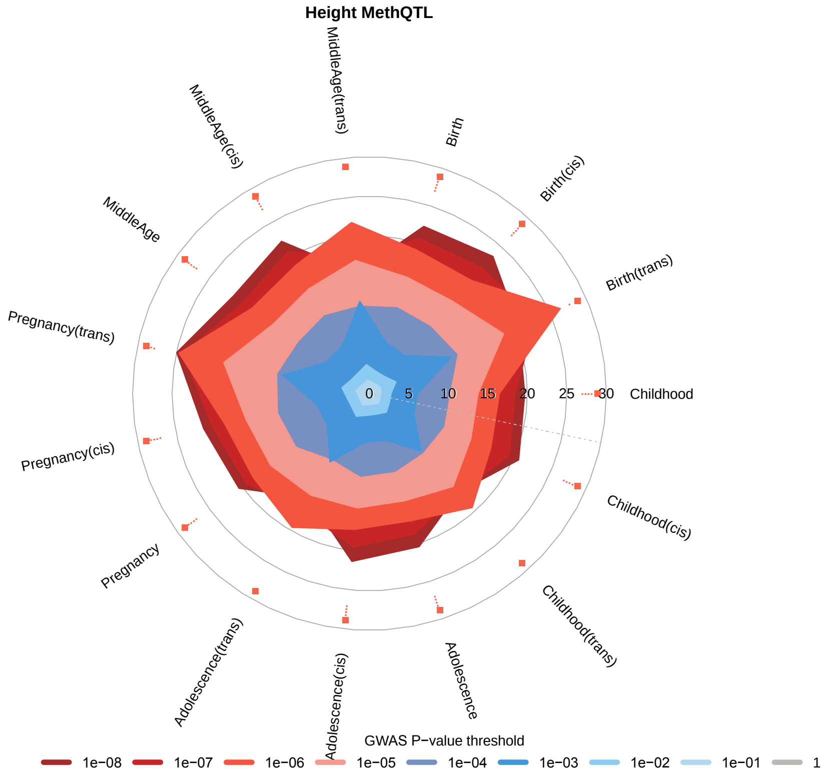

Height MethQTL
MiddleAge(trans)
Birth
Birth(cis)
Birth(trans)
Childhood
Childhood(cis)
Childhood(trans)
Adolescence
Adolescence(cis)
Adolescence(trans)
Pregnancy
Pregnancy(cis)
Pregnancy(trans)
MiddleAge
MiddleAge(cis)
0
5
10
15
20
25
30
GWAS P−value threshold
1e−08
1e−07
1e−06
1e−05
1e−04
1e−03
1e−02
1e−01
1

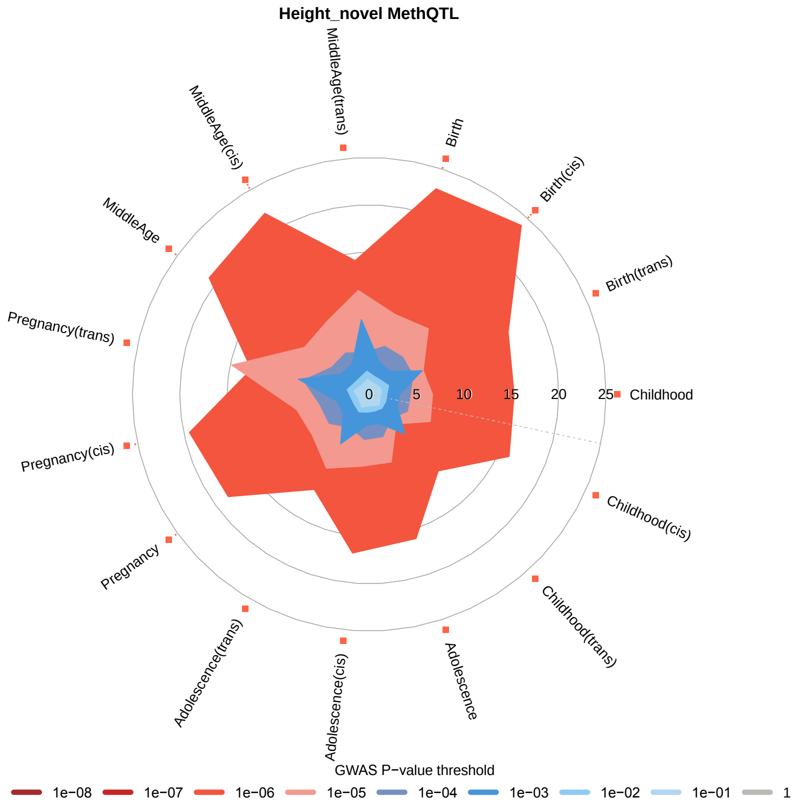

Height_novel MethQTL
MiddleAge(trans)
Birth
Birth(cis)
Birth(trans)
Childhood
Childhood(cis)
Childhood(trans)
Adolescence
Adolescence(cis)
Adolescence(trans)
Pregnancy
Pregnancy(cis)
Pregnancy(trans)
MiddleAge
MiddleAge(cis)
0
5
10
15
20
25
GWAS P−value threshold
1e−08
1e−07
1e−06
1e−05
1e−04
1e−03
1e−02
1e−01
1

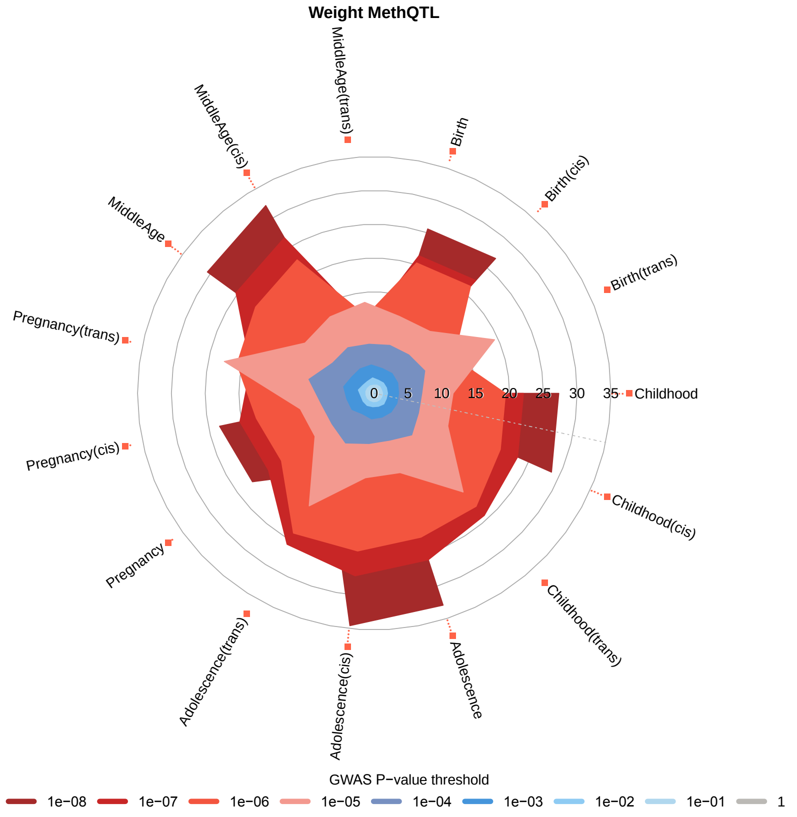

Weight MethQTL
MiddleAge(trans)
Birth
Birth(cis)
Birth(trans)
Childhood
Childhood(cis)
Childhood(trans)
Adolescence
Adolescence(cis)
Adolescence(trans)
Pregnancy
Pregnancy(cis)
Pregnancy(trans)
MiddleAge
MiddleAge(cis)
0
5
10
15
20
25
30
35
GWAS P−value threshold
1e−08
1e−07
1e−06
1e−05
1e−04
1e−03
1e−02
1e−01
1

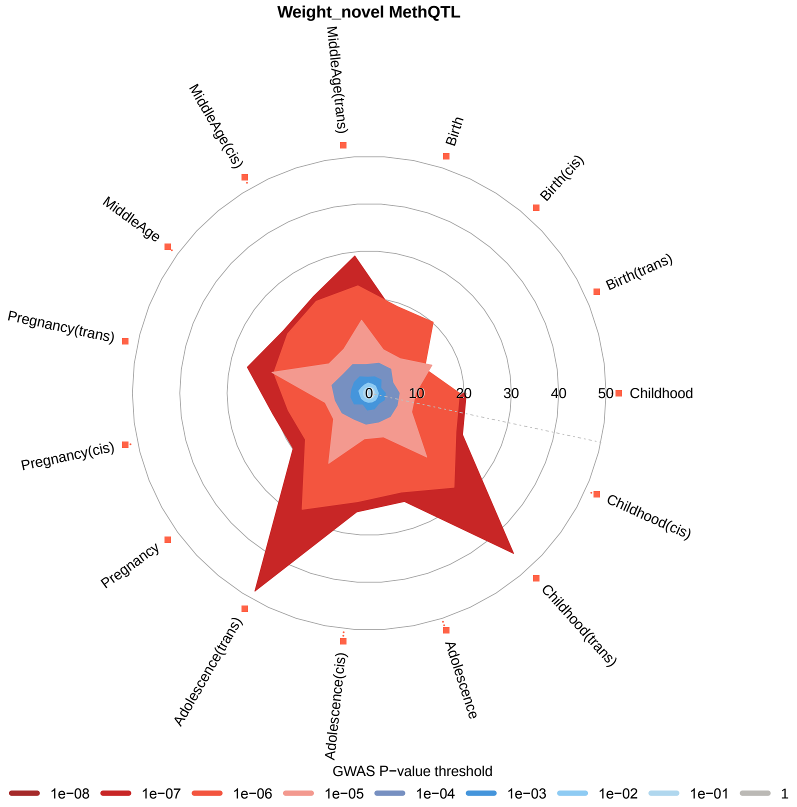

Weight_novel MethQTL
MiddleAge(trans)
Birth
Birth(cis)
Birth(trans)
Childhood
Childhood(cis)
Childhood(trans)
Adolescence
Adolescence(cis)
Adolescence(trans)
Pregnancy
Pregnancy(cis)
Pregnancy(trans)
MiddleAge
MiddleAge(cis)
0
10
20
30
40
50
GWAS P−value threshold
1e−08
1e−07
1e−06
1e−05
1e−04
1e−03
1e−02
1e−01
1

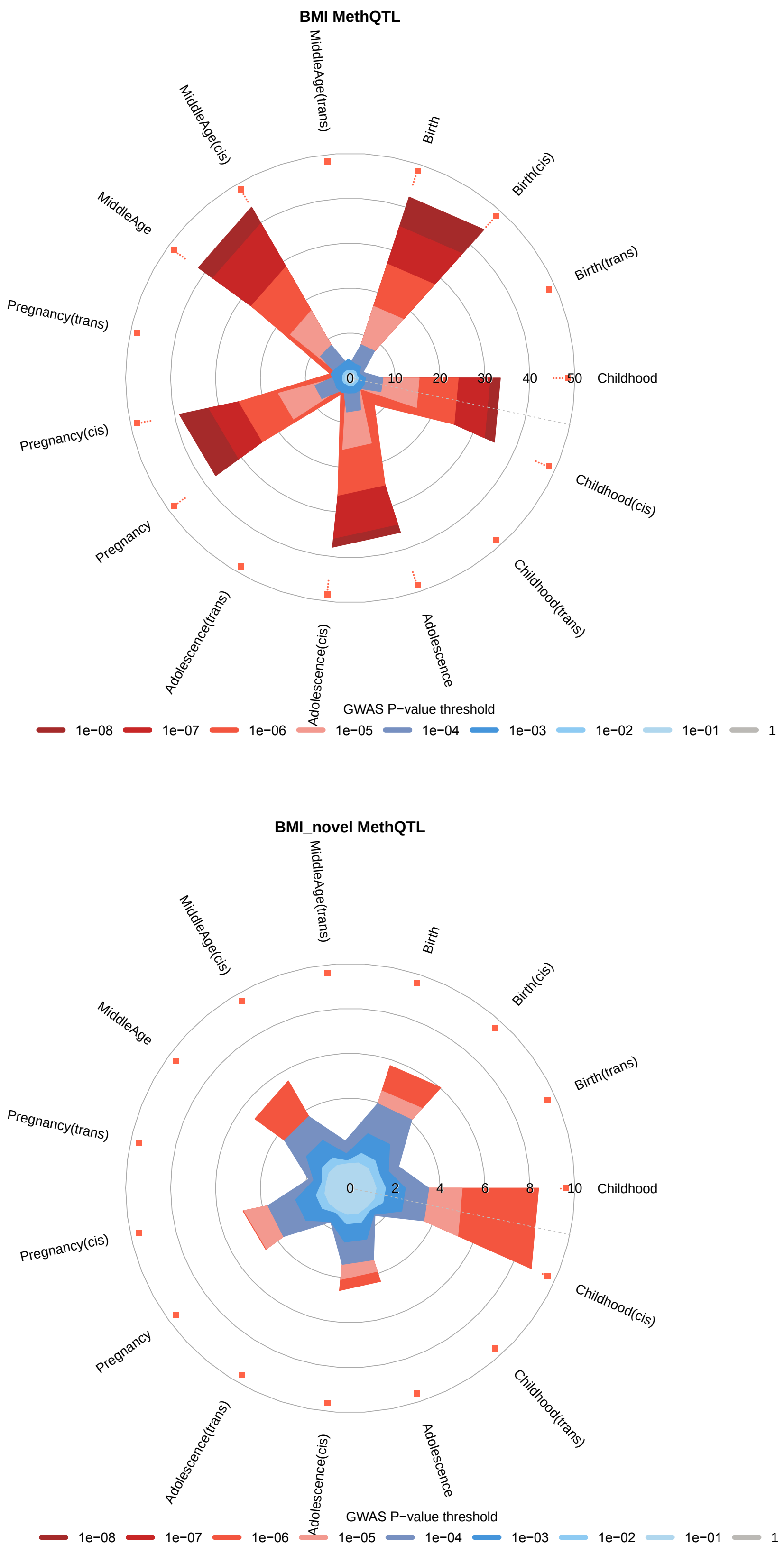

Figure S29: Garfield plots for mQTL enrichment.
Radial plots show the fold enrichment for each time-point where methylation profiles were measured at different GWAS significance thresholds. Small dots on the outer side of the plots show if the observed enrichment is significant for thresholds 10^{-5}, 10^{-6}, 10^{-7}, 10^{-8} in direction from outside to inside.

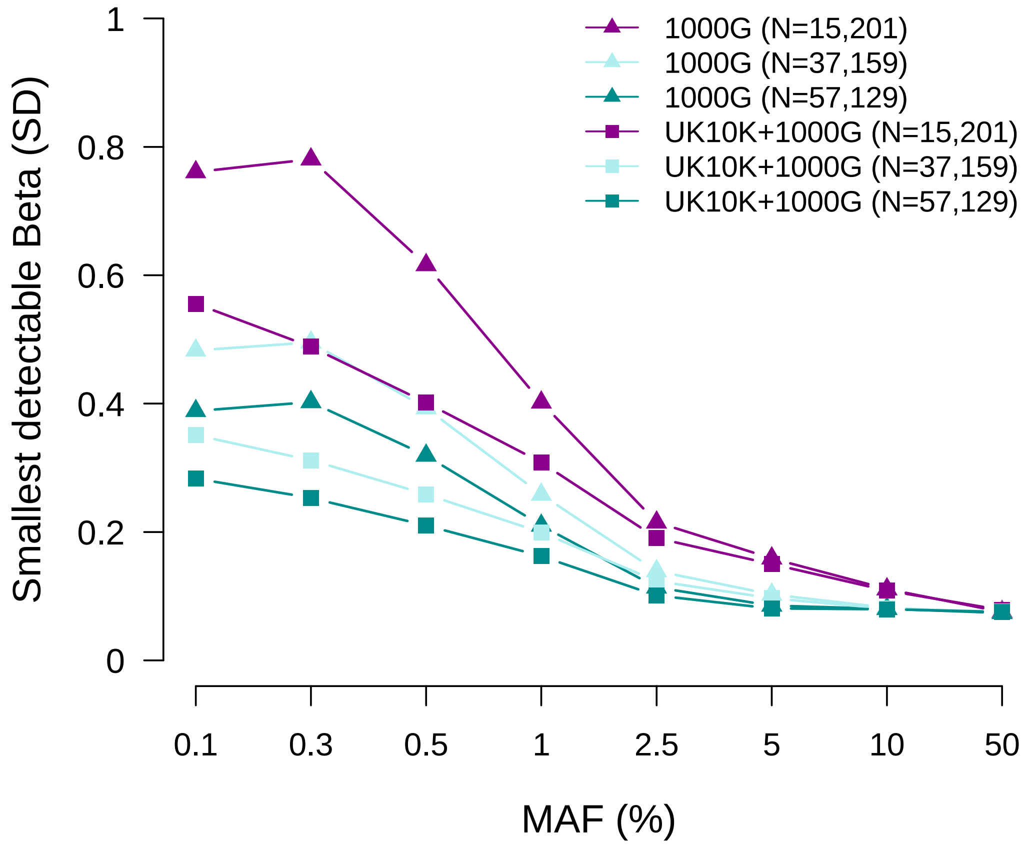

Figure S30: Power benefits due to imputation with UK10K+1000GP compared to 1000GP alone. Strength of single-variant associations detectable at 80% power as a function of Minor Allele Frequency (MAF) and sample size. Using data from chromosome 20, we calculated the smallest value of the strength of association beta (measured in standard deviations), that would be detectable under a linear dosage model at the genome-wide significance threshold ($P<1.85\times10^{-9}$), given the MAF and r^2 of each variant imputable from both the 1000GP and the UK10K+1000GP reference panels, for three representative sample sizes of our discovery stage (N=15,201 representing TFM, TLM, TRFM; N=37,159 representing WHR and hip circumference adjusted/unadjusted for BMI; and N=57,129 representing height, BMI, weight, waist circumference adjusted/unadjusted for BMI). The averages of these minimum detectable beta values by MAF and sample size are shown.

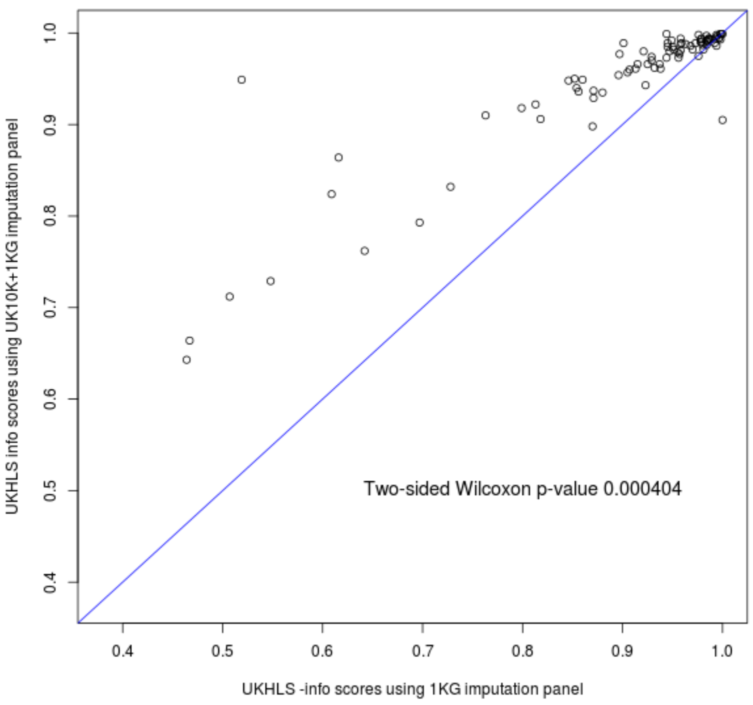

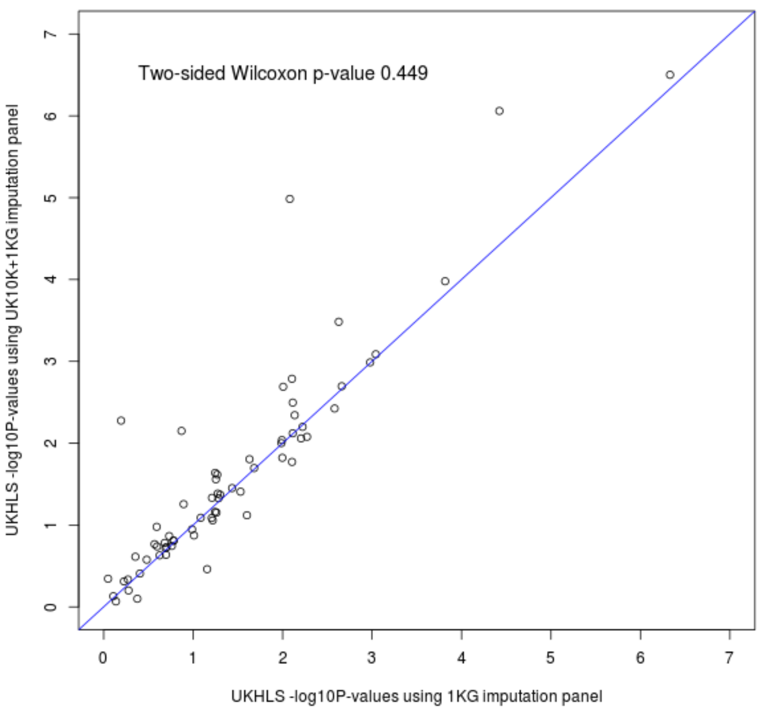

Figure S31: Imputation accuracy scores (top) and *P-values* (bottom) of variants imputed in UKHLS using UK10K+1000GP compared to 1000GP alone.

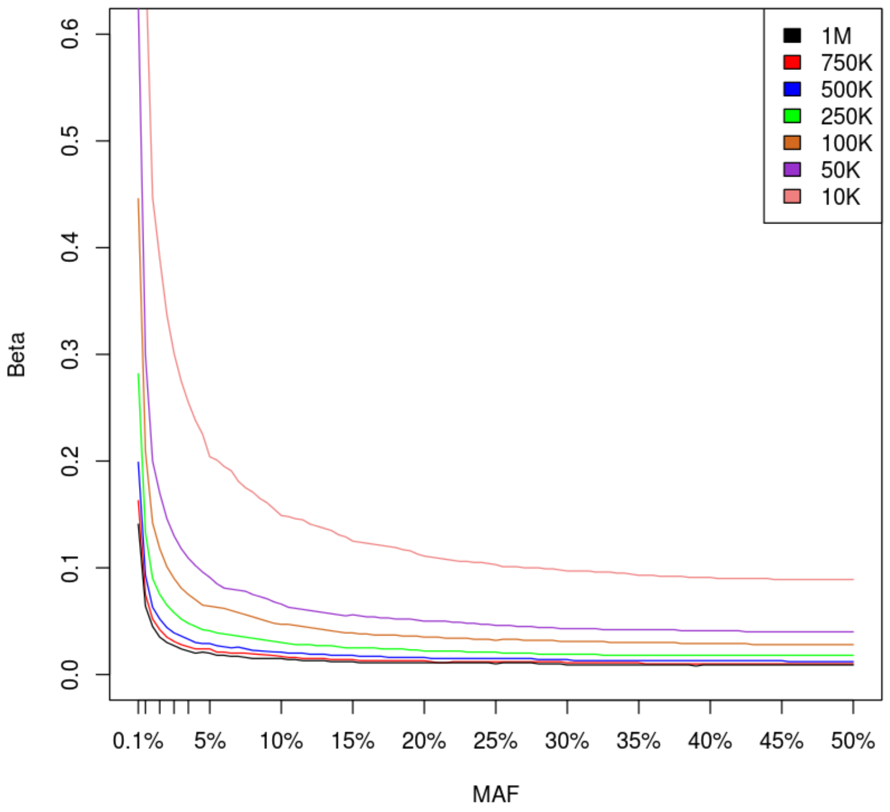

Effect size	MAF											
**Sample size**	**0.001**	**0.01**	**0.05**	**0.1**	**0.15**	**0.2**	**0.25**	**0.3**	**0.35**	**0.4**	**0.45**	**0.5**
**10K**	1.407	0.447	0.204	0.149	0.125	0.111	0.103	0.097	0.093	0.091	0.089	0.089
**50K**	0.63	0.2	0.091	0.066	0.056	0.05	0.046	0.043	0.042	0.041	0.04	0.04
**100K**	0.446	0.142	0.064	0.047	0.039	0.035	0.032	0.031	0.03	0.029	0.028	0.028
**250K**	0.282	0.09	0.041	0.03	0.025	0.022	0.021	0.019	0.019	0.018	0.018	0.018
**500K**	0.199	0.063	0.029	0.021	0.018	0.016	0.015	0.014	0.013	0.013	0.013	0.012
**750K**	0.163	0.052	0.024	0.017	0.014	0.013	0.012	0.011	0.011	0.01	0.01	0.01
**1M**	0.141	0.045	0.02	0.015	0.012	0.011	0.01	0.009	0.009	0.009	0.009	0.009

Figure S32: Effect sizes detectable with 80% power at the genome-wide significance threshold, $P<5\times10^{-8}$, as a function of minor allele frequencies and sample size.

Trait	BMI	Weight	Height	TFM	TLM	TRFM	Waist	Hip	WHR	WaistBMIadj	HipBMIadj	WHRBMIadj
ALSPAC WGS	1791	1812	1794	1683	1683	1683	1807	1808	1806	1785	1786	1784
TwinsUK WGS	1747	1747	1747	1716	1716	1716	1265	1266	1265	1265	1266	1265
ALSPAC GWA	4101	4132	4103	3815	3815	3815	4121	4115	4116	4121	4115	4116
TwinsUK GWA	3539	3539	3540	3275	3275	3275	2585	2582	2582	2583	2580	2580
1958 Birth Cohort	8015	8053	8080	--	--	--	8106	8091	8105	8027	8014	8025
INGI Friuli Venezia Giulia	1170	1172	1197	--	--	--	791	701	791	790	831	790
INCIPE 1	934	933	937	--	--	--	934	--	--	932	--	--
INCIPE 2	2035	2056	2056	--	--	--	2050	--	--	2043	--	--
LURIC	1569	1570	1570	--	--	--	1546	1547	1543	1546	1547	1543
Rotterdam 1	5954	5970	5961	2387	2386	2513	5665	5660	5660	5565	5561	5561
Rotterdam 2	2148	2148	2151	747	747	747	1938	1937	1937	1935	1934	1934
Rotterdam 3	3017	3017	3018	1578	1578	2488	2930	2931	2928	2919	2921	2917
TEENAGE	701	703	703	--	--	--	698	701	697	698	701	697
INGI-Val Borbera	1778	1779	1785	--	--	--	1754	--	--	1754	--	--
INGI Carlantino	472	472	471	--	--	--	397	400	388	397	400	388
HELIC MANOLIS	1019	1051	1043	--	--	--	1060	1050	1053	1005	998	1001
HELIC Pomak	932	942	933	--	--	--	887	883	879	875	871	867
arcOGEN	3908	3923	3925	--	--	--	--	--	--	--	--	--
UKHLS	8560	8620	8700	--	--	--	8727	--	--	8513	--	--
FINRISK	1249	1249	1249	--	--	--	1254	1254	1254	1247	1247	1247
LOLIPOP_EW610	915	916	927	--	--	--	914	916	919	909	909	909
LOLIPOP_EW_A	589	589	589	--	--	--	587	587	587	587	587	587
LOLIPOP_EW_P	650	650	650	--	--	--	649	649	649	649	649	649
**Total discovery (stage 1)**	**56793**	**57043**	**57129**	**15201**	**15200**	**16237**	**50665**	**37078**	**37159**	**50145**	**36917**	**36860**
Fenland	9101	9103	9106	8662	8661	8662	9100	9079	9082	9094	9077	9076
Copenhagen	28710	28736	28745	--	--	--	28687	28668	28677	28643	28631	28632
GenerationR	--	--	--	2008	2015	2005	--	--	--	--	--	--
SardiNIA	6481	6480	6480	--	--	--	6483	6481	6481	6483	6481	6481
GoT2D	32022	--	27544	--	--	--	29328	28680	28686	29320	28678	28684
UK Biobank	134509	134570	134798	--	--	--	134798	134650	134795	134584	134455	134594
**Total follow-up (stage 2)**	**210823**	**178889**	**206673**	**10670**	**10676**	**10667**	**208396**	**207558**	**207721**	**208124**	**207322**	**207467**
**Total discovery + follow-up (stage 1 + stage 2)**	**267616**	**235932**	**263802**	**25871**	**25876**	**26904**	**259061**	**244636**	**244880**	**258269**	**244239**	**244327**

BMI: body mass index; WHR: waist to hip ratio; WaistBMIadj: waist circumference adjusted for BMI; HipBMIadj: hip circumference adjusted for BMI; WHRBMIadj: waist to hip ratio adjusted for BMI; TFM: total fat mass; TLM: total lean mass; TRFM: trunk fat mass

Table S1: Sample sizes for the 12 anthropometric traits studied

Trait	Independent known variants	Same direction of effect	Binomial test *P*	Source of known variants
BMI	97	96	$<2.2x10^{-16}$	Locke et al 2015
Weight	14	14	$6.1x10^{-5}$	Thorleifsson et al 2009
Height	619	610	$<2.2x10^{-16}$	Wood et al 2015
TFM	12	12	$2.44x10^{-4}$	Lu et al 2016
Waist	45	44	$1.31x10^{-12}$	Shungin et al 2015
Hip	63	61	$<2.2x10^{-16}$	Shungin et al 2015
WHR	28	27	$1.08x10^{-7}$	Shungin et al 2015
WaistBMIadj	70	70	$<2.2x10^{-16}$	Shungin et al 2015
HipBMIadj	89	89	$<2.2x10^{-16}$	Shungin et al 2015
WHRBMIadj	39	39	$1.82x10^{-12}$	Shungin et al 2015

BMI: body mass index; WHR: waist to hip ratio; WaistBMIadj: waist circumference adjusted for BMI; HipBMIadj: hip circumference adjusted for BMI; WHRBMIadj: waist to hip ratio adjusted for BMI; TFM: total fat mass; TLM: total lean mass; TRFM: trunk fat mass

Table S13: Comparison of direction of effect between betas from our discovery phase and known loci. There are 28 variants reported for weight in Table 2 of Thorleifsson et al 2009, 14 of which are independent of each other (r^2<0.2 and 500 kb away from each other). From the 697 variants associated with height by Wood et al 2015, we kept the ones that were GWAS significant from the single-point analysis (623 remained) and we further excluded 4 non-distinct signals (r^2>0.2 within 500 kb). For body shape phenotypes, we also took forward independent variants identified in European and sex-combined samples from Shungin et al 2015.

Cohorts	N of duplicate pairs (pi-hat>0.98)	N of related pairs (pi-hat>0.2)	N of total pairs (N1 x N2)
1958 Birth Cohort vs UKBB	40	178	5,847x138,990
arcOGEN vs UKBB	63	194	2,762x138,990
GWAS TwinUK vs UKBB	68	243	3,980x138,990
UKHLS vs UKBB	88	450	9,175x138,990
WGS TwinsUK vs UKBB	43	117	1,754x138,990
Total	302	1,182	3,268,766,820

Table S19: Number of overlapping (pi-hat > 0.98) and related (pi-hat > 0.2) pairs between UK-based cohorts and UK Biobank (UKBB).

	Independent number of suggestive associations	Opposite Direction of Effects with BMI or Height	Proportion (%)	Binomial P-value
WaistBMIadj	146	77	52.74	0.280
HipBMIadj	155	57	36.77	1.000
WHRBMIadj	86	49	56.98	0.118
TFM	79	29	36.71	0.994
TLM	79	40	50.63	0.500
TRFM	82	33	40.24	0.970

WaistBMIadj: waist circumference adjusted for BMI; HipBMIadj: hip circumference adjusted for BMI; WHRBMIadj: waist to hip ratio adjusted for BMI; TFM: total fat mass; TLM: total lean mass; TRFM: trunk fat mass

Table S21: No evidence of collider bias for waist circumference adjusted for BMI analysis. Number of independent (pairwise r^2<0.2 and further than 500kb) variants associated with WaistBMIadj, HipBMIadj, WHRBMIadj, TFM, TLM, TRFM in the discovery meta-analysis with *P-value*<10^{-5}; number and proportion of those variants that had opposite direction of effects for WaistBMIadj, HipBMIadj, WHRBMIadj versus effect sizes for BMI and TFM, TLM, TRFM versus Height; binomial *P-value* of significance.

1. Golding, J., Pembrey, M. & Jones, R. ALSPAC--the Avon Longitudinal Study of Parents and Children. I. Study methodology. *Paediatr Perinat Epidemiol* **15**, 74-87 (2001).
2. Boyd, A. et al. Cohort Profile: The 'Children of the 90s'-the index offspring of the Avon Longitudinal Study of Parents and Children. *International Journal of Epidemiology* **42**, 111-127 (2013).
3. Moayyeri, A., Hammond, C.J., Valdes, A.M. & Spector, T.D. Cohort Profile: TwinsUK and healthy ageing twin study. *Int J Epidemiol* **42**, 76-85 (2013).
4. Walter, K. et al. The UK10K project identifies rare variants in health and disease. *Nature* **526**, 82-90 (2015).
5. Borodulin, K. et al. Forty-year trends in cardiovascular risk factors in Finland. *Eur J Public Health* **25**, 539-46 (2015).
6. Huang, J. et al. Improved imputation of low-frequency and rare variants using the UK10K haplotype reference panel. *Nat Commun* **6**, 8111 (2015).
7. Lynn, P. Sample design for Understanding Society. *Understanding Society Working Paper Series 2009-01* (2009).
8. Hofman, A. et al. The Rotterdam Study: 2016 objectives and design update. *Eur J Epidemiol* **30**, 661-708 (2015).
9. Hofman, A. et al. The Rotterdam Study: 2012 objectives and design update. *European Journal of Epidemiology* **26**, 657-86 (2011).
10. Winkelmann, B.R. et al. Rationale and design of the LURIC study - a resource for functional genomics, pharmacogenomics and long-term prognosis of cardiovascular disease. *Pharmacogenomics* **2**, S7-+ (2001).
11. Power, C. & Elliott, J. Cohort profile: 1958 British Birth Cohort (National Child Development Study). *International Journal of Epidemiology* **35**, 34-41 (2006).
12. Ntalla, I. et al. Body composition and eating behaviours in relation to dieting involvement in a sample of urban Greek adolescents from the TEENAGE (TEENs of Attica: Genes & Environment) study. *Public Health Nutr* **17**, 561-8 (2014).

13. Traglia, M. et al. Heritability and demographic analyses in the large isolated population of Val Borbera suggest advantages in mapping complex traits genes. *PLoS One* **4**, e7554 (2009).
14. Esko, T. et al. Genetic characterization of northeastern Italian population isolates in the context of broader European genetic diversity. *Eur J Hum Genet* **21**, 659-65 (2013).
15. Panoutsopoulou, K. et al. Insights into the genetic architecture of osteoarthritis from stage 1 of the arcOGEN study. *Annals of the Rheumatic Diseases* **70**, 864-867 (2011).
16. Zeggini, E. et al. Identification of new susceptibility loci for osteoarthritis (arcOGEN): a genome-wide association study. *Lancet* **380**, 815-23 (2012).
17. Kooner, J.S. et al. Genome-wide scan identifies variation in MLXIPL associated with plasma triglycerides. *Nat Genet* **40**, 149-51 (2008).
18. Chambers, J.C. et al. Common genetic variation near melatonin receptor MTNR1B contributes to raised plasma glucose and increased risk of type 2 diabetes among Indian Asians and European Caucasians. *Diabetes* **58**, 2703-8 (2009).
19. Sidore, C. et al. Genome sequencing elucidates Sardinian genetic architecture and augments association analyses for lipid and blood inflammatory markers. *Nat Genet* **47**, 1272-81 (2015).
20. Zoledziewska, M. et al. Height-reducing variants and selection for short stature in Sardinia. *Nat Genet* **47**, 1352-6 (2015).
21. Kruithof, C.J. et al. The Generation R Study: Biobank update 2015. *European Journal of Epidemiology* **29**, 911-927 (2014).
22. Jaddoe, V.W. et al. The Generation R Study: design and cohort update 2012. *Eur J Epidemiol* **27**, 739-56 (2012).
23. Gishti, O. et al. Fetal and infant growth patterns associated with total and abdominal fat distribution in school-age children. *J Clin Endocrinol Metab* **99**, 2557-66 (2014).
24. Sudlow, C. et al. UK biobank: an open access resource for identifying the causes of a wide range of complex diseases of middle and old age. *PLoS Med* **12**, e1001779 (2015).
25. Saxena, R. et al. Genome-wide association analysis identifies loci for type 2 diabetes and triglyceride levels. *Science* **316**, 1331-1336 (2007).
26. Guey, L.T. et al. Power in the Phenotypic Extremes: A Simulation Study of Power in Discovery and Replication of Rare Variants. *Genetic Epidemiology* **35**, 236-246 (2011).
27. Lindholm, E., Agardh, E., Tuomi, T., Groop, L. & Agardh, C.D. Classifying diabetes according to the new WHO clinical stages. *European Journal of Epidemiology* **17**, 983-989 (2001).
28. Lyssenko, V. et al. Clinical Risk Factors, DNA Variants, and the Development of Type 2 Diabetes. *New England Journal of Medicine* **359**, 2220-2232 (2008).
29. Scott, L.J. et al. A genome-wide association study of type 2 diabetes in Finns detects multiple susceptibility variants. *Science* **316**, 1341-1345 (2007).
30. Herder, C. et al. RANTES/CCL5 gene polymorphisms, serum concentrations, and incident type 2 diabetes: results from the MONICA/KORA Augsburg case-cohort study, 1984-2002. *European Journal of Endocrinology* **158**, R1-R5 (2008).
31. Huth, C. et al. IL6 gene promoter polymorphisms and type 2 diabetes - Joint analysis of individual participants' data from 21 studies. *Diabetes* **55**, 2915-2921 (2006).
32. Wichmann, H.E., Gieger, C. & Illig, T. KORA-gen--resource for population genetics, controls and a broad spectrum of disease phenotypes. *Gesundheitswesen* **67 Suppl 1**, S26-30 (2005).

33. Maller, J.B. et al. Bayesian refinement of association signals for 14 loci in 3 common diseases. *Nature Genetics* **44**, 1294-1301 (2012).
34. Burton, P.R. et al. Genome-wide association study of 14,000 cases of seven common diseases and 3,000 shared controls. *Nature* **447**, 661-678 (2007).
35. Zeggini, E. et al. Meta-analysis of genome-wide association data and large-scale replication identifies additional susceptibility loci for type 2 diabetes. *Nat Genet* **40**, 638-45 (2008).
36. Leitsalu, L. et al. Cohort Profile: Estonian Biobank of the Estonian Genome Center, University of Tartu. *International Journal of Epidemiology* **44**, 1137-1147 (2015).
37. Stancakova, A. et al. Association of 18 Confirmed Susceptibility Loci for Type 2 Diabetes With Indices of Insulin Release, Proinsulin Conversion, and Insulin Sensitivity in 5,327 Nondiabetic Finnish Men. *Diabetes* **58**, 2129-2136 (2009).
38. Stancakova, A. et al. Effects of 34 Risk Loci for Type 2 Diabetes or Hyperglycemia on Lipoprotein Subclasses and Their Composition in 6,580 Nondiabetic Finnish Men. *Diabetes* **60**, 1608-1616 (2011).
39. Ho, J.E. et al. Clinical and Genetic Correlates of Growth Differentiation Factor 15 in the Community. *Clinical Chemistry* **58**, 1582-1591 (2012).
40. Lind, L., Fors, N., Hall, J., Marttala, K. & Stenborg, A. A comparison of three different methods to evaluate endothelium-dependent vasodilation in the elderly the Prospective Investigation of the Vasculature in Uppsala Seniors (PIVUS) study. *Arteriosclerosis Thrombosis and Vascular Biology* **25**, 2368-2375 (2005).
41. Stefan, N., Fritsche, A. & Haring, H.U. Insulin resistance and congestive heart failure. *Jama-Journal of the American Medical Association* **294**, 2578-2578 (2005).
42. Rolfe, E.D. et al. Association between birth weight and visceral fat in adults. *American Journal of Clinical Nutrition* **92**, 347-352 (2010).
43. Nordestgaard, B.G., Benn, M., Schnohr, P. & Tybjaerg-Hansen, A. Nonfasting triglycerides and risk of myocardial infarction, ischemic heart disease, and death in men and women. *JAMA* **298**, 299-308 (2007).
44. Frikke-Schmidt, R. et al. Association of loss-of-function mutations in the ABCA1 gene with high-density lipoprotein cholesterol levels and risk of ischemic heart disease. *JAMA* **299**, 2524-32 (2008).
45. Relton, C.L. et al. Data Resource Profile: Accessible Resource for Integrated Epigenomic Studies (ARIES). *Int J Epidemiol* (2015).
46. Pidsley, R. et al. A data-driven approach to preprocessing Illumina 450K methylation array data. *BMC Genomics* **14**, 293 (2013).
47. Touleimat, N. & Tost, J. Complete pipeline for Infinium((R)) Human Methylation 450K BeadChip data processing using subset quantile normalization for accurate DNA methylation estimation. *Epigenomics* **4**, 325-41 (2012).
48. Naeem, H. et al. Reducing the risk of false discovery enabling identification of biologically significant genome-wide methylation status using the HumanMethylation450 array. *BMC Genomics* **15**, 51 (2014).
49. Shabalin, A.A. Matrix eQTL: ultra fast eQTL analysis via large matrix operations. *Bioinformatics* **28**, 1353-8 (2012).
50. Houseman, E.A. et al. DNA methylation arrays as surrogate measures of cell mixture distribution. *BMC Bioinformatics* **13**, 86 (2012).
51. The GTEx Consortium. The Genotype-Tissue Expression (GTEx) pilot analysis: multitissue gene regulation in humans. *Science* **348**, 648-60 (2015).

52. Felicity Payne, R.C., Nuno Rocha, Asha Seth, Julie Harris, Gillian Carpenter, William E. Bottomley, Eleanor Wheeler, Stephen Wong, Vladimir Saudek, David Savage, Stephen O'Rahilly, Jean-Claude Carel, Inês Barroso, Mark O'Driscoll, Robert Semple. Hypomorphism in human NSMCE2 linked to primordial dwarfism and insulin resistance. *The Journal of Clinical Investigation* **124**, 4028–4038 (2014).
53. Davydov, E.V. et al. Identifying a high fraction of the human genome to be under selective constraint using GERP++. *PLoS Comput Biol* **6**, e1001025 (2010).
54. Roadmap Epigenomics, C. et al. Integrative analysis of 111 reference human epigenomes. *Nature* **518**, 317-30 (2015).
55. Flicek, P. et al. Ensembl 2014. *Nucleic Acids Res* **42**, D749-55 (2014).
56. Boyle, A.P. et al. Annotation of functional variation in personal genomes using RegulomeDB. *Genome Res* **22**, 1790-7 (2012).
57. UniProt: a hub for protein information. *Nucleic Acids Res* **43**, D204-12 (2015).
58. Lindskog, C. The potential clinical impact of the tissue-based map of the human proteome. *Expert Rev Proteomics* **12**, 213-5 (2015).
59. Sarrias, M.R. et al. The Scavenger Receptor Cysteine-Rich (SRCR) domain: an ancient and highly conserved protein module of the innate immune system. *Crit Rev Immunol* **24**, 1-37 (2004).
60. Delaunay, A. et al. The ER-bound RING finger protein 5 (RNF5/RMA1) causes degenerative myopathy in transgenic mice and is deregulated in inclusion body myositis. *Plos One* **3**, e1609 (2008).
61. Concolino, P. et al. p.H282N and p.Y191H: 2 novel CYP21A2 mutations in Italian congenital adrenal hyperplasia patients. *Metabolism* **61**, 519-24 (2012).
62. Bolton, J.L. et al. Genome Wide Association Identifies Common Variants at the SERPINA6/SERPINA1 Locus Influencing Plasma Cortisol and Corticosteroid Binding Globulin. *Plos Genetics* **10**(2014).
63. Noakes, P.G. et al. The renal glomerulus of mice lacking s-laminin/laminin beta 2: nephrosis despite molecular compensation by laminin beta 1. *Nature Genetics* **10**, 400-6 (1995).
64. Wood, A.R. et al. Defining the role of common variation in the genomic and biological architecture of adult human height. *Nat Genet* **46**, 1173-86 (2014).
65. Cottle, D.L. et al. FHL3 binds MyoD and negatively regulates myotube formation. *J Cell Sci* **120**, 1423-35 (2007).
66. Kim, H.K. et al. Lowe syndrome: a single center's experience in Korea. *Korean J Pediatr* **57**, 140-8 (2014).
67. Eriksson, N. et al. Novel associations for hypothyroidism include known autoimmune risk loci. *Plos One* **7**, e34442 (2012).
68. Yang, Y., Topol, L., Lee, H. & Wu, J. Wnt5a and Wnt5b exhibit distinct activities in coordinating chondrocyte proliferation and differentiation. *Development* **130**, 1003-15 (2003).
69. Roifman, M. et al. De novo WNT5A-associated autosomal dominant Robinow syndrome suggests specificity of genotype and phenotype. *Clin Genet* **87**, 34-41 (2015).
70. Yamaguchi, T.P., Bradley, A., McMahon, A.P. & Jones, S. A Wnt5a pathway underlies outgrowth of multiple structures in the vertebrate embryo. *Development* **126**, 1211-23 (1999).

71. Koscielny, G. et al. The International Mouse Phenotyping Consortium Web Portal, a unified point of access for knockout mice and related phenotyping data. *Nucleic Acids Res* **42**, D802-9 (2014).
72. Provot, S. et al. Nkx3.2/Bapx1 acts as a negative regulator of chondrocyte maturation. *Development* **133**, 651-662 (2006).
73. Hellemans, J. et al. Homozygous inactivating mutations in the NKX3-2 gene result in spondylo-megaepiphyseal-metaphyseal dysplasia. *Am J Hum Genet* **85**, 916-22 (2009).
74. Rodriguez, J.M. et al. APPRIS: annotation of principal and alternative splice isoforms. *Nucleic Acids Research* **41**, D110-D117 (2013).
75. Zerbino, D.R. et al. Ensembl regulation resources. *Database (Oxford)* **2016**(2016).
76. Sanford LP, O.I., Gittenberger-de Groot AC, Sariola H, Friedman R, Boivin GP, Cardell EL, Doetschman T. TGFbeta2 knockout mice have multiple developmental defects that are non-overlapping with other TGFbeta knockout phenotypes. *Development.* **124**, 2659-70 (1997).
77. Lango Allen, H. et al. Hundreds of variants clustered in genomic loci and biological pathways affect human height. *Nature* **467**, 832-8 (2010).
78. He, M. et al. Meta-analysis of genome-wide association studies of adult height in East Asians identifies 17 novel loci. *Hum Mol Genet* **24**, 1791-800 (2015).
79. Velinov, M. et al. Limb-Girdle Muscular-Dystrophy Is Closely Linked to the Fibrillin Locus on Chromosome-15. *Connective Tissue Research* **29**, 13-21 (1993).
80. Boileau, C. et al. Autosomal-Dominant Marfan-Like Connective-Tissue Disorder with Aortic Dilation and Skeletal Anomalies Not Linked to the Fibrillin Genes. *American Journal of Human Genetics* **53**, 46-54 (1993).
81. Faivre, L. et al. In frame fibrillin-1 gene deletion in autosomal dominant Weill-Marchesani syndrome. *Journal of Medical Genetics* **40**, 34-36 (2003).
82. Uhlen, M. et al. Proteomics. Tissue-based map of the human proteome. *Science* **347**, 1260419 (2015).
83. Loewith, R. et al. Two TOR complexes, only one of which is rapamycin sensitive, have distinct roles in cell growth control. *Molecular Cell* **10**, 457-468 (2002).
84. Guertin, D.A. et al. Ablation in mice of the mTORC components raptor, rictor, or mLST8 reveals that mTORC2 is required for signaling to Akt-FOXO and PKC alpha but not S6K1. *Developmental Cell* **11**, 859-871 (2006).
85. Le Cam, L., Lacroix, M., Ciemerych, M.A., Sardet, C. & Sicinski, P. The E4F protein is required for mitotic progression during embryonic cell cycles. *Molecular and Cellular Biology* **24**, 6467-6475 (2004).
86. Harsay, E. & Schekman, R. Avl9p, a member of a novel protein superfamily, functions in the late secretory pathway. *Mol Biol Cell* **18**, 1203-19 (2007).
87. Mallon, A.M., Blake, A. & Hancock, J.M. EuroPhenome and EMPReSS: online mouse phenotyping resource. *Nucleic Acids Res* **36**, D715-8 (2008).
88. Ito, Y. et al. The Mohawk homeobox gene is a critical regulator of tendon differentiation. *Proceedings of the National Academy of Sciences of the United States of America* **107**, 10538-10542 (2010).
89. Zerbino, D.R., Wilder, S.P., Johnson, N., Juettemann, T. & Flicek, P.R. The ensembl regulatory build. *Genome Biol* **16**, 56 (2015).
90. Unger, S., Scherer, G. & Superti-Furga, A. Campomelic Dysplasia. (1993).
91. Sun, L. et al. Epigenetic regulation of SOX9 by the NF-kappaB signaling pathway in pancreatic cancer stem cells. *Stem Cells* **31**, 1454-66 (2013).

92. Altarejos, J.Y. et al. The Creb1 coactivator Crtc1 is required for energy balance and fertility. *Nature Medicine* **14**, 1112-1117 (2008).
93. Zhong, J. et al. Temporal profiling of the secretome during adipogenesis in humans. *J Proteome Res* **9**, 5228-38 (2010).
94. Pinnick, K.E. et al. Distinct developmental profile of lower-body adipose tissue defines resistance against obesity-associated metabolic complications. *Diabetes* **63**, 3785-97 (2014).
95. Dorflinger, U. et al. Activation of somatostatin receptor II expression by transcription factors MIBP1 and SEF-2 in the murine brain. *Mol Cell Biol* **19**, 3736-47 (1999).
96. Jin, W. et al. Schnurri-2 controls BMP-dependent adipogenesis via interaction with Smad proteins. *Dev Cell* **10**, 461-71 (2006).
97. Gudbjartsson, D.F. et al. Many sequence variants affecting diversity of adult human height. *Nat Genet* **40**, 609-15 (2008).
98. Wade, T.D. et al. Genetic variants associated with disordered eating. *Int J Eat Disord* **46**, 594-608 (2013).
99. Berendsen, A.D. & Olsen, B.R. Bone development. *Bone* **80**, 14-8 (2015).
100. Gurnett, C.A. et al. Asymmetric lower-limb malformations in individuals with homeobox PITX1 gene mutation. *Am J Hum Genet* **83**, 616-22 (2008).
101. Spielmann, M. et al. Homeotic Arm-to-Leg Transformation Associated with Genomic Rearrangements at the PITX1 Locus. *American Journal of Human Genetics* **91**, 629-635 (2012).
102. Szeto, D.P. et al. Role of the Bicoid-related homeodomain factor Pitx1 in specifying hindlimb in morphogenesis and pituitary development. *Genes & Development* **13**, 484-494 (1999).
103. van de Laar, I.M. et al. Mutations in SMAD3 cause a syndromic form of aortic aneurysms and dissections with early-onset osteoarthritis. *Nature Genetics* **43**, 121-6 (2011).
104. Yang, X. et al. TGF-beta/Smad3 signals repress chondrocyte hypertrophic differentiation and are required for maintaining articular cartilage. *J Cell Biol* **153**, 35-46 (2001).
105. Li, F.F. et al. Characterization of SMAD3 Gene Variants for Possible Roles in Ventricular Septal Defects and Other Congenital Heart Diseases. *Plos One* **10**, e0131542 (2015).
106. Qian, F. et al. Cleavage of polycystin-1 requires the receptor for egg jelly domain and is disrupted by human autosomal-dominant polycystic kidney disease 1-associated mutations. *Proceedings of the National Academy of Sciences of the United States of America* **99**, 16981-16986 (2002).
107. Jeffery, S., Saggar-Malik, A.K., Economides, D.L., Blackmore, S.E. & MacDermot, K.D. Apparent normalisation of fetal renal size in autosomal dominant polycystic kidney disease (PKD1). *Clin Genet* **53**, 303-7 (1998).
108. Jiang, S.T. et al. Defining a link with autosomal-dominant polycystic kidney disease in mice with congenitally low expression of Pkd1. *Am J Pathol* **168**, 205-20 (2006).
109. Ritchie, G.R., Dunham, I., Zeggini, E. & Flicek, P. Functional annotation of noncoding sequence variants. *Nat Methods* **11**, 294-6 (2014).
110. Aschard, H., Vilhjalmsson, B.J., Joshi, A.D., Price, A.L. & Kraft, P. Adjusting for Heritable Covariates Can Bias Effect Estimates in Genome-Wide Association Studies. *American Journal of Human Genetics* **96**, 329-339 (2015).
111. Halbig, K.M., Lekven, A.C. & Kunkel, G.R. The transcriptional activator ZNF143 is essential for normal development in zebrafish. *Bmc Molecular Biology* **13**(2012).

112. Shungin, D. et al. New genetic loci link adipose and insulin biology to body fat distribution. *Nature* **518**, 187-96 (2015).
113. Garbe, A.I. et al. Regulation of bone mass and osteoclast function depend on the F-actin modulator SWAP-70. *Journal of Bone and Mineral Research* **27**, 2085-96 (2012).
114. Duivenvoorde, L.P., van Schothorst, E.M., Bunschoten, A. & Keijer, J. Dietary restriction of mice on a high-fat diet induces substrate efficiency and improves metabolic health. *J Mol Endocrinol* **47**, 81-97 (2011).
115. Cunningham, F. et al. Ensembl 2015. *Nucleic Acids Res* **43**, D662-9 (2015).
116. Barrow, J.R. & Capecchi, M.R. Targeted disruption of the Hoxb-2 locus in mice interferes with expression of Hoxb-1 and Hoxb-4. *Development* **122**, 3817-28 (1996).
117. Manley, N.R., Barrow, J.R., Zhang, T. & Capecchi, M.R. Hoxb2 and hoxb4 act together to specify ventral body wall formation. *Dev Biol* **237**, 130-44 (2001).
118. Yamada, T. et al. SRC-1 is necessary for skeletal responses to sex hormones in both males and females. *Journal of Bone and Mineral Research* **19**, 1452-1461 (2004).
119. Li, Y. et al. ZNF322, a novel human C2H2 Kruppel-like zinc-finger protein, regulates transcriptional activation in MAPK signaling pathways. *Biochem Biophys Res Commun* **325**, 1383-92 (2004).
120. Grohmann, K. et al. Mutations in the gene encoding immunoglobulin mu-binding protein 2 cause spinal muscular atrophy with respiratory distress type 1. *Nature Genetics* **29**, 75-7 (2001).
